# Supplementary material for: Identification of Biochemical Pathways Responsible for Distinct Phenotypes Using Gene Ontology Causal Activity Models
Source: bioRxiv. 2023 May 25:2023.05.22.541760. Preprint. [Version 2] doi: 10.1101/2023.05.22.541760 (PMC10245817; doi:10.1101/2023.05.22.541760)
Supplement: Supplementary file 1 [file media-1.pdf]

Genes Used		shared
glycolysis	gluconeogenesis	shared
Gck (MGI:1270854)	*G6pc* (MGI:95607)	*Gpi1* (MGI:95797)
Hk1 (MGI:96103)	*G6pc2* (MGI:1277193)	*Aldoa* (MGI:87994)
Hk2 (MGI:1315197)	*G6pc3* (MGI:1915651)	*Aldob* (MGI:87995)
Hk3 (MGI:2670962)	*Slc37a1* (MGI:2446181)	*Aldoc* (MGI:101863)
Hkdc1 (MGI:238491	*Slc37a2* (MGI:1929693)	*Tpi1* (MGI:98797)
Pfkm (MGI:97548)	*Slc37a4* (MGI:1316650)	*Gapdh* (MGI:95640)
Pfkp (MGI:1891833)	*Fbp1* (MGI:95492)	*Gapdhs* (MGI:95653)
Pfkl (MGI:97547)	*Fbp2* (MGI:95491)	*Pgk1* (MGI:97555)
Pkm (MGI:97591)	*Pck1* (MGI:97501)	*Pgk2* (MGI:97563)
Pklr (MGI:97604)	*Pck2* (MGI:1860456)	*Pgam1* (MGI:97552)
	Pcx (MGI:97520)	*Pgam2* (MGI:1933118)
	Slc25a10 (MGI:1353497)	*Eno1* (MGI:95393)
	Slc25a11 (MGI:1915113)	*Eno1b* (MGI:3648653)
	Slc25a12 (MGI:1926080)	*Eno2* (MGI:95394)
	Slc25a13 (MGI:1354721)	*Eno3* (MGI:95395)
	Got1 (MGI:95791)	*Eno4* (MGI:2441717)
	Got2 (MGI:95792)	
	Mdh2 (MGI:97050)	
	Mdh1 (MGI:97051)	
	not annotated to a phenotype	

TermID	Term	Term Min P	Term Max P	Term P ratio	P	Q	k	n	K	N	k/n	K/N	k/K	n/N	Qset	Symbols
					1.68E-09	9.96E-07	6	19	139	21447	31.58%	0.65%	4.32%	0.09%	gluconeogenesis Jan 31	Fbp1, G6pc, Pck1, Pcx, Slc25a13, Slc37a4
					5.69E-02	1.27E-01	1	9	139	21447	11.11%	0.65%	0.72%	0.04%	glycolysis Jan 31	Gck
MP:0000189	hypoglycemia	1.68E-09	1.00E+00	1.68E-09	1.00E+00	1.00E+00	0	11	139	21447	0.00%	0.65%	0.00%	0.05%	shared jan 31	
					8.36E-08	2.47E-05	8	19	719	21447	42.11%	3.35%	1.11%	0.09%	gluconeogenesis Jan 31	Fbp1, G6pc, G6pc2, Got1, Pck1, Pcx, Slc25a13, Slc37a4
					2.64E-01	3.29E-01	1	9	719	21447	11.11%	3.35%	0.14%	0.04%	glycolysis Jan 31	Gck
MP:0005560	decreased circulating glucose level	8.36E-08	1.00E+00	8.36E-08	1.00E+00	1.00E+00	0	11	719	21447	0.00%	3.35%	0.00%	0.05%	shared jan 31	
					2.61E-06	5.14E-04	7	19	771	21447	36.84%	3.59%	0.91%	0.09%	gluconeogenesis Jan 31	G6pc, G6pc2, Pck1, Pck2, Pcx, Slc25a13, Slc37a4
					3.93E-02	1.10E-01	2	9	771	21447	22.22%	3.59%	0.26%	0.04%	glycolysis Jan 31	Gck, Hkdc1
MP:0000187	abnormal triglyceride level	2.61E-06	1.00E+00	2.61E-06	1.00E+00	1.00E+00	0	11	771	21447	0.00%	3.59%	0.00%	0.05%	shared jan 31	
					4.46E-06	5.28E-04	2	19	4	21447	10.53%	0.02%	50.00%	0.09%	gluconeogenesis Jan 31	G6pc, Slc37a4
					1.00E+00	1.00E+00	0	9	4	21447	0.00%	0.02%	0.00%	0.04%	glycolysis Jan 31	
MP:0031002	abnormal kidney glycogen level	4.46E-06	1.00E+00	4.46E-06	1.00E+00	1.00E+00	0	11	4	21447	0.00%	0.02%	0.00%	0.05%	shared jan 31	
					4.46E-06	5.28E-04	2	19	4	21447	10.53%	0.02%	50.00%	0.09%	gluconeogenesis Jan 31	G6pc, Slc37a4
					1.00E+00	1.00E+00	0	9	4	21447	0.00%	0.02%	0.00%	0.04%	glycolysis Jan 31	
MP:0031003	increased kidney glycogen level	4.46E-06	1.00E+00	4.46E-06	1.00E+00	1.00E+00	0	11	4	21447	0.00%	0.02%	0.00%	0.05%	shared jan 31	
					6.99E-06	6.31E-04	4	19	144	21447	21.05%	0.67%	2.78%	0.09%	gluconeogenesis Jan 31	G6pc, Pck1, Pcx, Slc25a13
					1.00E+00	1.00E+00	0	9	144	21447	0.00%	0.67%	0.00%	0.04%	glycolysis Jan 31	
MP:0009355	increased liver triglyceride level	6.99E-06	1.00E+00	6.99E-06	1.00E+00	1.00E+00	0	11	144	21447	0.00%	0.67%	0.00%	0.05%	shared jan 31	
					7.77E-06	6.31E-04	8	19	1309	21447	42.11%	6.10%	0.61%	0.09%	gluconeogenesis Jan 31	Fbp1, G6pc, G6pc2, Got1, Pck1, Pcx, Slc25a13, Slc37a4
					1.01E-01	1.82E-01	2	9	1309	21447	22.22%	6.10%	0.15%	0.04%	glycolysis Jan 31	Gck, Pfkm
MP:0000188	abnormal circulating glucose level	7.77E-06	5.00E-01	1.55E-05	5.00E-01	5.73E-01	1	11	1309	21447	9.09%	6.10%	0.08%	0.05%	shared jan 31	Gpi1
					1.00E+00	1.00E+00	0	19	99	21447	0.00%	0.46%	0.00%	0.09%	gluconeogenesis Jan 31	
					7.85E-06	1.94E-03	3	9	99	21447	33.33%	0.46%	3.03%	0.04%	glycolysis Jan 31	Hk1, Pfkm, Pklr
MP:0002640	reticulocytosis	7.85E-06	1.00E+00	7.85E-06	1.13E-03	3.47E-02	2	11	99	21447	18.18%	0.46%	2.02%	0.05%	shared jan 31	Gpi1, Tpi1
					8.53E-06	6.31E-04	6	19	591	21447	31.58%	2.76%	1.02%	0.09%	gluconeogenesis Jan 31	G6pc, G6pc2, Pck1, Pck2, Pcx, Slc37a4
					2.22E-01	2.91E-01	1	9	591	21447	11.11%	2.76%	0.17%	0.04%	glycolysis Jan 31	Gck
MP:0011969	abnormal circulating triglyceride level	8.53E-06	1.00E+00	8.53E-06	1.00E+00	1.00E+00	0	11	591	21447	0.00%	2.76%	0.00%	0.05%	shared jan 31	
					1.00E+00	1.00E+00	0	19	113	21447	0.00%	0.53%	0.00%	0.09%	gluconeogenesis Jan 31	
					1.17E-05	1.94E-03	3	9	113	21447	33.33%	0.53%	2.65%	0.04%	glycolysis Jan 31	Hk1, Pfkm, Pklr
MP:0011913	abnormal reticulocyte cell number	1.17E-05	1.00E+00	1.17E-05	1.47E-03	3.47E-02	2	11	113	21447	18.18%	0.53%	1.77%	0.05%	shared jan 31	Gpi1, Tpi1
					1.00E+00	1.00E+00	0	19	120	21447	0.00%	0.56%	0.00%	0.09%	gluconeogenesis Jan 31	
					1.40E-05	1.94E-03	3	9	120	21447	33.33%	0.56%	2.50%	0.04%	glycolysis Jan 31	Hk1, Pfkm, Pklr
MP:0002424	abnormal reticulocyte morphology	1.40E-05	1.00E+00	1.40E-05	1.65E-03	3.47E-02	2	11	120	21447	18.18%	0.56%	1.67%	0.05%	shared jan 31	Gpi1, Tpi1
					2.63E-05	1.73E-03	9	19	2065	21447	47.37%	9.63%	0.44%	0.09%	gluconeogenesis Jan 31	Fbp1, G6pc, G6pc2, G6pc3, Got1, Pck1, Pcx, Slc25a13, Slc37a4
					4.81E-02	1.16E-01	3	9	2065	21447	33.33%	9.63%	0.15%	0.04%	glycolysis Jan 31	Gck, Hkdc1, Pfkm
MP:0002078	abnormal glucose homeostasis	2.63E-05	6.72E-01	3.92E-05	6.72E-01	7.05E-01	1	11	2065	21447	9.09%	9.63%	0.05%	0.05%	shared jan 31	Gpi1
					1.00E+00	1.00E+00	0	19	20	21447	0.00%	0.09%	0.00%	0.09%	gluconeogenesis Jan 31	
					2.96E-05	3.08E-03	2	9	20	21447	22.22%	0.09%	10.00%	0.04%	glycolysis Jan 31	Pfkm, Pklr
MP:0031085	increased erythrocyte osmotic fragility	2.96E-05	1.00E+00	2.96E-05	1.00E+00	1.00E+00	0	11	20	21447	0.00%	0.09%	0.00%	0.05%	shared jan 31	
					4.11E-05	2.43E-03	5	19	462	21447	26.32%	2.15%	1.08%	0.09%	gluconeogenesis Jan 31	G6pc, Pck1, Pcx, Slc25a13, Slc37a4
					1.78E-01	2.56E-01	1	9	462	21447	11.11%	2.15%	0.22%	0.04%	glycolysis Jan 31	Gck
MP:0005317	increased triglyceride level	4.11E-05	1.00E+00	4.11E-05	1.00E+00	1.00E+00	0	11	462	21447	0.00%	2.15%	0.00%	0.05%	shared jan 31	
					1.56E-01	3.16E-01	1	19	190	21447	5.26%	0.89%	0.53%	0.09%	gluconeogenesis Jan 31	Slc37a1
					5.53E-05	4.60E-03	3	9	190	21447	33.33%	0.89%	1.58%	0.04%	glycolysis Jan 31	Hk1, Pfkm, Pklr
MP:0005344	increased circulating bilirubin level	5.53E-05	1.56E-01	3.55E-04	4.07E-03	5.87E-02	2	11	190	21447	18.18%	0.89%	1.05%	0.05%	shared jan 31	Gpi1, Tpi1
					5.72E-05	3.08E-03	4	19	246	21447	21.05%	1.15%	1.63%	0.09%	gluconeogenesis Jan 31	G6pc, Pck1, Pcx, Slc25a13
					9.86E-02	1.82E-01	1	9	246	21447	11.11%	1.15%	0.41%	0.04%	glycolysis Jan 31	Hkdc1
MP:0012778	abnormal liver triglyceride level	5.72E-05	1.00E+00	5.72E-05	1.00E+00	1.00E+00	0	11	246	21447	0.00%	1.15%	0.00%	0.05%	shared jan 31	
					1.65E-01	3.18E-01	1	19	203	21447	5.26%	0.95%	0.49%	0.09%	gluconeogenesis Jan 31	Slc37a1
					6.73E-05	4.67E-03	3	9	203	21447	33.33%	0.95%	1.48%	0.04%	glycolysis Jan 31	Hk1, Pfkm, Pklr
MP:0001569	abnormal circulating bilirubin level	6.73E-05	1.65E-01	4.07E-04	4.64E-03	6.35E-02	2	11	203	21447	18.18%	0.95%	0.99%	0.05%	shared jan 31	Gpi1, Tpi1
					1.00E+00	1.00E+00	0	19	33	21447	0.00%	0.15%	0.00%	0.09%	gluconeogenesis Jan 31	
					8.21E-05	4.88E-03	2	9	33	21447	22.22%	0.15%	6.06%	0.04%	glycolysis Jan 31	Pfkm, Pklr
MP:0003657	abnormal erythrocyte osmotic lysis	8.21E-05	1.00E+00	8.21E-05	1.68E-02	1.02E-01	1	11	33	21447	9.09%	0.15%	3.03%	0.05%	shared jan 31	Gpi1
					9.01E-05	4.44E-03	8	19	1834	21447	42.11%	8.55%	0.44%	0.09%	gluconeogenesis Jan 31	G6pc, G6pc2, G6pc3, Pck1, Pck2, Pcx, Slc25a13, Slc37a4
					1.76E-01	2.55E-01	2	9	1834	21447	22.22%	8.55%	0.11%	0.04%	glycolysis Jan 31	Gck, Hkdc1
MP:0001547	abnormal lipid level	9.01E-05	1.00E+00	9.01E-05	1.00E+00	1.00E+00	0	11	1834	21447	0.00%	8.55%	0.00%	0.05%	shared jan 31	
					1.00E+00	1.00E+00	0	19	513	21447	0.00%	2.39%	0.00%	0.09%	gluconeogenesis Jan 31	
					1.96E-01	2.72E-01	1	9	513	21447	11.11%	2.39%	0.19%	0.04%	glycolysis Jan 31	Hk2
MP:0009850	embryonic lethality between implantation and placentation	9.34E-05	1.00E+00	9.34E-05	9.34E-05	2.65E-02	4	11	513	21447	36.36%	2.39%	0.78%	0.05%	shared jan 31	Eno1, Gapdh, Gpi1, Tpi1
					1.00E+00	1.00E+00	0	19	38	21447	0.00%	0.18%	0.00%	0.09%	gluconeogenesis Jan 31	
					1.09E-04	5.68E-03	2	9	38	21447	22.22%	0.18%	5.26%	0.04%	glycolysis Jan 31	Pfkm, Pklr
MP:0010163	hemolysis	1.09E-04	1.00E+00	1.09E-04	1.93E-02	1.11E-01	1	11	38	21447	9.09%	0.18%	2.63%	0.05%	shared jan 31	Gpi1
					1.23E-04	5.28E-03	3	19	111	21447	15.79%	0.52%	2.70%	0.09%	gluconeogenesis Jan 31	G6pc, Pck1, Slc37a4
					4.56E-02	1.14E-01	1	9	111	21447	11.11%	0.52%	0.90%	0.04%	glycolysis Jan 31	Gck
MP:0012777	abnormal liver glycogen level	1.23E-04	1.00E+00	1.23E-04	1.00E+00	1.00E+00	0	11	111	21447	0.00%	0.52%	0.00%	0.05%	shared jan 31	

									1.25E-04	5.28E-03	4	19	301	21447	21.05%	1.40%	1.33%	0.09%	gluconeogenesis Jan 31	G6pc, Pck1, Pcx, Slc25a13
									1.19E-01	1.97E-01	1	9	301	21447	11.11%	1.40%	0.33%	0.04%	glycolysis Jan 31	Gck
				MP:0002628	hepatic steatosis	1.25E-04	1.44E-01	8.67E-04	1.44E-01	3.26E-01	1	11	301	21447	9.09%	1.40%	0.33%	0.05%	shared jan 31	Aldob
									1.00E+00	1.00E+00	0	19	42	21447	0.00%	0.20%	0.00%	0.09%	gluconeogenesis Jan 31	
									1.34E-04	6.18E-03	2	9	42	21447	22.22%	0.20%	4.76%	0.04%	glycolysis Jan 31	Hk1, Pklr
				MP:0001585	hemolytic anemia	1.34E-04	1.00E+00	1.34E-04	2.13E-02	1.12E-01	1	11	42	21447	9.09%	0.20%	2.38%	0.05%	shared jan 31	Tpi1
									1.45E-04	5.73E-03	8	19	1962	21447	42.11%	9.15%	0.41%	0.09%	gluconeogenesis Jan 31	G6pc, G6pc2, G6pc3, Pck1, Pck2, Pcx, Slc25a13, Slc37a4
									1.96E-01	2.72E-01	2	9	1962	21447	22.22%	9.15%	0.10%	0.04%	glycolysis Jan 31	Gck, Hkdc1
				MP:0002118	abnormal lipid homeostasis	1.45E-04	1.00E+00	1.45E-04	1.00E+00	1.00E+00	0	11	1962	21447	0.00%	9.15%	0.00%	0.05%	shared jan 31	
									1.90E-04	7.05E-03	4	19	336	21447	21.05%	1.57%	1.19%	0.09%	gluconeogenesis Jan 31	G6pc, Pck1, Pcx, Slc37a4
									1.33E-01	2.09E-01	1	9	336	21447	11.11%	1.57%	0.30%	0.04%	glycolysis Jan 31	Gck
				MP:0001552	increased circulating triglyceride level	1.90E-04	1.00E+00	1.90E-04	1.00E+00	1.00E+00	0	11	336	21447	0.00%	1.57%	0.00%	0.05%	shared jan 31	
									2.19E-04	7.64E-03	7	19	1532	21447	36.84%	7.14%	0.46%	0.09%	gluconeogenesis Jan 31	G6pc, G6pc2, G6pc3, Pck1, Pck2, Pcx, Slc37a4
									4.87E-01	5.26E-01	1	9	1532	21447	11.11%	7.14%	0.07%	0.04%	glycolysis Jan 31	Gck
				MP:0003949	abnormal circulating lipid level	2.19E-04	1.00E+00	2.19E-04	1.00E+00	1.00E+00	0	11	1532	21447	0.00%	7.14%	0.00%	0.05%	shared jan 31	
									2.39E-04	7.85E-03	2	19	26	21447	10.53%	0.12%	7.69%	0.09%	gluconeogenesis Jan 31	Pck1, Pcx
									1.00E+00	1.00E+00	0	9	26	21447	0.00%	0.12%	0.00%	0.04%	glycolysis Jan 31	
				MP:0013405	increased circulating lactate level	2.39E-04	1.00E+00	2.39E-04	1.00E+00	1.00E+00	0	11	26	21447	0.00%	0.12%	0.00%	0.05%	shared jan 31	
									1.00E+00	1.00E+00	0	19	338	21447	0.00%	1.58%	0.00%	0.09%	gluconeogenesis Jan 31	
									3.04E-04	1.11E-02	3	9	338	21447	33.33%	1.58%	0.89%	0.04%	glycolysis Jan 31	Hk1, Pfkm, Pklr
				MP:0000208	decreased hematocrit	3.04E-04	1.00E+00	3.04E-04	1.24E-02	9.30E-02	2	11	338	21447	18.18%	1.58%	0.59%	0.05%	shared jan 31	Gpi1, Tpi1
									2.61E-01	3.96E-01	1	19	339	21447	5.26%	1.58%	0.29%	0.09%	gluconeogenesis Jan 31	Mdh1
									3.06E-04	1.11E-02	3	9	339	21447	33.33%	1.58%	0.88%	0.04%	glycolysis Jan 31	Gck, Pfkl, Pklr
				MP:0005584	abnormal enzyme/coenzyme activity	3.06E-04	2.61E-01	1.17E-03	5.88E-04	2.65E-02	3	11	339	21447	27.27%	1.58%	0.88%	0.05%	shared jan 31	Gapdh, Gpi1, Tpi1
									1.00E+00	1.00E+00	0	19	65	21447	0.00%	0.30%	0.00%	0.09%	gluconeogenesis Jan 31	
									3.21E-04	1.11E-02	2	9	65	21447	22.22%	0.30%	3.08%	0.04%	glycolysis Jan 31	Pfkm, Pklr
				MP:0003656	abnormal erythrocyte physiology	3.21E-04	1.00E+00	3.21E-04	4.89E-04	2.65E-02	2	11	65	21447	18.18%	0.30%	3.08%	0.05%	shared jan 31	Gpi1, Tpi1
									1.00E+00	1.00E+00	0	19	1	21447	0.00%	0.00%	0.00%	0.09%	gluconeogenesis Jan 31	
									4.20E-04	1.34E-02	1	9	1	21447	11.11%	0.00%	100.00%	0.04%	glycolysis Jan 31	Hk2
				MP:0000874	irregular external granule cell layer thickness	4.20E-04	1.00E+00	4.20E-04	1.00E+00	1.00E+00	0	11	1	21447	0.00%	0.00%	0.00%	0.05%	shared jan 31	
									4.62E-04	1.40E-02	3	19	174	21447	15.79%	0.81%	1.72%	0.09%	gluconeogenesis Jan 31	G6pc, Pck1, Slc37a4
									2.27E-03	4.10E-02	2	9	174	21447	22.22%	0.81%	1.15%	0.04%	glycolysis Jan 31	Gck, Pfkm
				MP:0005437	abnormal glycogen level	4.62E-04	1.00E+00	4.62E-04	1.00E+00	1.00E+00	0	11	174	21447	0.00%	0.81%	0.00%	0.05%	shared jan 31	
									1.00E+00	1.00E+00	0	19	392	21447	0.00%	1.83%	0.00%	0.09%	gluconeogenesis Jan 31	
									4.69E-04	1.39E-02	3	9	392	21447	33.33%	1.83%	0.77%	0.04%	glycolysis Jan 31	Hk1, Pfkm, Pklr
				MP:0013663	increased myeloid cell number	4.69E-04	1.00E+00	4.69E-04	1.64E-02	1.02E-01	2	11	392	21447	18.18%	1.83%	0.51%	0.05%	shared jan 31	Gpi1, Tpi1
									4.73E-04	1.40E-02	12	19	5352	21447	63.16%	24.95%	0.22%	0.09%	gluconeogenesis Jan 31	Fbp1, G6pc, G6pc2, G6pc3, Got1, Pck1, Pck2, Pcx, Slc25a10, Slc25a13, Slc37a1, Slc37a4
									1.65E-01	2.42E-01	4	9	5352	21447	44.44%	24.95%	0.07%	0.04%	glycolysis Jan 31	Gck, Hk1, Pfkm, Pklr
				MP:0009642	abnormal blood homeostasis	4.73E-04	8.02E-01	5.90E-04	8.02E-01	8.28E-01	2	11	5352	21447	18.18%	24.95%	0.04%	0.05%	shared jan 31	Gpi1, Tpi1
									5.13E-04	1.45E-02	2	19	38	21447	10.53%	0.18%	5.26%	0.09%	gluconeogenesis Jan 31	Pck1, Pcx
									1.58E-02	7.91E-02	1	9	38	21447	11.11%	0.18%	2.63%	0.04%	glycolysis Jan 31	Pfkm
				MP:0013403	abnormal circulating lactate level	5.13E-04	1.00E+00	5.13E-04	1.00E+00	1.00E+00	0	11	38	21447	0.00%	0.18%	0.00%	0.05%	shared jan 31	
									1.00E+00	1.00E+00	0	19	1	21447	0.00%	0.00%	0.00%	0.09%	gluconeogenesis Jan 31	
									1.00E+00	1.00E+00	0	9	1	21447	0.00%	0.00%	0.00%	0.04%	glycolysis Jan 31	
				MP:0011607	abnormal glyceraldehyde-3-phosphate dehydrogenase (NAD+) (phosphorylating) activity	5.13E-04	1.00E+00	5.13E-04	5.13E-04	2.65E-02	1	11	1	21447	9.09%	0.00%	100.00%	0.05%	shared jan 31	Gapdh
									1.00E+00	1.00E+00	0	19	1	21447	0.00%	0.00%	0.00%	0.09%	gluconeogenesis Jan 31	
									1.00E+00	1.00E+00	0	9	1	21447	0.00%	0.00%	0.00%	0.04%	glycolysis Jan 31	
				MP:0011609	decreased glyceraldehyde-3-phosphate dehydrogenase (NAD+) (phosphorylating) activity	5.13E-04	1.00E+00	5.13E-04	5.13E-04	2.65E-02	1	11	1	21447	9.09%	0.00%	100.00%	0.05%	shared jan 31	Gapdh
									1.00E+00	1.00E+00	0	19	331	21447	0.00%	1.54%	0.00%	0.09%	gluconeogenesis Jan 31	
									1.00E+00	1.00E+00	0	9	331	21447	0.00%	1.54%	0.00%	0.04%	glycolysis Jan 31	
				MP:0002675	asthenozoospermia	5.48E-04	1.00E+00	5.48E-04	5.48E-04	2.65E-02	3	11	331	21447	27.27%	1.54%	0.91%	0.05%	shared jan 31	Eno4, Gapdhs, Pgk2
									1.00E+00	1.00E+00	0	19	331	21447	0.00%	1.54%	0.00%	0.09%	gluconeogenesis Jan 31	
									1.00E+00	1.00E+00	0	9	331	21447	0.00%	1.54%	0.00%	0.04%	glycolysis Jan 31	
				MP:0006205	embryonic lethality between implantation and somite formation	5.48E-04	1.00E+00	5.48E-04	5.48E-04	2.65E-02	3	11	331	21447	27.27%	1.54%	0.91%	0.05%	shared jan 31	Eno1, Gapdh, Tpi1
									6.16E-04	1.61E-02	3	19	192	21447	15.79%	0.90%	1.56%	0.09%	gluconeogenesis Jan 31	G6pc, Pck1, Slc37a4
									2.75E-03	4.52E-02	2	9	192	21447	22.22%	0.90%	1.04%	0.04%	glycolysis Jan 31	Gck, Pfkm
				MP:0005438	abnormal glycogen homeostasis	6.16E-04	1.00E+00	6.16E-04	1.00E+00	1.00E+00	0	11	192	21447	0.00%	0.90%	0.00%	0.05%	shared jan 31	
									6.27E-04	1.61E-02	2	19	42	21447	10.53%	0.20%	4.76%	0.09%	gluconeogenesis Jan 31	G6pc, Slc37a4
									1.00E+00	1.00E+00	0	9	42	21447	0.00%	0.20%	0.00%	0.04%	glycolysis Jan 31	
				MP:0010400	increased liver glycogen level	6.27E-04	1.00E+00	6.27E-04	1.00E+00	1.00E+00	0	11	42	21447	0.00%	0.20%	0.00%	0.05%	shared jan 31	
									6.57E-04	1.62E-02	2	19	43	21447	10.53%	0.20%	4.65%	0.09%	gluconeogenesis Jan 31	Pck1, Pcx
									1.00E+00	1.00E+00	0	9	43	21447	0.00%	0.20%	0.00%	0.04%	glycolysis Jan 31	
				MP:0002575	increased circulating ketone body level	6.57E-04	1.00E+00	6.57E-04	1.00E+00	1.00E+00	0	11	43	21447	0.00%	0.20%	0.00%	0.05%	shared jan 31	
									1.00E+00	1.00E+00	0	19	440	21447	0.00%	2.05%	0.00%	0.09%	gluconeogenesis Jan 31	
									6.57E-04	1.74E-02	3	9	440	21447	33.33%	2.05%	0.68%	0.04%	glycolysis Jan 31	Gck, Hk1, Pklr
				MP:0005563	abnormal hemoglobin content	6.57E-04	1.00E+00	6.57E-04	2.04E-02	1.11E-01	2	11	440	21447	18.18%	2.05%	0.45%	0.05%	shared jan 31	Gapdh, Tpi1
									2.49E-01	3.83E-01	2	19	1085	21447	10.53%	5.06%	0.18%	0.09%	gluconeogenesis Jan 31	Mdh1, Pcx
									6.69E-04	1.74E-02	4	9	1085	21447	44.44%	5.06%	0.37%	0.04%	glycolysis Jan 31	Gck, Pfkl, Pfkm, Pklr
				MP:0005266	abnormal metabolism	6.69E-04	2.49E-01	2.68E-03	1.61E-03	3.47E-02	4	11	1085	21447	36.36%	5.06%	0.37%	0.05%	shared jan 31	Gapdh, Gpi1, Pgam1, Tpi1

									1.00E+00	1.00E+00	0	19	457	21447	0.00%	2.13%	0.00%	0.09%	gluconeogenesis Jan 31	
									7.34E-04	1.80E-02	3	9	457	21447	33.33%	2.13%	0.66%	0.04%	glycolysis Jan 31	Hk1, Pfkm, Pklr
				MP:0002596	abnormal hematocrit	7.34E-04	1.00E+00	7.34E-04	1.40E-03	3.47E-02	3	11	457	21447	27.27%	2.13%	0.66%	0.05%	shared jan 31	Gapdh, Gpi1, Tpi1
									7.85E-04	1.86E-02	2	19	47	21447	10.53%	0.22%	4.26%	0.09%	gluconeogenesis Jan 31	Pck1, Pcx
									1.96E-02	8.22E-02	1	9	47	21447	11.11%	0.22%	2.13%	0.04%	glycolysis Jan 31	Gck
				MP:0030970	ketosis	7.85E-04	1.00E+00	7.85E-04	1.00E+00	1.00E+00	0	11	47	21447	0.00%	0.22%	0.00%	0.05%	shared jan 31	
									1.00E+00	1.00E+00	0	19	382	21447	0.00%	1.78%	0.00%	0.09%	gluconeogenesis Jan 31	
									1.00E+00	1.00E+00	0	9	382	21447	0.00%	1.78%	0.00%	0.04%	glycolysis Jan 31	
				MP:0002674	abnormal sperm motility	8.32E-04	1.00E+00	8.32E-04	8.32E-04	3.28E-02	3	11	382	21447	27.27%	1.78%	0.79%	0.05%	shared jan 31	Eno4, Gapdhs, Pgk2
									1.00E+00	1.00E+00	0	19	2	21447	0.00%	0.01%	0.00%	0.09%	gluconeogenesis Jan 31	
									8.39E-04	1.84E-02	1	9	2	21447	11.11%	0.01%	50.00%	0.04%	glycolysis Jan 31	Gck
				MP:0011604	abnormal glucokinase activity	8.39E-04	1.00E+00	8.39E-04	1.00E+00	1.00E+00	0	11	2	21447	0.00%	0.01%	0.00%	0.05%	shared jan 31	
									1.00E+00	1.00E+00	0	19	2	21447	0.00%	0.01%	0.00%	0.09%	gluconeogenesis Jan 31	
									8.39E-04	1.84E-02	1	9	2	21447	11.11%	0.01%	50.00%	0.04%	glycolysis Jan 31	Gck
				MP:0011606	decreased glucokinase activity	8.39E-04	1.00E+00	8.39E-04	1.00E+00	1.00E+00	0	11	2	21447	0.00%	0.01%	0.00%	0.05%	shared jan 31	
									1.07E-03	2.44E-02	2	19	55	21447	10.53%	0.26%	3.64%	0.09%	gluconeogenesis Jan 31	Got2, Mdh2
									1.00E+00	1.00E+00	0	9	55	21447	0.00%	0.26%	0.00%	0.04%	glycolysis Jan 31	
				MP:0003864	abnormal midbrain development	1.07E-03	1.00E+00	1.07E-03	1.00E+00	1.00E+00	0	11	55	21447	0.00%	0.26%	0.00%	0.05%	shared jan 31	
									1.00E+00	1.00E+00	0	19	424	21447	0.00%	1.98%	0.00%	0.09%	gluconeogenesis Jan 31	
									1.00E+00	1.00E+00	0	9	424	21447	0.00%	1.98%	0.00%	0.04%	glycolysis Jan 31	
				MP:0004543	abnormal sperm physiology	1.12E-03	1.00E+00	1.12E-03	1.12E-03	3.47E-02	3	11	424	21447	27.27%	1.98%	0.71%	0.05%	shared jan 31	Eno4, Gapdhs, Pgk2
									1.00E+00	1.00E+00	0	19	562	21447	0.00%	2.62%	0.00%	0.09%	gluconeogenesis Jan 31	
									1.34E-03	2.78E-02	3	9	562	21447	33.33%	2.62%	0.53%	0.04%	glycolysis Jan 31	Gck, Hkdc1, Pfkm
				MP:0005293	impaired glucose tolerance	1.34E-03	1.00E+00	1.34E-03	1.00E+00	1.00E+00	0	11	562	21447	0.00%	2.62%	0.00%	0.05%	shared jan 31	
									1.45E-03	3.08E-02	2	19	64	21447	10.53%	0.30%	3.12%	0.09%	gluconeogenesis Jan 31	Pcx, Slc25a13
									1.00E+00	1.00E+00	0	9	64	21447	0.00%	0.30%	0.00%	0.04%	glycolysis Jan 31	
				MP:0003383	abnormal gluconeogenesis	1.45E-03	1.00E+00	1.45E-03	1.00E+00	1.00E+00	0	11	64	21447	0.00%	0.30%	0.00%	0.05%	shared jan 31	
									1.50E-03	3.08E-02	2	19	65	21447	10.53%	0.30%	3.08%	0.09%	gluconeogenesis Jan 31	Pck1, Pcx
									1.00E+00	1.00E+00	0	9	65	21447	0.00%	0.30%	0.00%	0.04%	glycolysis Jan 31	
				MP:0003457	abnormal circulating ketone body level	1.50E-03	1.00E+00	1.50E-03	1.00E+00	1.00E+00	0	11	65	21447	0.00%	0.30%	0.00%	0.05%	shared jan 31	
									1.51E-03	3.08E-02	3	19	262	21447	15.79%	1.22%	1.15%	0.09%	gluconeogenesis Jan 31	G6pc3, Got1, Slc37a4
									1.00E+00	1.00E+00	0	9	262	21447	0.00%	1.22%	0.00%	0.04%	glycolysis Jan 31	
				MP:0000334	decreased granulocyte number	1.51E-03	1.00E+00	1.51E-03	1.00E+00	1.00E+00	0	11	262	21447	0.00%	1.22%	0.00%	0.05%	shared jan 31	
									1.00E+00	1.00E+00	0	19	3	21447	0.00%	0.01%	0.00%	0.09%	gluconeogenesis Jan 31	
									1.00E+00	1.00E+00	0	9	3	21447	0.00%	0.01%	0.00%	0.04%	glycolysis Jan 31	
				MP:0009835	absent sperm annulus	1.54E-03	1.00E+00	1.54E-03	1.54E-03	3.47E-02	1	11	3	21447	9.09%	0.01%	33.33%	0.05%	shared jan 31	Eno4
									1.70E-03	3.36E-02	13	19	7085	21447	68.42%	33.03%	0.18%	0.09%	gluconeogenesis Jan 31	Fbp1, G6pc, G6pc2, G6pc3, Got1, Pck1, Pck2, Pcx, Slc25a10, Slc25a12, Slc25a13, Slc37a1, Slc37a4
									1.40E-01	2.16E-01	5	9	7085	21447	55.56%	33.03%	0.07%	0.04%	glycolysis Jan 31	Gck, Hk1, Hkdc1, Pfkm, Pklr
				MP:0001764	abnormal homeostasis	1.70E-03	7.59E-01	2.24E-03	7.59E-01	7.87E-01	3	11	7085	21447	27.27%	33.03%	0.04%	0.05%	shared jan 31	Gpi1, Pgam1, Tpi1
									1.88E-03	3.58E-02	3	19	283	21447	15.79%	1.32%	1.06%	0.09%	gluconeogenesis Jan 31	G6pc2, Pck1, Pck2
									1.00E+00	1.00E+00	0	9	283	21447	0.00%	1.32%	0.00%	0.04%	glycolysis Jan 31	
				MP:0002644	decreased circulating triglyceride level	1.88E-03	1.00E+00	1.88E-03	1.00E+00	1.00E+00	0	11	283	21447	0.00%	1.32%	0.00%	0.05%	shared jan 31	
									1.00E+00	1.00E+00	0	19	159	21447	0.00%	0.74%	0.00%	0.09%	gluconeogenesis Jan 31	
									1.90E-03	3.76E-02	2	9	159	21447	22.22%	0.74%	1.26%	0.04%	glycolysis Jan 31	Gck, Pfkm
				MP:0003059	decreased insulin secretion	1.90E-03	1.00E+00	1.90E-03	1.00E+00	1.00E+00	0	11	159	21447	0.00%	0.74%	0.00%	0.05%	shared jan 31	
									1.00E+00	1.00E+00	0	19	129	21447	0.00%	0.60%	0.00%	0.09%	gluconeogenesis Jan 31	
									1.00E+00	1.00E+00	0	9	129	21447	0.00%	0.60%	0.00%	0.04%	glycolysis Jan 31	
				MP:0005642	decreased mean corpuscular hemoglobin concentration	1.91E-03	1.00E+00	1.91E-03	1.91E-03	3.66E-02	2	11	129	21447	18.18%	0.60%	1.55%	0.05%	shared jan 31	Gpi1, Tpi1
									1.95E-03	3.58E-02	14	19	8239	21447	73.68%	38.42%	0.17%	0.09%	gluconeogenesis Jan 31	Fbp1, G6pc, G6pc2, G6pc3, Got1, Mdh1, Pck1, Pck2, Pcx, Slc25a10, Slc25a12, Slc25a13, Slc37a1, Slc37a4
									8.27E-02	1.60E-01	6	9	8239	21447	66.67%	38.42%	0.07%	0.04%	glycolysis Jan 31	Gck, Hk1, Hkdc1, Pfkl, Pfkm, Pklr
				MP:0005376	homeostasis/metabolism phenotype	1.95E-03	6.65E-01	2.93E-03	6.65E-01	7.01E-01	4	11	8239	21447	36.36%	38.42%	0.05%	0.05%	shared jan 31	Gapdh, Gpi1, Pgam1, Tpi1
									1.00E+00	1.00E+00	0	19	1146	21447	0.00%	5.34%	0.00%	0.09%	gluconeogenesis Jan 31	
									8.00E-02	1.56E-01	2	9	1146	21447	22.22%	5.34%	0.17%	0.04%	glycolysis Jan 31	Hk2, Pkm
				MP:0013292	embryonic lethality prior to organogenesis	1.98E-03	1.00E+00	1.98E-03	1.98E-03	3.66E-02	4	11	1146	21447	36.36%	5.34%	0.35%	0.05%	shared jan 31	Eno1, Gapdh, Gpi1, Tpi1
									1.00E+00	1.00E+00	0	19	163	21447	0.00%	0.76%	0.00%	0.09%	gluconeogenesis Jan 31	
									2.00E-03	3.77E-02	2	9	163	21447	22.22%	0.76%	1.23%	0.04%	glycolysis Jan 31	Hk1, Pklr
				MP:0000245	abnormal erythropoiesis	2.00E-03	1.00E+00	2.00E-03	8.05E-02	2.28E-01	1	11	163	21447	9.09%	0.76%	0.61%	0.05%	shared jan 31	Tpi1
									2.00E-03	3.58E-02	6	19	1602	21447	31.58%	7.47%	0.37%	0.09%	gluconeogenesis Jan 31	G6pc, Pck1, Pcx, Slc25a13, Slc37a1, Slc37a4
									2.88E-03	4.52E-02	4	9	1602	21447	44.44%	7.47%	0.25%	0.04%	glycolysis Jan 31	Gck, Hk1, Hkdc1, Pfkm
				MP:0000598	abnormal liver morphology	2.00E-03	1.96E-01	1.02E-02	1.96E-01	3.77E-01	2	11	1602	21447	18.18%	7.47%	0.12%	0.05%	shared jan 31	Aldob, Gpi1
									1.00E+00	1.00E+00	0	19	6	21447	0.00%	0.03%	0.00%	0.09%	gluconeogenesis Jan 31	
									2.52E-03	4.36E-02	1	9	6	21447	11.11%	0.03%	16.67%	0.04%	glycolysis Jan 31	Hk1
				MP:0002814	hyperchromasia	2.52E-03	1.00E+00	2.52E-03	1.00E+00	1.00E+00	0	11	6	21447	0.00%	0.03%	0.00%	0.05%	shared jan 31	
									2.66E-03	4.63E-02	5	19	1144	21447	26.32%	5.33%	0.44%	0.09%	gluconeogenesis Jan 31	G6pc, G6pc3, Pck1, Slc25a12, Slc37a4
									3.89E-01	4.40E-01	1	9	1144	21447	11.11%	5.33%	0.09%	0.04%	glycolysis Jan 31	Gck
				MP:0001731	abnormal postnatal growth	2.66E-03	1.00E+00	2.66E-03	1.00E+00	1.00E+00	0	11	1144	21447	0.00%	5.33%	0.00%	0.05%	shared jan 31	
									2.91E-03	4.91E-02	2	19	91	21447	10.53%	0.42%	2.20%	0.09%	gluconeogenesis Jan 31	G6pc, Slc37a4

					3.76E-02	1.08E-01	1	9	91	21447	11.11%	0.42%	1.10%	0.04%	glycolysis Jan 31	Pfkm
MP:0005440	increased glycogen level	2.91E-03	1.00E+00	2.91E-03	1.00E+00	1.00E+00	0	11	91	21447	0.00%	0.42%	0.00%	0.05%	shared jan 31	
					1.00E+00	1.00E+00	0	19	7	21447	0.00%	0.03%	0.00%	0.09%	gluconeogenesis Jan 31	
					2.93E-03	4.52E-02	1	9	7	21447	11.11%	0.03%	14.29%	0.04%	glycolysis Jan 31	Gck
MP:0006353	increased glycosylated hemoglobin level	2.93E-03	1.00E+00	2.93E-03	1.00E+00	1.00E+00	0	11	7	21447	0.00%	0.03%	0.00%	0.05%	shared jan 31	
					1.00E+00	1.00E+00	0	19	1629	21447	0.00%	7.60%	0.00%	0.09%	gluconeogenesis Jan 31	
					3.06E-03	4.55E-02	4	9	1629	21447	44.44%	7.60%	0.25%	0.04%	glycolysis Jan 31	Gck, Hk1, Pfkm, Pklr
MP:0002447	abnormal erythrocyte morphology	3.06E-03	1.00E+00	3.06E-03	4.55E-02	1.63E-01	3	11	1629	21447	27.27%	7.60%	0.18%	0.05%	shared jan 31	Gapdh, Gpi1, Tpi1
					1.00E+00	1.00E+00	0	19	6	21447	0.00%	0.03%	0.00%	0.09%	gluconeogenesis Jan 31	
					1.00E+00	1.00E+00	0	9	6	21447	0.00%	0.03%	0.00%	0.04%	glycolysis Jan 31	
MP:0008993	abnormal portal triad morphology	3.07E-03	1.00E+00	3.07E-03	3.07E-03	5.33E-02	1	11	6	21447	9.09%	0.03%	16.67%	0.05%	shared jan 31	Aldob
					3.20E-03	5.26E-02	3	19	341	21447	15.79%	1.59%	0.88%	0.09%	gluconeogenesis Jan 31	G6pc, Pcx, Slc37a4
					1.00E+00	1.00E+00	0	9	341	21447	0.00%	1.59%	0.00%	0.04%	glycolysis Jan 31	
MP:0003068	enlarged kidney	3.20E-03	1.00E+00	3.20E-03	1.00E+00	1.00E+00	0	11	341	21447	0.00%	1.59%	0.00%	0.05%	shared jan 31	
					1.06E-01	2.66E-01	3	19	1308	21447	15.79%	6.10%	0.23%	0.09%	gluconeogenesis Jan 31	Got1, Mdh1, Mdh2
					1.01E-01	1.82E-01	2	9	1308	21447	22.22%	6.10%	0.15%	0.04%	glycolysis Jan 31	Hk2, Pkm
MP:0013293	embryonic lethality prior to tooth bud stage	3.21E-03	1.06E-01	3.04E-02	3.21E-03	5.33E-02	4	11	1308	21447	36.36%	6.10%	0.31%	0.05%	shared jan 31	Eno1, Gapdh, Gpi1, Tpi1
					3.33E-03	5.33E-02	6	19	1771	21447	31.58%	8.26%	0.34%	0.09%	gluconeogenesis Jan 31	G6pc, Pck1, Pcx, Slc25a13, Slc37a1, Slc37a4
					4.16E-03	4.65E-02	4	9	1771	21447	44.44%	8.26%	0.23%	0.04%	glycolysis Jan 31	Gck, Hk1, Hkdc1, Pfkm
MP:0002138	abnormal hepatobiliary system morphology	3.33E-03	2.29E-01	1.45E-02	2.29E-01	4.08E-01	2	11	1771	21447	18.18%	8.26%	0.11%	0.05%	shared jan 31	Aldob, Gpi1
					3.54E-03	5.51E-02	1	19	4	21447	5.26%	0.02%	25.00%	0.09%	gluconeogenesis Jan 31	Pck1
					1.00E+00	1.00E+00	0	9	4	21447	0.00%	0.02%	0.00%	0.04%	glycolysis Jan 31	
MP:0009128	decreased brown fat cell number	3.54E-03	1.00E+00	3.54E-03	1.00E+00	1.00E+00	0	11	4	21447	0.00%	0.02%	0.00%	0.05%	shared jan 31	
					1.00E+00	1.00E+00	0	19	1699	21447	0.00%	7.92%	0.00%	0.09%	gluconeogenesis Jan 31	
					3.57E-03	4.65E-02	4	9	1699	21447	44.44%	7.92%	0.24%	0.04%	glycolysis Jan 31	Gck, Hk1, Pfkm, Pklr
MP:0013659	abnormal erythroid lineage cell morphology	3.57E-03	1.00E+00	3.57E-03	5.06E-02	1.71E-01	3	11	1699	21447	27.27%	7.92%	0.18%	0.05%	shared jan 31	Gapdh, Gpi1, Tpi1
					3.29E-01	4.47E-01	3	19	2280	21447	15.79%	10.63%	0.13%	0.09%	gluconeogenesis Jan 31	Got1, Mdh1, Mdh2
					6.17E-02	1.34E-01	3	9	2280	21447	33.33%	10.63%	0.13%	0.04%	glycolysis Jan 31	Gck, Hk2, Pkm
MP:0008762	embryonic lethality	3.60E-03	3.29E-01	1.10E-02	3.60E-03	5.67E-02	5	11	2280	21447	45.45%	10.63%	0.22%	0.05%	shared jan 31	Eno1, Gapdh, Gpi1, Pgam1, Tpi1
					1.79E-01	3.29E-01	1	19	222	21447	5.26%	1.04%	0.45%	0.09%	gluconeogenesis Jan 31	Pcx
					3.66E-03	4.65E-02	2	9	222	21447	22.22%	1.04%	0.90%	0.04%	glycolysis Jan 31	Gck, Pfkm
MP:0003564	abnormal insulin secretion	3.66E-03	1.00E+00	3.66E-03	1.00E+00	1.00E+00	0	11	222	21447	0.00%	1.04%	0.00%	0.05%	shared jan 31	
					1.82E-01	3.31E-01	1	19	225	21447	5.26%	1.05%	0.44%	0.09%	gluconeogenesis Jan 31	Pcx
					3.76E-03	4.65E-02	2	9	225	21447	22.22%	1.05%	0.89%	0.04%	glycolysis Jan 31	Gck, Pfkm
MP:0014195	abnormal endocrine pancreas secretion	3.76E-03	1.00E+00	3.76E-03	1.00E+00	1.00E+00	0	11	225	21447	0.00%	1.05%	0.00%	0.05%	shared jan 31	
					1.00E+00	1.00E+00	0	19	9	21447	0.00%	0.04%	0.00%	0.09%	gluconeogenesis Jan 31	
					3.77E-03	4.65E-02	1	9	9	21447	11.11%	0.04%	11.11%	0.04%	glycolysis Jan 31	Gck
MP:0006351	abnormal glycosylated hemoglobin level	3.77E-03	1.00E+00	3.77E-03	1.00E+00	1.00E+00	0	11	9	21447	0.00%	0.04%	0.00%	0.05%	shared jan 31	
					1.88E-01	3.36E-01	1	19	234	21447	5.26%	1.09%	0.43%	0.09%	gluconeogenesis Jan 31	Pcx
					4.06E-03	4.65E-02	2	9	234	21447	22.22%	1.09%	0.85%	0.04%	glycolysis Jan 31	Gck, Pfkm
MP:0002694	abnormal pancreas secretion	4.06E-03	1.00E+00	4.06E-03	1.00E+00	1.00E+00	0	11	234	21447	0.00%	1.09%	0.00%	0.05%	shared jan 31	
					1.00E+00	1.00E+00	0	19	8	21447	0.00%	0.04%	0.00%	0.09%	gluconeogenesis Jan 31	
					1.00E+00	1.00E+00	0	9	8	21447	0.00%	0.04%	0.00%	0.04%	glycolysis Jan 31	
MP:0008992	abnormal portal lobule morphology	4.10E-03	1.00E+00	4.10E-03	4.10E-03	5.87E-02	1	11	8	21447	9.09%	0.04%	12.50%	0.05%	shared jan 31	Aldob
					4.11E-03	6.23E-02	3	19	373	21447	15.79%	1.74%	0.80%	0.09%	gluconeogenesis Jan 31	G6pc2, Pck1, Pck2
					1.46E-01	2.20E-01	1	9	373	21447	11.11%	1.74%	0.27%	0.04%	glycolysis Jan 31	Hkdc1
MP:0005318	decreased triglyceride level	4.11E-03	1.00E+00	4.11E-03	1.00E+00	1.00E+00	0	11	373	21447	0.00%	1.74%	0.00%	0.05%	shared jan 31	
					1.00E+00	1.00E+00	0	19	236	21447	0.00%	1.10%	0.00%	0.09%	gluconeogenesis Jan 31	
					4.13E-03	4.65E-02	2	9	236	21447	22.22%	1.10%	0.85%	0.04%	glycolysis Jan 31	Hk1, Pklr
MP:0001770	abnormal iron level	4.13E-03	1.00E+00	4.13E-03	1.00E+00	1.00E+00	0	11	236	21447	0.00%	1.10%	0.00%	0.05%	shared jan 31	
					4.24E-03	6.23E-02	3	19	377	21447	15.79%	1.76%	0.80%	0.09%	gluconeogenesis Jan 31	G6pc, Pck1, Slc37a4
					1.00E+00	1.00E+00	0	9	377	21447	0.00%	1.76%	0.00%	0.04%	glycolysis Jan 31	
MP:0000599	enlarged liver	4.24E-03	1.00E+00	4.24E-03	1.77E-01	3.72E-01	1	11	377	21447	9.09%	1.76%	0.27%	0.05%	shared jan 31	Gpi1
					1.00E+00	1.00E+00	0	19	242	21447	0.00%	1.13%	0.00%	0.09%	gluconeogenesis Jan 31	
					4.33E-03	4.65E-02	2	9	242	21447	22.22%	1.13%	0.83%	0.04%	glycolysis Jan 31	Hk1, Pklr
MP:0005637	abnormal iron homeostasis	4.33E-03	1.00E+00	4.33E-03	1.00E+00	1.00E+00	0	11	242	21447	0.00%	1.13%	0.00%	0.05%	shared jan 31	
					4.42E-03	6.23E-02	1	19	5	21447	5.26%	0.02%	20.00%	0.09%	gluconeogenesis Jan 31	Pcx
					1.00E+00	1.00E+00	0	9	5	21447	0.00%	0.02%	0.00%	0.04%	glycolysis Jan 31	
MP:0003839	abnormal insulin clearance	4.42E-03	1.00E+00	4.42E-03	1.00E+00	1.00E+00	0	11	5	21447	0.00%	0.02%	0.00%	0.05%	shared jan 31	
					4.42E-03	6.23E-02	1	19	5	21447	5.26%	0.02%	20.00%	0.09%	gluconeogenesis Jan 31	Slc25a12
					1.00E+00	1.00E+00	0	9	5	21447	0.00%	0.02%	0.00%	0.04%	glycolysis Jan 31	
MP:0030585	enhanced behavioral response to amphetamine	4.42E-03	1.00E+00	4.42E-03	1.00E+00	1.00E+00	0	11	5	21447	0.00%	0.02%	0.00%	0.05%	shared jan 31	
					1.00E+00	1.00E+00	0	19	11	21447	0.00%	0.05%	0.00%	0.09%	gluconeogenesis Jan 31	
					4.61E-03	4.65E-02	1	9	11	21447	11.11%	0.05%	9.09%	0.04%	glycolysis Jan 31	Pfkm
MP:0031318	enhanced skeletal muscle regeneration	4.61E-03	1.00E+00	4.61E-03	1.00E+00	1.00E+00	0	11	11	21447	0.00%	0.05%	0.00%	0.05%	shared jan 31	
					2.03E-01	3.46E-01	1	19	254	21447	5.26%	1.18%	0.39%	0.09%	gluconeogenesis Jan 31	Pcx
					4.76E-03	4.65E-02	2	9	254	21447	22.22%	1.18%	0.79%	0.04%	glycolysis Jan 31	Gck, Pfkm
MP:0003562	abnormal pancreatic beta cell physiology	4.76E-03	1.00E+00	4.76E-03	1.00E+00	1.00E+00	0	11	254	21447	0.00%	1.18%	0.00%	0.05%	shared jan 31	
					1.00E+00	1.00E+00	0	19	886	21447	0.00%	4.13%	0.00%	0.09%	gluconeogenesis Jan 31	
					4.90E-03	4.65E-02	3	9	886	21447	33.33%	4.13%	0.34%	0.04%	glycolysis Jan 31	Hk1, Pfkm, Pklr

				MP:0013661	abnormal myeloid cell number	4.90E-03	1.00E+00	4.90E-03	7.32E-02	2.16E-01	2	11	886	21447	18.18%	4.13%	0.23%	0.05%	shared jan 31	Gpi1, Tpi1
									2.06E-01	3.47E-01	1	19	258	21447	5.26%	1.20%	0.39%	0.09%	gluconeogenesis Jan 31	Pcx
									4.91E-03	4.65E-02	2	9	258	21447	22.22%	1.20%	0.78%	0.04%	glycolysis Jan 31	Gck, Pfkm
				MP:0010147	abnormal endocrine pancreas physiology	4.91E-03	1.00E+00	4.91E-03	1.00E+00	1.00E+00	0	11	258	21447	0.00%	1.20%	0.00%	0.05%	shared jan 31	
									1.00E+00	1.00E+00	0	19	258	21447	0.00%	1.20%	0.00%	0.09%	gluconeogenesis Jan 31	
									4.91E-03	4.65E-02	2	9	258	21447	22.22%	1.20%	0.78%	0.04%	glycolysis Jan 31	Hk1, Pklr
				MP:0002590	increased mean corpuscular volume	4.91E-03	1.00E+00	4.91E-03	7.38E-03	8.02E-02	2	11	258	21447	18.18%	1.20%	0.78%	0.05%	shared jan 31	Gpi1, Tpi1
									5.52E-01	6.08E-01	1	19	887	21447	5.26%	4.14%	0.11%	0.09%	gluconeogenesis Jan 31	Pcx
									4.91E-03	4.65E-02	3	9	887	21447	33.33%	4.14%	0.34%	0.04%	glycolysis Jan 31	Gck, Hkdc1, Pfkm
				MP:0005291	abnormal glucose tolerance	4.91E-03	1.00E+00	4.91E-03	1.00E+00	1.00E+00	0	11	887	21447	0.00%	4.14%	0.00%	0.05%	shared jan 31	
									1.00E+00	1.00E+00	0	19	12	21447	0.00%	0.06%	0.00%	0.09%	gluconeogenesis Jan 31	
									5.03E-03	4.65E-02	1	9	12	21447	11.11%	0.06%	8.33%	0.04%	glycolysis Jan 31	Pklr
				MP:0010034	abnormal erythrocyte clearance	5.03E-03	1.00E+00	5.03E-03	1.00E+00	1.00E+00	0	11	12	21447	0.00%	0.06%	0.00%	0.05%	shared jan 31	
									1.00E+00	1.00E+00	0	19	12	21447	0.00%	0.06%	0.00%	0.09%	gluconeogenesis Jan 31	
									5.03E-03	4.65E-02	1	9	12	21447	11.11%	0.06%	8.33%	0.04%	glycolysis Jan 31	Pfkm
				MP:0013404	decreased circulating lactate level	5.03E-03	1.00E+00	5.03E-03	1.00E+00	1.00E+00	0	11	12	21447	0.00%	0.06%	0.00%	0.05%	shared jan 31	
									1.00E+00	1.00E+00	0	19	12	21447	0.00%	0.06%	0.00%	0.09%	gluconeogenesis Jan 31	
									5.03E-03	4.65E-02	1	9	12	21447	11.11%	0.06%	8.33%	0.04%	glycolysis Jan 31	Pklr
				MP:0010035	increased erythrocyte clearance	5.03E-03	1.00E+00	5.03E-03	1.00E+00	1.00E+00	0	11	12	21447	0.00%	0.06%	0.00%	0.05%	shared jan 31	
									1.00E+00	1.00E+00	0	19	10	21447	0.00%	0.05%	0.00%	0.09%	gluconeogenesis Jan 31	
									1.00E+00	1.00E+00	0	9	10	21447	0.00%	0.05%	0.00%	0.04%	glycolysis Jan 31	
				MP:0009837	abnormal sperm end piece morphology	5.12E-03	1.00E+00	5.12E-03	5.12E-03	6.72E-02	1	11	10	21447	9.09%	0.05%	10.00%	0.05%	shared jan 31	Eno4
									5.23E-03	6.83E-02	2	19	123	21447	10.53%	0.57%	1.63%	0.09%	gluconeogenesis Jan 31	G6pc, Pck1
									1.00E+00	1.00E+00	0	9	123	21447	0.00%	0.57%	0.00%	0.04%	glycolysis Jan 31	
				MP:0000603	pale liver	5.23E-03	1.00E+00	5.23E-03	6.13E-02	1.95E-01	1	11	123	21447	9.09%	0.57%	0.81%	0.05%	shared jan 31	Aldob
									5.30E-03	6.83E-02	1	19	6	21447	5.26%	0.03%	16.67%	0.09%	gluconeogenesis Jan 31	Slc25a13
									1.00E+00	1.00E+00	0	9	6	21447	0.00%	0.03%	0.00%	0.04%	glycolysis Jan 31	
				MP:0006013	absent endolymphatic sac	5.30E-03	1.00E+00	5.30E-03	1.00E+00	1.00E+00	0	11	6	21447	0.00%	0.03%	0.00%	0.05%	shared jan 31	
									5.30E-03	6.83E-02	1	19	6	21447	5.26%	0.03%	16.67%	0.09%	gluconeogenesis Jan 31	Slc37a4
									1.00E+00	1.00E+00	0	9	6	21447	0.00%	0.03%	0.00%	0.04%	glycolysis Jan 31	
				MP:0005046	absent spleen white pulp	5.30E-03	1.00E+00	5.30E-03	1.00E+00	1.00E+00	0	11	6	21447	0.00%	0.03%	0.00%	0.05%	shared jan 31	
									5.30E-03	6.83E-02	1	19	6	21447	5.26%	0.03%	16.67%	0.09%	gluconeogenesis Jan 31	Slc25a12
									1.00E+00	1.00E+00	0	9	6	21447	0.00%	0.03%	0.00%	0.04%	glycolysis Jan 31	
				MP:0030635	increased taurine level	5.30E-03	1.00E+00	5.30E-03	1.00E+00	1.00E+00	0	11	6	21447	0.00%	0.03%	0.00%	0.05%	shared jan 31	
									1.00E+00	1.00E+00	0	19	13	21447	0.00%	0.06%	0.00%	0.09%	gluconeogenesis Jan 31	
									5.44E-03	4.82E-02	1	9	13	21447	11.11%	0.06%	7.69%	0.04%	glycolysis Jan 31	Pklr
				MP:0005639	hemosiderosis	5.44E-03	1.00E+00	5.44E-03	1.00E+00	1.00E+00	0	11	13	21447	0.00%	0.06%	0.00%	0.05%	shared jan 31	
									1.00E+00	1.00E+00	0	19	273	21447	0.00%	1.27%	0.00%	0.09%	gluconeogenesis Jan 31	
									5.48E-03	4.82E-02	2	9	273	21447	22.22%	1.27%	0.73%	0.04%	glycolysis Jan 31	Hk1, Pfkm
				MP:0004952	increased spleen weight	5.48E-03	1.00E+00	5.48E-03	8.23E-03	8.36E-02	2	11	273	21447	18.18%	1.27%	0.73%	0.05%	shared jan 31	Gpi1, Tpi1
									8.44E-02	2.50E-01	4	19	1924	21447	21.05%	8.97%	0.21%	0.09%	gluconeogenesis Jan 31	Got2, Pck2, Slc37a2, Slc37a4
									5.63E-03	4.82E-02	4	9	1924	21447	44.44%	8.97%	0.21%	0.04%	glycolysis Jan 31	Hk1, Hk3, Pfkm, Pklr
				MP:0002221	abnormal lymph organ size	5.63E-03	2.59E-01	2.17E-02	2.59E-01	4.34E-01	2	11	1924	21447	18.18%	8.97%	0.10%	0.05%	shared jan 31	Gpi1, Tpi1
									1.00E+00	1.00E+00	0	19	11	21447	0.00%	0.05%	0.00%	0.09%	gluconeogenesis Jan 31	
									1.00E+00	1.00E+00	0	9	11	21447	0.00%	0.05%	0.00%	0.04%	glycolysis Jan 31	
				MP:0031084	decreased erythrocyte osmotic fragility	5.63E-03	1.00E+00	5.63E-03	5.63E-03	7.09E-02	1	11	11	21447	9.09%	0.05%	9.09%	0.05%	shared jan 31	Gpi1
									1.00E+00	1.00E+00	0	19	934	21447	0.00%	4.35%	0.00%	0.09%	gluconeogenesis Jan 31	
									5.68E-03	4.82E-02	3	9	934	21447	33.33%	4.35%	0.32%	0.04%	glycolysis Jan 31	Gck, Hk1, Pklr
				MP:0001588	abnormal hemoglobin	5.68E-03	1.00E+00	5.68E-03	1.04E-02	9.30E-02	3	11	934	21447	27.27%	4.35%	0.32%	0.05%	shared jan 31	Gapdh, Gpi1, Tpi1
									5.69E-03	7.16E-02	6	19	1972	21447	31.58%	9.19%	0.30%	0.09%	gluconeogenesis Jan 31	Got1, Got2, Mdh1, Mdh2, Pcx, Slc25a12
									4.27E-02	1.11E-01	3	9	1972	21447	33.33%	9.19%	0.15%	0.04%	glycolysis Jan 31	Hkdc1, Pfkl, Pkm
				MP:0011100	preweaning lethality, complete penetrance	5.69E-03	7.31E-02	7.78E-02	7.31E-02	2.16E-01	3	11	1972	21447	27.27%	9.19%	0.15%	0.05%	shared jan 31	Aldoa, Gapdh, Gpi1
									1.00E+00	1.00E+00	0	19	286	21447	0.00%	1.33%	0.00%	0.09%	gluconeogenesis Jan 31	
									6.00E-03	4.93E-02	2	9	286	21447	22.22%	1.33%	0.70%	0.04%	glycolysis Jan 31	Hkdc1, Pkm
				MP:0003731	abnormal retina outer nuclear layer morphology	6.00E-03	1.00E+00	6.00E-03	1.00E+00	1.00E+00	0	11	286	21447	0.00%	1.33%	0.00%	0.05%	shared jan 31	
									6.13E-03	7.32E-02	6	19	2002	21447	31.58%	9.33%	0.30%	0.09%	gluconeogenesis Jan 31	G6pc, Pck1, Pcx, Slc25a13, Slc37a1, Slc37a4
									6.49E-03	5.00E-02	4	9	2002	21447	44.44%	9.33%	0.20%	0.04%	glycolysis Jan 31	Gck, Hk1, Hkdc1, Pfkm
				MP:0005370	liver/biliary system phenotype	6.13E-03	7.59E-02	8.07E-02	7.59E-02	2.19E-01	3	11	2002	21447	27.27%	9.33%	0.15%	0.05%	shared jan 31	Aldob, Gapdh, Gpi1
									1.00E+00	1.00E+00	0	19	12	21447	0.00%	0.06%	0.00%	0.09%	gluconeogenesis Jan 31	
									1.00E+00	1.00E+00	0	9	12	21447	0.00%	0.06%	0.00%	0.04%	glycolysis Jan 31	
				MP:0008091	decreased T-helper 2 cell number	6.14E-03	1.00E+00	6.14E-03	6.14E-03	7.43E-02	1	11	12	21447	9.09%	0.06%	8.33%	0.05%	shared jan 31	Pgam1
									6.19E-03	7.32E-02	1	19	7	21447	5.26%	0.03%	14.29%	0.09%	gluconeogenesis Jan 31	Slc25a12
									1.00E+00	1.00E+00	0	9	7	21447	0.00%	0.03%	0.00%	0.04%	glycolysis Jan 31	
				MP:0001400	hyperresponsive	6.19E-03	1.00E+00	6.19E-03	1.00E+00	1.00E+00	0	11	7	21447	0.00%	0.03%	0.00%	0.05%	shared jan 31	
									6.19E-03	7.32E-02	1	19	7	21447	5.26%	0.03%	14.29%	0.09%	gluconeogenesis Jan 31	G6pc2
									1.00E+00	1.00E+00	0	9	7	21447	0.00%	0.03%	0.00%	0.04%	glycolysis Jan 31	
				MP:0002869	increased anti-insulin autoantibody level	6.19E-03	1.00E+00	6.19E-03	1.00E+00	1.00E+00	0	11	7	21447	0.00%	0.03%	0.00%	0.05%	shared jan 31	
									1.00E+00	1.00E+00	0	19	15	21447	0.00%	0.07%	0.00%	0.09%	gluconeogenesis Jan 31	
									6.28E-03	4.93E-02	1	9	15	21447	11.11%	0.07%	6.67%	0.04%	glycolysis Jan 31	Pfkl
				MP:0008859	abnormal hair cycle catagen phase	6.28E-03	1.00E+00	6.28E-03	1.00E+00	1.00E+00	0	11	15	21447	0.00%	0.07%	0.00%	0.05%	shared jan 31	

					1.00E+00	1.00E+00	0	19	15	21447	0.00%	0.07%	0.00%	0.09%	gluconeogenesis Jan 31	
					6.28E-03	4.93E-02	1	9	15	21447	11.11%	0.07%	6.67%	0.04%	glycolysis Jan 31	Pfkm
MP:0013161	enhanced muscle regeneration	6.28E-03	1.00E+00	6.28E-03	1.00E+00	1.00E+00	0	11	15	21447	0.00%	0.07%	0.00%	0.05%	shared jan 31	
					1.00E+00	1.00E+00	0	19	15	21447	0.00%	0.07%	0.00%	0.09%	gluconeogenesis Jan 31	
					6.28E-03	4.93E-02	1	9	15	21447	11.11%	0.07%	6.67%	0.04%	glycolysis Jan 31	Hk1
MP:0010375	increased kidney iron level	6.28E-03	1.00E+00	6.28E-03	1.00E+00	1.00E+00	0	11	15	21447	0.00%	0.07%	0.00%	0.05%	shared jan 31	
					1.00E+00	1.00E+00	0	19	239	21447	0.00%	1.11%	0.00%	0.09%	gluconeogenesis Jan 31	
					1.00E+00	1.00E+00	0	9	239	21447	0.00%	1.11%	0.00%	0.04%	glycolysis Jan 31	
MP:0005640	abnormal mean corpuscular hemoglobin concentration	6.37E-03	1.00E+00	6.37E-03	6.37E-03	7.43E-02	2	11	239	21447	18.18%	1.11%	0.84%	0.05%	shared jan 31	Gpi1, Tpi1
					6.63E-03	7.66E-02	2	19	139	21447	10.53%	0.65%	1.44%	0.09%	gluconeogenesis Jan 31	G6pc, Pcx
					1.00E+00	1.00E+00	0	9	139	21447	0.00%	0.65%	0.00%	0.04%	glycolysis Jan 31	
MP:0003917	increased kidney weight	6.63E-03	1.00E+00	6.63E-03	1.00E+00	1.00E+00	0	11	139	21447	0.00%	0.65%	0.00%	0.05%	shared jan 31	
					1.00E+00	1.00E+00	0	19	16	21447	0.00%	0.07%	0.00%	0.09%	gluconeogenesis Jan 31	
					6.70E-03	5.06E-02	1	9	16	21447	11.11%	0.07%	6.25%	0.04%	glycolysis Jan 31	Hk1
MP:0008742	abnormal kidney iron level	6.70E-03	1.00E+00	6.70E-03	1.00E+00	1.00E+00	0	11	16	21447	0.00%	0.07%	0.00%	0.05%	shared jan 31	
					6.73E-03	7.66E-02	2	19	140	21447	10.53%	0.65%	1.43%	0.09%	gluconeogenesis Jan 31	Pck1, Pcx
					5.72E-02	1.27E-01	1	9	140	21447	11.11%	0.65%	0.71%	0.04%	glycolysis Jan 31	Gck
MP:0001554	increased circulating free fatty acids level	6.73E-03	1.00E+00	6.73E-03	1.00E+00	1.00E+00	0	11	140	21447	0.00%	0.65%	0.00%	0.05%	shared jan 31	
					6.00E-01	6.45E-01	1	19	1008	21447	5.26%	4.70%	0.10%	0.09%	gluconeogenesis Jan 31	Slc37a2
					7.03E-03	5.21E-02	3	9	1008	21447	33.33%	4.70%	0.30%	0.04%	glycolysis Jan 31	Hk1, Pfkm, Pklr
MP:0000691	enlarged spleen	7.03E-03	6.00E-01	1.17E-02	9.16E-02	2.53E-01	2	11	1008	21447	18.18%	4.70%	0.20%	0.05%	shared jan 31	Gpi1, Tpi1
					1.00E+00	1.00E+00	0	19	313	21447	0.00%	1.46%	0.00%	0.09%	gluconeogenesis Jan 31	
					7.14E-03	5.21E-02	2	9	313	21447	22.22%	1.46%	0.64%	0.04%	glycolysis Jan 31	Hk1, Pklr
MP:0002874	decreased hemoglobin content	7.14E-03	1.00E+00	7.14E-03	1.07E-02	9.30E-02	2	11	313	21447	18.18%	1.46%	0.64%	0.05%	shared jan 31	Gapdh, Tpi1
					1.00E+00	1.00E+00	0	19	14	21447	0.00%	0.07%	0.00%	0.09%	gluconeogenesis Jan 31	
					1.00E+00	1.00E+00	0	9	14	21447	0.00%	0.07%	0.00%	0.04%	glycolysis Jan 31	
MP:0010220	decreased T-helper 17 cell number	7.16E-03	1.00E+00	7.16E-03	7.16E-03	8.02E-02	1	11	14	21447	9.09%	0.07%	7.14%	0.05%	shared jan 31	Pgam1
					2.49E-01	3.83E-01	1	19	320	21447	5.26%	1.49%	0.31%	0.09%	gluconeogenesis Jan 31	Slc25a13
					7.45E-03	5.22E-02	2	9	320	21447	22.22%	1.49%	0.62%	0.04%	glycolysis Jan 31	Gck, Pfkm
MP:0002727	decreased circulating insulin level	7.45E-03	1.00E+00	7.45E-03	1.00E+00	1.00E+00	0	11	320	21447	0.00%	1.49%	0.00%	0.05%	shared jan 31	
					1.00E+00	1.00E+00	0	19	18	21447	0.00%	0.08%	0.00%	0.09%	gluconeogenesis Jan 31	
					7.53E-03	5.22E-02	1	9	18	21447	11.11%	0.08%	5.56%	0.04%	glycolysis Jan 31	Pklr
MP:0010751	decreased susceptibility to parasitic infection induced morbidity/mortality	7.53E-03	1.00E+00	7.53E-03	1.00E+00	1.00E+00	0	11	18	21447	0.00%	0.08%	0.00%	0.05%	shared jan 31	
					1.00E+00	1.00E+00	0	19	18	21447	0.00%	0.08%	0.00%	0.09%	gluconeogenesis Jan 31	
					7.53E-03	5.22E-02	1	9	18	21447	11.11%	0.08%	5.56%	0.04%	glycolysis Jan 31	Gck
MP:0011819	increased pancreatic beta cell proliferation	7.53E-03	1.00E+00	7.53E-03	1.00E+00	1.00E+00	0	11	18	21447	0.00%	0.08%	0.00%	0.05%	shared jan 31	
					7.95E-03	8.55E-02	1	19	9	21447	5.26%	0.04%	11.11%	0.09%	gluconeogenesis Jan 31	Pck1
					1.00E+00	1.00E+00	0	9	9	21447	0.00%	0.04%	0.00%	0.04%	glycolysis Jan 31	
MP:0009126	abnormal brown fat cell number	7.95E-03	1.00E+00	7.95E-03	1.00E+00	1.00E+00	0	11	9	21447	0.00%	0.04%	0.00%	0.05%	shared jan 31	
					7.95E-03	8.55E-02	1	19	9	21447	5.26%	0.04%	11.11%	0.09%	gluconeogenesis Jan 31	Slc25a12
					1.00E+00	1.00E+00	0	9	9	21447	0.00%	0.04%	0.00%	0.04%	glycolysis Jan 31	
MP:0030733	decreased gamma-aminobutyric acid level	7.95E-03	1.00E+00	7.95E-03	1.00E+00	1.00E+00	0	11	9	21447	0.00%	0.04%	0.00%	0.05%	shared jan 31	
					7.95E-03	8.55E-02	1	19	9	21447	5.26%	0.04%	11.11%	0.09%	gluconeogenesis Jan 31	G6pc3
					1.00E+00	1.00E+00	0	9	9	21447	0.00%	0.04%	0.00%	0.04%	glycolysis Jan 31	
MP:0005074	impaired granulocyte bactericidal activity	7.95E-03	1.00E+00	7.95E-03	1.00E+00	1.00E+00	0	11	9	21447	0.00%	0.04%	0.00%	0.05%	shared jan 31	
					1.00E+00	1.00E+00	0	19	16	21447	0.00%	0.07%	0.00%	0.09%	gluconeogenesis Jan 31	
					1.00E+00	1.00E+00	0	9	16	21447	0.00%	0.07%	0.00%	0.04%	glycolysis Jan 31	
MP:0009834	abnormal sperm annulus morphology	8.18E-03	1.00E+00	8.18E-03	8.18E-03	8.36E-02	1	11	16	21447	9.09%	0.07%	6.25%	0.05%	shared jan 31	Eno4
					1.00E+00	1.00E+00	0	19	20	21447	0.00%	0.09%	0.00%	0.09%	gluconeogenesis Jan 31	
					8.36E-03	5.70E-02	1	9	20	21447	11.11%	0.09%	5.00%	0.04%	glycolysis Jan 31	Hk2
MP:0009977	abnormal cerebellar granule cell migration	8.36E-03	1.00E+00	8.36E-03	1.00E+00	1.00E+00	0	11	20	21447	0.00%	0.09%	0.00%	0.05%	shared jan 31	
					8.39E-03	8.86E-02	2	19	157	21447	10.53%	0.73%	1.27%	0.09%	gluconeogenesis Jan 31	G6pc3, Slc37a4
					1.00E+00	1.00E+00	0	9	157	21447	0.00%	0.73%	0.00%	0.04%	glycolysis Jan 31	
MP:0000222	decreased neutrophil cell number	8.39E-03	1.00E+00	8.39E-03	1.00E+00	1.00E+00	0	11	157	21447	0.00%	0.73%	0.00%	0.05%	shared jan 31	
					8.83E-03	8.86E-02	1	19	10	21447	5.26%	0.05%	10.00%	0.09%	gluconeogenesis Jan 31	Slc25a13
					1.00E+00	1.00E+00	0	9	10	21447	0.00%	0.05%	0.00%	0.04%	glycolysis Jan 31	
MP:0004369	absent utricle	8.83E-03	1.00E+00	8.83E-03	1.00E+00	1.00E+00	0	11	10	21447	0.00%	0.05%	0.00%	0.05%	shared jan 31	
					8.83E-03	8.86E-02	1	19	10	21447	5.26%	0.05%	10.00%	0.09%	gluconeogenesis Jan 31	Pck1
					1.00E+00	1.00E+00	0	9	10	21447	0.00%	0.05%	0.00%	0.04%	glycolysis Jan 31	
MP:0009122	decreased white fat cell lipid droplet size	8.83E-03	1.00E+00	8.83E-03	1.00E+00	1.00E+00	0	11	10	21447	0.00%	0.05%	0.00%	0.05%	shared jan 31	
					8.83E-03	8.86E-02	1	19	10	21447	5.26%	0.05%	10.00%	0.09%	gluconeogenesis Jan 31	G6pc
					1.00E+00	1.00E+00	0	9	10	21447	0.00%	0.05%	0.00%	0.04%	glycolysis Jan 31	
MP:0009810	increased urine uric acid level	8.83E-03	1.00E+00	8.83E-03	1.00E+00	1.00E+00	0	11	10	21447	0.00%	0.05%	0.00%	0.05%	shared jan 31	
					9.45E-03	9.32E-02	3	19	504	21447	15.79%	2.35%	0.60%	0.09%	gluconeogenesis Jan 31	G6pc, Slc25a12, Slc37a4
					1.00E+00	1.00E+00	0	9	504	21447	0.00%	2.35%	0.00%	0.04%	glycolysis Jan 31	
MP:0002064	seizures	9.45E-03	1.00E+00	9.45E-03	1.00E+00	1.00E+00	0	11	504	21447	0.00%	2.35%	0.00%	0.05%	shared jan 31	
					1.00E+00	1.00E+00	0	19	307	21447	0.00%	1.43%	0.00%	0.09%	gluconeogenesis Jan 31	
					1.00E+00	1.00E+00	0	9	307	21447	0.00%	1.43%	0.00%	0.04%	glycolysis Jan 31	
MP:0011096	embryonic lethality between implantation and somite formation, complete penetrance	1.03E-02	1.00E+00	1.03E-02	1.03E-02	9.30E-02	2	11	307	21447	18.18%	1.43%	0.65%	0.05%	shared jan 31	Eno1, Tpi1
					5.69E-01	6.20E-01	1	19	930	21447	5.26%	4.34%	0.11%	0.09%	gluconeogenesis Jan 31	Slc37a4

					3.29E-01	3.88E-01	1	9	930	21447	11.11%	4.34%	0.11%	0.04%	glycolysis Jan 31	Hk2
MP:0020846	abnormal cell motility	1.03E-02	5.69E-01	1.81E-02	1.03E-02	9.30E-02	3	11	930	21447	27.27%	4.34%	0.32%	0.05%	shared jan 31	Eno4, Gapdhs, Pgk2
					1.03E-02	9.64E-02	2	19	175	21447	10.53%	0.82%	1.14%	0.09%	gluconeogenesis Jan 31	G6pc3, Slc37a4
					1.00E+00	1.00E+00	0	9	175	21447	0.00%	0.82%	0.00%	0.04%	glycolysis Jan 31	
MP:0002463	abnormal neutrophil physiology	1.03E-02	1.00E+00	1.03E-02	1.00E+00	1.00E+00	0	11	175	21447	0.00%	0.82%	0.00%	0.05%	shared jan 31	
					1.00E+00	1.00E+00	0	19	380	21447	0.00%	1.77%	0.00%	0.09%	gluconeogenesis Jan 31	
					1.04E-02	6.79E-02	2	9	380	21447	22.22%	1.77%	0.53%	0.04%	glycolysis Jan 31	Hk1, Pklr
MP:0002875	decreased erythrocyte cell number	1.04E-02	1.00E+00	1.04E-02	1.55E-02	1.02E-01	2	11	380	21447	18.18%	1.77%	0.53%	0.05%	shared jan 31	Gpi1, Tpi1
					1.00E+00	1.00E+00	0	19	25	21447	0.00%	0.12%	0.00%	0.09%	gluconeogenesis Jan 31	
					1.04E-02	6.79E-02	1	9	25	21447	11.11%	0.12%	4.00%	0.04%	glycolysis Jan 31	Pfkm
MP:0010401	increased skeletal muscle glycogen level	1.04E-02	1.00E+00	1.04E-02	1.00E+00	1.00E+00	0	11	25	21447	0.00%	0.12%	0.00%	0.05%	shared jan 31	
					1.00E+00	1.00E+00	0	19	25	21447	0.00%	0.12%	0.00%	0.09%	gluconeogenesis Jan 31	
					1.04E-02	6.79E-02	1	9	25	21447	11.11%	0.12%	4.00%	0.04%	glycolysis Jan 31	Pfkm
MP:0009414	skeletal muscle fiber necrosis	1.04E-02	1.00E+00	1.04E-02	1.00E+00	1.00E+00	0	11	25	21447	0.00%	0.12%	0.00%	0.05%	shared jan 31	
					1.05E-02	9.64E-02	3	19	524	21447	15.79%	2.44%	0.57%	0.09%	gluconeogenesis Jan 31	Pck1, Slc25a12, Slc25a13
					1.00E+00	1.00E+00	0	9	524	21447	0.00%	2.44%	0.00%	0.04%	glycolysis Jan 31	
MP:0005332	abnormal amino acid level	1.05E-02	1.00E+00	1.05E-02	1.00E+00	1.00E+00	0	11	524	21447	0.00%	2.44%	0.00%	0.05%	shared jan 31	
					1.06E-02	9.64E-02	1	19	12	21447	5.26%	0.06%	8.33%	0.09%	gluconeogenesis Jan 31	Slc25a12
					1.00E+00	1.00E+00	0	9	12	21447	0.00%	0.06%	0.00%	0.04%	glycolysis Jan 31	
MP:0030731	abnormal gamma-aminobutyric acid level	1.06E-02	1.00E+00	1.06E-02	1.00E+00	1.00E+00	0	11	12	21447	0.00%	0.06%	0.00%	0.05%	shared jan 31	
					1.06E-02	9.64E-02	1	19	12	21447	5.26%	0.06%	8.33%	0.09%	gluconeogenesis Jan 31	Slc25a13
					1.00E+00	1.00E+00	0	9	12	21447	0.00%	0.06%	0.00%	0.04%	glycolysis Jan 31	
MP:0000041	absent endolymphatic duct	1.06E-02	1.00E+00	1.06E-02	1.00E+00	1.00E+00	0	11	12	21447	0.00%	0.06%	0.00%	0.05%	shared jan 31	
					1.06E-02	9.64E-02	1	19	12	21447	5.26%	0.06%	8.33%	0.09%	gluconeogenesis Jan 31	Pcx
					1.00E+00	1.00E+00	0	9	12	21447	0.00%	0.06%	0.00%	0.04%	glycolysis Jan 31	
MP:0030973	impaired gluconeogenesis	1.06E-02	1.00E+00	1.06E-02	1.00E+00	1.00E+00	0	11	12	21447	0.00%	0.06%	0.00%	0.05%	shared jan 31	
					1.09E-02	9.78E-02	2	19	180	21447	10.53%	0.84%	1.11%	0.09%	gluconeogenesis Jan 31	Got2, Mdh1
					1.00E+00	1.00E+00	0	9	180	21447	0.00%	0.84%	0.00%	0.04%	glycolysis Jan 31	
MP:0002884	abnormal pharyngeal arch morphology	1.09E-02	1.00E+00	1.09E-02	1.00E+00	1.00E+00	0	11	180	21447	0.00%	0.84%	0.00%	0.05%	shared jan 31	
					1.00E+00	1.00E+00	0	19	390	21447	0.00%	1.82%	0.00%	0.09%	gluconeogenesis Jan 31	
					1.09E-02	6.98E-02	2	9	390	21447	22.22%	1.82%	0.51%	0.04%	glycolysis Jan 31	Hk1, Pklr
MP:0001577	anemia	1.09E-02	1.00E+00	1.09E-02	1.83E-01	3.73E-01	1	11	390	21447	9.09%	1.82%	0.26%	0.05%	shared jan 31	Tpi1
					1.45E-02	1.07E-01	10	19	5731	21447	52.63%	26.72%	0.17%	0.09%	gluconeogenesis Jan 31	Fbp2, Got1, Got2, Mdh1, Mdh2, Pck1, Pcx, Slc25a11, Slc25a12, Slc37a4
					1.41E-02	7.28E-02	6	9	5731	21447	66.67%	26.72%	0.10%	0.04%	glycolysis Jan 31	Gck, Hk2, Hkdc1, Pfkl, Pfkm, Pkm
MP:0010770	preweaning lethality	1.11E-02	1.45E-02	7.71E-01	1.11E-02	9.30E-02	7	11	5731	21447	63.64%	26.72%	0.12%	0.05%	shared jan 31	Aldoa, Aldob, Eno1, Gapdh, Gpi1, Pgam1, Tpi1
					1.00E+00	1.00E+00	0	19	22	21447	0.00%	0.10%	0.00%	0.09%	gluconeogenesis Jan 31	
					1.00E+00	1.00E+00	0	9	22	21447	0.00%	0.10%	0.00%	0.04%	glycolysis Jan 31	
MP:0002811	macrocytic anemia	1.12E-02	1.00E+00	1.12E-02	1.12E-02	9.30E-02	1	11	22	21447	9.09%	0.10%	4.55%	0.05%	shared jan 31	Tpi1
					1.00E+00	1.00E+00	0	19	22	21447	0.00%	0.10%	0.00%	0.09%	gluconeogenesis Jan 31	
					1.00E+00	1.00E+00	0	9	22	21447	0.00%	0.10%	0.00%	0.04%	glycolysis Jan 31	
MP:0000248	macrocytosis	1.12E-02	1.00E+00	1.12E-02	1.12E-02	9.30E-02	1	11	22	21447	9.09%	0.10%	4.55%	0.05%	shared jan 31	Tpi1
					1.00E+00	1.00E+00	0	19	396	21447	0.00%	1.85%	0.00%	0.09%	gluconeogenesis Jan 31	
					1.12E-02	7.01E-02	2	9	396	21447	22.22%	1.85%	0.51%	0.04%	glycolysis Jan 31	Hk1, Pfkm
MP:0004951	abnormal spleen weight	1.12E-02	1.00E+00	1.12E-02	1.68E-02	1.02E-01	2	11	396	21447	18.18%	1.85%	0.51%	0.05%	shared jan 31	Gpi1, Tpi1
					1.00E+00	1.00E+00	0	19	397	21447	0.00%	1.85%	0.00%	0.09%	gluconeogenesis Jan 31	
					1.13E-02	7.01E-02	2	9	397	21447	22.22%	1.85%	0.50%	0.04%	glycolysis Jan 31	Pfkm, Pklr
MP:0013707	abnormal hematopoietic precursor cell morphology	1.13E-02	1.00E+00	1.13E-02	1.00E+00	1.00E+00	0	11	397	21447	0.00%	1.85%	0.00%	0.05%	shared jan 31	
					1.21E-02	1.07E-01	12	19	7559	21447	63.16%	35.25%	0.16%	0.09%	gluconeogenesis Jan 31	Fbp2, G6pc, G6pc3, Got1, Got2, Mdh1, Mdh2, Pck1, Pcx, Slc25a11, Slc25a12, Slc37a4
					1.17E-02	7.09E-02	7	9	7559	21447	77.78%	35.25%	0.09%	0.04%	glycolysis Jan 31	Gck, Hk2, Hkdc1, Pfkl, Pfkm, Pklr, Pkm
MP:0010768	mortality/aging	1.17E-02	5.20E-02	2.24E-01	5.20E-02	1.74E-01	7	11	7559	21447	63.64%	35.25%	0.09%	0.05%	shared jan 31	Aldoa, Aldob, Eno1, Gapdh, Gpi1, Pgam1, Tpi1
					5.03E-02	1.97E-01	2	19	410	21447	10.53%	1.91%	0.49%	0.09%	gluconeogenesis Jan 31	G6pc2, Pcx
					1.20E-02	7.09E-02	2	9	410	21447	22.22%	1.91%	0.49%	0.04%	glycolysis Jan 31	Gck, Pfkm
MP:0002693	abnormal pancreas physiology	1.20E-02	1.00E+00	1.20E-02	1.00E+00	1.00E+00	0	11	410	21447	0.00%	1.91%	0.00%	0.05%	shared jan 31	
					1.53E-01	3.15E-01	4	19	2381	21447	21.05%	11.10%	0.17%	0.09%	gluconeogenesis Jan 31	Got2, Pck2, Slc37a2, Slc37a4
					1.21E-02	7.09E-02	4	9	2381	21447	44.44%	11.10%	0.17%	0.04%	glycolysis Jan 31	Hk1, Hk3, Pfkm, Pklr
MP:0002722	abnormal immune system organ morphology	1.21E-02	3.50E-01	3.45E-02	3.50E-01	4.88E-01	2	11	2381	21447	18.18%	11.10%	0.08%	0.05%	shared jan 31	Gpi1, Tpi1
					1.00E+00	1.00E+00	0	19	29	21447	0.00%	0.14%	0.00%	0.09%	gluconeogenesis Jan 31	
					1.21E-02	7.09E-02	1	9	29	21447	11.11%	0.14%	3.45%	0.04%	glycolysis Jan 31	Pfkm
MP:0014071	increased cardiac muscle glycogen level	1.21E-02	1.00E+00	1.21E-02	1.00E+00	1.00E+00	0	11	29	21447	0.00%	0.14%	0.00%	0.05%	shared jan 31	
					1.18E-01	2.81E-01	5	19	3020	21447	26.32%	14.08%	0.17%	0.09%	gluconeogenesis Jan 31	Got1, Got2, Mdh1, Mdh2, Slc25a11
					1.22E-01	1.99E-01	3	9	3020	21447	33.33%	14.08%	0.10%	0.04%	glycolysis Jan 31	Gck, Hk2, Pkm
MP:0002080	prenatal lethality	1.22E-02	1.22E-01	9.99E-02	1.22E-02	9.30E-02	5	11	3020	21447	45.45%	14.08%	0.17%	0.05%	shared jan 31	Eno1, Gapdh, Gpi1, Pgam1, Tpi1
					1.00E+00	1.00E+00	0	19	335	21447	0.00%	1.56%	0.00%	0.09%	gluconeogenesis Jan 31	
					1.00E+00	1.00E+00	0	9	335	21447	0.00%	1.56%	0.00%	0.04%	glycolysis Jan 31	
MP:0010831	lethality, incomplete penetrance	1.22E-02	1.00E+00	1.22E-02	1.22E-02	9.30E-02	2	11	335	21447	18.18%	1.56%	0.60%	0.05%	shared jan 31	Gapdh, Gpi1
					1.00E+00	1.00E+00	0	19	24	21447	0.00%	0.11%	0.00%	0.09%	gluconeogenesis Jan 31	
					1.00E+00	1.00E+00	0	9	24	21447	0.00%	0.11%	0.00%	0.04%	glycolysis Jan 31	
MP:0011106	embryonic lethality between implantation and somite formation, incomplete penetrance	1.22E-02	1.00E+00	1.22E-02	1.22E-02	9.30E-02	1	11	24	21447	9.09%	0.11%	4.17%	0.05%	shared jan 31	Gapdh
					1.23E-02	1.07E-01	1	19	14	21447	5.26%	0.07%	7.14%	0.09%	gluconeogenesis Jan 31	Slc25a12

					1.00E+00	1.00E+00	0	9	14	21447	0.00%	0.07%	0.00%	0.04%	glycolysis Jan 31	
MP:0030634	abnormal taurine level	1.23E-02	1.00E+00	1.23E-02	1.00E+00	1.00E+00	0	11	14	21447	0.00%	0.07%	0.00%	0.05%	shared jan 31	
					1.00E+00	1.00E+00	0	19	30	21447	0.00%	0.14%	0.00%	0.09%	gluconeogenesis Jan 31	
					1.25E-02	7.23E-02	1	9	30	21447	11.11%	0.14%	3.33%	0.04%	glycolysis Jan 31	Gck
MP:0010087	increased circulating fructosamine level	1.25E-02	1.00E+00	1.25E-02	1.00E+00	1.00E+00	0	11	30	21447	0.00%	0.14%	0.00%	0.05%	shared jan 31	
					1.26E-02	1.07E-01	2	19	194	21447	10.53%	0.90%	1.03%	0.09%	gluconeogenesis Jan 31	Pck1, Pcx
					7.85E-02	1.56E-01	1	9	194	21447	11.11%	0.90%	0.52%	0.04%	glycolysis Jan 31	Gck
MP:0005281	increased fatty acids level	1.26E-02	1.00E+00	1.26E-02	1.00E+00	1.00E+00	0	11	194	21447	0.00%	0.90%	0.00%	0.05%	shared jan 31	
					1.27E-02	1.07E-01	2	19	195	21447	10.53%	0.91%	1.03%	0.09%	gluconeogenesis Jan 31	Fbp2, Slc25a10
					1.00E+00	1.00E+00	0	9	195	21447	0.00%	0.91%	0.00%	0.04%	glycolysis Jan 31	
MP:0010097	abnormal retina blood vessel morphology	1.27E-02	1.00E+00	1.27E-02	1.00E+00	1.00E+00	0	11	195	21447	0.00%	0.91%	0.00%	0.05%	shared jan 31	
					2.22E-02	1.22E-01	4	19	1253	21447	21.05%	5.84%	0.32%	0.09%	gluconeogenesis Jan 31	G6pc, Pck1, Slc37a2, Slc37a4
					1.28E-02	7.27E-02	3	9	1253	21447	33.33%	5.84%	0.24%	0.04%	glycolysis Jan 31	Hk1, Pfkm, Pklr
MP:0013328	visceromegaly	1.28E-02	1.32E-01	9.68E-02	1.32E-01	3.12E-01	2	11	1253	21447	18.18%	5.84%	0.16%	0.05%	shared jan 31	Gpi1, Tpi1
					1.00E+00	1.00E+00	0	19	31	21447	0.00%	0.14%	0.00%	0.09%	gluconeogenesis Jan 31	
					1.29E-02	7.27E-02	1	9	31	21447	11.11%	0.14%	3.23%	0.04%	glycolysis Jan 31	Pfkl
MP:0003413	hair follicle degeneration	1.29E-02	1.00E+00	1.29E-02	1.00E+00	1.00E+00	0	11	31	21447	0.00%	0.14%	0.00%	0.05%	shared jan 31	
					1.30E-02	1.07E-01	4	19	1068	21447	21.05%	4.98%	0.37%	0.09%	gluconeogenesis Jan 31	G6pc, G6pc3, Slc25a12, Slc37a4
					3.69E-01	4.26E-01	1	9	1068	21447	11.11%	4.98%	0.09%	0.04%	glycolysis Jan 31	Gck
MP:0001732	postnatal growth retardation	1.30E-02	1.00E+00	1.30E-02	1.00E+00	1.00E+00	0	11	1068	21447	0.00%	4.98%	0.00%	0.05%	shared jan 31	
					1.00E+00	1.00E+00	0	19	1013	21447	0.00%	4.72%	0.00%	0.09%	gluconeogenesis Jan 31	
					1.00E+00	1.00E+00	0	9	1013	21447	0.00%	4.72%	0.00%	0.04%	glycolysis Jan 31	
MP:0001925	male infertility	1.30E-02	1.00E+00	1.30E-02	1.30E-02	9.55E-02	3	11	1013	21447	27.27%	4.72%	0.30%	0.05%	shared jan 31	Eno4, Gapdhs, Pgk2
					1.32E-02	1.07E-01	1	19	15	21447	5.26%	0.07%	6.67%	0.09%	gluconeogenesis Jan 31	G6pc
					1.00E+00	1.00E+00	0	9	15	21447	0.00%	0.07%	0.00%	0.04%	glycolysis Jan 31	
MP:0011435	increased urine magnesium level	1.32E-02	1.00E+00	1.32E-02	1.00E+00	1.00E+00	0	11	15	21447	0.00%	0.07%	0.00%	0.05%	shared jan 31	
					1.32E-02	1.07E-01	1	19	15	21447	5.26%	0.07%	6.67%	0.09%	gluconeogenesis Jan 31	Pck1
					1.00E+00	1.00E+00	0	9	15	21447	0.00%	0.07%	0.00%	0.04%	glycolysis Jan 31	
MP:0011174	lipodystrophy	1.32E-02	1.00E+00	1.32E-02	1.00E+00	1.00E+00	0	11	15	21447	0.00%	0.07%	0.00%	0.05%	shared jan 31	
					1.00E+00	1.00E+00	0	19	32	21447	0.00%	0.15%	0.00%	0.09%	gluconeogenesis Jan 31	
					1.34E-02	7.28E-02	1	9	32	21447	11.11%	0.15%	3.12%	0.04%	glycolysis Jan 31	Pfkm
MP:0009412	skeletal muscle fiber degeneration	1.34E-02	1.00E+00	1.34E-02	1.00E+00	1.00E+00	0	11	32	21447	0.00%	0.15%	0.00%	0.05%	shared jan 31	
					1.00E+00	1.00E+00	0	19	27	21447	0.00%	0.13%	0.00%	0.09%	gluconeogenesis Jan 31	
					1.00E+00	1.00E+00	0	9	27	21447	0.00%	0.13%	0.00%	0.04%	glycolysis Jan 31	
MP:0003806	abnormal nucleotide metabolism	1.38E-02	1.00E+00	1.38E-02	1.38E-02	9.85E-02	1	11	27	21447	9.09%	0.13%	3.70%	0.05%	shared jan 31	Pgam1
					1.00E+00	1.00E+00	0	19	33	21447	0.00%	0.15%	0.00%	0.09%	gluconeogenesis Jan 31	
					1.38E-02	7.28E-02	1	9	33	21447	11.11%	0.15%	3.03%	0.04%	glycolysis Jan 31	Pfkm
MP:0014070	abnormal cardiac muscle glycogen level	1.38E-02	1.00E+00	1.38E-02	1.00E+00	1.00E+00	0	11	33	21447	0.00%	0.15%	0.00%	0.05%	shared jan 31	
					1.00E+00	1.00E+00	0	19	33	21447	0.00%	0.15%	0.00%	0.09%	gluconeogenesis Jan 31	
					1.38E-02	7.28E-02	1	9	33	21447	11.11%	0.15%	3.03%	0.04%	glycolysis Jan 31	Pklr
MP:0011178	increased erythroblast number	1.38E-02	1.00E+00	1.38E-02	1.00E+00	1.00E+00	0	11	33	21447	0.00%	0.15%	0.00%	0.05%	shared jan 31	
					1.00E+00	1.00E+00	0	19	33	21447	0.00%	0.15%	0.00%	0.09%	gluconeogenesis Jan 31	
					1.38E-02	7.28E-02	1	9	33	21447	11.11%	0.15%	3.03%	0.04%	glycolysis Jan 31	Gck
MP:0009113	increased pancreatic beta cell mass	1.38E-02	1.00E+00	1.38E-02	1.00E+00	1.00E+00	0	11	33	21447	0.00%	0.15%	0.00%	0.05%	shared jan 31	
					1.41E-02	1.07E-01	1	19	16	21447	5.26%	0.07%	6.25%	0.09%	gluconeogenesis Jan 31	G6pc
					1.00E+00	1.00E+00	0	9	16	21447	0.00%	0.07%	0.00%	0.04%	glycolysis Jan 31	
MP:0009350	decreased urine pH	1.41E-02	1.00E+00	1.41E-02	1.00E+00	1.00E+00	0	11	16	21447	0.00%	0.07%	0.00%	0.05%	shared jan 31	
					1.41E-02	1.07E-01	1	19	16	21447	5.26%	0.07%	6.25%	0.09%	gluconeogenesis Jan 31	Pck1
					1.00E+00	1.00E+00	0	9	16	21447	0.00%	0.07%	0.00%	0.04%	glycolysis Jan 31	
MP:0005309	increased circulating ammonia level	1.41E-02	1.00E+00	1.41E-02	1.00E+00	1.00E+00	0	11	16	21447	0.00%	0.07%	0.00%	0.05%	shared jan 31	
					1.41E-02	1.07E-01	1	19	16	21447	5.26%	0.07%	6.25%	0.09%	gluconeogenesis Jan 31	G6pc
					1.00E+00	1.00E+00	0	9	16	21447	0.00%	0.07%	0.00%	0.04%	glycolysis Jan 31	
MP:0011363	renal glomerulus atrophy	1.41E-02	1.00E+00	1.41E-02	1.00E+00	1.00E+00	0	11	16	21447	0.00%	0.07%	0.00%	0.05%	shared jan 31	
					1.00E+00	1.00E+00	0	19	34	21447	0.00%	0.16%	0.00%	0.09%	gluconeogenesis Jan 31	
					1.42E-02	7.28E-02	1	9	34	21447	11.11%	0.16%	2.94%	0.04%	glycolysis Jan 31	Pkm
MP:0011093	embryonic lethality at implantation, complete penetrance	1.42E-02	1.00E+00	1.42E-02	1.00E+00	1.00E+00	0	11	34	21447	0.00%	0.16%	0.00%	0.05%	shared jan 31	
					1.00E+00	1.00E+00	0	19	34	21447	0.00%	0.16%	0.00%	0.09%	gluconeogenesis Jan 31	
					1.42E-02	7.28E-02	1	9	34	21447	11.11%	0.16%	2.94%	0.04%	glycolysis Jan 31	Gck
MP:0011628	increased mitochondrial number	1.42E-02	1.00E+00	1.42E-02	1.00E+00	1.00E+00	0	11	34	21447	0.00%	0.16%	0.00%	0.05%	shared jan 31	
					1.42E-02	1.07E-01	2	19	207	21447	10.53%	0.97%	0.97%	0.09%	gluconeogenesis Jan 31	G6pc, Pck1
					1.00E+00	1.00E+00	0	9	207	21447	0.00%	0.97%	0.00%	0.04%	glycolysis Jan 31	
MP:0002981	increased liver weight	1.42E-02	1.00E+00	1.42E-02	1.01E-01	2.66E-01	1	11	207	21447	9.09%	0.97%	0.48%	0.05%	shared jan 31	Gpi1
					2.62E-01	3.96E-01	3	19	2013	21447	15.79%	9.39%	0.15%	0.09%	gluconeogenesis Jan 31	G6pc3, Got1, Slc37a4
					2.04E-01	2.80E-01	2	9	2013	21447	22.22%	9.39%	0.10%	0.04%	glycolysis Jan 31	Hk1, Pklr
MP:0011182	decreased hematopoietic cell number	1.49E-02	2.62E-01	5.69E-02	1.49E-02	1.02E-01	4	11	2013	21447	36.36%	9.39%	0.20%	0.05%	shared jan 31	Aldoa, Gpi1, Pgam1, Tpi1
					1.50E-02	1.07E-01	1	19	17	21447	5.26%	0.08%	5.88%	0.09%	gluconeogenesis Jan 31	Pcx
					1.00E+00	1.00E+00	0	9	17	21447	0.00%	0.08%	0.00%	0.04%	glycolysis Jan 31	
MP:0013242	abnormal amino acid metabolism	1.50E-02	1.00E+00	1.50E-02	1.00E+00	1.00E+00	0	11	17	21447	0.00%	0.08%	0.00%	0.05%	shared jan 31	
					1.50E-02	1.07E-01	1	19	17	21447	5.26%	0.08%	5.88%	0.09%	gluconeogenesis Jan 31	Slc25a12
	abnormal behavioral response to				1.00E+00	1.00E+00	0	9	17	21447	0.00%	0.08%	0.00%	0.04%	glycolysis Jan 31	

MP:0013894	abnormal behavioral response to amphetamine	1.50E-02	1.00E+00	1.50E-02	1.00E+00	1.00E+00	0	11	17	21447	0.00%	0.08%	0.00%	0.05%	shared jan 31	
					1.50E-02	1.07E-01	1	19	17	21447	5.26%	0.08%	5.88%	0.09%	gluconeogenesis Jan 31	Slc25a13
					1.00E+00	1.00E+00	0	9	17	21447	0.00%	0.08%	0.00%	0.04%	glycolysis Jan 31	
MP:0003069	abnormal superior semicircular canal morphology	1.50E-02	1.00E+00	1.50E-02	1.00E+00	1.00E+00	0	11	17	21447	0.00%	0.08%	0.00%	0.05%	shared jan 31	
					1.50E-02	1.07E-01	1	19	17	21447	5.26%	0.08%	5.88%	0.09%	gluconeogenesis Jan 31	G6pc
					1.00E+00	1.00E+00	0	9	17	21447	0.00%	0.08%	0.00%	0.04%	glycolysis Jan 31	
MP:0002901	increased urine phosphate level	1.50E-02	1.00E+00	1.50E-02	1.00E+00	1.00E+00	0	11	17	21447	0.00%	0.08%	0.00%	0.05%	shared jan 31	
					1.51E-02	1.07E-01	11	19	6733	21447	57.89%	31.39%	0.16%	0.09%	gluconeogenesis Jan 31	Fbp2, G6pc, Got1, Got2, Mdh1, Mdh2, Pck1, Pcx, Slc25a11, Slc25a12, Slc37a4
					3.16E-02	1.00E-01	6	9	6733	21447	66.67%	31.39%	0.09%	0.04%	glycolysis Jan 31	Gck, Hk2, Hkdc1, Pfkl, Pfkm, Pkm
MP:0010769	abnormal survival	1.51E-02	3.16E-02	4.77E-01	2.78E-02	1.24E-01	7	11	6733	21447	63.64%	31.39%	0.10%	0.05%	shared jan 31	Aldoa, Aldob, Eno1, Gapdh, Gpi1, Pgam1, Tpi1
					1.52E-02	1.07E-01	2	19	214	21447	10.53%	1.00%	0.93%	0.09%	gluconeogenesis Jan 31	G6pc, Pcx
					1.00E+00	1.00E+00	0	9	214	21447	0.00%	1.00%	0.00%	0.04%	glycolysis Jan 31	
MP:0002707	abnormal kidney weight	1.52E-02	1.00E+00	1.52E-02	1.00E+00	1.00E+00	0	11	214	21447	0.00%	1.00%	0.00%	0.05%	shared jan 31	
					1.00E+00	1.00E+00	0	19	30	21447	0.00%	0.14%	0.00%	0.09%	gluconeogenesis Jan 31	
					1.00E+00	1.00E+00	0	9	30	21447	0.00%	0.14%	0.00%	0.04%	glycolysis Jan 31	
MP:0008089	abnormal T-helper 2 cell number	1.53E-02	1.00E+00	1.53E-02	1.53E-02	1.02E-01	1	11	30	21447	9.09%	0.14%	3.33%	0.05%	shared jan 31	Pgam1
					3.44E-01	4.57E-01	1	19	470	21447	5.26%	2.19%	0.21%	0.09%	gluconeogenesis Jan 31	Pcx
					1.56E-02	7.90E-02	2	9	470	21447	22.22%	2.19%	0.43%	0.04%	glycolysis Jan 31	Gck, Pfkm
MP:0013561	abnormal endocrine gland physiology	1.56E-02	1.00E+00	1.56E-02	1.00E+00	1.00E+00	0	11	470	21447	0.00%	2.19%	0.00%	0.05%	shared jan 31	
					1.00E+00	1.00E+00	0	19	31	21447	0.00%	0.14%	0.00%	0.09%	gluconeogenesis Jan 31	
					1.00E+00	1.00E+00	0	9	31	21447	0.00%	0.14%	0.00%	0.04%	glycolysis Jan 31	
MP:0010218	abnormal T-helper 17 cell number	1.58E-02	1.00E+00	1.58E-02	1.58E-02	1.02E-01	1	11	31	21447	9.09%	0.14%	3.23%	0.05%	shared jan 31	Pgam1
					1.58E-02	1.09E-01	1	19	18	21447	5.26%	0.08%	5.56%	0.09%	gluconeogenesis Jan 31	Pck1
					1.00E+00	1.00E+00	0	9	18	21447	0.00%	0.08%	0.00%	0.04%	glycolysis Jan 31	
MP:0005308	abnormal circulating ammonia level	1.58E-02	1.00E+00	1.58E-02	1.00E+00	1.00E+00	0	11	18	21447	0.00%	0.08%	0.00%	0.05%	shared jan 31	
					1.58E-02	1.09E-01	1	19	18	21447	5.26%	0.08%	5.56%	0.09%	gluconeogenesis Jan 31	Pck1
					1.00E+00	1.00E+00	0	9	18	21447	0.00%	0.08%	0.00%	0.04%	glycolysis Jan 31	
MP:0009120	abnormal white fat cell lipid droplet size	1.58E-02	1.00E+00	1.58E-02	1.00E+00	1.00E+00	0	11	18	21447	0.00%	0.08%	0.00%	0.05%	shared jan 31	
					1.00E+00	1.00E+00	0	19	39	21447	0.00%	0.18%	0.00%	0.09%	gluconeogenesis Jan 31	
					1.63E-02	7.91E-02	1	9	39	21447	11.11%	0.18%	2.56%	0.04%	glycolysis Jan 31	Pfkm
MP:0002944	increased lactate dehydrogenase level	1.63E-02	1.00E+00	1.63E-02	1.00E+00	1.00E+00	0	11	39	21447	0.00%	0.18%	0.00%	0.05%	shared jan 31	
					1.00E+00	1.00E+00	0	19	39	21447	0.00%	0.18%	0.00%	0.09%	gluconeogenesis Jan 31	
					1.63E-02	7.91E-02	1	9	39	21447	11.11%	0.18%	2.56%	0.04%	glycolysis Jan 31	Hk1
MP:0005097	polychromatophilia	1.63E-02	1.00E+00	1.63E-02	1.98E-02	1.11E-01	1	11	39	21447	9.09%	0.18%	2.56%	0.05%	shared jan 31	Tpi1
					1.00E+00	1.00E+00	0	19	40	21447	0.00%	0.19%	0.00%	0.09%	gluconeogenesis Jan 31	
					1.67E-02	7.91E-02	1	9	40	21447	11.11%	0.19%	2.50%	0.04%	glycolysis Jan 31	Pfkm
MP:0005039	hypoxia	1.67E-02	1.00E+00	1.67E-02	1.00E+00	1.00E+00	0	11	40	21447	0.00%	0.19%	0.00%	0.05%	shared jan 31	
					1.67E-02	1.12E-01	1	19	19	21447	5.26%	0.09%	5.26%	0.09%	gluconeogenesis Jan 31	G6pc
					1.00E+00	1.00E+00	0	9	19	21447	0.00%	0.09%	0.00%	0.04%	glycolysis Jan 31	
MP:0002959	increased urine microalbumin level	1.67E-02	1.00E+00	1.67E-02	1.00E+00	1.00E+00	0	11	19	21447	0.00%	0.09%	0.00%	0.05%	shared jan 31	
					1.00E+00	1.00E+00	0	19	33	21447	0.00%	0.15%	0.00%	0.09%	gluconeogenesis Jan 31	
					1.00E+00	1.00E+00	0	9	33	21447	0.00%	0.15%	0.00%	0.04%	glycolysis Jan 31	
MP:0030590	abnormal sperm fibrous sheath morphology	1.68E-02	1.00E+00	1.68E-02	1.68E-02	1.02E-01	1	11	33	21447	9.09%	0.15%	3.03%	0.05%	shared jan 31	Eno4
					1.68E-02	1.12E-01	2	19	226	21447	10.53%	1.05%	0.88%	0.09%	gluconeogenesis Jan 31	G6pc, Slc37a4
					1.00E+00	1.00E+00	0	9	226	21447	0.00%	1.05%	0.00%	0.04%	glycolysis Jan 31	
MP:0003055	abnormal long bone epiphyseal plate morphology	1.68E-02	1.00E+00	1.68E-02	1.00E+00	1.00E+00	0	11	226	21447	0.00%	1.05%	0.00%	0.05%	shared jan 31	
					1.00E+00	1.00E+00	0	19	493	21447	0.00%	2.30%	0.00%	0.09%	gluconeogenesis Jan 31	
					1.71E-02	7.91E-02	2	9	493	21447	22.22%	2.30%	0.41%	0.04%	glycolysis Jan 31	Hk1, Pklr
MP:0000226	abnormal mean corpuscular volume	1.71E-02	1.00E+00	1.71E-02	2.53E-02	1.21E-01	2	11	493	21447	18.18%	2.30%	0.41%	0.05%	shared jan 31	Gpi1, Tpi1
					1.00E+00	1.00E+00	0	19	41	21447	0.00%	0.19%	0.00%	0.09%	gluconeogenesis Jan 31	
					1.71E-02	7.91E-02	1	9	41	21447	11.11%	0.19%	2.44%	0.04%	glycolysis Jan 31	Pfkm
MP:0014069	abnormal skeletal muscle glycogen level	1.71E-02	1.00E+00	1.71E-02	1.00E+00	1.00E+00	0	11	41	21447	0.00%	0.19%	0.00%	0.05%	shared jan 31	
					1.23E-01	2.84E-01	3	19	1400	21447	15.79%	6.53%	0.21%	0.09%	gluconeogenesis Jan 31	Got2, Slc37a2, Slc37a4
					1.73E-02	7.91E-02	3	9	1400	21447	33.33%	6.53%	0.21%	0.04%	glycolysis Jan 31	Hk1, Pfkm, Pklr
MP:0002224	abnormal spleen size	1.73E-02	1.58E-01	1.09E-01	1.58E-01	3.44E-01	2	11	1400	21447	18.18%	6.53%	0.14%	0.05%	shared jan 31	Gpi1, Tpi1
					1.00E+00	1.00E+00	0	19	42	21447	0.00%	0.20%	0.00%	0.09%	gluconeogenesis Jan 31	
					1.75E-02	7.91E-02	1	9	42	21447	11.11%	0.20%	2.38%	0.04%	glycolysis Jan 31	Hk1
MP:0008807	increased liver iron level	1.75E-02	1.00E+00	1.75E-02	1.00E+00	1.00E+00	0	11	42	21447	0.00%	0.20%	0.00%	0.05%	shared jan 31	
					1.00E+00	1.00E+00	0	19	42	21447	0.00%	0.20%	0.00%	0.09%	gluconeogenesis Jan 31	
					1.75E-02	7.91E-02	1	9	42	21447	11.11%	0.20%	2.38%	0.04%	glycolysis Jan 31	Gck
MP:0001759	increased urine glucose level	1.75E-02	1.00E+00	1.75E-02	1.00E+00	1.00E+00	0	11	42	21447	0.00%	0.20%	0.00%	0.05%	shared jan 31	
					1.00E+00	1.00E+00	0	19	42	21447	0.00%	0.20%	0.00%	0.09%	gluconeogenesis Jan 31	
					1.75E-02	7.91E-02	1	9	42	21447	11.11%	0.20%	2.38%	0.04%	glycolysis Jan 31	Pfkm
MP:0003852	skeletal muscle necrosis	1.75E-02	1.00E+00	1.75E-02	1.00E+00	1.00E+00	0	11	42	21447	0.00%	0.20%	0.00%	0.05%	shared jan 31	
					1.76E-02	1.12E-01	1	19	20	21447	5.26%	0.09%	5.00%	0.09%	gluconeogenesis Jan 31	G6pc
					1.00E+00	1.00E+00	0	9	20	21447	0.00%	0.09%	0.00%	0.04%	glycolysis Jan 31	
MP:0009809	abnormal urine uric acid level	1.76E-02	1.00E+00	1.76E-02	1.00E+00	1.00E+00	0	11	20	21447	0.00%	0.09%	0.00%	0.05%	shared jan 31	
					1.76E-02	1.12E-01	1	19	20	21447	5.26%	0.09%	5.00%	0.09%	gluconeogenesis Jan 31	Slc25a13
					1.00E+00	1.00E+00	0	9	20	21447	0.00%	0.09%	0.00%	0.04%	glycolysis Jan 31	

MP:0000036	absent semicircular canals	1.76E-02	1.00E+00	1.76E-02	1.00E+00	1.00E+00	0	11	20	21447	0.00%	0.09%	0.00%	0.05%	shared jan 31	
					1.76E-02	1.12E-01	1	19	20	21447	5.26%	0.09%	5.00%	0.09%	gluconeogenesis Jan 31	Got1
					1.00E+00	1.00E+00	0	9	20	21447	0.00%	0.09%	0.00%	0.04%	glycolysis Jan 31	
MP:0002607	decreased basophil cell number	1.76E-02	1.00E+00	1.76E-02	1.00E+00	1.00E+00	0	11	20	21447	0.00%	0.09%	0.00%	0.05%	shared jan 31	
					1.76E-02	1.12E-01	1	19	20	21447	5.26%	0.09%	5.00%	0.09%	gluconeogenesis Jan 31	Pck1
					1.00E+00	1.00E+00	0	9	20	21447	0.00%	0.09%	0.00%	0.04%	glycolysis Jan 31	
MP:0009136	decreased brown fat cell size	1.76E-02	1.00E+00	1.76E-02	1.00E+00	1.00E+00	0	11	20	21447	0.00%	0.09%	0.00%	0.05%	shared jan 31	
					1.76E-02	1.12E-01	1	19	20	21447	5.26%	0.09%	5.00%	0.09%	gluconeogenesis Jan 31	Slc25a13
					1.00E+00	1.00E+00	0	9	20	21447	0.00%	0.09%	0.00%	0.04%	glycolysis Jan 31	
MP:0003148	decreased cochlear coiling	1.76E-02	1.00E+00	1.76E-02	1.00E+00	1.00E+00	0	11	20	21447	0.00%	0.09%	0.00%	0.05%	shared jan 31	
					1.78E-02	1.12E-01	2	19	233	21447	10.53%	1.09%	0.86%	0.09%	gluconeogenesis Jan 31	Got2, Mdh2
					1.00E+00	1.00E+00	0	9	233	21447	0.00%	1.09%	0.00%	0.04%	glycolysis Jan 31	
MP:0003229	abnormal vitelline vasculature morphology	1.78E-02	1.00E+00	1.78E-02	1.00E+00	1.00E+00	0	11	233	21447	0.00%	1.09%	0.00%	0.05%	shared jan 31	
					1.00E+00	1.00E+00	0	19	43	21447	0.00%	0.20%	0.00%	0.09%	gluconeogenesis Jan 31	
					1.79E-02	7.92E-02	1	9	43	21447	11.11%	0.20%	2.33%	0.04%	glycolysis Jan 31	Gck
MP:0011818	abnormal pancreatic beta cell proliferation	1.79E-02	1.00E+00	1.79E-02	1.00E+00	1.00E+00	0	11	43	21447	0.00%	0.20%	0.00%	0.05%	shared jan 31	
					1.00E+00	1.00E+00	0	19	43	21447	0.00%	0.20%	0.00%	0.09%	gluconeogenesis Jan 31	
					1.79E-02	7.92E-02	1	9	43	21447	11.11%	0.20%	2.33%	0.04%	glycolysis Jan 31	Gck
MP:0031116	microvesicular hepatic steatosis	1.79E-02	1.00E+00	1.79E-02	1.00E+00	1.00E+00	0	11	43	21447	0.00%	0.20%	0.00%	0.05%	shared jan 31	
					1.00E+00	1.00E+00	0	19	510	21447	0.00%	2.38%	0.00%	0.09%	gluconeogenesis Jan 31	
					1.82E-02	7.96E-02	2	9	510	21447	22.22%	2.38%	0.39%	0.04%	glycolysis Jan 31	Hk1, Pklr
MP:0001586	abnormal erythrocyte cell number	1.82E-02	1.00E+00	1.82E-02	2.69E-02	1.23E-01	2	11	510	21447	18.18%	2.38%	0.39%	0.05%	shared jan 31	Gpi1, Tpi1
					1.82E-02	1.13E-01	8	19	4113	21447	42.11%	19.18%	0.19%	0.09%	gluconeogenesis Jan 31	Fbp2, G6pc2, G6pc3, Got1, Got2, Pck2, Slc37a2, Slc37a4
					7.51E-02	1.53E-01	4	9	4113	21447	44.44%	19.18%	0.10%	0.04%	glycolysis Jan 31	Hk1, Hk3, Pfkm, Pklr
MP:0000685	abnormal immune system morphology	1.82E-02	1.43E-01	1.27E-01	1.43E-01	3.26E-01	4	11	4113	21447	36.36%	19.18%	0.10%	0.05%	shared jan 31	Aldoa, Gpi1, Pgam1, Tpi1
					1.84E-02	1.13E-01	1	19	21	21447	5.26%	0.10%	4.76%	0.09%	gluconeogenesis Jan 31	Pck1
					1.00E+00	1.00E+00	0	9	21	21447	0.00%	0.10%	0.00%	0.04%	glycolysis Jan 31	
MP:0003458	decreased circulating ketone body level	1.84E-02	1.00E+00	1.84E-02	1.00E+00	1.00E+00	0	11	21	21447	0.00%	0.10%	0.00%	0.05%	shared jan 31	
					1.84E-02	1.13E-01	1	19	21	21447	5.26%	0.10%	4.76%	0.09%	gluconeogenesis Jan 31	G6pc
					1.00E+00	1.00E+00	0	9	21	21447	0.00%	0.10%	0.00%	0.04%	glycolysis Jan 31	
MP:0008821	increased blood uric acid level	1.84E-02	1.00E+00	1.84E-02	1.00E+00	1.00E+00	0	11	21	21447	0.00%	0.10%	0.00%	0.05%	shared jan 31	
					1.00E+00	1.00E+00	0	19	45	21447	0.00%	0.21%	0.00%	0.09%	gluconeogenesis Jan 31	
					1.87E-02	8.03E-02	1	9	45	21447	11.11%	0.21%	2.22%	0.04%	glycolysis Jan 31	Pklr
MP:0011177	abnormal erythroblast number	1.87E-02	1.00E+00	1.87E-02	1.00E+00	1.00E+00	0	11	45	21447	0.00%	0.21%	0.00%	0.05%	shared jan 31	
					1.00E+00	1.00E+00	0	19	45	21447	0.00%	0.21%	0.00%	0.09%	gluconeogenesis Jan 31	
					1.87E-02	8.03E-02	1	9	45	21447	11.11%	0.21%	2.22%	0.04%	glycolysis Jan 31	Pkm
MP:0008527	embryonic lethality at implantation	1.87E-02	1.00E+00	1.87E-02	1.00E+00	1.00E+00	0	11	45	21447	0.00%	0.21%	0.00%	0.05%	shared jan 31	
					1.00E+00	1.00E+00	0	19	37	21447	0.00%	0.17%	0.00%	0.09%	gluconeogenesis Jan 31	
					1.00E+00	1.00E+00	0	9	37	21447	0.00%	0.17%	0.00%	0.04%	glycolysis Jan 31	
MP:0005576	decreased pulmonary ventilation	1.88E-02	1.00E+00	1.88E-02	1.88E-02	1.11E-01	1	11	37	21447	9.09%	0.17%	2.70%	0.05%	shared jan 31	Aldoc
					1.90E-02	1.13E-01	2	19	241	21447	10.53%	1.12%	0.83%	0.09%	gluconeogenesis Jan 31	Got2, Mdh2
					1.00E+00	1.00E+00	0	9	241	21447	0.00%	1.12%	0.00%	0.04%	glycolysis Jan 31	
MP:0001700	abnormal embryo turning	1.90E-02	1.00E+00	1.90E-02	1.00E+00	1.00E+00	0	11	241	21447	0.00%	1.12%	0.00%	0.05%	shared jan 31	
					1.93E-02	1.13E-01	2	19	243	21447	10.53%	1.13%	0.82%	0.09%	gluconeogenesis Jan 31	G6pc, Slc37a4
					1.00E+00	1.00E+00	0	9	243	21447	0.00%	1.13%	0.00%	0.04%	glycolysis Jan 31	
MP:0006395	abnormal epiphyseal plate morphology	1.93E-02	1.00E+00	1.93E-02	1.00E+00	1.00E+00	0	11	243	21447	0.00%	1.13%	0.00%	0.05%	shared jan 31	
					1.93E-02	1.13E-01	1	19	22	21447	5.26%	0.10%	4.55%	0.09%	gluconeogenesis Jan 31	Slc25a12
					1.00E+00	1.00E+00	0	9	22	21447	0.00%	0.10%	0.00%	0.04%	glycolysis Jan 31	
MP:0010149	abnormal synaptic dopamine release	1.93E-02	1.00E+00	1.93E-02	1.00E+00	1.00E+00	0	11	22	21447	0.00%	0.10%	0.00%	0.05%	shared jan 31	
					1.93E-02	1.13E-01	1	19	22	21447	5.26%	0.10%	4.55%	0.09%	gluconeogenesis Jan 31	G6pc
					1.00E+00	1.00E+00	0	9	22	21447	0.00%	0.10%	0.00%	0.04%	glycolysis Jan 31	
MP:0002900	abnormal urine phosphate level	1.93E-02	1.00E+00	1.93E-02	1.00E+00	1.00E+00	0	11	22	21447	0.00%	0.10%	0.00%	0.05%	shared jan 31	
					1.00E+00	1.00E+00	0	19	38	21447	0.00%	0.18%	0.00%	0.09%	gluconeogenesis Jan 31	
					1.00E+00	1.00E+00	0	9	38	21447	0.00%	0.18%	0.00%	0.04%	glycolysis Jan 31	
MP:0010217	abnormal T-helper 17 cell morphology	1.93E-02	1.00E+00	1.93E-02	1.93E-02	1.11E-01	1	11	38	21447	9.09%	0.18%	2.63%	0.05%	shared jan 31	Pgam1
					1.00E+00	1.00E+00	0	19	47	21447	0.00%	0.22%	0.00%	0.09%	gluconeogenesis Jan 31	
					1.96E-02	8.22E-02	1	9	47	21447	11.11%	0.22%	2.13%	0.04%	glycolysis Jan 31	Gck
MP:0001758	abnormal urine glucose level	1.96E-02	1.00E+00	1.96E-02	1.00E+00	1.00E+00	0	11	47	21447	0.00%	0.22%	0.00%	0.05%	shared jan 31	
					1.00E+00	1.00E+00	0	19	538	21447	0.00%	2.51%	0.00%	0.09%	gluconeogenesis Jan 31	
					2.01E-02	8.37E-02	2	9	538	21447	22.22%	2.51%	0.37%	0.04%	glycolysis Jan 31	Hkdc1, Pkm
MP:0006069	abnormal retina neuronal layer morphology	2.01E-02	1.00E+00	2.01E-02	1.00E+00	1.00E+00	0	11	538	21447	0.00%	2.51%	0.00%	0.05%	shared jan 31	
					2.02E-02	1.17E-01	1	19	23	21447	5.26%	0.11%	4.35%	0.09%	gluconeogenesis Jan 31	Slc25a13
					1.00E+00	1.00E+00	0	9	23	21447	0.00%	0.11%	0.00%	0.04%	glycolysis Jan 31	
MP:0002858	abnormal posterior semicircular canal morphology	2.02E-02	1.00E+00	2.02E-02	1.00E+00	1.00E+00	0	11	23	21447	0.00%	0.11%	0.00%	0.05%	shared jan 31	
					2.03E-02	1.17E-01	2	19	250	21447	10.53%	1.17%	0.80%	0.09%	gluconeogenesis Jan 31	Got2, Mdh2
					1.00E+00	1.00E+00	0	9	250	21447	0.00%	1.17%	0.00%	0.04%	glycolysis Jan 31	
MP:0003232	abnormal forebrain development	2.03E-02	1.00E+00	2.03E-02	1.00E+00	1.00E+00	0	11	250	21447	0.00%	1.17%	0.00%	0.05%	shared jan 31	
					1.00E+00	1.00E+00	0	19	40	21447	0.00%	0.19%	0.00%	0.09%	gluconeogenesis Jan 31	
					1.00E+00	1.00E+00	0	9	40	21447	0.00%	0.19%	0.00%	0.04%	glycolysis Jan 31	

MP:0013764	decreased T-helper cell number	2.03E-02	1.00E+00	2.03E-02	2.03E-02	1.11E-01	1	11	40	21447	9.09%	0.19%	2.50%	0.05%	shared jan 31	Pgam1
					1.00E+00	1.00E+00	0	19	49	21447	0.00%	0.23%	0.00%	0.09%	gluconeogenesis Jan 31	
					2.04E-02	8.39E-02	1	9	49	21447	11.11%	0.23%	2.04%	0.04%	glycolysis Jan 31	Pfkm
MP:0009415	skeletal muscle degeneration	2.04E-02	1.00E+00	2.04E-02	1.00E+00	1.00E+00	0	11	49	21447	0.00%	0.23%	0.00%	0.05%	shared jan 31	
					1.00E+00	1.00E+00	0	19	50	21447	0.00%	0.23%	0.00%	0.09%	gluconeogenesis Jan 31	
					2.08E-02	8.48E-02	1	9	50	21447	11.11%	0.23%	2.00%	0.04%	glycolysis Jan 31	Pfkm
MP:0013237	abnormal skeletal muscle regeneration	2.08E-02	1.00E+00	2.08E-02	1.00E+00	1.00E+00	0	11	50	21447	0.00%	0.23%	0.00%	0.05%	shared jan 31	
					1.00E+00	1.00E+00	0	19	41	21447	0.00%	0.19%	0.00%	0.09%	gluconeogenesis Jan 31	
					1.00E+00	1.00E+00	0	9	41	21447	0.00%	0.19%	0.00%	0.04%	glycolysis Jan 31	
MP:0004929	decreased epididymis weight	2.08E-02	1.00E+00	2.08E-02	2.08E-02	1.11E-01	1	11	41	21447	9.09%	0.19%	2.44%	0.05%	shared jan 31	Eno4
					2.11E-02	1.20E-01	1	19	24	21447	5.26%	0.11%	4.17%	0.09%	gluconeogenesis Jan 31	Slc37a4
					1.00E+00	1.00E+00	0	9	24	21447	0.00%	0.11%	0.00%	0.04%	glycolysis Jan 31	
MP:0001548	hyperlipidemia	2.11E-02	1.00E+00	2.11E-02	1.00E+00	1.00E+00	0	11	24	21447	0.00%	0.11%	0.00%	0.05%	shared jan 31	
					1.00E+00	1.00E+00	0	19	51	21447	0.00%	0.24%	0.00%	0.09%	gluconeogenesis Jan 31	
					2.12E-02	8.56E-02	1	9	51	21447	11.11%	0.24%	1.96%	0.04%	glycolysis Jan 31	Hk3
MP:0002768	small adrenal glands	2.12E-02	1.00E+00	2.12E-02	1.00E+00	1.00E+00	0	11	51	21447	0.00%	0.24%	0.00%	0.05%	shared jan 31	
					2.19E-02	1.22E-01	1	19	25	21447	5.26%	0.12%	4.00%	0.09%	gluconeogenesis Jan 31	Pck1
					1.00E+00	1.00E+00	0	9	25	21447	0.00%	0.12%	0.00%	0.04%	glycolysis Jan 31	
MP:0009135	abnormal brown fat cell size	2.19E-02	1.00E+00	2.19E-02	1.00E+00	1.00E+00	0	11	25	21447	0.00%	0.12%	0.00%	0.05%	shared jan 31	
					1.00E+00	1.00E+00	0	19	53	21447	0.00%	0.25%	0.00%	0.09%	gluconeogenesis Jan 31	
					2.20E-02	8.81E-02	1	9	53	21447	11.11%	0.25%	1.89%	0.04%	glycolysis Jan 31	Hk1
MP:0008738	abnormal liver iron level	2.20E-02	1.00E+00	2.20E-02	1.00E+00	1.00E+00	0	11	53	21447	0.00%	0.25%	0.00%	0.05%	shared jan 31	
					2.22E-02	1.22E-01	2	19	262	21447	10.53%	1.22%	0.76%	0.09%	gluconeogenesis Jan 31	Pck1, Pcx
					1.05E-01	1.87E-01	1	9	262	21447	11.11%	1.22%	0.38%	0.04%	glycolysis Jan 31	Gck
MP:0001553	abnormal circulating free fatty acids level	2.22E-02	1.00E+00	2.22E-02	1.00E+00	1.00E+00	0	11	262	21447	0.00%	1.22%	0.00%	0.05%	shared jan 31	
					2.23E-02	1.22E-01	2	19	263	21447	10.53%	1.23%	0.76%	0.09%	gluconeogenesis Jan 31	G6pc3, Slc37a4
					1.00E+00	1.00E+00	0	9	263	21447	0.00%	1.23%	0.00%	0.04%	glycolysis Jan 31	
MP:0002462	abnormal granulocyte physiology	2.23E-02	1.00E+00	2.23E-02	1.00E+00	1.00E+00	0	11	263	21447	0.00%	1.23%	0.00%	0.05%	shared jan 31	
					1.00E+00	1.00E+00	0	19	54	21447	0.00%	0.25%	0.00%	0.09%	gluconeogenesis Jan 31	
					2.24E-02	8.88E-02	1	9	54	21447	11.11%	0.25%	1.85%	0.04%	glycolysis Jan 31	Pfkm
MP:0011084	lethality at weaning, incomplete penetrance	2.24E-02	1.00E+00	2.24E-02	1.00E+00	1.00E+00	0	11	54	21447	0.00%	0.25%	0.00%	0.05%	shared jan 31	
					2.28E-02	1.24E-01	1	19	26	21447	5.26%	0.12%	3.85%	0.09%	gluconeogenesis Jan 31	G6pc3
					1.00E+00	1.00E+00	0	9	26	21447	0.00%	0.12%	0.00%	0.04%	glycolysis Jan 31	
MP:0002712	increased circulating glucagon level	2.28E-02	1.00E+00	2.28E-02	1.00E+00	1.00E+00	0	11	26	21447	0.00%	0.12%	0.00%	0.05%	shared jan 31	
					1.00E+00	1.00E+00	0	19	55	21447	0.00%	0.26%	0.00%	0.09%	gluconeogenesis Jan 31	
					2.28E-02	8.88E-02	1	9	55	21447	11.11%	0.26%	1.82%	0.04%	glycolysis Jan 31	Pklr
MP:0011176	abnormal erythroblast morphology	2.28E-02	1.00E+00	2.28E-02	1.00E+00	1.00E+00	0	11	55	21447	0.00%	0.26%	0.00%	0.05%	shared jan 31	
					1.00E+00	1.00E+00	0	19	55	21447	0.00%	0.26%	0.00%	0.09%	gluconeogenesis Jan 31	
					2.28E-02	8.88E-02	1	9	55	21447	11.11%	0.26%	1.82%	0.04%	glycolysis Jan 31	Gck
MP:0009172	small pancreatic islets	2.28E-02	1.00E+00	2.28E-02	1.00E+00	1.00E+00	0	11	55	21447	0.00%	0.26%	0.00%	0.05%	shared jan 31	
					2.31E-02	1.25E-01	3	19	705	21447	15.79%	3.29%	0.43%	0.09%	gluconeogenesis Jan 31	G6pc, Pck1, Slc37a4
					2.60E-01	3.26E-01	1	9	705	21447	11.11%	3.29%	0.14%	0.04%	glycolysis Jan 31	Pfkm
MP:0004848	abnormal liver size	2.31E-02	3.08E-01	7.52E-02	3.08E-01	4.66E-01	1	11	705	21447	9.09%	3.29%	0.14%	0.05%	shared jan 31	Gpi1
					2.37E-02	1.25E-01	1	19	27	21447	5.26%	0.13%	3.70%	0.09%	gluconeogenesis Jan 31	Pck1
					1.00E+00	1.00E+00	0	9	27	21447	0.00%	0.13%	0.00%	0.04%	glycolysis Jan 31	
MP:0030604	abnormal ammonia homeostasis	2.37E-02	1.00E+00	2.37E-02	1.00E+00	1.00E+00	0	11	27	21447	0.00%	0.13%	0.00%	0.05%	shared jan 31	
					2.37E-02	1.25E-01	1	19	27	21447	5.26%	0.13%	3.70%	0.09%	gluconeogenesis Jan 31	G6pc3
					1.00E+00	1.00E+00	0	9	27	21447	0.00%	0.13%	0.00%	0.04%	glycolysis Jan 31	
MP:0009176	increased pancreatic alpha cell number	2.37E-02	1.00E+00	2.37E-02	1.00E+00	1.00E+00	0	11	27	21447	0.00%	0.13%	0.00%	0.05%	shared jan 31	
					1.00E+00	1.00E+00	0	19	57	21447	0.00%	0.27%	0.00%	0.09%	gluconeogenesis Jan 31	
					2.37E-02	9.12E-02	1	9	57	21447	11.11%	0.27%	1.75%	0.04%	glycolysis Jan 31	Hkdc1
MP:0008511	decreased retina inner nuclear layer thickness	2.37E-02	1.00E+00	2.37E-02	1.00E+00	1.00E+00	0	11	57	21447	0.00%	0.27%	0.00%	0.05%	shared jan 31	
					1.00E+00	1.00E+00	0	19	47	21447	0.00%	0.22%	0.00%	0.09%	gluconeogenesis Jan 31	
					1.00E+00	1.00E+00	0	9	47	21447	0.00%	0.22%	0.00%	0.04%	glycolysis Jan 31	
MP:0009836	abnormal sperm principal piece morphology	2.38E-02	1.00E+00	2.38E-02	2.38E-02	1.21E-01	1	11	47	21447	9.09%	0.22%	2.13%	0.05%	shared jan 31	Eno4
					2.44E-02	1.26E-01	3	19	719	21447	15.79%	3.35%	0.42%	0.09%	gluconeogenesis Jan 31	G6pc3, Slc37a2, Slc37a4
					1.00E+00	1.00E+00	0	9	719	21447	0.00%	3.35%	0.00%	0.04%	glycolysis Jan 31	
MP:0002419	abnormal innate immunity	2.44E-02	1.00E+00	2.44E-02	1.00E+00	1.00E+00	0	11	719	21447	0.00%	3.35%	0.00%	0.05%	shared jan 31	
					1.00E+00	1.00E+00	0	19	484	21447	0.00%	2.26%	0.00%	0.09%	gluconeogenesis Jan 31	
					1.86E-01	2.65E-01	1	9	484	21447	11.11%	2.26%	0.21%	0.04%	glycolysis Jan 31	Hk1
MP:0001589	abnormal mean corpuscular hemoglobin	2.44E-02	1.00E+00	2.44E-02	2.44E-02	1.21E-01	2	11	484	21447	18.18%	2.26%	0.41%	0.05%	shared jan 31	Gpi1, Tpi1
					1.00E+00	1.00E+00	0	19	59	21447	0.00%	0.28%	0.00%	0.09%	gluconeogenesis Jan 31	
					2.45E-02	9.26E-02	1	9	59	21447	11.11%	0.28%	1.69%	0.04%	glycolysis Jan 31	Pklr
MP:0003135	increased erythroid progenitor cell number	2.45E-02	1.00E+00	2.45E-02	1.00E+00	1.00E+00	0	11	59	21447	0.00%	0.28%	0.00%	0.05%	shared jan 31	
					1.00E+00	1.00E+00	0	19	59	21447	0.00%	0.28%	0.00%	0.09%	gluconeogenesis Jan 31	
					2.45E-02	9.26E-02	1	9	59	21447	11.11%	0.28%	1.69%	0.04%	glycolysis Jan 31	Pklr
MP:0009395	increased nucleated erythrocyte cell number	2.45E-02	1.00E+00	2.45E-02	1.00E+00	1.00E+00	0	11	59	21447	0.00%	0.28%	0.00%	0.05%	shared jan 31	
					2.45E-02	1.26E-01	1	19	28	21447	5.26%	0.13%	3.57%	0.09%	gluconeogenesis Jan 31	Slc25a13
					1.00E+00	1.00E+00	0	9	28	21447	0.00%	0.13%	0.00%	0.04%	glycolysis Jan 31	
MP:0000037	abnormal lateral semicircular canal morphology	2.45E-02	1.00E+00	2.45E-02	1.00E+00	1.00E+00	0	11	28	21447	0.00%	0.13%	0.00%	0.05%	shared jan 31	

					2.45E-02	1.26E-01	1	19	28	21447	5.26%	0.13%	3.57%	0.09%	gluconeogenesis Jan 31	Pck1
					1.00E+00	1.00E+00	0	9	28	21447	0.00%	0.13%	0.00%	0.04%	glycolysis Jan 31	
MP:0008853	decreased abdominal adipose tissue amount	2.45E-02	1.00E+00	2.45E-02	1.00E+00	1.00E+00	0	11	28	21447	0.00%	0.13%	0.00%	0.05%	shared jan 31	
					1.00E+00	1.00E+00	0	19	49	21447	0.00%	0.23%	0.00%	0.09%	gluconeogenesis Jan 31	
					1.00E+00	1.00E+00	0	9	49	21447	0.00%	0.23%	0.00%	0.04%	glycolysis Jan 31	
MP:0008080	abnormal CD8-positive, alpha-beta T cell differentiation	2.49E-02	1.00E+00	2.49E-02	2.49E-02	1.21E-01	1	11	49	21447	9.09%	0.23%	2.04%	0.05%	shared jan 31	Pgam1
					2.51E-02	1.28E-01	3	19	727	21447	15.79%	3.39%	0.41%	0.09%	gluconeogenesis Jan 31	G6pc, Pcx, Slc37a4
					1.00E+00	1.00E+00	0	9	727	21447	0.00%	3.39%	0.00%	0.04%	glycolysis Jan 31	
MP:0002706	abnormal kidney size	2.51E-02	1.00E+00	2.51E-02	1.00E+00	1.00E+00	0	11	727	21447	0.00%	3.39%	0.00%	0.05%	shared jan 31	
					1.00E+00	1.00E+00	0	19	493	21447	0.00%	2.30%	0.00%	0.09%	gluconeogenesis Jan 31	
					1.00E+00	1.00E+00	0	9	493	21447	0.00%	2.30%	0.00%	0.04%	glycolysis Jan 31	
MP:0011092	embryonic lethality, complete penetrance	2.53E-02	1.00E+00	2.53E-02	2.53E-02	1.21E-01	2	11	493	21447	18.18%	2.30%	0.41%	0.05%	shared jan 31	Gpi1, Pgam1
					1.00E+00	1.00E+00	0	19	61	21447	0.00%	0.28%	0.00%	0.09%	gluconeogenesis Jan 31	
					2.53E-02	9.37E-02	1	9	61	21447	11.11%	0.28%	1.64%	0.04%	glycolysis Jan 31	Hkdc1
MP:0011996	abnormal retina inner nuclear layer thickness	2.53E-02	1.00E+00	2.53E-02	1.00E+00	1.00E+00	0	11	61	21447	0.00%	0.28%	0.00%	0.05%	shared jan 31	
					1.00E+00	1.00E+00	0	19	50	21447	0.00%	0.23%	0.00%	0.09%	gluconeogenesis Jan 31	
					1.00E+00	1.00E+00	0	9	50	21447	0.00%	0.23%	0.00%	0.04%	glycolysis Jan 31	
MP:0004927	abnormal epididymis weight	2.54E-02	1.00E+00	2.54E-02	2.54E-02	1.21E-01	1	11	50	21447	9.09%	0.23%	2.00%	0.05%	shared jan 31	Eno4
					2.56E-02	1.29E-01	2	19	283	21447	10.53%	1.32%	0.71%	0.09%	gluconeogenesis Jan 31	Pck1, Pcx
					1.13E-01	1.91E-01	1	9	283	21447	11.11%	1.32%	0.35%	0.04%	glycolysis Jan 31	Gck
MP:0010358	abnormal free fatty acids level	2.56E-02	1.00E+00	2.56E-02	1.00E+00	1.00E+00	0	11	283	21447	0.00%	1.32%	0.00%	0.05%	shared jan 31	
					1.00E+00	1.00E+00	0	19	613	21447	0.00%	2.86%	0.00%	0.09%	gluconeogenesis Jan 31	
					2.57E-02	9.37E-02	2	9	613	21447	22.22%	2.86%	0.33%	0.04%	glycolysis Jan 31	Hkdc1, Pkm
MP:0003727	abnormal retina layer morphology	2.57E-02	1.00E+00	2.57E-02	1.00E+00	1.00E+00	0	11	613	21447	0.00%	2.86%	0.00%	0.05%	shared jan 31	
					1.00E+00	1.00E+00	0	19	62	21447	0.00%	0.29%	0.00%	0.09%	gluconeogenesis Jan 31	
					2.57E-02	9.37E-02	1	9	62	21447	11.11%	0.29%	1.61%	0.04%	glycolysis Jan 31	Pklr
MP:0012397	abnormal nucleated erythrocyte cell number	2.57E-02	1.00E+00	2.57E-02	1.00E+00	1.00E+00	0	11	62	21447	0.00%	0.29%	0.00%	0.05%	shared jan 31	
					1.00E+00	1.00E+00	0	19	618	21447	0.00%	2.88%	0.00%	0.09%	gluconeogenesis Jan 31	
					2.61E-02	9.37E-02	2	9	618	21447	22.22%	2.88%	0.32%	0.04%	glycolysis Jan 31	Hk1, Pklr
MP:0013662	decreased myeloid cell number	2.61E-02	1.00E+00	2.61E-02	3.84E-02	1.51E-01	2	11	618	21447	18.18%	2.88%	0.32%	0.05%	shared jan 31	Gpi1, Tpi1
					1.00E+00	1.00E+00	0	19	63	21447	0.00%	0.29%	0.00%	0.09%	gluconeogenesis Jan 31	
					2.61E-02	9.37E-02	1	9	63	21447	11.11%	0.29%	1.59%	0.04%	glycolysis Jan 31	Hkdc1
MP:0003926	decreased cellular glucose uptake	2.61E-02	1.00E+00	2.61E-02	1.00E+00	1.00E+00	0	11	63	21447	0.00%	0.29%	0.00%	0.05%	shared jan 31	
					1.00E+00	1.00E+00	0	19	63	21447	0.00%	0.29%	0.00%	0.09%	gluconeogenesis Jan 31	
					2.61E-02	9.37E-02	1	9	63	21447	11.11%	0.29%	1.59%	0.04%	glycolysis Jan 31	Gck
MP:0009114	decreased pancreatic beta cell mass	2.61E-02	1.00E+00	2.61E-02	1.00E+00	1.00E+00	0	11	63	21447	0.00%	0.29%	0.00%	0.05%	shared jan 31	
					2.63E-02	1.32E-01	1	19	30	21447	5.26%	0.14%	3.33%	0.09%	gluconeogenesis Jan 31	Slc25a12
					1.00E+00	1.00E+00	0	9	30	21447	0.00%	0.14%	0.00%	0.04%	glycolysis Jan 31	
MP:0012468	decreased striatum size	2.63E-02	1.00E+00	2.63E-02	1.00E+00	1.00E+00	0	11	30	21447	0.00%	0.14%	0.00%	0.05%	shared jan 31	
					1.00E+00	1.00E+00	0	19	52	21447	0.00%	0.24%	0.00%	0.09%	gluconeogenesis Jan 31	
					1.00E+00	1.00E+00	0	9	52	21447	0.00%	0.24%	0.00%	0.04%	glycolysis Jan 31	
MP:0002338	abnormal pulmonary ventilation	2.64E-02	1.00E+00	2.64E-02	2.64E-02	1.23E-01	1	11	52	21447	9.09%	0.24%	1.92%	0.05%	shared jan 31	Aldoc
					1.00E+00	1.00E+00	0	19	64	21447	0.00%	0.30%	0.00%	0.09%	gluconeogenesis Jan 31	
					2.65E-02	9.44E-02	1	9	64	21447	11.11%	0.30%	1.56%	0.04%	glycolysis Jan 31	Hkdc1
MP:0008518	retina outer nuclear layer degeneration	2.65E-02	1.00E+00	2.65E-02	1.00E+00	1.00E+00	0	11	64	21447	0.00%	0.30%	0.00%	0.05%	shared jan 31	
					1.00E+00	1.00E+00	0	19	1326	21447	0.00%	6.18%	0.00%	0.09%	gluconeogenesis Jan 31	
					4.37E-01	4.87E-01	1	9	1326	21447	11.11%	6.18%	0.08%	0.04%	glycolysis Jan 31	Hk1
MP:0001924	infertility	2.68E-02	1.00E+00	2.68E-02	2.68E-02	1.23E-01	3	11	1326	21447	27.27%	6.18%	0.23%	0.05%	shared jan 31	Eno4, Gapdhs, Pgk2
					1.00E+00	1.00E+00	0	19	65	21447	0.00%	0.30%	0.00%	0.09%	gluconeogenesis Jan 31	
					2.70E-02	9.49E-02	1	9	65	21447	11.11%	0.30%	1.54%	0.04%	glycolysis Jan 31	Pfkm
MP:0014068	abnormal muscle glycogen level	2.70E-02	1.00E+00	2.70E-02	1.00E+00	1.00E+00	0	11	65	21447	0.00%	0.30%	0.00%	0.05%	shared jan 31	
					5.12E-01	5.83E-01	3	19	3016	21447	15.79%	14.06%	0.10%	0.09%	gluconeogenesis Jan 31	G6pc3, Got1, Slc37a4
					2.73E-02	9.49E-02	4	9	3016	21447	44.44%	14.06%	0.13%	0.04%	glycolysis Jan 31	Gck, Hk1, Pfkm, Pklr
MP:0013658	abnormal myeloid cell morphology	2.73E-02	5.12E-01	5.33E-02	1.93E-01	3.77E-01	3	11	3016	21447	27.27%	14.06%	0.10%	0.05%	shared jan 31	Gapdh, Gpi1, Tpi1
					4.35E-01	5.22E-01	1	19	634	21447	5.26%	2.96%	0.16%	0.09%	gluconeogenesis Jan 31	Slc25a13
					2.74E-02	9.49E-02	2	9	634	21447	22.22%	2.96%	0.32%	0.04%	glycolysis Jan 31	Gck, Pfkm
MP:0001560	abnormal circulating insulin level	2.74E-02	1.00E+00	2.74E-02	1.00E+00	1.00E+00	0	11	634	21447	0.00%	2.96%	0.00%	0.05%	shared jan 31	
					1.00E+00	1.00E+00	0	19	67	21447	0.00%	0.31%	0.00%	0.09%	gluconeogenesis Jan 31	
					2.78E-02	9.55E-02	1	9	67	21447	11.11%	0.31%	1.49%	0.04%	glycolysis Jan 31	Pfkm
MP:0002943	abnormal lactate dehydrogenase level	2.78E-02	1.00E+00	2.78E-02	1.00E+00	1.00E+00	0	11	67	21447	0.00%	0.31%	0.00%	0.05%	shared jan 31	
					1.00E+00	1.00E+00	0	19	55	21447	0.00%	0.26%	0.00%	0.09%	gluconeogenesis Jan 31	
					1.00E+00	1.00E+00	0	9	55	21447	0.00%	0.26%	0.00%	0.04%	glycolysis Jan 31	
MP:0002434	abnormal T-helper 2 cell morphology	2.79E-02	1.00E+00	2.79E-02	2.79E-02	1.24E-01	1	11	55	21447	9.09%	0.26%	1.82%	0.05%	shared jan 31	Pgam1
					2.80E-02	1.38E-01	1	19	32	21447	5.26%	0.15%	3.12%	0.09%	gluconeogenesis Jan 31	G6pc
					1.00E+00	1.00E+00	0	9	32	21447	0.00%	0.15%	0.00%	0.04%	glycolysis Jan 31	
MP:0008820	abnormal blood uric acid level	2.80E-02	1.00E+00	2.80E-02	1.00E+00	1.00E+00	0	11	32	21447	0.00%	0.15%	0.00%	0.05%	shared jan 31	
					2.80E-02	1.38E-01	1	19	32	21447	5.26%	0.15%	3.12%	0.09%	gluconeogenesis Jan 31	G6pc
					1.00E+00	1.00E+00	0	9	32	21447	0.00%	0.15%	0.00%	0.04%	glycolysis Jan 31	
MP:0009348	abnormal urine pH	2.80E-02	1.00E+00	2.80E-02	1.00E+00	1.00E+00	0	11	32	21447	0.00%	0.15%	0.00%	0.05%	shared jan 31	
					1.00E+00	1.00E+00	0	19	56	21447	0.00%	0.26%	0.00%	0.09%	gluconeogenesis Jan 31	

									1.00E+00	1.00E+00	0	9	56	21447	0.00%	0.26%	0.00%	0.04%	glycolysis Jan 31	
				MP:0005573	increased pulmonary respiratory rate	2.84E-02	1.00E+00	2.84E-02	2.84E-02	1.24E-01	1	11	56	21447	9.09%	0.26%	1.79%	0.05%	shared jan 31	Aldoc
									1.00E+00	1.00E+00	0	19	69	21447	0.00%	0.32%	0.00%	0.09%	gluconeogenesis Jan 31	
									2.86E-02	9.75E-02	1	9	69	21447	11.11%	0.32%	1.45%	0.04%	glycolysis Jan 31	Gck
				MP:0003896	prolonged PR interval	2.86E-02	1.00E+00	2.86E-02	1.00E+00	1.00E+00	0	11	69	21447	0.00%	0.32%	0.00%	0.05%	shared jan 31	
									2.88E-02	1.41E-01	1	19	33	21447	5.26%	0.15%	3.03%	0.09%	gluconeogenesis Jan 31	Slc25a12
									1.00E+00	1.00E+00	0	9	33	21447	0.00%	0.15%	0.00%	0.04%	glycolysis Jan 31	
				MP:0003245	abnormal GABAergic neuron morphology	2.88E-02	1.00E+00	2.88E-02	1.00E+00	1.00E+00	0	11	33	21447	0.00%	0.15%	0.00%	0.05%	shared jan 31	
									4.45E-01	5.27E-01	1	19	654	21447	5.26%	3.05%	0.15%	0.09%	gluconeogenesis Jan 31	Pck1
									2.90E-02	9.77E-02	2	9	654	21447	22.22%	3.05%	0.31%	0.04%	glycolysis Jan 31	Gck, Pfkm
				MP:0005559	increased circulating glucose level	2.90E-02	4.45E-01	6.52E-02	2.89E-01	4.57E-01	1	11	654	21447	9.09%	3.05%	0.15%	0.05%	shared jan 31	Gpi1
									6.11E-02	2.19E-01	1	19	71	21447	5.26%	0.33%	1.41%	0.09%	gluconeogenesis Jan 31	Pck1
									2.94E-02	9.77E-02	1	9	71	21447	11.11%	0.33%	1.41%	0.04%	glycolysis Jan 31	Gck
				MP:0010398	decreased liver glycogen level	2.94E-02	1.00E+00	2.94E-02	1.00E+00	1.00E+00	0	11	71	21447	0.00%	0.33%	0.00%	0.05%	shared jan 31	
									1.00E+00	1.00E+00	0	19	71	21447	0.00%	0.33%	0.00%	0.09%	gluconeogenesis Jan 31	
									2.94E-02	9.77E-02	1	9	71	21447	11.11%	0.33%	1.41%	0.04%	glycolysis Jan 31	Hk1
				MP:0008476	increased spleen red pulp amount	2.94E-02	1.00E+00	2.94E-02	1.00E+00	1.00E+00	0	11	71	21447	0.00%	0.33%	0.00%	0.05%	shared jan 31	
									2.97E-02	1.43E-01	1	19	34	21447	5.26%	0.16%	2.94%	0.09%	gluconeogenesis Jan 31	G6pc
									1.00E+00	1.00E+00	0	9	34	21447	0.00%	0.16%	0.00%	0.04%	glycolysis Jan 31	
				MP:0011434	abnormal urine magnesium level	2.97E-02	1.00E+00	2.97E-02	1.00E+00	1.00E+00	0	11	34	21447	0.00%	0.16%	0.00%	0.05%	shared jan 31	
									2.97E-02	1.43E-01	1	19	34	21447	5.26%	0.16%	2.94%	0.09%	gluconeogenesis Jan 31	Slc25a13
									1.00E+00	1.00E+00	0	9	34	21447	0.00%	0.16%	0.00%	0.04%	glycolysis Jan 31	
				MP:0002730	head shaking	2.97E-02	1.00E+00	2.97E-02	1.00E+00	1.00E+00	0	11	34	21447	0.00%	0.16%	0.00%	0.05%	shared jan 31	
									1.00E+00	1.00E+00	0	19	72	21447	0.00%	0.34%	0.00%	0.09%	gluconeogenesis Jan 31	
									2.98E-02	9.77E-02	1	9	72	21447	11.11%	0.34%	1.39%	0.04%	glycolysis Jan 31	Pfkm
				MP:0000750	abnormal muscle regeneration	2.98E-02	1.00E+00	2.98E-02	1.00E+00	1.00E+00	0	11	72	21447	0.00%	0.34%	0.00%	0.05%	shared jan 31	
									1.00E+00	1.00E+00	0	19	72	21447	0.00%	0.34%	0.00%	0.09%	gluconeogenesis Jan 31	
									2.98E-02	9.77E-02	1	9	72	21447	11.11%	0.34%	1.39%	0.04%	glycolysis Jan 31	Gck
				MP:0003058	increased insulin secretion	2.98E-02	1.00E+00	2.98E-02	1.00E+00	1.00E+00	0	11	72	21447	0.00%	0.34%	0.00%	0.05%	shared jan 31	
									3.06E-02	1.46E-01	1	19	35	21447	5.26%	0.16%	2.86%	0.09%	gluconeogenesis Jan 31	Slc37a4
									1.00E+00	1.00E+00	0	9	35	21447	0.00%	0.16%	0.00%	0.04%	glycolysis Jan 31	
				MP:0000065	abnormal bone marrow cavity morphology	3.06E-02	1.00E+00	3.06E-02	1.00E+00	1.00E+00	0	11	35	21447	0.00%	0.16%	0.00%	0.05%	shared jan 31	
									4.62E-01	5.38E-01	2	19	1735	21447	10.53%	8.09%	0.12%	0.09%	gluconeogenesis Jan 31	Pck1, Pck2
									3.06E-02	9.91E-02	3	9	1735	21447	33.33%	8.09%	0.17%	0.04%	glycolysis Jan 31	Hk1, Hk3, Pfkm
				MP:0002060	abnormal skin morphology	3.06E-02	1.00E+00	3.06E-02	1.00E+00	1.00E+00	0	11	1735	21447	0.00%	8.09%	0.00%	0.05%	shared jan 31	
									1.96E-01	3.41E-01	3	19	1737	21447	15.79%	8.10%	0.17%	0.09%	gluconeogenesis Jan 31	Got2, Slc37a2, Slc37a4
									3.07E-02	9.91E-02	3	9	1737	21447	33.33%	8.10%	0.17%	0.04%	glycolysis Jan 31	Hk1, Pfkm, Pklr
				MP:0000689	abnormal spleen morphology	3.07E-02	2.22E-01	1.38E-01	2.22E-01	4.07E-01	2	11	1737	21447	18.18%	8.10%	0.12%	0.05%	shared jan 31	Gpi1, Tpi1
									1.00E+00	1.00E+00	0	19	61	21447	0.00%	0.28%	0.00%	0.09%	gluconeogenesis Jan 31	
									1.00E+00	1.00E+00	0	9	61	21447	0.00%	0.28%	0.00%	0.04%	glycolysis Jan 31	
				MP:0004043	abnormal pH regulation	3.09E-02	1.00E+00	3.09E-02	3.09E-02	1.31E-01	1	11	61	21447	9.09%	0.28%	1.64%	0.05%	shared jan 31	Pgam1
									1.00E+00	1.00E+00	0	19	61	21447	0.00%	0.28%	0.00%	0.09%	gluconeogenesis Jan 31	
									1.00E+00	1.00E+00	0	9	61	21447	0.00%	0.28%	0.00%	0.04%	glycolysis Jan 31	
				MP:0009237	kinked sperm flagellum	3.09E-02	1.00E+00	3.09E-02	3.09E-02	1.31E-01	1	11	61	21447	9.09%	0.28%	1.64%	0.05%	shared jan 31	Eno4
									1.00E+00	1.00E+00	0	19	75	21447	0.00%	0.35%	0.00%	0.09%	gluconeogenesis Jan 31	
									3.10E-02	9.93E-02	1	9	75	21447	11.11%	0.35%	1.33%	0.04%	glycolysis Jan 31	Hk1
				MP:0002642	anisocytosis	3.10E-02	1.00E+00	3.10E-02	1.00E+00	1.00E+00	0	11	75	21447	0.00%	0.35%	0.00%	0.05%	shared jan 31	
									1.00E+00	1.00E+00	0	19	63	21447	0.00%	0.29%	0.00%	0.09%	gluconeogenesis Jan 31	
									1.00E+00	1.00E+00	0	9	63	21447	0.00%	0.29%	0.00%	0.04%	glycolysis Jan 31	
				MP:0005630	increased lung weight	3.18E-02	1.00E+00	3.18E-02	3.18E-02	1.34E-01	1	11	63	21447	9.09%	0.29%	1.59%	0.05%	shared jan 31	Gpi1
									5.46E-01	6.06E-01	3	19	3161	21447	15.79%	14.74%	0.09%	0.09%	gluconeogenesis Jan 31	G6pc3, Got1, Slc37a4
									3.20E-02	1.00E-01	4	9	3161	21447	44.44%	14.74%	0.13%	0.04%	glycolysis Jan 31	Gck, Hk1, Pfkm, Pklr
				MP:0013657	abnormal blood cell morphology	3.20E-02	5.46E-01	5.85E-02	6.57E-02	2.01E-01	4	11	3161	21447	36.36%	14.74%	0.13%	0.05%	shared jan 31	Gapdh, Gpi1, Pgam1, Tpi1
									5.47E-01	6.06E-01	3	19	3165	21447	15.79%	14.76%	0.09%	0.09%	gluconeogenesis Jan 31	Pck1, Pck2, Slc25a13
									3.21E-02	1.00E-01	4	9	3165	21447	44.44%	14.76%	0.13%	0.04%	glycolysis Jan 31	Hk1, Hk3, Pfkl, Pfkm
				MP:0010771	integument phenotype	3.21E-02	1.00E+00	3.21E-02	1.00E+00	1.00E+00	0	11	3165	21447	0.00%	14.76%	0.00%	0.05%	shared jan 31	
									1.00E+00	1.00E+00	0	19	80	21447	0.00%	0.37%	0.00%	0.09%	gluconeogenesis Jan 31	
									3.31E-02	1.02E-01	1	9	80	21447	11.11%	0.37%	1.25%	0.04%	glycolysis Jan 31	Pfkm
				MP:0004809	increased hematopoietic stem cell number	3.31E-02	1.00E+00	3.31E-02	1.00E+00	1.00E+00	0	11	80	21447	0.00%	0.37%	0.00%	0.05%	shared jan 31	
									3.31E-02	1.56E-01	1	19	38	21447	5.26%	0.18%	2.63%	0.09%	gluconeogenesis Jan 31	Slc25a13
									1.00E+00	1.00E+00	0	9	38	21447	0.00%	0.18%	0.00%	0.04%	glycolysis Jan 31	
				MP:0006011	abnormal endolymphatic duct morphology	3.31E-02	1.00E+00	3.31E-02	1.00E+00	1.00E+00	0	11	38	21447	0.00%	0.18%	0.00%	0.05%	shared jan 31	
									3.31E-02	1.56E-01	1	19	38	21447	5.26%	0.18%	2.63%	0.09%	gluconeogenesis Jan 31	Slc37a4
									1.00E+00	1.00E+00	0	9	38	21447	0.00%	0.18%	0.00%	0.04%	glycolysis Jan 31	
				MP:0000131	abnormal long bone epiphysis morphology	3.31E-02	1.00E+00	3.31E-02	1.00E+00	1.00E+00	0	11	38	21447	0.00%	0.18%	0.00%	0.05%	shared jan 31	
									1.00E+00	1.00E+00	0	19	1443	21447	0.00%	6.73%	0.00%	0.09%	gluconeogenesis Jan 31	
									1.00E+00	1.00E+00	0	9	1443	21447	0.00%	6.73%	0.00%	0.04%	glycolysis Jan 31	
				MP:0003698	abnormal male reproductive system physiology	3.34E-02	1.00E+00	3.34E-02	3.34E-02	1.38E-01	3	11	1443	21447	27.27%	6.73%	0.21%	0.05%	shared jan 31	Eno4, Gapdhs, Pgk2
									1.00E+00	1.00E+00	0	19	81	21447	0.00%	0.38%	0.00%	0.09%	gluconeogenesis Jan 31	
									3.35E-02	1.02E-01	1	9	81	21447	11.11%	0.38%	1.23%	0.04%	glycolysis Jan 31	Hkdc1

MP:0012143	decreased a-wave amplitude	3.35E-02	1.00E+00	3.35E-02	1.00E+00	1.00E+00	0	11	81	21447	0.00%	0.38%	0.00%	0.05%	shared jan 31	
					1.00E+00	1.00E+00	0	19	81	21447	0.00%	0.38%	0.00%	0.09%	gluconeogenesis Jan 31	
					3.35E-02	1.02E-01	1	9	81	21447	11.11%	0.38%	1.23%	0.04%	glycolysis Jan 31	Pklr
MP:0005026	decreased susceptibility to parasitic infection	3.35E-02	1.00E+00	3.35E-02	1.00E+00	1.00E+00	0	11	81	21447	0.00%	0.38%	0.00%	0.05%	shared jan 31	
					3.39E-02	1.58E-01	3	19	817	21447	15.79%	3.81%	0.37%	0.09%	gluconeogenesis Jan 31	G6pc3, Got1, Slc37a4
					1.00E+00	1.00E+00	0	9	817	21447	0.00%	3.81%	0.00%	0.04%	glycolysis Jan 31	
MP:0020201	abnormal granulocyte number	3.39E-02	1.00E+00	3.39E-02	1.00E+00	1.00E+00	0	11	817	21447	0.00%	3.81%	0.00%	0.05%	shared jan 31	
					1.00E+00	1.00E+00	0	19	82	21447	0.00%	0.38%	0.00%	0.09%	gluconeogenesis Jan 31	
					3.39E-02	1.03E-01	1	9	82	21447	11.11%	0.38%	1.22%	0.04%	glycolysis Jan 31	Hk2
MP:0000872	abnormal cerebellum external granule cell layer morphology	3.39E-02	1.00E+00	3.39E-02	1.00E+00	1.00E+00	0	11	82	21447	0.00%	0.38%	0.00%	0.05%	shared jan 31	
					3.41E-02	1.58E-01	3	19	819	21447	15.79%	3.82%	0.37%	0.09%	gluconeogenesis Jan 31	G6pc2, Slc37a2, Slc37a4
					1.00E+00	1.00E+00	0	9	819	21447	0.00%	3.82%	0.00%	0.04%	glycolysis Jan 31	
MP:0003009	abnormal cytokine secretion	3.41E-02	1.00E+00	3.41E-02	3.48E-01	4.88E-01	1	11	819	21447	9.09%	3.82%	0.12%	0.05%	shared jan 31	Pgam1
					1.00E+00	1.00E+00	0	19	83	21447	0.00%	0.39%	0.00%	0.09%	gluconeogenesis Jan 31	
					3.43E-02	1.03E-01	1	9	83	21447	11.11%	0.39%	1.20%	0.04%	glycolysis Jan 31	Hkdc1
MP:0012030	abnormal a-wave amplitude	3.43E-02	1.00E+00	3.43E-02	1.00E+00	1.00E+00	0	11	83	21447	0.00%	0.39%	0.00%	0.05%	shared jan 31	
					1.00E+00	1.00E+00	0	19	83	21447	0.00%	0.39%	0.00%	0.09%	gluconeogenesis Jan 31	
					3.43E-02	1.03E-01	1	9	83	21447	11.11%	0.39%	1.20%	0.04%	glycolysis Jan 31	Gck
MP:0011635	abnormal mitochondrial crista morphology	3.43E-02	1.00E+00	3.43E-02	1.00E+00	1.00E+00	0	11	83	21447	0.00%	0.39%	0.00%	0.05%	shared jan 31	
					1.00E+00	1.00E+00	0	19	84	21447	0.00%	0.39%	0.00%	0.09%	gluconeogenesis Jan 31	
					3.47E-02	1.03E-01	1	9	84	21447	11.11%	0.39%	1.19%	0.04%	glycolysis Jan 31	Hkdc1
MP:0012034	abnormal a-wave shape	3.47E-02	1.00E+00	3.47E-02	1.00E+00	1.00E+00	0	11	84	21447	0.00%	0.39%	0.00%	0.05%	shared jan 31	
					3.49E-02	1.60E-01	1	19	40	21447	5.26%	0.19%	2.50%	0.09%	gluconeogenesis Jan 31	G6pc
					1.00E+00	1.00E+00	0	9	40	21447	0.00%	0.19%	0.00%	0.04%	glycolysis Jan 31	
MP:0003324	increased liver adenoma incidence	3.49E-02	1.00E+00	3.49E-02	1.00E+00	1.00E+00	0	11	40	21447	0.00%	0.19%	0.00%	0.05%	shared jan 31	
					3.51E-02	1.60E-01	2	19	336	21447	10.53%	1.57%	0.60%	0.09%	gluconeogenesis Jan 31	Pcx, Slc25a10
					1.00E+00	1.00E+00	0	9	336	21447	0.00%	1.57%	0.00%	0.04%	glycolysis Jan 31	
MP:0010052	increased grip strength	3.51E-02	1.00E+00	3.51E-02	1.00E+00	1.00E+00	0	11	336	21447	0.00%	1.57%	0.00%	0.05%	shared jan 31	
					3.55E-02	1.60E-01	2	19	338	21447	10.53%	1.58%	0.59%	0.09%	gluconeogenesis Jan 31	G6pc, Pck1
					1.00E+00	1.00E+00	0	9	338	21447	0.00%	1.58%	0.00%	0.04%	glycolysis Jan 31	
MP:0004847	abnormal liver weight	3.55E-02	1.00E+00	3.55E-02	1.60E-01	3.46E-01	1	11	338	21447	9.09%	1.58%	0.30%	0.05%	shared jan 31	Gpi1
					1.00E+00	1.00E+00	0	19	86	21447	0.00%	0.40%	0.00%	0.09%	gluconeogenesis Jan 31	
					3.55E-02	1.05E-01	1	9	86	21447	11.11%	0.40%	1.16%	0.04%	glycolysis Jan 31	Pklr
MP:0002416	abnormal proerythroblast morphology	3.55E-02	1.00E+00	3.55E-02	1.00E+00	1.00E+00	0	11	86	21447	0.00%	0.40%	0.00%	0.05%	shared jan 31	
					6.11E-02	2.19E-01	1	19	71	21447	5.26%	0.33%	1.41%	0.09%	gluconeogenesis Jan 31	Pck2
					1.00E+00	1.00E+00	0	9	71	21447	0.00%	0.33%	0.00%	0.04%	glycolysis Jan 31	
MP:0004930	small epididymis	3.58E-02	1.00E+00	3.58E-02	3.58E-02	1.47E-01	1	11	71	21447	9.09%	0.33%	1.41%	0.05%	shared jan 31	Eno4
					1.00E+00	1.00E+00	0	19	88	21447	0.00%	0.41%	0.00%	0.09%	gluconeogenesis Jan 31	
					3.63E-02	1.06E-01	1	9	88	21447	11.11%	0.41%	1.14%	0.04%	glycolysis Jan 31	Gck
MP:0011634	abnormal mitochondrial inner membrane morphology	3.63E-02	1.00E+00	3.63E-02	1.00E+00	1.00E+00	0	11	88	21447	0.00%	0.41%	0.00%	0.05%	shared jan 31	
					3.66E-02	1.63E-01	1	19	42	21447	5.26%	0.20%	2.38%	0.09%	gluconeogenesis Jan 31	Pck1
					1.00E+00	1.00E+00	0	9	42	21447	0.00%	0.20%	0.00%	0.04%	glycolysis Jan 31	
MP:0005670	abnormal white adipose tissue physiology	3.66E-02	1.00E+00	3.66E-02	1.00E+00	1.00E+00	0	11	42	21447	0.00%	0.20%	0.00%	0.05%	shared jan 31	
					3.66E-02	1.63E-01	1	19	42	21447	5.26%	0.20%	2.38%	0.09%	gluconeogenesis Jan 31	G6pc
					1.00E+00	1.00E+00	0	9	42	21447	0.00%	0.20%	0.00%	0.04%	glycolysis Jan 31	
MP:0011346	renal tubule atrophy	3.66E-02	1.00E+00	3.66E-02	1.00E+00	1.00E+00	0	11	42	21447	0.00%	0.20%	0.00%	0.05%	shared jan 31	
					1.00E+00	1.00E+00	0	19	89	21447	0.00%	0.41%	0.00%	0.09%	gluconeogenesis Jan 31	
					3.67E-02	1.07E-01	1	9	89	21447	11.11%	0.41%	1.12%	0.04%	glycolysis Jan 31	Gck
MP:0009112	abnormal pancreatic beta cell mass	3.67E-02	1.00E+00	3.67E-02	1.00E+00	1.00E+00	0	11	89	21447	0.00%	0.41%	0.00%	0.05%	shared jan 31	
					3.72E-02	1.64E-01	2	19	347	21447	10.53%	1.62%	0.58%	0.09%	gluconeogenesis Jan 31	Fbp2, Slc25a10
					1.00E+00	1.00E+00	0	9	347	21447	0.00%	1.62%	0.00%	0.04%	glycolysis Jan 31	
MP:0002792	abnormal retina vasculature morphology	3.72E-02	1.00E+00	3.72E-02	1.00E+00	1.00E+00	0	11	347	21447	0.00%	1.62%	0.00%	0.05%	shared jan 31	
					3.74E-02	1.64E-01	1	19	43	21447	5.26%	0.20%	2.33%	0.09%	gluconeogenesis Jan 31	Slc25a12
					1.00E+00	1.00E+00	0	9	43	21447	0.00%	0.20%	0.00%	0.04%	glycolysis Jan 31	
MP:0010957	abnormal aerobic respiration	3.74E-02	1.00E+00	3.74E-02	1.00E+00	1.00E+00	0	11	43	21447	0.00%	0.20%	0.00%	0.05%	shared jan 31	
					3.78E-02	1.65E-01	2	19	350	21447	10.53%	1.63%	0.57%	0.09%	gluconeogenesis Jan 31	Pck1, Pcx
					1.38E-01	2.14E-01	1	9	350	21447	11.11%	1.63%	0.29%	0.04%	glycolysis Jan 31	Gck
MP:0005280	abnormal fatty acids level	3.78E-02	1.00E+00	3.78E-02	1.00E+00	1.00E+00	0	11	350	21447	0.00%	1.63%	0.00%	0.05%	shared jan 31	
					7.85E-02	2.42E-01	1	19	92	21447	5.26%	0.43%	1.09%	0.09%	gluconeogenesis Jan 31	Pck1
					3.80E-02	1.09E-01	1	9	92	21447	11.11%	0.43%	1.09%	0.04%	glycolysis Jan 31	Gck
MP:0005439	decreased glycogen level	3.80E-02	1.00E+00	3.80E-02	1.00E+00	1.00E+00	0	11	92	21447	0.00%	0.43%	0.00%	0.05%	shared jan 31	
					3.81E-02	1.65E-01	3	19	856	21447	15.79%	3.99%	0.35%	0.09%	gluconeogenesis Jan 31	G6pc3, Got1, Slc37a4
					1.00E+00	1.00E+00	0	9	856	21447	0.00%	3.99%	0.00%	0.04%	glycolysis Jan 31	
MP:0002441	abnormal granulocyte morphology	3.81E-02	1.00E+00	3.81E-02	1.00E+00	1.00E+00	0	11	856	21447	0.00%	3.99%	0.00%	0.05%	shared jan 31	
					1.00E+00	1.00E+00	0	19	76	21447	0.00%	0.35%	0.00%	0.09%	gluconeogenesis Jan 31	
					1.00E+00	1.00E+00	0	9	76	21447	0.00%	0.35%	0.00%	0.04%	glycolysis Jan 31	
MP:0009838	abnormal sperm axoneme morphology	3.83E-02	1.00E+00	3.83E-02	3.83E-02	1.51E-01	1	11	76	21447	9.09%	0.35%	1.32%	0.05%	shared jan 31	Eno4
					1.00E+00	1.00E+00	0	19	93	21447	0.00%	0.43%	0.00%	0.09%	gluconeogenesis Jan 31	
					3.84E-02	1.09E-01	1	9	93	21447	11.11%	0.43%	1.08%	0.04%	glycolysis Jan 31	Pfkm
MP:0000749	muscle degeneration	3.84E-02	1.00E+00	3.84E-02	1.00E+00	1.00E+00	0	11	93	21447	0.00%	0.43%	0.00%	0.05%	shared jan 31	

					1.00E+00	1.00E+00	0	19	77	21447	0.00%	0.36%	0.00%	0.09%	gluconeogenesis Jan 31	
					1.00E+00	1.00E+00	0	9	77	21447	0.00%	0.36%	0.00%	0.04%	glycolysis Jan 31	
MP:0020451	decreased sperm progressive motility	3.88E-02	1.00E+00	3.88E-02	3.88E-02	1.51E-01	1	11	77	21447	9.09%	0.36%	1.30%	0.05%	shared jan 31	Pgk2
					1.00E+00	1.00E+00	0	19	77	21447	0.00%	0.36%	0.00%	0.09%	gluconeogenesis Jan 31	
					1.00E+00	1.00E+00	0	9	77	21447	0.00%	0.36%	0.00%	0.04%	glycolysis Jan 31	
MP:0011095	embryonic lethality between implantation and placentation, complete penetrance	3.88E-02	1.00E+00	3.88E-02	3.88E-02	1.51E-01	1	11	77	21447	9.09%	0.36%	1.30%	0.05%	shared jan 31	Gpi1
					3.45E-01	4.57E-01	4	19	3351	21447	21.05%	15.62%	0.12%	0.09%	gluconeogenesis Jan 31	G6pc3, Pck1, Slc25a12, Slc37a4
					3.88E-02	1.10E-01	4	9	3351	21447	44.44%	15.62%	0.12%	0.04%	glycolysis Jan 31	Gck, Hk1, Hk2, Pfkm
MP:0001265	decreased body size	3.88E-02	3.45E-01	1.13E-01	2.40E-01	4.18E-01	3	11	3351	21447	27.27%	15.62%	0.09%	0.05%	shared jan 31	Aldob, Gpi1, Tpi1
					1.00E+00	1.00E+00	0	19	95	21447	0.00%	0.44%	0.00%	0.09%	gluconeogenesis Jan 31	
					3.92E-02	1.10E-01	1	9	95	21447	11.11%	0.44%	1.05%	0.04%	glycolysis Jan 31	Gck
MP:0010519	atrioventricular block	3.92E-02	1.00E+00	3.92E-02	1.00E+00	1.00E+00	0	11	95	21447	0.00%	0.44%	0.00%	0.05%	shared jan 31	
					1.00E+00	1.00E+00	0	19	78	21447	0.00%	0.36%	0.00%	0.09%	gluconeogenesis Jan 31	
					1.00E+00	1.00E+00	0	9	78	21447	0.00%	0.36%	0.00%	0.04%	glycolysis Jan 31	
MP:0008688	decreased interleukin-2 secretion	3.93E-02	1.00E+00	3.93E-02	3.93E-02	1.51E-01	1	11	78	21447	9.09%	0.36%	1.28%	0.05%	shared jan 31	Pgam1
					1.00E+00	1.00E+00	0	19	96	21447	0.00%	0.45%	0.00%	0.09%	gluconeogenesis Jan 31	
					3.96E-02	1.10E-01	1	9	96	21447	11.11%	0.45%	1.04%	0.04%	glycolysis Jan 31	Hk2
MP:0011097	embryonic lethality between somite formation and embryo turning, complete penetrance	3.96E-02	1.00E+00	3.96E-02	1.00E+00	1.00E+00	0	11	96	21447	0.00%	0.45%	0.00%	0.05%	shared jan 31	
					1.00E+00	1.00E+00	0	19	79	21447	0.00%	0.37%	0.00%	0.09%	gluconeogenesis Jan 31	
					1.00E+00	1.00E+00	0	9	79	21447	0.00%	0.37%	0.00%	0.04%	glycolysis Jan 31	
MP:0020450	abnormal sperm progressive motility	3.98E-02	1.00E+00	3.98E-02	3.98E-02	1.51E-01	1	11	79	21447	9.09%	0.37%	1.27%	0.05%	shared jan 31	Pgk2
					3.98E-02	1.69E-01	2	19	360	21447	10.53%	1.68%	0.56%	0.09%	gluconeogenesis Jan 31	Got2, Mdh2
					1.00E+00	1.00E+00	0	9	360	21447	0.00%	1.68%	0.00%	0.04%	glycolysis Jan 31	
MP:0003720	abnormal neural tube closure	3.98E-02	1.00E+00	3.98E-02	1.00E+00	1.00E+00	0	11	360	21447	0.00%	1.68%	0.00%	0.05%	shared jan 31	
					4.00E-02	1.69E-01	1	19	46	21447	5.26%	0.21%	2.17%	0.09%	gluconeogenesis Jan 31	Slc37a4
					1.00E+00	1.00E+00	0	9	46	21447	0.00%	0.21%	0.00%	0.04%	glycolysis Jan 31	
MP:0000133	abnormal long bone metaphysis morphology	4.00E-02	1.00E+00	4.00E-02	1.00E+00	1.00E+00	0	11	46	21447	0.00%	0.21%	0.00%	0.05%	shared jan 31	
					4.00E-02	1.69E-01	1	19	46	21447	5.26%	0.21%	2.17%	0.09%	gluconeogenesis Jan 31	Slc37a4
					1.00E+00	1.00E+00	0	9	46	21447	0.00%	0.21%	0.00%	0.04%	glycolysis Jan 31	
MP:0006396	decreased long bone epiphyseal plate size	4.00E-02	1.00E+00	4.00E-02	1.00E+00	1.00E+00	0	11	46	21447	0.00%	0.21%	0.00%	0.05%	shared jan 31	
					1.00E+00	1.00E+00	0	19	98	21447	0.00%	0.46%	0.00%	0.09%	gluconeogenesis Jan 31	
					4.04E-02	1.11E-01	1	9	98	21447	11.11%	0.46%	1.02%	0.04%	glycolysis Jan 31	Hkdc1
MP:0012144	decreased b-wave amplitude	4.04E-02	1.00E+00	4.04E-02	1.00E+00	1.00E+00	0	11	98	21447	0.00%	0.46%	0.00%	0.05%	shared jan 31	
					1.00E+00	1.00E+00	0	19	99	21447	0.00%	0.46%	0.00%	0.09%	gluconeogenesis Jan 31	
					4.08E-02	1.11E-01	1	9	99	21447	11.11%	0.46%	1.01%	0.04%	glycolysis Jan 31	Pfkm
MP:0012106	impaired exercise endurance	4.08E-02	1.00E+00	4.08E-02	1.00E+00	1.00E+00	0	11	99	21447	0.00%	0.46%	0.00%	0.05%	shared jan 31	
					4.08E-02	1.71E-01	1	19	47	21447	5.26%	0.22%	2.13%	0.09%	gluconeogenesis Jan 31	G6pc3
					1.00E+00	1.00E+00	0	9	47	21447	0.00%	0.22%	0.00%	0.04%	glycolysis Jan 31	
MP:0002695	abnormal circulating glucagon level	4.08E-02	1.00E+00	4.08E-02	1.00E+00	1.00E+00	0	11	47	21447	0.00%	0.22%	0.00%	0.05%	shared jan 31	
					1.00E+00	1.00E+00	0	19	100	21447	0.00%	0.47%	0.00%	0.09%	gluconeogenesis Jan 31	
					4.12E-02	1.11E-01	1	9	100	21447	11.11%	0.47%	1.00%	0.04%	glycolysis Jan 31	Pfkl
MP:0000427	abnormal hair cycle	4.12E-02	1.00E+00	4.12E-02	1.00E+00	1.00E+00	0	11	100	21447	0.00%	0.47%	0.00%	0.05%	shared jan 31	
					1.00E+00	1.00E+00	0	19	100	21447	0.00%	0.47%	0.00%	0.09%	gluconeogenesis Jan 31	
					4.12E-02	1.11E-01	1	9	100	21447	11.11%	0.47%	1.00%	0.04%	glycolysis Jan 31	Gck
MP:0010392	prolonged QRS complex duration	4.12E-02	1.00E+00	4.12E-02	1.00E+00	1.00E+00	0	11	100	21447	0.00%	0.47%	0.00%	0.05%	shared jan 31	
					1.00E+00	1.00E+00	0	19	82	21447	0.00%	0.38%	0.00%	0.09%	gluconeogenesis Jan 31	
					1.00E+00	1.00E+00	0	9	82	21447	0.00%	0.38%	0.00%	0.04%	glycolysis Jan 31	
MP:0008040	decreased NK T cell number	4.13E-02	1.00E+00	4.13E-02	4.13E-02	1.55E-01	1	11	82	21447	9.09%	0.38%	1.22%	0.05%	shared jan 31	Pgam1
					1.00E+00	1.00E+00	0	19	101	21447	0.00%	0.47%	0.00%	0.09%	gluconeogenesis Jan 31	
					4.16E-02	1.11E-01	1	9	101	21447	11.11%	0.47%	0.99%	0.04%	glycolysis Jan 31	Hkdc1
MP:0012031	abnormal b-wave amplitude	4.16E-02	1.00E+00	4.16E-02	1.00E+00	1.00E+00	0	11	101	21447	0.00%	0.47%	0.00%	0.05%	shared jan 31	
					1.00E+00	1.00E+00	0	19	101	21447	0.00%	0.47%	0.00%	0.09%	gluconeogenesis Jan 31	
					4.16E-02	1.11E-01	1	9	101	21447	11.11%	0.47%	0.99%	0.04%	glycolysis Jan 31	Hkdc1
MP:0012035	abnormal b-wave shape	4.16E-02	1.00E+00	4.16E-02	1.00E+00	1.00E+00	0	11	101	21447	0.00%	0.47%	0.00%	0.05%	shared jan 31	
					1.00E+00	1.00E+00	0	19	83	21447	0.00%	0.39%	0.00%	0.09%	gluconeogenesis Jan 31	
					1.00E+00	1.00E+00	0	9	83	21447	0.00%	0.39%	0.00%	0.04%	glycolysis Jan 31	
MP:0005290	decreased oxygen consumption	4.18E-02	1.00E+00	4.18E-02	4.18E-02	1.55E-01	1	11	83	21447	9.09%	0.39%	1.20%	0.05%	shared jan 31	Pgam1
					1.00E+00	1.00E+00	0	19	102	21447	0.00%	0.48%	0.00%	0.09%	gluconeogenesis Jan 31	
					4.20E-02	1.11E-01	1	9	102	21447	11.11%	0.48%	0.98%	0.04%	glycolysis Jan 31	Gck
MP:0010636	bundle branch block	4.20E-02	1.00E+00	4.20E-02	1.00E+00	1.00E+00	0	11	102	21447	0.00%	0.48%	0.00%	0.05%	shared jan 31	
					1.00E+00	1.00E+00	0	19	102	21447	0.00%	0.48%	0.00%	0.09%	gluconeogenesis Jan 31	
					4.20E-02	1.11E-01	1	9	102	21447	11.11%	0.48%	0.98%	0.04%	glycolysis Jan 31	Gck
MP:0010560	intraventricular block	4.20E-02	1.00E+00	4.20E-02	1.00E+00	1.00E+00	0	11	102	21447	0.00%	0.48%	0.00%	0.05%	shared jan 31	
					4.25E-02	1.77E-01	1	19	49	21447	5.26%	0.23%	2.04%	0.09%	gluconeogenesis Jan 31	Slc37a4
					1.00E+00	1.00E+00	0	9	49	21447	0.00%	0.23%	0.00%	0.04%	glycolysis Jan 31	
MP:0008720	impaired neutrophil chemotaxis	4.25E-02	1.00E+00	4.25E-02	1.00E+00	1.00E+00	0	11	49	21447	0.00%	0.23%	0.00%	0.05%	shared jan 31	
					1.00E+00	1.00E+00	0	19	104	21447	0.00%	0.48%	0.00%	0.09%	gluconeogenesis Jan 31	
					4.28E-02	1.11E-01	1	9	104	21447	11.11%	0.48%	0.96%	0.04%	glycolysis Jan 31	Gck
MP:0003901	abnormal PR interval	4.28E-02	1.00E+00	4.28E-02	1.00E+00	1.00E+00	0	11	104	21447	0.00%	0.48%	0.00%	0.05%	shared jan 31	
					4.31E-02	1.77E-01	2	19	376	21447	10.53%	1.75%	0.53%	0.09%	gluconeogenesis Jan 31	Fbp2, Slc25a10

					1.00E+00	1.00E+00	0	9	376	21447	0.00%	1.75%	0.00%	0.04%	glycolysis Jan 31	
MP:0031346	abnormal eye vasculature morphology	4.31E-02	1.00E+00	4.31E-02	1.00E+00	1.00E+00	0	11	376	21447	0.00%	1.75%	0.00%	0.05%	shared jan 31	
					5.34E-01	6.00E-01	2	19	1978	21447	10.53%	9.22%	0.10%	0.09%	gluconeogenesis Jan 31	Fbp2, G6pc3
					4.31E-02	1.11E-01	3	9	1978	21447	33.33%	9.22%	0.15%	0.04%	glycolysis Jan 31	Hk1, Pfkm, Pklr
MP:0011181	increased hematopoietic cell number	4.31E-02	5.34E-01	8.07E-02	2.70E-01	4.40E-01	2	11	1978	21447	18.18%	9.22%	0.10%	0.05%	shared jan 31	Gpi1, Tpi1
					4.34E-02	1.77E-01	1	19	50	21447	5.26%	0.23%	2.00%	0.09%	gluconeogenesis Jan 31	G6pc3
					1.00E+00	1.00E+00	0	9	50	21447	0.00%	0.23%	0.00%	0.04%	glycolysis Jan 31	
MP:0020577	abnormal pancreatic alpha cell number	4.34E-02	1.00E+00	4.34E-02	1.00E+00	1.00E+00	0	11	50	21447	0.00%	0.23%	0.00%	0.05%	shared jan 31	
					4.34E-02	1.77E-01	1	19	50	21447	5.26%	0.23%	2.00%	0.09%	gluconeogenesis Jan 31	Slc25a12
					1.00E+00	1.00E+00	0	9	50	21447	0.00%	0.23%	0.00%	0.04%	glycolysis Jan 31	
MP:0010070	decreased serotonin level	4.34E-02	1.00E+00	4.34E-02	1.00E+00	1.00E+00	0	11	50	21447	0.00%	0.23%	0.00%	0.05%	shared jan 31	
					1.00E+00	1.00E+00	0	19	106	21447	0.00%	0.49%	0.00%	0.09%	gluconeogenesis Jan 31	
					4.36E-02	1.12E-01	1	9	106	21447	11.11%	0.49%	0.94%	0.04%	glycolysis Jan 31	Pklr
MP:0009786	decreased susceptibility to infection induced morbidity/mortality	4.36E-02	1.00E+00	4.36E-02	1.00E+00	1.00E+00	0	11	106	21447	0.00%	0.49%	0.00%	0.05%	shared jan 31	
					1.00E+00	1.00E+00	0	19	87	21447	0.00%	0.41%	0.00%	0.09%	gluconeogenesis Jan 31	
					1.00E+00	1.00E+00	0	9	87	21447	0.00%	0.41%	0.00%	0.04%	glycolysis Jan 31	
MP:0004882	enlarged lung	4.37E-02	1.00E+00	4.37E-02	4.37E-02	1.60E-01	1	11	87	21447	9.09%	0.41%	1.15%	0.05%	shared jan 31	Gpi1
					1.00E+00	1.00E+00	0	19	107	21447	0.00%	0.50%	0.00%	0.09%	gluconeogenesis Jan 31	
					4.40E-02	1.12E-01	1	9	107	21447	11.11%	0.50%	0.93%	0.04%	glycolysis Jan 31	Pfkm
MP:0008569	lethality at weaning	4.40E-02	1.00E+00	4.40E-02	1.00E+00	1.00E+00	0	11	107	21447	0.00%	0.50%	0.00%	0.05%	shared jan 31	
					1.00E+00	1.00E+00	0	19	108	21447	0.00%	0.50%	0.00%	0.09%	gluconeogenesis Jan 31	
					4.44E-02	1.13E-01	1	9	108	21447	11.11%	0.50%	0.93%	0.04%	glycolysis Jan 31	Pklr
MP:0009788	increased susceptibility to bacterial infection induced morbidity/mortality	4.44E-02	1.00E+00	4.44E-02	1.00E+00	1.00E+00	0	11	108	21447	0.00%	0.50%	0.00%	0.05%	shared jan 31	
					1.00E+00	1.00E+00	0	19	89	21447	0.00%	0.41%	0.00%	0.09%	gluconeogenesis Jan 31	
					1.00E+00	1.00E+00	0	9	89	21447	0.00%	0.41%	0.00%	0.04%	glycolysis Jan 31	
MP:0003333	liver fibrosis	4.47E-02	1.00E+00	4.47E-02	4.47E-02	1.62E-01	1	11	89	21447	9.09%	0.41%	1.12%	0.05%	shared jan 31	Aldob
					4.51E-02	1.82E-01	1	19	52	21447	5.26%	0.24%	1.92%	0.09%	gluconeogenesis Jan 31	Slc25a13
					1.00E+00	1.00E+00	0	9	52	21447	0.00%	0.24%	0.00%	0.04%	glycolysis Jan 31	
MP:0006090	abnormal utricle morphology	4.51E-02	1.00E+00	4.51E-02	1.00E+00	1.00E+00	0	11	52	21447	0.00%	0.24%	0.00%	0.05%	shared jan 31	
					4.51E-02	1.82E-01	1	19	52	21447	5.26%	0.24%	1.92%	0.09%	gluconeogenesis Jan 31	G6pc2
					1.00E+00	1.00E+00	0	9	52	21447	0.00%	0.24%	0.00%	0.04%	glycolysis Jan 31	
MP:0004803	increased susceptibility to autoimmune diabetes	4.51E-02	1.00E+00	4.51E-02	1.00E+00	1.00E+00	0	11	52	21447	0.00%	0.24%	0.00%	0.05%	shared jan 31	
					1.00E+00	1.00E+00	0	19	110	21447	0.00%	0.51%	0.00%	0.09%	gluconeogenesis Jan 31	
					4.52E-02	1.13E-01	1	9	110	21447	11.11%	0.51%	0.91%	0.04%	glycolysis Jan 31	Hk2
MP:0004948	abnormal neuronal precursor proliferation	4.52E-02	1.00E+00	4.52E-02	1.00E+00	1.00E+00	0	11	110	21447	0.00%	0.51%	0.00%	0.05%	shared jan 31	
					1.00E+00	1.00E+00	0	19	110	21447	0.00%	0.51%	0.00%	0.09%	gluconeogenesis Jan 31	
					4.52E-02	1.13E-01	1	9	110	21447	11.11%	0.51%	0.91%	0.04%	glycolysis Jan 31	Pfkm
MP:0002899	fatigue	4.52E-02	1.00E+00	4.52E-02	1.00E+00	1.00E+00	0	11	110	21447	0.00%	0.51%	0.00%	0.05%	shared jan 31	
					4.68E-02	1.86E-01	1	19	54	21447	5.26%	0.25%	1.85%	0.09%	gluconeogenesis Jan 31	G6pc2
					1.00E+00	1.00E+00	0	9	54	21447	0.00%	0.25%	0.00%	0.04%	glycolysis Jan 31	
MP:0004031	insulitis	4.68E-02	1.00E+00	4.68E-02	1.00E+00	1.00E+00	0	11	54	21447	0.00%	0.25%	0.00%	0.05%	shared jan 31	
					1.00E+00	1.00E+00	0	19	114	21447	0.00%	0.53%	0.00%	0.09%	gluconeogenesis Jan 31	
					4.68E-02	1.16E-01	1	9	114	21447	11.11%	0.53%	0.88%	0.04%	glycolysis Jan 31	Gck
MP:0010086	abnormal circulating fructosamine level	4.68E-02	1.00E+00	4.68E-02	1.00E+00	1.00E+00	0	11	114	21447	0.00%	0.53%	0.00%	0.05%	shared jan 31	
					4.69E-02	1.86E-01	2	19	394	21447	10.53%	1.84%	0.51%	0.09%	gluconeogenesis Jan 31	Pcx, Slc37a1
					1.00E+00	1.00E+00	0	9	394	21447	0.00%	1.84%	0.00%	0.04%	glycolysis Jan 31	
MP:0010024	increased total body fat amount	4.69E-02	1.00E+00	4.69E-02	1.00E+00	1.00E+00	0	11	394	21447	0.00%	1.84%	0.00%	0.05%	shared jan 31	
					9.71E-02	2.59E-01	1	19	115	21447	5.26%	0.54%	0.87%	0.09%	gluconeogenesis Jan 31	Pck1
					4.72E-02	1.16E-01	1	9	115	21447	11.11%	0.54%	0.87%	0.04%	glycolysis Jan 31	Hkdc1
MP:0009356	decreased liver triglyceride level	4.72E-02	1.00E+00	4.72E-02	1.00E+00	1.00E+00	0	11	115	21447	0.00%	0.54%	0.00%	0.05%	shared jan 31	
					1.00E+00	1.00E+00	0	19	116	21447	0.00%	0.54%	0.00%	0.09%	gluconeogenesis Jan 31	
					4.76E-02	1.16E-01	1	9	116	21447	11.11%	0.54%	0.86%	0.04%	glycolysis Jan 31	Hk2
MP:0006206	embryonic lethality between somite formation and embryo turning	4.76E-02	1.00E+00	4.76E-02	1.00E+00	1.00E+00	0	11	116	21447	0.00%	0.54%	0.00%	0.05%	shared jan 31	
					1.00E+00	1.00E+00	0	19	95	21447	0.00%	0.44%	0.00%	0.09%	gluconeogenesis Jan 31	
					1.00E+00	1.00E+00	0	9	95	21447	0.00%	0.44%	0.00%	0.04%	glycolysis Jan 31	
MP:0008076	abnormal CD4-positive T cell differentiation	4.77E-02	1.00E+00	4.77E-02	4.77E-02	1.67E-01	1	11	95	21447	9.09%	0.44%	1.05%	0.05%	shared jan 31	Pgam1
					1.00E+00	1.00E+00	0	19	95	21447	0.00%	0.44%	0.00%	0.09%	gluconeogenesis Jan 31	
					1.00E+00	1.00E+00	0	9	95	21447	0.00%	0.44%	0.00%	0.04%	glycolysis Jan 31	
MP:0005572	abnormal pulmonary respiratory rate	4.77E-02	1.00E+00	4.77E-02	4.77E-02	1.67E-01	1	11	95	21447	9.09%	0.44%	1.05%	0.05%	shared jan 31	Aldoc
					1.00E+00	1.00E+00	0	19	117	21447	0.00%	0.55%	0.00%	0.09%	gluconeogenesis Jan 31	
					4.80E-02	1.16E-01	1	9	117	21447	11.11%	0.55%	0.85%	0.04%	glycolysis Jan 31	Pfkm
MP:0001157	small seminal vesicle	4.80E-02	1.00E+00	4.80E-02	1.00E+00	1.00E+00	0	11	117	21447	0.00%	0.55%	0.00%	0.05%	shared jan 31	
					1.00E+00	1.00E+00	0	19	96	21447	0.00%	0.45%	0.00%	0.09%	gluconeogenesis Jan 31	
					1.00E+00	1.00E+00	0	9	96	21447	0.00%	0.45%	0.00%	0.04%	glycolysis Jan 31	
MP:0005629	abnormal lung weight	4.82E-02	1.00E+00	4.82E-02	4.82E-02	1.67E-01	1	11	96	21447	9.09%	0.45%	1.04%	0.05%	shared jan 31	Gpi1
					1.00E+00	1.00E+00	0	19	119	21447	0.00%	0.55%	0.00%	0.09%	gluconeogenesis Jan 31	
					4.89E-02	1.17E-01	1	9	119	21447	11.11%	0.55%	0.84%	0.04%	glycolysis Jan 31	Pfkm
MP:0009404	centrally nucleated skeletal muscle fibers	4.89E-02	1.00E+00	4.89E-02	1.00E+00	1.00E+00	0	11	119	21447	0.00%	0.55%	0.00%	0.05%	shared jan 31	
					3.24E-01	4.45E-01	5	19	4277	21447	26.32%	19.94%	0.12%	0.09%	gluconeogenesis Jan 31	Fbp2, G6pc2, G6pc3, Got1, Slc37a4
	abnormal hematopoietic cell				8.48E-02	1.62E-01	4	9	4277	21447	44.44%	19.94%	0.09%	0.04%	glycolysis Jan 31	Gck, Hk1, Pfkm, Pklr

MP:0013656	abnormal hematopoietic cell morphology	4.98E-02	3.24E-01	1.54E-01	4.98E-02	1.71E-01	5	11	4277	21447	45.45%	19.94%	0.12%	0.05%	shared jan 31	Aldoa, Gapdh, Gpi1, Pgam1, Tpi1
					1.00E+00	1.00E+00	0	19	122	21447	0.00%	0.57%	0.00%	0.09%	gluconeogenesis Jan 31	
					5.01E-02	1.20E-01	1	9	122	21447	11.11%	0.57%	0.82%	0.04%	glycolysis Jan 31	Gck
MP:0011925	abnormal heart echocardiography feature	5.01E-02	1.00E+00	5.01E-02	1.00E+00	1.00E+00	0	11	122	21447	0.00%	0.57%	0.00%	0.05%	shared jan 31	
					5.02E-02	1.97E-01	1	19	58	21447	5.26%	0.27%	1.72%	0.09%	gluconeogenesis Jan 31	Slc37a2
					1.00E+00	1.00E+00	0	9	58	21447	0.00%	0.27%	0.00%	0.04%	glycolysis Jan 31	
MP:0008657	increased interleukin-1 beta secretion	5.02E-02	1.00E+00	5.02E-02	1.00E+00	1.00E+00	0	11	58	21447	0.00%	0.27%	0.00%	0.05%	shared jan 31	
					1.00E+00	1.00E+00	0	19	124	21447	0.00%	0.58%	0.00%	0.09%	gluconeogenesis Jan 31	
					5.09E-02	1.21E-01	1	9	124	21447	11.11%	0.58%	0.81%	0.04%	glycolysis Jan 31	Hkdc1
MP:0012029	abnormal electroretinogram waveform feature	5.09E-02	1.00E+00	5.09E-02	1.00E+00	1.00E+00	0	11	124	21447	0.00%	0.58%	0.00%	0.05%	shared jan 31	
					1.00E+00	1.00E+00	0	19	127	21447	0.00%	0.59%	0.00%	0.09%	gluconeogenesis Jan 31	
					5.21E-02	1.23E-01	1	9	127	21447	11.11%	0.59%	0.79%	0.04%	glycolysis Jan 31	Gck
MP:0009170	abnormal pancreatic islet size	5.21E-02	1.00E+00	5.21E-02	1.00E+00	1.00E+00	0	11	127	21447	0.00%	0.59%	0.00%	0.05%	shared jan 31	
					1.00E+00	1.00E+00	0	19	128	21447	0.00%	0.60%	0.00%	0.09%	gluconeogenesis Jan 31	
					5.25E-02	1.23E-01	1	9	128	21447	11.11%	0.60%	0.78%	0.04%	glycolysis Jan 31	Pfkm
MP:0002332	abnormal exercise endurance	5.25E-02	1.00E+00	5.25E-02	1.00E+00	1.00E+00	0	11	128	21447	0.00%	0.60%	0.00%	0.05%	shared jan 31	
					1.00E+00	1.00E+00	0	19	106	21447	0.00%	0.49%	0.00%	0.09%	gluconeogenesis Jan 31	
					1.00E+00	1.00E+00	0	9	106	21447	0.00%	0.49%	0.00%	0.04%	glycolysis Jan 31	
MP:0004800	decreased susceptibility to experimental autoimmune encephalomyelitis	5.31E-02	1.00E+00	5.31E-02	5.31E-02	1.76E-01	1	11	106	21447	9.09%	0.49%	0.94%	0.05%	shared jan 31	Pgam1
					1.00E+00	1.00E+00	0	19	130	21447	0.00%	0.61%	0.00%	0.09%	gluconeogenesis Jan 31	
					5.33E-02	1.24E-01	1	9	130	21447	11.11%	0.61%	0.77%	0.04%	glycolysis Jan 31	Hkdc1
MP:0003925	abnormal cellular glucose uptake	5.33E-02	1.00E+00	5.33E-02	1.00E+00	1.00E+00	0	11	130	21447	0.00%	0.61%	0.00%	0.05%	shared jan 31	
					5.39E-02	2.08E-01	3	19	984	21447	15.79%	4.59%	0.30%	0.09%	gluconeogenesis Jan 31	G6pc, G6pc3, Slc37a4
					3.45E-01	4.02E-01	1	9	984	21447	11.11%	4.59%	0.10%	0.04%	glycolysis Jan 31	Gck
MP:0000180	abnormal circulating cholesterol level	5.39E-02	1.00E+00	5.39E-02	1.00E+00	1.00E+00	0	11	984	21447	0.00%	4.59%	0.00%	0.05%	shared jan 31	
					1.00E+00	1.00E+00	0	19	132	21447	0.00%	0.62%	0.00%	0.09%	gluconeogenesis Jan 31	
					5.41E-02	1.26E-01	1	9	132	21447	11.11%	0.62%	0.76%	0.04%	glycolysis Jan 31	Gck
MP:0006141	abnormal atrioventricular node conduction	5.41E-02	1.00E+00	5.41E-02	1.00E+00	1.00E+00	0	11	132	21447	0.00%	0.62%	0.00%	0.05%	shared jan 31	
					5.44E-02	2.08E-01	1	19	63	21447	5.26%	0.29%	1.59%	0.09%	gluconeogenesis Jan 31	Pcx
					1.00E+00	1.00E+00	0	9	63	21447	0.00%	0.29%	0.00%	0.04%	glycolysis Jan 31	
MP:0010953	abnormal fatty acid oxidation	5.44E-02	1.00E+00	5.44E-02	1.00E+00	1.00E+00	0	11	63	21447	0.00%	0.29%	0.00%	0.05%	shared jan 31	
					5.44E-02	2.08E-01	1	19	63	21447	5.26%	0.29%	1.59%	0.09%	gluconeogenesis Jan 31	G6pc3
					1.00E+00	1.00E+00	0	9	63	21447	0.00%	0.29%	0.00%	0.04%	glycolysis Jan 31	
MP:0005216	abnormal pancreatic alpha cell morphology	5.44E-02	1.00E+00	5.44E-02	1.00E+00	1.00E+00	0	11	63	21447	0.00%	0.29%	0.00%	0.05%	shared jan 31	
					5.44E-02	2.08E-01	1	19	63	21447	5.26%	0.29%	1.59%	0.09%	gluconeogenesis Jan 31	G6pc
					1.00E+00	1.00E+00	0	9	63	21447	0.00%	0.29%	0.00%	0.04%	glycolysis Jan 31	
MP:0009640	abnormal renal tubule epithelium morphology	5.44E-02	1.00E+00	5.44E-02	1.00E+00	1.00E+00	0	11	63	21447	0.00%	0.29%	0.00%	0.05%	shared jan 31	
					1.99E-01	3.43E-01	2	19	932	21447	10.53%	4.35%	0.21%	0.09%	gluconeogenesis Jan 31	G6pc2, Pcx
					5.54E-02	1.27E-01	2	9	932	21447	22.22%	4.35%	0.21%	0.04%	glycolysis Jan 31	Gck, Pfkm
MP:0002164	abnormal gland physiology	5.54E-02	1.00E+00	5.54E-02	1.00E+00	1.00E+00	0	11	932	21447	0.00%	4.35%	0.00%	0.05%	shared jan 31	
					5.55E-02	2.11E-01	4	19	1672	21447	21.05%	7.80%	0.24%	0.09%	gluconeogenesis Jan 31	Fbp2, Got2, Mdh2, Slc25a10
					5.18E-01	5.50E-01	1	9	1672	21447	11.11%	7.80%	0.06%	0.04%	glycolysis Jan 31	Pfkm
MP:0001614	abnormal blood vessel morphology	5.55E-02	1.00E+00	5.55E-02	1.00E+00	1.00E+00	0	11	1672	21447	0.00%	7.80%	0.00%	0.05%	shared jan 31	
					1.00E+00	1.00E+00	0	19	136	21447	0.00%	0.63%	0.00%	0.09%	gluconeogenesis Jan 31	
					5.57E-02	1.27E-01	1	9	136	21447	11.11%	0.63%	0.74%	0.04%	glycolysis Jan 31	Hk2
MP:0006009	abnormal neuronal migration	5.57E-02	1.00E+00	5.57E-02	1.00E+00	1.00E+00	0	11	136	21447	0.00%	0.63%	0.00%	0.05%	shared jan 31	
					1.00E+00	1.00E+00	0	19	137	21447	0.00%	0.64%	0.00%	0.09%	gluconeogenesis Jan 31	
					5.61E-02	1.27E-01	1	9	137	21447	11.11%	0.64%	0.73%	0.04%	glycolysis Jan 31	Pklr
MP:0006410	abnormal common myeloid progenitor cell morphology	5.61E-02	1.00E+00	5.61E-02	1.00E+00	1.00E+00	0	11	137	21447	0.00%	0.64%	0.00%	0.05%	shared jan 31	
					5.61E-02	2.12E-01	2	19	436	21447	10.53%	2.03%	0.46%	0.09%	gluconeogenesis Jan 31	Got2, Mdh2
					1.00E+00	1.00E+00	0	9	436	21447	0.00%	2.03%	0.00%	0.04%	glycolysis Jan 31	
MP:0001695	abnormal gastrulation	5.61E-02	1.00E+00	5.61E-02	1.00E+00	1.00E+00	0	11	436	21447	0.00%	2.03%	0.00%	0.05%	shared jan 31	
					1.00E+00	1.00E+00	0	19	138	21447	0.00%	0.64%	0.00%	0.09%	gluconeogenesis Jan 31	
					5.65E-02	1.27E-01	1	9	138	21447	11.11%	0.64%	0.72%	0.04%	glycolysis Jan 31	Pklr
MP:0008801	abnormal erythroid progenitor cell morphology	5.65E-02	1.00E+00	5.65E-02	1.00E+00	1.00E+00	0	11	138	21447	0.00%	0.64%	0.00%	0.05%	shared jan 31	
					2.72E-01	4.08E-01	4	19	3011	21447	21.05%	14.04%	0.13%	0.09%	gluconeogenesis Jan 31	Fbp2, G6pc3, Got1, Slc37a4
					1.21E-01	1.98E-01	3	9	3011	21447	33.33%	14.04%	0.10%	0.04%	glycolysis Jan 31	Hk1, Pfkm, Pklr
MP:0011180	abnormal hematopoietic cell number	5.65E-02	2.72E-01	2.07E-01	5.65E-02	1.85E-01	4	11	3011	21447	36.36%	14.04%	0.13%	0.05%	shared jan 31	Aldoa, Gpi1, Pgam1, Tpi1
					1.00E+00	1.00E+00	0	19	139	21447	0.00%	0.65%	0.00%	0.09%	gluconeogenesis Jan 31	
					5.69E-02	1.27E-01	1	9	139	21447	11.11%	0.65%	0.72%	0.04%	glycolysis Jan 31	Gck
MP:0003898	abnormal QRS complex	5.69E-02	1.00E+00	5.69E-02	1.00E+00	1.00E+00	0	11	139	21447	0.00%	0.65%	0.00%	0.05%	shared jan 31	
					5.69E-02	2.13E-01	1	19	66	21447	5.26%	0.31%	1.52%	0.09%	gluconeogenesis Jan 31	Slc25a13
					1.00E+00	1.00E+00	0	9	66	21447	0.00%	0.31%	0.00%	0.04%	glycolysis Jan 31	
MP:0004425	abnormal otolith organ morphology	5.69E-02	1.00E+00	5.69E-02	1.00E+00	1.00E+00	0	11	66	21447	0.00%	0.31%	0.00%	0.05%	shared jan 31	
					1.00E+00	1.00E+00	0	19	114	21447	0.00%	0.53%	0.00%	0.09%	gluconeogenesis Jan 31	
					1.00E+00	1.00E+00	0	9	114	21447	0.00%	0.53%	0.00%	0.04%	glycolysis Jan 31	
MP:0008038	abnormal NK T cell number	5.70E-02	1.00E+00	5.70E-02	5.70E-02	1.85E-01	1	11	114	21447	9.09%	0.53%	0.88%	0.05%	shared jan 31	Pgam1
					1.00E+00	1.00E+00	0	19	140	21447	0.00%	0.65%	0.00%	0.09%	gluconeogenesis Jan 31	
					5.72E-02	1.27E-01	1	9	140	21447	11.11%	0.65%	0.71%	0.04%	glycolysis Jan 31	Hkdc1
MP:0003733	abnormal retina inner nuclear layer morphology	5.72E-02	1.00E+00	5.72E-02	1.00E+00	1.00E+00	0	11	140	21447	0.00%	0.65%	0.00%	0.05%	shared jan 31	

					1.00E+00	1.00E+00	0	19	115	21447	0.00%	0.54%	0.00%	0.09%	gluconeogenesis Jan 31	
					1.00E+00	1.00E+00	0	9	115	21447	0.00%	0.54%	0.00%	0.04%	glycolysis Jan 31	
MP:0008036	abnormal NK T cell morphology	5.74E-02	1.00E+00	5.74E-02	5.74E-02	1.85E-01	1	11	115	21447	9.09%	0.54%	0.87%	0.05%	shared jan 31	Pgam1
					1.00E+00	1.00E+00	0	19	143	21447	0.00%	0.67%	0.00%	0.09%	gluconeogenesis Jan 31	
					5.84E-02	1.29E-01	1	9	143	21447	11.11%	0.67%	0.70%	0.04%	glycolysis Jan 31	Pfkm
MP:0002625	heart left ventricle hypertrophy	5.84E-02	1.00E+00	5.84E-02	1.00E+00	1.00E+00	0	11	143	21447	0.00%	0.67%	0.00%	0.05%	shared jan 31	
					5.85E-02	2.17E-01	2	19	446	21447	10.53%	2.08%	0.45%	0.09%	gluconeogenesis Jan 31	G6pc, Slc37a4
					1.00E+00	1.00E+00	0	9	446	21447	0.00%	2.08%	0.00%	0.04%	glycolysis Jan 31	
MP:0000163	abnormal cartilage morphology	5.85E-02	1.00E+00	5.85E-02	1.00E+00	1.00E+00	0	11	446	21447	0.00%	2.08%	0.00%	0.05%	shared jan 31	
					5.86E-02	2.17E-01	1	19	68	21447	5.26%	0.32%	1.47%	0.09%	gluconeogenesis Jan 31	Slc37a4
					1.00E+00	1.00E+00	0	9	68	21447	0.00%	0.32%	0.00%	0.04%	glycolysis Jan 31	
MP:0008722	abnormal chemokine secretion	5.86E-02	1.00E+00	5.86E-02	1.00E+00	1.00E+00	0	11	68	21447	0.00%	0.32%	0.00%	0.05%	shared jan 31	
					5.94E-02	2.18E-01	1	19	69	21447	5.26%	0.32%	1.45%	0.09%	gluconeogenesis Jan 31	Slc37a1
					1.00E+00	1.00E+00	0	9	69	21447	0.00%	0.32%	0.00%	0.04%	glycolysis Jan 31	
MP:0011960	abnormal eye anterior chamber depth	5.94E-02	1.00E+00	5.94E-02	1.00E+00	1.00E+00	0	11	69	21447	0.00%	0.32%	0.00%	0.05%	shared jan 31	
					1.00E+00	1.00E+00	0	19	146	21447	0.00%	0.68%	0.00%	0.09%	gluconeogenesis Jan 31	
					5.96E-02	1.31E-01	1	9	146	21447	11.11%	0.68%	0.68%	0.04%	glycolysis Jan 31	Hk2
MP:0009967	abnormal neuron proliferation	5.96E-02	1.00E+00	5.96E-02	1.00E+00	1.00E+00	0	11	146	21447	0.00%	0.68%	0.00%	0.05%	shared jan 31	
					1.22E-01	2.84E-01	1	19	146	21447	5.26%	0.68%	0.68%	0.09%	gluconeogenesis Jan 31	Slc37a2
					5.96E-02	1.31E-01	1	9	146	21447	11.11%	0.68%	0.68%	0.04%	glycolysis Jan 31	Pfkm
MP:0001148	enlarged testis	5.96E-02	1.00E+00	5.96E-02	1.00E+00	1.00E+00	0	11	146	21447	0.00%	0.68%	0.00%	0.05%	shared jan 31	
					6.02E-02	2.19E-01	1	19	70	21447	5.26%	0.33%	1.43%	0.09%	gluconeogenesis Jan 31	Pcx
					1.00E+00	1.00E+00	0	9	70	21447	0.00%	0.33%	0.00%	0.04%	glycolysis Jan 31	
MP:0010180	increased susceptibility to weight loss	6.02E-02	1.00E+00	6.02E-02	1.00E+00	1.00E+00	0	11	70	21447	0.00%	0.33%	0.00%	0.05%	shared jan 31	
					6.11E-02	2.19E-01	1	19	71	21447	5.26%	0.33%	1.41%	0.09%	gluconeogenesis Jan 31	Pcx
					1.00E+00	1.00E+00	0	9	71	21447	0.00%	0.33%	0.00%	0.04%	glycolysis Jan 31	
MP:0010951	abnormal lipid oxidation	6.11E-02	1.00E+00	6.11E-02	1.00E+00	1.00E+00	0	11	71	21447	0.00%	0.33%	0.00%	0.05%	shared jan 31	
					6.19E-02	2.21E-01	1	19	72	21447	5.26%	0.34%	1.39%	0.09%	gluconeogenesis Jan 31	G6pc
					1.00E+00	1.00E+00	0	9	72	21447	0.00%	0.34%	0.00%	0.04%	glycolysis Jan 31	
MP:0004970	kidney atrophy	6.19E-02	1.00E+00	6.19E-02	1.00E+00	1.00E+00	0	11	72	21447	0.00%	0.34%	0.00%	0.05%	shared jan 31	
					1.00E+00	1.00E+00	0	19	153	21447	0.00%	0.71%	0.00%	0.09%	gluconeogenesis Jan 31	
					6.24E-02	1.35E-01	1	9	153	21447	11.11%	0.71%	0.65%	0.04%	glycolysis Jan 31	Hk2
MP:0000854	abnormal cerebellum development	6.24E-02	1.00E+00	6.24E-02	1.00E+00	1.00E+00	0	11	153	21447	0.00%	0.71%	0.00%	0.05%	shared jan 31	
					1.00E+00	1.00E+00	0	19	153	21447	0.00%	0.71%	0.00%	0.09%	gluconeogenesis Jan 31	
					6.24E-02	1.35E-01	1	9	153	21447	11.11%	0.71%	0.65%	0.04%	glycolysis Jan 31	Hk1
MP:0002356	abnormal spleen red pulp morphology	6.24E-02	1.00E+00	6.24E-02	7.57E-02	2.19E-01	1	11	153	21447	9.09%	0.71%	0.65%	0.05%	shared jan 31	Tpi1
					6.28E-02	2.22E-01	1	19	73	21447	5.26%	0.34%	1.37%	0.09%	gluconeogenesis Jan 31	Slc25a13
					1.00E+00	1.00E+00	0	9	73	21447	0.00%	0.34%	0.00%	0.04%	glycolysis Jan 31	
MP:0002428	abnormal semicircular canal morphology	6.28E-02	1.00E+00	6.28E-02	1.00E+00	1.00E+00	0	11	73	21447	0.00%	0.34%	0.00%	0.05%	shared jan 31	
					1.00E+00	1.00E+00	0	19	126	21447	0.00%	0.59%	0.00%	0.09%	gluconeogenesis Jan 31	
					1.00E+00	1.00E+00	0	9	126	21447	0.00%	0.59%	0.00%	0.04%	glycolysis Jan 31	
MP:0013761	abnormal T-helper cell number	6.28E-02	1.00E+00	6.28E-02	6.28E-02	1.97E-01	1	11	126	21447	9.09%	0.59%	0.79%	0.05%	shared jan 31	Pgam1
					1.00E+00	1.00E+00	0	19	127	21447	0.00%	0.59%	0.00%	0.09%	gluconeogenesis Jan 31	
					1.00E+00	1.00E+00	0	9	127	21447	0.00%	0.59%	0.00%	0.04%	glycolysis Jan 31	
MP:0009831	abnormal sperm midpiece morphology	6.33E-02	1.00E+00	6.33E-02	6.33E-02	1.97E-01	1	11	127	21447	9.09%	0.59%	0.79%	0.05%	shared jan 31	Eno4
					6.35E-02	2.24E-01	3	19	1052	21447	15.79%	4.91%	0.29%	0.09%	gluconeogenesis Jan 31	G6pc, G6pc3, Slc37a4
					3.64E-01	4.22E-01	1	9	1052	21447	11.11%	4.91%	0.10%	0.04%	glycolysis Jan 31	Gck
MP:0003947	abnormal cholesterol level	6.35E-02	1.00E+00	6.35E-02	1.00E+00	1.00E+00	0	11	1052	21447	0.00%	4.91%	0.00%	0.05%	shared jan 31	
					1.00E+00	1.00E+00	0	19	128	21447	0.00%	0.60%	0.00%	0.09%	gluconeogenesis Jan 31	
					1.00E+00	1.00E+00	0	9	128	21447	0.00%	0.60%	0.00%	0.04%	glycolysis Jan 31	
MP:0008686	abnormal interleukin-2 secretion	6.37E-02	1.00E+00	6.37E-02	6.37E-02	1.97E-01	1	11	128	21447	9.09%	0.60%	0.78%	0.05%	shared jan 31	Pgam1
					6.44E-02	2.26E-01	1	19	75	21447	5.26%	0.35%	1.33%	0.09%	gluconeogenesis Jan 31	Pck1
					1.00E+00	1.00E+00	0	9	75	21447	0.00%	0.35%	0.00%	0.04%	glycolysis Jan 31	
MP:0009133	decreased white fat cell size	6.44E-02	1.00E+00	6.44E-02	1.00E+00	1.00E+00	0	11	75	21447	0.00%	0.35%	0.00%	0.05%	shared jan 31	
					6.48E-02	2.26E-01	2	19	473	21447	10.53%	2.21%	0.42%	0.09%	gluconeogenesis Jan 31	Got2, Slc37a4
					1.00E+00	1.00E+00	0	9	473	21447	0.00%	2.21%	0.00%	0.04%	glycolysis Jan 31	
MP:0000692	small spleen	6.48E-02	1.00E+00	6.48E-02	1.00E+00	1.00E+00	0	11	473	21447	0.00%	2.21%	0.00%	0.05%	shared jan 31	
					1.00E+00	1.00E+00	0	19	1023	21447	0.00%	4.77%	0.00%	0.09%	gluconeogenesis Jan 31	
					6.55E-02	1.40E-01	2	9	1023	21447	22.22%	4.77%	0.20%	0.04%	glycolysis Jan 31	Hk1, Pklr
MP:0000192	abnormal mineral level	6.55E-02	1.00E+00	6.55E-02	1.00E+00	1.00E+00	0	11	1023	21447	0.00%	4.77%	0.00%	0.05%	shared jan 31	
					6.06E-01	6.51E-01	1	19	1025	21447	5.26%	4.78%	0.10%	0.09%	gluconeogenesis Jan 31	Fbp2
					6.57E-02	1.40E-01	2	9	1025	21447	22.22%	4.78%	0.20%	0.04%	glycolysis Jan 31	Gck, Pfkm
MP:0011110	preweaning lethality, incomplete penetrance	6.57E-02	6.06E-01	1.08E-01	9.43E-02	2.58E-01	2	11	1025	21447	18.18%	4.78%	0.20%	0.05%	shared jan 31	Aldob, Gapdh
					6.63E-02	2.29E-01	3	19	1071	21447	15.79%	4.99%	0.28%	0.09%	gluconeogenesis Jan 31	G6pc, G6pc3, Slc37a4
					3.69E-01	4.26E-01	1	9	1071	21447	11.11%	4.99%	0.09%	0.04%	glycolysis Jan 31	Gck
MP:0012224	abnormal sterol level	6.63E-02	1.00E+00	6.63E-02	1.00E+00	1.00E+00	0	11	1071	21447	0.00%	4.99%	0.00%	0.05%	shared jan 31	
					1.00E+00	1.00E+00	0	19	1033	21447	0.00%	4.82%	0.00%	0.09%	gluconeogenesis Jan 31	
					6.66E-02	1.41E-01	2	9	1033	21447	22.22%	4.82%	0.19%	0.04%	glycolysis Jan 31	Hk1, Pklr
MP:0005636	abnormal mineral homeostasis	6.66E-02	1.00E+00	6.66E-02	1.00E+00	1.00E+00	0	11	1033	21447	0.00%	4.82%	0.00%	0.05%	shared jan 31	
					1.00E+00	1.00E+00	0	19	134	21447	0.00%	0.62%	0.00%	0.09%	gluconeogenesis Jan 31	

					1.00E+00	1.00E+00	0	9	134	21447	0.00%	0.62%	0.00%	0.04%	glycolysis Jan 31	
MP:0002314	abnormal respiratory mechanics	6.66E-02	1.00E+00	6.66E-02	6.66E-02	2.01E-01	1	11	134	21447	9.09%	0.62%	0.75%	0.05%	shared jan 31	Aldoc
					6.69E-02	2.29E-01	1	19	78	21447	5.26%	0.36%	1.28%	0.09%	gluconeogenesis Jan 31	Pck1
					1.00E+00	1.00E+00	0	9	78	21447	0.00%	0.36%	0.00%	0.04%	glycolysis Jan 31	
MP:0009289	decreased epididymal fat pad weight	6.69E-02	1.00E+00	6.69E-02	1.00E+00	1.00E+00	0	11	78	21447	0.00%	0.36%	0.00%	0.05%	shared jan 31	
					6.70E-02	2.29E-01	2	19	482	21447	10.53%	2.25%	0.41%	0.09%	gluconeogenesis Jan 31	Got2, Mdh1
					1.00E+00	1.00E+00	0	9	482	21447	0.00%	2.25%	0.00%	0.04%	glycolysis Jan 31	
MP:0003935	abnormal craniofacial development	6.70E-02	1.00E+00	6.70E-02	1.00E+00	1.00E+00	0	11	482	21447	0.00%	2.25%	0.00%	0.05%	shared jan 31	
					1.00E+00	1.00E+00	0	19	135	21447	0.00%	0.63%	0.00%	0.09%	gluconeogenesis Jan 31	
					1.00E+00	1.00E+00	0	9	135	21447	0.00%	0.63%	0.00%	0.04%	glycolysis Jan 31	
MP:0010183	abnormal CD4-positive helper T cell morphology	6.71E-02	1.00E+00	6.71E-02	6.71E-02	2.01E-01	1	11	135	21447	9.09%	0.63%	0.74%	0.05%	shared jan 31	Pgam1
					1.00E+00	1.00E+00	0	19	165	21447	0.00%	0.77%	0.00%	0.09%	gluconeogenesis Jan 31	
					6.72E-02	1.42E-01	1	9	165	21447	11.11%	0.77%	0.61%	0.04%	glycolysis Jan 31	Hkdc1
MP:0008515	thin retina outer nuclear layer	6.72E-02	1.00E+00	6.72E-02	1.00E+00	1.00E+00	0	11	165	21447	0.00%	0.77%	0.00%	0.05%	shared jan 31	
					1.00E+00	1.00E+00	0	19	166	21447	0.00%	0.77%	0.00%	0.09%	gluconeogenesis Jan 31	
					6.76E-02	1.42E-01	1	9	166	21447	11.11%	0.77%	0.60%	0.04%	glycolysis Jan 31	Hkdc1
MP:0011997	abnormal retina outer nuclear layer thickness	6.76E-02	1.00E+00	6.76E-02	1.00E+00	1.00E+00	0	11	166	21447	0.00%	0.77%	0.00%	0.05%	shared jan 31	
					6.77E-02	2.30E-01	1	19	79	21447	5.26%	0.37%	1.27%	0.09%	gluconeogenesis Jan 31	Slc25a13
					1.00E+00	1.00E+00	0	9	79	21447	0.00%	0.37%	0.00%	0.04%	glycolysis Jan 31	
MP:0002729	abnormal inner ear canal morphology	6.77E-02	1.00E+00	6.77E-02	1.00E+00	1.00E+00	0	11	79	21447	0.00%	0.37%	0.00%	0.05%	shared jan 31	
					1.00E+00	1.00E+00	0	19	169	21447	0.00%	0.79%	0.00%	0.09%	gluconeogenesis Jan 31	
					6.87E-02	1.44E-01	1	9	169	21447	11.11%	0.79%	0.59%	0.04%	glycolysis Jan 31	Pfkm
MP:0004808	abnormal hematopoietic stem cell morphology	6.87E-02	1.00E+00	6.87E-02	1.00E+00	1.00E+00	0	11	169	21447	0.00%	0.79%	0.00%	0.05%	shared jan 31	
					1.00E+00	1.00E+00	0	19	170	21447	0.00%	0.79%	0.00%	0.09%	gluconeogenesis Jan 31	
					6.91E-02	1.44E-01	1	9	170	21447	11.11%	0.79%	0.59%	0.04%	glycolysis Jan 31	Gck
MP:0010559	heart block	6.91E-02	1.00E+00	6.91E-02	1.00E+00	1.00E+00	0	11	170	21447	0.00%	0.79%	0.00%	0.05%	shared jan 31	
					7.01E-02	2.36E-01	4	19	1806	21447	21.05%	8.42%	0.22%	0.09%	gluconeogenesis Jan 31	G6pc2, G6pc3, Slc37a2, Slc37a4
					1.00E+00	1.00E+00	0	9	1806	21447	0.00%	8.42%	0.00%	0.04%	glycolysis Jan 31	
MP:0002442	abnormal leukocyte physiology	7.01E-02	1.00E+00	7.01E-02	1.00E+00	1.00E+00	0	11	1806	21447	0.00%	8.42%	0.00%	0.05%	shared jan 31	
					7.02E-02	2.36E-01	1	19	82	21447	5.26%	0.38%	1.22%	0.09%	gluconeogenesis Jan 31	G6pc
					1.00E+00	1.00E+00	0	9	82	21447	0.00%	0.38%	0.00%	0.04%	glycolysis Jan 31	
MP:0004967	abnormal kidney epithelium morphology	7.02E-02	1.00E+00	7.02E-02	1.00E+00	1.00E+00	0	11	82	21447	0.00%	0.38%	0.00%	0.05%	shared jan 31	
					1.00E+00	1.00E+00	0	19	173	21447	0.00%	0.81%	0.00%	0.09%	gluconeogenesis Jan 31	
					7.03E-02	1.46E-01	1	9	173	21447	11.11%	0.81%	0.58%	0.04%	glycolysis Jan 31	Pfkm
MP:0004907	abnormal seminal vesicle size	7.03E-02	1.00E+00	7.03E-02	1.00E+00	1.00E+00	0	11	173	21447	0.00%	0.81%	0.00%	0.05%	shared jan 31	
					7.06E-02	2.36E-01	3	19	1100	21447	15.79%	5.13%	0.27%	0.09%	gluconeogenesis Jan 31	G6pc, G6pc3, Slc37a4
					3.77E-01	4.34E-01	1	9	1100	21447	11.11%	5.13%	0.09%	0.04%	glycolysis Jan 31	Gck
MP:0005278	abnormal cholesterol homeostasis	7.06E-02	1.00E+00	7.06E-02	1.00E+00	1.00E+00	0	11	1100	21447	0.00%	5.13%	0.00%	0.05%	shared jan 31	
					7.11E-02	2.36E-01	1	19	83	21447	5.26%	0.39%	1.20%	0.09%	gluconeogenesis Jan 31	Pcx
					1.00E+00	1.00E+00	0	9	83	21447	0.00%	0.39%	0.00%	0.04%	glycolysis Jan 31	
MP:0011118	abnormal susceptibility to weight loss	7.11E-02	1.00E+00	7.11E-02	1.00E+00	1.00E+00	0	11	83	21447	0.00%	0.39%	0.00%	0.05%	shared jan 31	
					7.12E-02	2.36E-01	5	19	2603	21447	26.32%	12.14%	0.19%	0.09%	gluconeogenesis Jan 31	G6pc, Pck1, Pcx, Slc37a2, Slc37a4
					8.55E-02	1.62E-01	3	9	2603	21447	33.33%	12.14%	0.12%	0.04%	glycolysis Jan 31	Hk1, Pfkm, Pklr
MP:0031094	organomegaly	7.12E-02	3.93E-01	1.81E-01	3.93E-01	5.07E-01	2	11	2603	21447	18.18%	12.14%	0.08%	0.05%	shared jan 31	Gpi1, Tpi1
					7.27E-02	2.37E-01	1	19	85	21447	5.26%	0.40%	1.18%	0.09%	gluconeogenesis Jan 31	Slc25a12
					1.00E+00	1.00E+00	0	9	85	21447	0.00%	0.40%	0.00%	0.04%	glycolysis Jan 31	
MP:0005445	abnormal neurotransmitter secretion	7.27E-02	1.00E+00	7.27E-02	1.00E+00	1.00E+00	0	11	85	21447	0.00%	0.40%	0.00%	0.05%	shared jan 31	
					7.27E-02	2.37E-01	1	19	85	21447	5.26%	0.40%	1.18%	0.09%	gluconeogenesis Jan 31	Slc37a2
					1.00E+00	1.00E+00	0	9	85	21447	0.00%	0.40%	0.00%	0.04%	glycolysis Jan 31	
MP:0008735	increased susceptibility to endotoxin shock	7.27E-02	1.00E+00	7.27E-02	1.00E+00	1.00E+00	0	11	85	21447	0.00%	0.40%	0.00%	0.05%	shared jan 31	
					7.28E-02	2.37E-01	6	19	3466	21447	31.58%	16.16%	0.17%	0.09%	gluconeogenesis Jan 31	Fbp2, Got2, Mdh1, Mdh2, Slc25a10, Slc37a1
					4.40E-01	4.89E-01	2	9	3466	21447	22.22%	16.16%	0.06%	0.04%	glycolysis Jan 31	Gck, Pfkm
MP:0002127	abnormal cardiovascular system morphology	7.28E-02	5.51E-01	1.32E-01	5.51E-01	6.05E-01	2	11	3466	21447	18.18%	16.16%	0.06%	0.05%	shared jan 31	Aldob, Gpi1
					2.86E-01	4.16E-01	5	19	4073	21447	26.32%	18.99%	0.12%	0.09%	gluconeogenesis Jan 31	G6pc3, Pck1, Pcx, Slc25a12, Slc37a4
					7.29E-02	1.50E-01	4	9	4073	21447	44.44%	18.99%	0.10%	0.04%	glycolysis Jan 31	Gck, Hk1, Hk2, Pfkm
MP:0003956	abnormal body size	7.29E-02	3.49E-01	2.09E-01	3.49E-01	4.88E-01	3	11	4073	21447	27.27%	18.99%	0.07%	0.05%	shared jan 31	Aldob, Gpi1, Tpi1
					7.35E-02	2.38E-01	1	19	86	21447	5.26%	0.40%	1.16%	0.09%	gluconeogenesis Jan 31	Pck1
					1.00E+00	1.00E+00	0	9	86	21447	0.00%	0.40%	0.00%	0.04%	glycolysis Jan 31	
MP:0009116	abnormal brown fat cell morphology	7.35E-02	1.00E+00	7.35E-02	1.00E+00	1.00E+00	0	11	86	21447	0.00%	0.40%	0.00%	0.05%	shared jan 31	
					1.00E+00	1.00E+00	0	19	182	21447	0.00%	0.85%	0.00%	0.09%	gluconeogenesis Jan 31	
					7.38E-02	1.51E-01	1	9	182	21447	11.11%	0.85%	0.55%	0.04%	glycolysis Jan 31	Pklr
MP:0020184	abnormal susceptibility to parasitic infection	7.38E-02	1.00E+00	7.38E-02	1.00E+00	1.00E+00	0	11	182	21447	0.00%	0.85%	0.00%	0.05%	shared jan 31	
					7.48E-02	2.40E-01	4	19	1846	21447	21.05%	8.61%	0.22%	0.09%	gluconeogenesis Jan 31	G6pc2, G6pc3, Slc37a2, Slc37a4
					1.00E+00	1.00E+00	0	9	1846	21447	0.00%	8.61%	0.00%	0.04%	glycolysis Jan 31	
MP:0001819	abnormal immune cell physiology	7.48E-02	1.00E+00	7.48E-02	1.00E+00	1.00E+00	0	11	1846	21447	0.00%	8.61%	0.00%	0.05%	shared jan 31	
					7.52E-02	2.40E-01	1	19	88	21447	5.26%	0.41%	1.14%	0.09%	gluconeogenesis Jan 31	Slc25a12
					1.00E+00	1.00E+00	0	9	88	21447	0.00%	0.41%	0.00%	0.04%	glycolysis Jan 31	
MP:0005322	abnormal serotonin level	7.52E-02	1.00E+00	7.52E-02	1.00E+00	1.00E+00	0	11	88	21447	0.00%	0.41%	0.00%	0.05%	shared jan 31	
					1.00E+00	1.00E+00	0	19	186	21447	0.00%	0.87%	0.00%	0.09%	gluconeogenesis Jan 31	
	abnormal adrenal gland				7.54E-02	1.53E-01	1	9	186	21447	11.11%	0.87%	0.54%	0.04%	glycolysis Jan 31	Hk3

MP:0000639	abnormal adrenal gland morphology	7.54E-02	1.00E+00	7.54E-02	1.00E+00	1.00E+00	0	11	186	21447	0.00%	0.87%	0.00%	0.05%	shared jan 31	
					7.56E-02	2.40E-01	3	19	1132	21447	15.79%	5.28%	0.27%	0.09%	gluconeogenesis Jan 31	Pck1, Pcx, Slc37a1
					3.86E-01	4.39E-01	1	9	1132	21447	11.11%	5.28%	0.09%	0.04%	glycolysis Jan 31	Pfkm
MP:0005452	abnormal adipose tissue amount	7.56E-02	1.00E+00	7.56E-02	1.00E+00	1.00E+00	0	11	1132	21447	0.00%	5.28%	0.00%	0.05%	shared jan 31	
					7.60E-02	2.40E-01	1	19	89	21447	5.26%	0.41%	1.12%	0.09%	gluconeogenesis Jan 31	Slc25a13
					1.00E+00	1.00E+00	0	9	89	21447	0.00%	0.41%	0.00%	0.04%	glycolysis Jan 31	
MP:0000034	abnormal inner ear vestibule morphology	7.60E-02	1.00E+00	7.60E-02	1.00E+00	1.00E+00	0	11	89	21447	0.00%	0.41%	0.00%	0.05%	shared jan 31	
					7.63E-02	2.40E-01	3	19	1136	21447	15.79%	5.30%	0.26%	0.09%	gluconeogenesis Jan 31	Got2, Mdh1, Mdh2
					1.00E+00	1.00E+00	0	9	1136	21447	0.00%	5.30%	0.00%	0.04%	glycolysis Jan 31	
MP:0002085	abnormal embryonic tissue morphology	7.63E-02	1.00E+00	7.63E-02	4.51E-01	5.46E-01	1	11	1136	21447	9.09%	5.30%	0.09%	0.05%	shared jan 31	Gpi1
					9.04E-01	9.05E-01	1	19	2484	21447	5.26%	11.58%	0.04%	0.09%	gluconeogenesis Jan 31	Pck1
					7.63E-02	1.54E-01	3	9	2484	21447	33.33%	11.58%	0.12%	0.04%	glycolysis Jan 31	Hk2, Pklr, Pkm
MP:0002169	no abnormal phenotype detected	7.63E-02	9.04E-01	8.45E-02	3.70E-01	4.99E-01	2	11	2484	21447	18.18%	11.58%	0.08%	0.05%	shared jan 31	Aldoc, Tpi1
					9.04E-01	9.05E-01	1	19	2487	21447	5.26%	11.60%	0.04%	0.09%	gluconeogenesis Jan 31	Pck1
					7.65E-02	1.54E-01	3	9	2487	21447	33.33%	11.60%	0.12%	0.04%	glycolysis Jan 31	Hk2, Pklr, Pkm
MP:0002873	normal phenotype	7.65E-02	9.04E-01	8.47E-02	3.70E-01	4.99E-01	2	11	2487	21447	18.18%	11.60%	0.08%	0.05%	shared jan 31	Aldoc, Tpi1
					7.66E-02	2.40E-01	4	19	1861	21447	21.05%	8.68%	0.21%	0.09%	gluconeogenesis Jan 31	G6pc2, G6pc3, Slc37a2, Slc37a4
					1.00E+00	1.00E+00	0	9	1861	21447	0.00%	8.68%	0.00%	0.04%	glycolysis Jan 31	
MP:0002421	abnormal cell-mediated immunity	7.66E-02	1.00E+00	7.66E-02	1.00E+00	1.00E+00	0	11	1861	21447	0.00%	8.68%	0.00%	0.05%	shared jan 31	
					7.76E-02	2.42E-01	1	19	91	21447	5.26%	0.42%	1.10%	0.09%	gluconeogenesis Jan 31	Slc37a1
					1.00E+00	1.00E+00	0	9	91	21447	0.00%	0.42%	0.00%	0.04%	glycolysis Jan 31	
MP:0005205	abnormal eye anterior chamber morphology	7.76E-02	1.00E+00	7.76E-02	1.00E+00	1.00E+00	0	11	91	21447	0.00%	0.42%	0.00%	0.05%	shared jan 31	
					1.30E-01	2.92E-01	1	19	157	21447	5.26%	0.73%	0.64%	0.09%	gluconeogenesis Jan 31	Pck2
					1.00E+00	1.00E+00	0	9	157	21447	0.00%	0.73%	0.00%	0.04%	glycolysis Jan 31	
MP:0004926	abnormal epididymis size	7.77E-02	1.00E+00	7.77E-02	7.77E-02	2.22E-01	1	11	157	21447	9.09%	0.73%	0.64%	0.05%	shared jan 31	Eno4
					7.85E-02	2.42E-01	2	19	528	21447	10.53%	2.46%	0.38%	0.09%	gluconeogenesis Jan 31	G6pc3, Slc37a4
					1.00E+00	1.00E+00	0	9	528	21447	0.00%	2.46%	0.00%	0.04%	glycolysis Jan 31	
MP:0012440	abnormal neutrophil cell number	7.85E-02	1.00E+00	7.85E-02	1.00E+00	1.00E+00	0	11	528	21447	0.00%	2.46%	0.00%	0.05%	shared jan 31	
					1.00E+00	1.00E+00	0	19	195	21447	0.00%	0.91%	0.00%	0.09%	gluconeogenesis Jan 31	
					7.89E-02	1.56E-01	1	9	195	21447	11.11%	0.91%	0.51%	0.04%	glycolysis Jan 31	Gck
MP:0005217	abnormal pancreatic beta cell morphology	7.89E-02	1.00E+00	7.89E-02	1.00E+00	1.00E+00	0	11	195	21447	0.00%	0.91%	0.00%	0.05%	shared jan 31	
					1.59E-01	3.16E-01	1	19	195	21447	5.26%	0.91%	0.51%	0.09%	gluconeogenesis Jan 31	Pck1
					7.89E-02	1.56E-01	1	9	195	21447	11.11%	0.91%	0.51%	0.04%	glycolysis Jan 31	Gck
MP:0001559	hyperglycemia	7.89E-02	1.00E+00	7.89E-02	1.00E+00	1.00E+00	0	11	195	21447	0.00%	0.91%	0.00%	0.05%	shared jan 31	
					1.00E+00	1.00E+00	0	19	196	21447	0.00%	0.91%	0.00%	0.09%	gluconeogenesis Jan 31	
					7.93E-02	1.56E-01	1	9	196	21447	11.11%	0.91%	0.51%	0.04%	glycolysis Jan 31	Pklr
MP:0009787	increased susceptibility to infection induced morbidity/mortality	7.93E-02	1.00E+00	7.93E-02	1.00E+00	1.00E+00	0	11	196	21447	0.00%	0.91%	0.00%	0.05%	shared jan 31	
					1.61E-01	3.16E-01	1	19	197	21447	5.26%	0.92%	0.51%	0.09%	gluconeogenesis Jan 31	Pcx
					7.97E-02	1.56E-01	1	9	197	21447	11.11%	0.92%	0.51%	0.04%	glycolysis Jan 31	Gck
MP:0013278	decreased fasting circulating glucose level	7.97E-02	1.00E+00	7.97E-02	1.00E+00	1.00E+00	0	11	197	21447	0.00%	0.92%	0.00%	0.05%	shared jan 31	
					8.01E-02	2.46E-01	1	19	94	21447	5.26%	0.44%	1.06%	0.09%	gluconeogenesis Jan 31	Slc25a12
					1.00E+00	1.00E+00	0	9	94	21447	0.00%	0.44%	0.00%	0.04%	glycolysis Jan 31	
MP:0001524	impaired limb coordination	8.01E-02	1.00E+00	8.01E-02	1.00E+00	1.00E+00	0	11	94	21447	0.00%	0.44%	0.00%	0.05%	shared jan 31	
					8.07E-02	2.46E-01	4	19	1895	21447	21.05%	8.84%	0.21%	0.09%	gluconeogenesis Jan 31	G6pc2, G6pc3, Slc37a2, Slc37a4
					1.00E+00	1.00E+00	0	9	1895	21447	0.00%	8.84%	0.00%	0.04%	glycolysis Jan 31	
MP:0002420	abnormal adaptive immunity	8.07E-02	1.00E+00	8.07E-02	1.00E+00	1.00E+00	0	11	1895	21447	0.00%	8.84%	0.00%	0.05%	shared jan 31	
					8.09E-02	2.46E-01	1	19	95	21447	5.26%	0.44%	1.05%	0.09%	gluconeogenesis Jan 31	Slc25a13
					1.00E+00	1.00E+00	0	9	95	21447	0.00%	0.44%	0.00%	0.04%	glycolysis Jan 31	
MP:0004427	abnormal vestibular labyrinth morphology	8.09E-02	1.00E+00	8.09E-02	1.00E+00	1.00E+00	0	11	95	21447	0.00%	0.44%	0.00%	0.05%	shared jan 31	
					2.77E-01	4.10E-01	2	19	1166	21447	10.53%	5.44%	0.17%	0.09%	gluconeogenesis Jan 31	G6pc3, Slc25a13
					8.24E-02	1.60E-01	2	9	1166	21447	22.22%	5.44%	0.17%	0.04%	glycolysis Jan 31	Gck, Pfkm
MP:0005418	abnormal circulating hormone level	8.24E-02	1.00E+00	8.24E-02	1.00E+00	1.00E+00	0	11	1166	21447	0.00%	5.44%	0.00%	0.05%	shared jan 31	
					1.00E+00	1.00E+00	0	19	2073	21447	0.00%	9.67%	0.00%	0.09%	gluconeogenesis Jan 31	
					6.00E-01	6.23E-01	1	9	2073	21447	11.11%	9.67%	0.05%	0.04%	glycolysis Jan 31	Hk1
MP:0002161	abnormal fertility/fecundity	8.25E-02	1.00E+00	8.25E-02	8.25E-02	2.32E-01	3	11	2073	21447	27.27%	9.67%	0.14%	0.05%	shared jan 31	Eno4, Gapdhs, Pgk2
					8.32E-02	2.50E-01	2	19	546	21447	10.53%	2.55%	0.37%	0.09%	gluconeogenesis Jan 31	Got2, Mdh2
					1.00E+00	1.00E+00	0	9	546	21447	0.00%	2.55%	0.00%	0.04%	glycolysis Jan 31	
MP:0002084	abnormal developmental patterning	8.32E-02	1.00E+00	8.32E-02	1.00E+00	1.00E+00	0	11	546	21447	0.00%	2.55%	0.00%	0.05%	shared jan 31	
					8.32E-02	2.50E-01	2	19	546	21447	10.53%	2.55%	0.37%	0.09%	gluconeogenesis Jan 31	G6pc3, Slc37a4
					1.00E+00	1.00E+00	0	9	546	21447	0.00%	2.55%	0.00%	0.04%	glycolysis Jan 31	
MP:0005065	abnormal neutrophil morphology	8.32E-02	1.00E+00	8.32E-02	1.00E+00	1.00E+00	0	11	546	21447	0.00%	2.55%	0.00%	0.05%	shared jan 31	
					6.57E-01	6.92E-01	1	19	1173	21447	5.26%	5.47%	0.09%	0.09%	gluconeogenesis Jan 31	Slc37a4
					8.33E-02	1.60E-01	2	9	1173	21447	22.22%	5.47%	0.17%	0.04%	glycolysis Jan 31	Hk1, Pklr
MP:0002123	abnormal definitive hematopoiesis	8.33E-02	6.57E-01	1.27E-01	1.19E-01	2.89E-01	2	11	1173	21447	18.18%	5.47%	0.17%	0.05%	shared jan 31	Pgam1, Tpi1
					2.82E-01	4.14E-01	2	19	1183	21447	10.53%	5.52%	0.17%	0.09%	gluconeogenesis Jan 31	Fbp2, Slc25a10
					8.46E-02	1.62E-01	2	9	1183	21447	22.22%	5.52%	0.17%	0.04%	glycolysis Jan 31	Hkdc1, Pkm
MP:0001325	abnormal retina morphology	8.46E-02	4.64E-01	1.82E-01	4.64E-01	5.54E-01	1	11	1183	21447	9.09%	5.52%	0.08%	0.05%	shared jan 31	Aldoa
					8.50E-02	2.50E-01	1	19	100	21447	5.26%	0.47%	1.00%	0.09%	gluconeogenesis Jan 31	Slc25a10
					1.00E+00	1.00E+00	0	9	100	21447	0.00%	0.47%	0.00%	0.04%	glycolysis Jan 31	
MP:0008806	increased circulating amylase level	8.50E-02	1.00E+00	8.50E-02	1.00E+00	1.00E+00	0	11	100	21447	0.00%	0.47%	0.00%	0.05%	shared jan 31	

					8.58E-02	2.50E-01	1	19	101	21447	5.26%	0.47%	0.99%	0.09%	gluconeogenesis Jan 31	Got1
					1.00E+00	1.00E+00	0	9	101	21447	0.00%	0.47%	0.00%	0.04%	glycolysis Jan 31	
MP:0012442	abnormal basophil cell number	8.58E-02	1.00E+00	8.58E-02	1.00E+00	1.00E+00	0	11	101	21447	0.00%	0.47%	0.00%	0.05%	shared jan 31	
					8.58E-02	2.50E-01	1	19	101	21447	5.26%	0.47%	0.99%	0.09%	gluconeogenesis Jan 31	Got1
					1.00E+00	1.00E+00	0	9	101	21447	0.00%	0.47%	0.00%	0.04%	glycolysis Jan 31	
MP:0002422	abnormal basophil morphology	8.58E-02	1.00E+00	8.58E-02	1.00E+00	1.00E+00	0	11	101	21447	0.00%	0.47%	0.00%	0.05%	shared jan 31	
					8.58E-02	2.50E-01	1	19	101	21447	5.26%	0.47%	0.99%	0.09%	gluconeogenesis Jan 31	Pcx
					1.00E+00	1.00E+00	0	9	101	21447	0.00%	0.47%	0.00%	0.04%	glycolysis Jan 31	
MP:0010379	decreased respiratory quotient	8.58E-02	1.00E+00	8.58E-02	1.00E+00	1.00E+00	0	11	101	21447	0.00%	0.47%	0.00%	0.05%	shared jan 31	
					8.58E-02	2.50E-01	1	19	101	21447	5.26%	0.47%	0.99%	0.09%	gluconeogenesis Jan 31	Slc37a2
					1.00E+00	1.00E+00	0	9	101	21447	0.00%	0.47%	0.00%	0.04%	glycolysis Jan 31	
MP:0008705	increased interleukin-6 secretion	8.58E-02	1.00E+00	8.58E-02	1.00E+00	1.00E+00	0	11	101	21447	0.00%	0.47%	0.00%	0.05%	shared jan 31	
					8.61E-02	2.50E-01	3	19	1196	21447	15.79%	5.58%	0.25%	0.09%	gluconeogenesis Jan 31	Fbp2, Slc37a1, Slc37a2
					4.03E-01	4.55E-01	1	9	1196	21447	11.11%	5.58%	0.08%	0.04%	glycolysis Jan 31	Hk3
MP:0005193	abnormal anterior eye segment morphology	8.61E-02	4.68E-01	1.84E-01	4.68E-01	5.54E-01	1	11	1196	21447	9.09%	5.58%	0.08%	0.05%	shared jan 31	Gapdh
					1.00E+00	1.00E+00	0	19	1196	21447	0.00%	5.58%	0.00%	0.09%	gluconeogenesis Jan 31	
					8.62E-02	1.63E-01	2	9	1196	21447	22.22%	5.58%	0.17%	0.04%	glycolysis Jan 31	Hk1, Pklr
MP:0001765	abnormal ion homeostasis	8.62E-02	1.00E+00	8.62E-02	1.00E+00	1.00E+00	0	11	1196	21447	0.00%	5.58%	0.00%	0.05%	shared jan 31	
					1.00E+00	1.00E+00	0	19	175	21447	0.00%	0.82%	0.00%	0.09%	gluconeogenesis Jan 31	
					1.00E+00	1.00E+00	0	9	175	21447	0.00%	0.82%	0.00%	0.04%	glycolysis Jan 31	
MP:0013760	abnormal T-helper cell morphology	8.62E-02	1.00E+00	8.62E-02	8.62E-02	2.40E-01	1	11	175	21447	9.09%	0.82%	0.57%	0.05%	shared jan 31	Pgam1
					6.65E-01	6.96E-01	1	19	1200	21447	5.26%	5.60%	0.08%	0.09%	gluconeogenesis Jan 31	Slc37a4
					8.67E-02	1.63E-01	2	9	1200	21447	22.22%	5.60%	0.17%	0.04%	glycolysis Jan 31	Hk1, Pklr
MP:0013693	abnormal hemopoiesis	8.67E-02	6.65E-01	1.30E-01	1.23E-01	2.96E-01	2	11	1200	21447	18.18%	5.60%	0.17%	0.05%	shared jan 31	Pgam1, Tpi1
					1.75E-01	3.26E-01	1	19	216	21447	5.26%	1.01%	0.46%	0.09%	gluconeogenesis Jan 31	Got2
					8.71E-02	1.63E-01	1	9	216	21447	11.11%	1.01%	0.46%	0.04%	glycolysis Jan 31	Hk2
MP:0006108	abnormal hindbrain development	8.71E-02	1.00E+00	8.71E-02	1.00E+00	1.00E+00	0	11	216	21447	0.00%	1.01%	0.00%	0.05%	shared jan 31	
					8.72E-02	2.51E-01	4	19	1946	21447	21.05%	9.07%	0.21%	0.09%	gluconeogenesis Jan 31	G6pc2, G6pc3, Slc37a2, Slc37a4
					1.94E-01	2.70E-01	2	9	1946	21447	22.22%	9.07%	0.10%	0.04%	glycolysis Jan 31	Pfkm, Pklr
MP:0012382	abnormal blood cell physiology	8.72E-02	2.63E-01	3.31E-01	2.63E-01	4.35E-01	2	11	1946	21447	18.18%	9.07%	0.10%	0.05%	shared jan 31	Gpi1, Tpi1
					8.74E-02	2.51E-01	1	19	103	21447	5.26%	0.48%	0.97%	0.09%	gluconeogenesis Jan 31	Pck1
					1.00E+00	1.00E+00	0	9	103	21447	0.00%	0.48%	0.00%	0.04%	glycolysis Jan 31	
MP:0005459	decreased percent body fat/body weight	8.74E-02	1.00E+00	8.74E-02	1.00E+00	1.00E+00	0	11	103	21447	0.00%	0.48%	0.00%	0.05%	shared jan 31	
					2.92E-01	4.22E-01	2	19	1213	21447	10.53%	5.66%	0.16%	0.09%	gluconeogenesis Jan 31	Fbp2, Slc25a10
					8.83E-02	1.65E-01	2	9	1213	21447	22.22%	5.66%	0.16%	0.04%	glycolysis Jan 31	Hkdc1, Pkm
MP:0002864	abnormal ocular fundus morphology	8.83E-02	4.73E-01	1.87E-01	4.73E-01	5.55E-01	1	11	1213	21447	9.09%	5.66%	0.08%	0.05%	shared jan 31	Aldoa
					8.90E-02	2.54E-01	2	19	568	21447	10.53%	2.65%	0.35%	0.09%	gluconeogenesis Jan 31	G6pc, Slc37a4
					1.00E+00	1.00E+00	0	9	568	21447	0.00%	2.65%	0.00%	0.04%	glycolysis Jan 31	
MP:0002113	abnormal skeleton development	8.90E-02	1.00E+00	8.90E-02	1.00E+00	1.00E+00	0	11	568	21447	0.00%	2.65%	0.00%	0.05%	shared jan 31	
					8.94E-02	2.54E-01	7	19	4565	21447	36.84%	21.29%	0.15%	0.09%	gluconeogenesis Jan 31	Fbp2, Got1, Got2, Mdh1, Mdh2, Slc25a10, Slc37a1
					6.02E-01	6.24E-01	2	9	4565	21447	22.22%	21.29%	0.04%	0.04%	glycolysis Jan 31	Gck, Pfkm
MP:0005385	cardiovascular system phenotype	8.94E-02	6.02E-01	1.49E-01	4.25E-01	5.36E-01	3	11	4565	21447	27.27%	21.29%	0.07%	0.05%	shared jan 31	Aldob, Gapdh, Gpi1
					9.07E-02	2.56E-01	1	19	107	21447	5.26%	0.50%	0.93%	0.09%	gluconeogenesis Jan 31	G6pc
					1.00E+00	1.00E+00	0	9	107	21447	0.00%	0.50%	0.00%	0.04%	glycolysis Jan 31	
MP:0011497	abnormal glomerular capsule visceral layer morphology	9.07E-02	1.00E+00	9.07E-02	1.00E+00	1.00E+00	0	11	107	21447	0.00%	0.50%	0.00%	0.05%	shared jan 31	
					9.07E-02	2.56E-01	1	19	107	21447	5.26%	0.50%	0.93%	0.09%	gluconeogenesis Jan 31	G6pc
					1.00E+00	1.00E+00	0	9	107	21447	0.00%	0.50%	0.00%	0.04%	glycolysis Jan 31	
MP:0005326	abnormal podocyte morphology	9.07E-02	1.00E+00	9.07E-02	1.00E+00	1.00E+00	0	11	107	21447	0.00%	0.50%	0.00%	0.05%	shared jan 31	
					9.14E-02	2.57E-01	2	19	577	21447	10.53%	2.69%	0.35%	0.09%	gluconeogenesis Jan 31	Got2, Mdh2
					2.18E-01	2.89E-01	1	9	577	21447	11.11%	2.69%	0.17%	0.04%	glycolysis Jan 31	Pfkm
MP:0000259	abnormal vascular development	9.14E-02	1.00E+00	9.14E-02	1.00E+00	1.00E+00	0	11	577	21447	0.00%	2.69%	0.00%	0.05%	shared jan 31	
					9.23E-02	2.57E-01	1	19	109	21447	5.26%	0.51%	0.92%	0.09%	gluconeogenesis Jan 31	Slc25a13
					1.00E+00	1.00E+00	0	9	109	21447	0.00%	0.51%	0.00%	0.04%	glycolysis Jan 31	
MP:0001522	impaired swimming	9.23E-02	1.00E+00	9.23E-02	1.00E+00	1.00E+00	0	11	109	21447	0.00%	0.51%	0.00%	0.05%	shared jan 31	
					9.39E-02	2.57E-01	2	19	586	21447	10.53%	2.73%	0.34%	0.09%	gluconeogenesis Jan 31	Pck1, Slc25a12
					2.21E-01	2.91E-01	1	9	586	21447	11.11%	2.73%	0.17%	0.04%	glycolysis Jan 31	Gck
MP:0011085	postnatal lethality, complete penetrance	9.39E-02	1.00E+00	9.39E-02	1.00E+00	1.00E+00	0	11	586	21447	0.00%	2.73%	0.00%	0.05%	shared jan 31	
					9.39E-02	2.57E-01	1	19	111	21447	5.26%	0.52%	0.90%	0.09%	gluconeogenesis Jan 31	G6pc
					1.00E+00	1.00E+00	0	9	111	21447	0.00%	0.52%	0.00%	0.04%	glycolysis Jan 31	
MP:0001654	hepatic necrosis	9.39E-02	1.00E+00	9.39E-02	1.00E+00	1.00E+00	0	11	111	21447	0.00%	0.52%	0.00%	0.05%	shared jan 31	
					9.39E-02	2.57E-01	1	19	111	21447	5.26%	0.52%	0.90%	0.09%	gluconeogenesis Jan 31	Got1
					1.00E+00	1.00E+00	0	9	111	21447	0.00%	0.52%	0.00%	0.04%	glycolysis Jan 31	
MP:0010506	prolonged RR interval	9.39E-02	1.00E+00	9.39E-02	1.00E+00	1.00E+00	0	11	111	21447	0.00%	0.52%	0.00%	0.05%	shared jan 31	
					1.00E+00	1.00E+00	0	19	234	21447	0.00%	1.09%	0.00%	0.09%	gluconeogenesis Jan 31	
					9.40E-02	1.75E-01	1	9	234	21447	11.11%	1.09%	0.43%	0.04%	glycolysis Jan 31	Pfkm
MP:0010579	increased heart left ventricle size	9.40E-02	1.00E+00	9.40E-02	1.00E+00	1.00E+00	0	11	234	21447	0.00%	1.09%	0.00%	0.05%	shared jan 31	
					9.47E-02	2.57E-01	2	19	589	21447	10.53%	2.75%	0.34%	0.09%	gluconeogenesis Jan 31	G6pc, Slc37a4
					1.00E+00	1.00E+00	0	9	589	21447	0.00%	2.75%	0.00%	0.04%	glycolysis Jan 31	
MP:0005178	increased circulating cholesterol level	9.47E-02	1.00E+00	9.47E-02	1.00E+00	1.00E+00	0	11	589	21447	0.00%	2.75%	0.00%	0.05%	shared jan 31	

					9.47E-02	2.57E-01	1	19	112	21447	5.26%	0.52%	0.89%	0.09%	gluconeogenesis Jan 31	Slc37a2
					1.00E+00	1.00E+00	0	9	112	21447	0.00%	0.52%	0.00%	0.04%	glycolysis Jan 31	
MP:0008656	abnormal interleukin-1 beta secretion	9.47E-02	1.00E+00	9.47E-02	1.00E+00	1.00E+00	0	11	112	21447	0.00%	0.52%	0.00%	0.05%	shared jan 31	
					9.47E-02	2.57E-01	1	19	112	21447	5.26%	0.52%	0.89%	0.09%	gluconeogenesis Jan 31	Pck1
					1.00E+00	1.00E+00	0	9	112	21447	0.00%	0.52%	0.00%	0.04%	glycolysis Jan 31	
MP:0001783	decreased white adipose tissue amount	9.47E-02	1.00E+00	9.47E-02	1.00E+00	1.00E+00	0	11	112	21447	0.00%	0.52%	0.00%	0.05%	shared jan 31	
					9.50E-02	2.57E-01	2	19	590	21447	10.53%	2.75%	0.34%	0.09%	gluconeogenesis Jan 31	G6pc, Slc25a13
					2.22E-01	2.91E-01	1	9	590	21447	11.11%	2.75%	0.17%	0.04%	glycolysis Jan 31	Gck
MP:0009643	abnormal urine homeostasis	9.50E-02	1.00E+00	9.50E-02	1.00E+00	1.00E+00	0	11	590	21447	0.00%	2.75%	0.00%	0.05%	shared jan 31	
					9.55E-02	2.57E-01	1	19	113	21447	5.26%	0.53%	0.88%	0.09%	gluconeogenesis Jan 31	Got2
					1.00E+00	1.00E+00	0	9	113	21447	0.00%	0.53%	0.00%	0.04%	glycolysis Jan 31	
MP:0013294	prenatal lethality prior to heart atrial septation	9.55E-02	1.00E+00	9.55E-02	1.00E+00	1.00E+00	0	11	113	21447	0.00%	0.53%	0.00%	0.05%	shared jan 31	
					1.00E+00	1.00E+00	0	19	195	21447	0.00%	0.91%	0.00%	0.09%	gluconeogenesis Jan 31	
					1.00E+00	1.00E+00	0	9	195	21447	0.00%	0.91%	0.00%	0.04%	glycolysis Jan 31	
MP:0005087	decreased acute inflammation	9.56E-02	1.00E+00	9.56E-02	9.56E-02	2.60E-01	1	11	195	21447	9.09%	0.91%	0.51%	0.05%	shared jan 31	Pgam1
					9.63E-02	2.58E-01	1	19	114	21447	5.26%	0.53%	0.88%	0.09%	gluconeogenesis Jan 31	Pck1
					1.00E+00	1.00E+00	0	9	114	21447	0.00%	0.53%	0.00%	0.04%	glycolysis Jan 31	
MP:0009546	absent gastric milk in neonates	9.63E-02	1.00E+00	9.63E-02	1.00E+00	1.00E+00	0	11	114	21447	0.00%	0.53%	0.00%	0.05%	shared jan 31	
					1.00E+00	1.00E+00	0	19	241	21447	0.00%	1.12%	0.00%	0.09%	gluconeogenesis Jan 31	
					9.67E-02	1.79E-01	1	9	241	21447	11.11%	1.12%	0.41%	0.04%	glycolysis Jan 31	Hk1
MP:0005562	decreased mean corpuscular hemoglobin	9.67E-02	1.00E+00	9.67E-02	1.17E-01	2.88E-01	1	11	241	21447	9.09%	1.12%	0.41%	0.05%	shared jan 31	Gpi1
					1.45E-01	3.08E-01	7	19	5119	21447	36.84%	23.87%	0.14%	0.09%	gluconeogenesis Jan 31	Fbp2, G6pc2, G6pc3, Got1, Got2, Slc37a2, Slc37a4
					1.45E-01	2.20E-01	4	9	5119	21447	44.44%	23.87%	0.08%	0.04%	glycolysis Jan 31	Gck, Hk1, Pfkm, Pklr
MP:0002396	abnormal hematopoietic system morphology/development	9.72E-02	1.45E-01	6.69E-01	9.72E-02	2.61E-01	5	11	5119	21447	45.45%	23.87%	0.10%	0.05%	shared jan 31	Aldoa, Gapdh, Gpi1, Pgam1, Tpi1
					9.79E-02	2.59E-01	5	19	2855	21447	26.32%	13.31%	0.18%	0.09%	gluconeogenesis Jan 31	Fbp2, G6pc2, G6pc3, Got1, Slc37a4
					1.00E+00	1.00E+00	0	9	2855	21447	0.00%	13.31%	0.00%	0.04%	glycolysis Jan 31	
MP:0008246	abnormal leukocyte morphology	9.79E-02	1.00E+00	9.79E-02	4.41E-01	5.46E-01	2	11	2855	21447	18.18%	13.31%	0.07%	0.05%	shared jan 31	Aldoa, Pgam1
					1.63E-01	3.17E-01	1	19	200	21447	5.26%	0.93%	0.50%	0.09%	gluconeogenesis Jan 31	Slc25a10
					1.00E+00	1.00E+00	0	9	200	21447	0.00%	0.93%	0.00%	0.04%	glycolysis Jan 31	
MP:0011965	decreased total retina thickness	9.79E-02	1.00E+00	9.79E-02	9.79E-02	2.61E-01	1	11	200	21447	9.09%	0.93%	0.50%	0.05%	shared jan 31	Aldoa
					9.88E-02	2.59E-01	1	19	117	21447	5.26%	0.55%	0.85%	0.09%	gluconeogenesis Jan 31	Pck1
					1.00E+00	1.00E+00	0	9	117	21447	0.00%	0.55%	0.00%	0.04%	glycolysis Jan 31	
MP:0020998	abnormal epididymal fat pad weight	9.88E-02	1.00E+00	9.88E-02	1.00E+00	1.00E+00	0	11	117	21447	0.00%	0.55%	0.00%	0.05%	shared jan 31	
					9.88E-02	2.59E-01	1	19	117	21447	5.26%	0.55%	0.85%	0.09%	gluconeogenesis Jan 31	Slc25a12
					1.00E+00	1.00E+00	0	9	117	21447	0.00%	0.55%	0.00%	0.04%	glycolysis Jan 31	
MP:0004077	abnormal striatum morphology	9.88E-02	1.00E+00	9.88E-02	1.00E+00	1.00E+00	0	11	117	21447	0.00%	0.55%	0.00%	0.05%	shared jan 31	
					9.88E-02	2.59E-01	2	19	604	21447	10.53%	2.82%	0.33%	0.09%	gluconeogenesis Jan 31	Got2, Mdh2
					1.00E+00	1.00E+00	0	9	604	21447	0.00%	2.82%	0.00%	0.04%	glycolysis Jan 31	
MP:0002151	abnormal neural tube morphology	9.88E-02	1.00E+00	9.88E-02	1.00E+00	1.00E+00	0	11	604	21447	0.00%	2.82%	0.00%	0.05%	shared jan 31	
					1.05E-01	2.65E-01	3	19	1303	21447	15.79%	6.08%	0.23%	0.09%	gluconeogenesis Jan 31	G6pc3, Slc25a12, Slc25a13
					9.99E-02	1.82E-01	2	9	1303	21447	22.22%	6.08%	0.15%	0.04%	glycolysis Jan 31	Gck, Pfkm
MP:0003953	abnormal hormone level	9.99E-02	1.00E+00	9.99E-02	1.00E+00	1.00E+00	0	11	1303	21447	0.00%	6.08%	0.00%	0.05%	shared jan 31	
					1.00E+00	1.00E+00	0	19	205	21447	0.00%	0.96%	0.00%	0.09%	gluconeogenesis Jan 31	
					1.00E+00	1.00E+00	0	9	205	21447	0.00%	0.96%	0.00%	0.04%	glycolysis Jan 31	
MP:0001860	liver inflammation	1.00E-01	1.00E+00	1.00E-01	1.00E-01	2.65E-01	1	11	205	21447	9.09%	0.96%	0.49%	0.05%	shared jan 31	Aldob
					1.00E-01	2.60E-01	1	19	119	21447	5.26%	0.55%	0.84%	0.09%	gluconeogenesis Jan 31	Slc25a12
					1.00E+00	1.00E+00	0	9	119	21447	0.00%	0.55%	0.00%	0.04%	glycolysis Jan 31	
MP:0008535	enlarged lateral ventricles	1.00E-01	1.00E+00	1.00E-01	1.00E+00	1.00E+00	0	11	119	21447	0.00%	0.55%	0.00%	0.05%	shared jan 31	
					2.00E-01	3.44E-01	1	19	251	21447	5.26%	1.17%	0.40%	0.09%	gluconeogenesis Jan 31	Pck1
					1.01E-01	1.82E-01	1	9	251	21447	11.11%	1.17%	0.40%	0.04%	glycolysis Jan 31	Hk1
MP:0003717	pallor	1.01E-01	1.00E+00	1.01E-01	1.00E+00	1.00E+00	0	11	251	21447	0.00%	1.17%	0.00%	0.05%	shared jan 31	
					1.01E-01	2.60E-01	5	19	2882	21447	26.32%	13.44%	0.17%	0.09%	gluconeogenesis Jan 31	Fbp2, G6pc2, G6pc3, Got1, Slc37a4
					1.00E+00	1.00E+00	0	9	2882	21447	0.00%	13.44%	0.00%	0.04%	glycolysis Jan 31	
MP:0000716	abnormal immune system cell morphology	1.01E-01	1.00E+00	1.01E-01	4.46E-01	5.46E-01	2	11	2882	21447	18.18%	13.44%	0.07%	0.05%	shared jan 31	Aldoa, Pgam1
					1.01E-01	2.60E-01	1	19	120	21447	5.26%	0.56%	0.83%	0.09%	gluconeogenesis Jan 31	Pck1
					1.00E+00	1.00E+00	0	9	120	21447	0.00%	0.56%	0.00%	0.04%	glycolysis Jan 31	
MP:0009132	abnormal white fat cell size	1.01E-01	1.00E+00	1.01E-01	1.00E+00	1.00E+00	0	11	120	21447	0.00%	0.56%	0.00%	0.05%	shared jan 31	
					1.01E-01	2.60E-01	1	19	120	21447	5.26%	0.56%	0.83%	0.09%	gluconeogenesis Jan 31	Pck1
					1.00E+00	1.00E+00	0	9	120	21447	0.00%	0.56%	0.00%	0.04%	glycolysis Jan 31	
MP:0009269	decreased fat cell size	1.01E-01	1.00E+00	1.01E-01	1.00E+00	1.00E+00	0	11	120	21447	0.00%	0.56%	0.00%	0.05%	shared jan 31	
					1.03E-01	2.63E-01	1	19	122	21447	5.26%	0.57%	0.82%	0.09%	gluconeogenesis Jan 31	Slc37a2
					1.00E+00	1.00E+00	0	9	122	21447	0.00%	0.57%	0.00%	0.04%	glycolysis Jan 31	
MP:0008650	abnormal interleukin-1 secretion	1.03E-01	1.00E+00	1.03E-01	1.00E+00	1.00E+00	0	11	122	21447	0.00%	0.57%	0.00%	0.05%	shared jan 31	
					1.00E+00	1.00E+00	0	19	257	21447	0.00%	1.20%	0.00%	0.09%	gluconeogenesis Jan 31	
					1.03E-01	1.84E-01	1	9	257	21447	11.11%	1.20%	0.39%	0.04%	glycolysis Jan 31	Gck
MP:0013279	increased fasting circulating glucose level	1.03E-01	1.00E+00	1.03E-01	1.00E+00	1.00E+00	0	11	257	21447	0.00%	1.20%	0.00%	0.05%	shared jan 31	
					1.00E+00	1.00E+00	0	19	257	21447	0.00%	1.20%	0.00%	0.09%	gluconeogenesis Jan 31	
					1.03E-01	1.84E-01	1	9	257	21447	11.11%	1.20%	0.39%	0.04%	glycolysis Jan 31	Gck
MP:0005331	insulin resistance	1.03E-01	1.00E+00	1.03E-01	1.00E+00	1.00E+00	0	11	257	21447	0.00%	1.20%	0.00%	0.05%	shared jan 31	

					1.04E-01	2.65E-01	1	19	124	21447	5.26%	0.58%	0.81%	0.09%	gluconeogenesis Jan 31	G6pc2
					1.00E+00	1.00E+00	0	9	124	21447	0.00%	0.58%	0.00%	0.04%	glycolysis Jan 31	
MP:0001869	pancreas inflammation	1.04E-01	1.00E+00	1.04E-01	1.00E+00	1.00E+00	0	11	124	21447	0.00%	0.58%	0.00%	0.05%	shared jan 31	
					1.04E-01	2.65E-01	1	19	124	21447	5.26%	0.58%	0.81%	0.09%	gluconeogenesis Jan 31	Mdh1
					1.00E+00	1.00E+00	0	9	124	21447	0.00%	0.58%	0.00%	0.04%	glycolysis Jan 31	
MP:0002953	thick ventricular wall	1.04E-01	1.00E+00	1.04E-01	1.00E+00	1.00E+00	0	11	124	21447	0.00%	0.58%	0.00%	0.05%	shared jan 31	
					1.76E-01	3.26E-01	1	19	217	21447	5.26%	1.01%	0.46%	0.09%	gluconeogenesis Jan 31	Slc25a10
					1.00E+00	1.00E+00	0	9	217	21447	0.00%	1.01%	0.00%	0.04%	glycolysis Jan 31	
MP:0011963	abnormal total retina thickness	1.06E-01	1.00E+00	1.06E-01	1.06E-01	2.76E-01	1	11	217	21447	9.09%	1.01%	0.46%	0.05%	shared jan 31	Aldoa
					1.06E-01	2.66E-01	4	19	2083	21447	21.05%	9.71%	0.19%	0.09%	gluconeogenesis Jan 31	G6pc2, G6pc3, Slc37a2, Slc37a4
					2.15E-01	2.89E-01	2	9	2083	21447	22.22%	9.71%	0.10%	0.04%	glycolysis Jan 31	Pfkm, Pklr
MP:0001545	abnormal hematopoietic system physiology	1.06E-01	2.90E-01	3.65E-01	2.90E-01	4.57E-01	2	11	2083	21447	18.18%	9.71%	0.10%	0.05%	shared jan 31	Gpi1, Tpi1
					7.11E-01	7.29E-01	1	19	1355	21447	5.26%	6.32%	0.07%	0.09%	gluconeogenesis Jan 31	G6pc3
					1.07E-01	1.90E-01	2	9	1355	21447	22.22%	6.32%	0.15%	0.04%	glycolysis Jan 31	Gck, Hk3
MP:0013560	abnormal endocrine gland morphology	1.07E-01	1.00E+00	1.07E-01	1.00E+00	1.00E+00	0	11	1355	21447	0.00%	6.32%	0.00%	0.05%	shared jan 31	
					1.08E-01	2.69E-01	1	19	128	21447	5.26%	0.60%	0.78%	0.09%	gluconeogenesis Jan 31	Slc25a12
					1.00E+00	1.00E+00	0	9	128	21447	0.00%	0.60%	0.00%	0.04%	glycolysis Jan 31	
MP:0001905	abnormal dopamine level	1.08E-01	1.00E+00	1.08E-01	1.00E+00	1.00E+00	0	11	128	21447	0.00%	0.60%	0.00%	0.05%	shared jan 31	
					1.08E-01	2.69E-01	1	19	129	21447	5.26%	0.60%	0.78%	0.09%	gluconeogenesis Jan 31	Slc37a2
					1.00E+00	1.00E+00	0	9	129	21447	0.00%	0.60%	0.00%	0.04%	glycolysis Jan 31	
MP:0008560	increased tumor necrosis factor secretion	1.08E-01	1.00E+00	1.08E-01	1.00E+00	1.00E+00	0	11	129	21447	0.00%	0.60%	0.00%	0.05%	shared jan 31	
					7.15E-01	7.32E-01	1	19	1369	21447	5.26%	6.38%	0.07%	0.09%	gluconeogenesis Jan 31	Slc37a4
					1.09E-01	1.91E-01	2	9	1369	21447	22.22%	6.38%	0.15%	0.04%	glycolysis Jan 31	Hk1, Pklr
MP:0002429	abnormal blood cell morphology/development	1.09E-01	7.15E-01	1.52E-01	1.53E-01	3.34E-01	2	11	1369	21447	18.18%	6.38%	0.15%	0.05%	shared jan 31	Pgam1, Tpi1
					1.00E+00	1.00E+00	0	19	273	21447	0.00%	1.27%	0.00%	0.09%	gluconeogenesis Jan 31	
					1.09E-01	1.91E-01	1	9	273	21447	11.11%	1.27%	0.37%	0.04%	glycolysis Jan 31	Pfkl
MP:0010680	abnormal skin adnexa physiology	1.09E-01	1.00E+00	1.09E-01	1.00E+00	1.00E+00	0	11	273	21447	0.00%	1.27%	0.00%	0.05%	shared jan 31	
					1.09E-01	2.69E-01	9	19	6756	21447	47.37%	31.50%	0.13%	0.09%	gluconeogenesis Jan 31	Fbp2, G6pc, G6pc2, Pck1, Pcx, Slc25a10, Slc25a12, Slc25a13, Slc37a4
					9.67E-01	9.69E-01	1	9	6756	21447	11.11%	31.50%	0.01%	0.04%	glycolysis Jan 31	Pfkm
MP:0005386	behavior/neurological phenotype	1.09E-01	9.67E-01	1.13E-01	9.06E-01	9.14E-01	2	11	6756	21447	18.18%	31.50%	0.03%	0.05%	shared jan 31	Aldoa, Aldoc
					1.09E-01	2.69E-01	1	19	130	21447	5.26%	0.61%	0.77%	0.09%	gluconeogenesis Jan 31	Got1
					1.00E+00	1.00E+00	0	9	130	21447	0.00%	0.61%	0.00%	0.04%	glycolysis Jan 31	
MP:0010504	abnormal RR interval	1.09E-01	1.00E+00	1.09E-01	1.00E+00	1.00E+00	0	11	130	21447	0.00%	0.61%	0.00%	0.05%	shared jan 31	
					1.00E+00	1.00E+00	0	19	274	21447	0.00%	1.28%	0.00%	0.09%	gluconeogenesis Jan 31	
					1.09E-01	1.91E-01	1	9	274	21447	11.11%	1.28%	0.36%	0.04%	glycolysis Jan 31	Pfkl
MP:0000377	abnormal hair follicle morphology	1.09E-01	1.00E+00	1.09E-01	1.00E+00	1.00E+00	0	11	274	21447	0.00%	1.28%	0.00%	0.05%	shared jan 31	
					1.00E+00	1.00E+00	0	19	274	21447	0.00%	1.28%	0.00%	0.09%	gluconeogenesis Jan 31	
					1.09E-01	1.91E-01	1	9	274	21447	11.11%	1.28%	0.36%	0.04%	glycolysis Jan 31	Gck
MP:0006035	abnormal mitochondrial morphology	1.09E-01	1.00E+00	1.09E-01	1.00E+00	1.00E+00	0	11	274	21447	0.00%	1.28%	0.00%	0.05%	shared jan 31	
					1.00E+00	1.00E+00	0	19	225	21447	0.00%	1.05%	0.00%	0.09%	gluconeogenesis Jan 31	
					1.00E+00	1.00E+00	0	9	225	21447	0.00%	1.05%	0.00%	0.04%	glycolysis Jan 31	
MP:0013762	abnormal effector T cell number	1.10E-01	1.00E+00	1.10E-01	1.10E-01	2.79E-01	1	11	225	21447	9.09%	1.05%	0.44%	0.05%	shared jan 31	Pgam1
					1.00E+00	1.00E+00	0	19	276	21447	0.00%	1.29%	0.00%	0.09%	gluconeogenesis Jan 31	
					1.10E-01	1.91E-01	1	9	276	21447	11.11%	1.29%	0.36%	0.04%	glycolysis Jan 31	Pklr
MP:0009785	abnormal susceptibility to infection induced morbidity/mortality	1.10E-01	1.00E+00	1.10E-01	1.00E+00	1.00E+00	0	11	276	21447	0.00%	1.29%	0.00%	0.05%	shared jan 31	
					1.00E+00	1.00E+00	0	19	276	21447	0.00%	1.29%	0.00%	0.09%	gluconeogenesis Jan 31	
					1.10E-01	1.91E-01	1	9	276	21447	11.11%	1.29%	0.36%	0.04%	glycolysis Jan 31	Hk1
MP:0010067	increased red blood cell distribution width	1.10E-01	1.00E+00	1.10E-01	1.00E+00	1.00E+00	0	11	276	21447	0.00%	1.29%	0.00%	0.05%	shared jan 31	
					1.00E+00	1.00E+00	0	19	227	21447	0.00%	1.06%	0.00%	0.09%	gluconeogenesis Jan 31	
					1.00E+00	1.00E+00	0	9	227	21447	0.00%	1.06%	0.00%	0.04%	glycolysis Jan 31	
MP:0004881	abnormal lung size	1.10E-01	1.00E+00	1.10E-01	1.10E-01	2.79E-01	1	11	227	21447	9.09%	1.06%	0.44%	0.05%	shared jan 31	Gpi1
					1.00E+00	1.00E+00	0	19	227	21447	0.00%	1.06%	0.00%	0.09%	gluconeogenesis Jan 31	
					1.00E+00	1.00E+00	0	9	227	21447	0.00%	1.06%	0.00%	0.04%	glycolysis Jan 31	
MP:0020870	decreased thigmotaxis	1.10E-01	1.00E+00	1.10E-01	1.10E-01	2.79E-01	1	11	227	21447	9.09%	1.06%	0.44%	0.05%	shared jan 31	Aldoa
					1.11E-01	2.69E-01	2	19	646	21447	10.53%	3.01%	0.31%	0.09%	gluconeogenesis Jan 31	G6pc, Slc37a4
					1.00E+00	1.00E+00	0	9	646	21447	0.00%	3.01%	0.00%	0.04%	glycolysis Jan 31	
MP:0003982	increased cholesterol level	1.11E-01	1.00E+00	1.11E-01	1.00E+00	1.00E+00	0	11	646	21447	0.00%	3.01%	0.00%	0.05%	shared jan 31	
					1.11E-01	2.69E-01	1	19	132	21447	5.26%	0.62%	0.76%	0.09%	gluconeogenesis Jan 31	G6pc2
					1.00E+00	1.00E+00	0	9	132	21447	0.00%	0.62%	0.00%	0.04%	glycolysis Jan 31	
MP:0004392	abnormal CD8-positive, alpha-beta T cell physiology	1.11E-01	1.00E+00	1.11E-01	1.00E+00	1.00E+00	0	11	132	21447	0.00%	0.62%	0.00%	0.05%	shared jan 31	
					1.11E-01	2.69E-01	1	19	132	21447	5.26%	0.62%	0.76%	0.09%	gluconeogenesis Jan 31	Slc25a12
					1.00E+00	1.00E+00	0	9	132	21447	0.00%	0.62%	0.00%	0.04%	glycolysis Jan 31	
MP:0002797	increased thigmotaxis	1.11E-01	1.00E+00	1.11E-01	1.00E+00	1.00E+00	0	11	132	21447	0.00%	0.62%	0.00%	0.05%	shared jan 31	
					1.00E+00	1.00E+00	0	19	2348	21447	0.00%	10.95%	0.00%	0.09%	gluconeogenesis Jan 31	
					6.48E-01	6.65E-01	1	9	2348	21447	11.11%	10.95%	0.04%	0.04%	glycolysis Jan 31	Hk1
MP:0001919	abnormal reproductive system physiology	1.11E-01	1.00E+00	1.11E-01	1.11E-01	2.79E-01	3	11	2348	21447	27.27%	10.95%	0.13%	0.05%	shared jan 31	Eno4, Gapdhs, Pgk2
					1.00E+00	1.00E+00	0	19	278	21447	0.00%	1.30%	0.00%	0.09%	gluconeogenesis Jan 31	
					1.11E-01	1.91E-01	1	9	278	21447	11.11%	1.30%	0.36%	0.04%	glycolysis Jan 31	Hkdc1
MP:0003728	abnormal retina photoreceptor layer morphology	1.11E-01	1.00E+00	1.11E-01	1.00E+00	1.00E+00	0	11	278	21447	0.00%	1.30%	0.00%	0.05%	shared jan 31	

									1.11E-01	2.69E-01	2	19	647	21447	10.53%	3.02%	0.31%	0.09%	gluconeogenesis Jan 31	G6pc, Slc37a4
									1.00E+00	1.00E+00	0	9	647	21447	0.00%	3.02%	0.00%	0.04%	glycolysis Jan 31	
				MP:0012226	increased sterol level	1.11E-01	1.00E+00	1.11E-01	1.00E+00	1.00E+00	0	11	647	21447	0.00%	3.02%	0.00%	0.05%	shared jan 31	
									1.00E+00	1.00E+00	0	19	281	21447	0.00%	1.31%	0.00%	0.09%	gluconeogenesis Jan 31	
									1.12E-01	1.91E-01	1	9	281	21447	11.11%	1.31%	0.36%	0.04%	glycolysis Jan 31	Pfkm
				MP:0010578	abnormal heart left ventricle size	1.12E-01	1.00E+00	1.12E-01	1.00E+00	1.00E+00	0	11	281	21447	0.00%	1.31%	0.00%	0.05%	shared jan 31	
									2.53E-01	3.87E-01	4	19	2916	21447	21.05%	13.60%	0.14%	0.09%	gluconeogenesis Jan 31	Fbp2, Slc25a10, Slc37a1, Slc37a2
									1.12E-01	1.91E-01	3	9	2916	21447	33.33%	13.60%	0.10%	0.04%	glycolysis Jan 31	Hk3, Hkdc1, Pkm
				MP:0002092	abnormal eye morphology	1.12E-01	4.53E-01	2.48E-01	4.53E-01	5.46E-01	2	11	2916	21447	18.18%	13.60%	0.07%	0.05%	shared jan 31	Aldoa, Gapdh
									3.53E-01	4.61E-01	2	19	1395	21447	10.53%	6.50%	0.14%	0.09%	gluconeogenesis Jan 31	Pck2, Slc25a10
									1.12E-01	1.91E-01	2	9	1395	21447	22.22%	6.50%	0.14%	0.04%	glycolysis Jan 31	Gck, Pfkm
				MP:0005319	abnormal enzyme/coenzyme level	1.12E-01	1.00E+00	1.12E-01	1.00E+00	1.00E+00	0	11	1395	21447	0.00%	6.50%	0.00%	0.05%	shared jan 31	
									1.12E-01	2.70E-01	1	19	134	21447	5.26%	0.62%	0.75%	0.09%	gluconeogenesis Jan 31	Slc37a4
									1.00E+00	1.00E+00	0	9	134	21447	0.00%	0.62%	0.00%	0.04%	glycolysis Jan 31	
				MP:0011518	abnormal cell chemotaxis	1.12E-01	1.00E+00	1.12E-01	1.00E+00	1.00E+00	0	11	134	21447	0.00%	0.62%	0.00%	0.05%	shared jan 31	
									1.12E-01	2.70E-01	1	19	134	21447	5.26%	0.62%	0.75%	0.09%	gluconeogenesis Jan 31	Slc37a4
									1.00E+00	1.00E+00	0	9	134	21447	0.00%	0.62%	0.00%	0.04%	glycolysis Jan 31	
				MP:0000333	decreased bone marrow cell number	1.12E-01	1.00E+00	1.12E-01	1.00E+00	1.00E+00	0	11	134	21447	0.00%	0.62%	0.00%	0.05%	shared jan 31	
									1.00E+00	1.00E+00	0	19	284	21447	0.00%	1.32%	0.00%	0.09%	gluconeogenesis Jan 31	
									1.13E-01	1.91E-01	1	9	284	21447	11.11%	1.32%	0.35%	0.04%	glycolysis Jan 31	Gck
				MP:0011108	embryonic lethality during organogenesis, incomplete penetrance	1.13E-01	1.00E+00	1.13E-01	1.00E+00	1.00E+00	0	11	284	21447	0.00%	1.32%	0.00%	0.05%	shared jan 31	
									2.23E-01	3.67E-01	6	19	4703	21447	31.58%	21.93%	0.13%	0.09%	gluconeogenesis Jan 31	G6pc, G6pc3, Pck1, Pcx, Slc25a12, Slc37a4
									1.13E-01	1.91E-01	4	9	4703	21447	44.44%	21.93%	0.09%	0.04%	glycolysis Jan 31	Gck, Hk1, Hk2, Pfkm
				MP:0002089	abnormal postnatal growth/weight/body size	1.13E-01	4.46E-01	2.54E-01	4.46E-01	5.46E-01	3	11	4703	21447	27.27%	21.93%	0.06%	0.05%	shared jan 31	Aldob, Gpi1, Tpi1
									1.00E+00	1.00E+00	0	19	233	21447	0.00%	1.09%	0.00%	0.09%	gluconeogenesis Jan 31	
									1.00E+00	1.00E+00	0	9	233	21447	0.00%	1.09%	0.00%	0.04%	glycolysis Jan 31	
				MP:0005288	abnormal oxygen consumption	1.13E-01	1.00E+00	1.13E-01	1.13E-01	2.83E-01	1	11	233	21447	9.09%	1.09%	0.43%	0.05%	shared jan 31	Pgam1
									1.00E+00	1.00E+00	0	19	237	21447	0.00%	1.11%	0.00%	0.09%	gluconeogenesis Jan 31	
									1.00E+00	1.00E+00	0	9	237	21447	0.00%	1.11%	0.00%	0.04%	glycolysis Jan 31	
				MP:0005561	increased mean corpuscular hemoglobin	1.15E-01	1.00E+00	1.15E-01	1.15E-01	2.85E-01	1	11	237	21447	9.09%	1.11%	0.42%	0.05%	shared jan 31	Tpi1
									1.00E+00	1.00E+00	0	19	293	21447	0.00%	1.37%	0.00%	0.09%	gluconeogenesis Jan 31	
									1.16E-01	1.95E-01	1	9	293	21447	11.11%	1.37%	0.34%	0.04%	glycolysis Jan 31	Pfkm
				MP:0002059	abnormal seminal vesicle morphology	1.16E-01	1.00E+00	1.16E-01	1.00E+00	1.00E+00	0	11	293	21447	0.00%	1.37%	0.00%	0.05%	shared jan 31	
									1.17E-01	2.81E-01	3	19	1368	21447	15.79%	6.38%	0.22%	0.09%	gluconeogenesis Jan 31	Got2, Mdh2, Slc25a12
									1.00E+00	1.00E+00	0	9	1368	21447	0.00%	6.38%	0.00%	0.04%	glycolysis Jan 31	
				MP:0000783	abnormal forebrain morphology	1.17E-01	1.00E+00	1.17E-01	1.00E+00	1.00E+00	0	11	1368	21447	0.00%	6.38%	0.00%	0.05%	shared jan 31	
									1.00E+00	1.00E+00	0	19	298	21447	0.00%	1.39%	0.00%	0.09%	gluconeogenesis Jan 31	
									1.18E-01	1.97E-01	1	9	298	21447	11.11%	1.39%	0.34%	0.04%	glycolysis Jan 31	Pfkm
				MP:0001574	abnormal oxygen level	1.18E-01	1.00E+00	1.18E-01	1.00E+00	1.00E+00	0	11	298	21447	0.00%	1.39%	0.00%	0.05%	shared jan 31	
									1.19E-01	2.82E-01	1	19	142	21447	5.26%	0.66%	0.70%	0.09%	gluconeogenesis Jan 31	G6pc
									1.00E+00	1.00E+00	0	9	142	21447	0.00%	0.66%	0.00%	0.04%	glycolysis Jan 31	
				MP:0002705	dilated renal tubule	1.19E-01	1.00E+00	1.19E-01	1.00E+00	1.00E+00	0	11	142	21447	0.00%	0.66%	0.00%	0.05%	shared jan 31	
									1.00E+00	1.00E+00	0	19	300	21447	0.00%	1.40%	0.00%	0.09%	gluconeogenesis Jan 31	
									1.19E-01	1.97E-01	1	9	300	21447	11.11%	1.40%	0.33%	0.04%	glycolysis Jan 31	Pfkm
				MP:0003084	abnormal skeletal muscle fiber morphology	1.19E-01	1.00E+00	1.19E-01	1.00E+00	1.00E+00	0	11	300	21447	0.00%	1.40%	0.00%	0.05%	shared jan 31	
									1.00E+00	1.00E+00	0	19	246	21447	0.00%	1.15%	0.00%	0.09%	gluconeogenesis Jan 31	
									1.00E+00	1.00E+00	0	9	246	21447	0.00%	1.15%	0.00%	0.04%	glycolysis Jan 31	
				MP:0005351	decreased susceptibility to autoimmune disorder	1.19E-01	1.00E+00	1.19E-01	1.19E-01	2.89E-01	1	11	246	21447	9.09%	1.15%	0.41%	0.05%	shared jan 31	Pgam1
									1.19E-01	2.82E-01	1	19	143	21447	5.26%	0.67%	0.70%	0.09%	gluconeogenesis Jan 31	Slc25a10
									1.00E+00	1.00E+00	0	9	143	21447	0.00%	0.67%	0.00%	0.04%	glycolysis Jan 31	
				MP:0008804	abnormal circulating amylase level	1.19E-01	1.00E+00	1.19E-01	1.00E+00	1.00E+00	0	11	143	21447	0.00%	0.67%	0.00%	0.05%	shared jan 31	
									1.19E-01	2.82E-01	1	19	143	21447	5.26%	0.67%	0.70%	0.09%	gluconeogenesis Jan 31	Slc25a13
									1.00E+00	1.00E+00	0	9	143	21447	0.00%	0.67%	0.00%	0.04%	glycolysis Jan 31	
				MP:0001967	deafness	1.19E-01	1.00E+00	1.19E-01	1.00E+00	1.00E+00	0	11	143	21447	0.00%	0.67%	0.00%	0.05%	shared jan 31	
									1.33E-01	2.94E-01	3	19	1447	21447	15.79%	6.75%	0.21%	0.09%	gluconeogenesis Jan 31	Fbp2, Slc25a10, Slc37a2
									1.19E-01	1.97E-01	2	9	1447	21447	22.22%	6.75%	0.14%	0.04%	glycolysis Jan 31	Hkdc1, Pkm
				MP:0005195	abnormal posterior eye segment morphology	1.19E-01	1.67E-01	7.15E-01	1.67E-01	3.55E-01	2	11	1447	21447	18.18%	6.75%	0.14%	0.05%	shared jan 31	Aldoa, Gapdh
									1.20E-01	2.82E-01	1	19	144	21447	5.26%	0.67%	0.69%	0.09%	gluconeogenesis Jan 31	G6pc
									1.00E+00	1.00E+00	0	9	144	21447	0.00%	0.67%	0.00%	0.04%	glycolysis Jan 31	
				MP:0002828	abnormal renal glomerular capsule morphology	1.20E-01	1.00E+00	1.20E-01	1.00E+00	1.00E+00	0	11	144	21447	0.00%	0.67%	0.00%	0.05%	shared jan 31	
									2.38E-01	3.78E-01	1	19	305	21447	5.26%	1.42%	0.33%	0.09%	gluconeogenesis Jan 31	G6pc
									1.21E-01	1.98E-01	1	9	305	21447	11.11%	1.42%	0.33%	0.04%	glycolysis Jan 31	Pfkm
				MP:0000316	cellular necrosis	1.21E-01	1.00E+00	1.21E-01	1.00E+00	1.00E+00	0	11	305	21447	0.00%	1.42%	0.00%	0.05%	shared jan 31	
									1.22E-01	2.84E-01	3	19	1392	21447	15.79%	6.49%	0.22%	0.09%	gluconeogenesis Jan 31	Pck1, Slc25a12, Slc37a4
									4.53E-01	4.99E-01	1	9	1392	21447	11.11%	6.49%	0.07%	0.04%	glycolysis Jan 31	Gck
				MP:0002082	postnatal lethality	1.22E-01	5.22E-01	2.33E-01	5.22E-01	5.89E-01	1	11	1392	21447	9.09%	6.49%	0.07%	0.05%	shared jan 31	Gpi1
									1.23E-01	2.84E-01	1	19	147	21447	5.26%	0.69%	0.68%	0.09%	gluconeogenesis Jan 31	Pck1
									1.00E+00	1.00E+00	0	9	147	21447	0.00%	0.69%	0.00%	0.04%	glycolysis Jan 31	
				MP:0001438	aphagia	1.23E-01	1.00E+00	1.23E-01	1.00E+00	1.00E+00	0	11	147	21447	0.00%	0.69%	0.00%	0.05%	shared jan 31	
									1.00E+00	1.00E+00	0	19	311	21447	0.00%	1.45%	0.00%	0.09%	gluconeogenesis Jan 31	

					1.23E-01	2.00E-01	1	9	311	21447	11.11%	1.45%	0.32%	0.04%	glycolysis Jan 31	Hk1
MP:0010066	abnormal red blood cell distribution width	1.23E-01	1.00E+00	1.23E-01	1.00E+00	1.00E+00	0	11	311	21447	0.00%	1.45%	0.00%	0.05%	shared jan 31	
					4.34E-01	5.22E-01	5	19	4840	21447	26.32%	22.57%	0.10%	0.09%	gluconeogenesis Jan 31	G6pc, G6pc2, Pcx, Slc25a12, Slc37a4
					1.23E-01	2.00E-01	4	9	4840	21447	44.44%	22.57%	0.08%	0.04%	glycolysis Jan 31	Gck, Hk2, Hkdc1, Pfkm
MP:0005621	abnormal cell physiology	1.23E-01	4.67E-01	2.64E-01	4.67E-01	5.54E-01	3	11	4840	21447	27.27%	22.57%	0.06%	0.05%	shared jan 31	Eno4, Gapdhs, Pgk2
					1.23E-01	2.84E-01	4	19	2201	21447	21.05%	10.26%	0.18%	0.09%	gluconeogenesis Jan 31	G6pc, Pcx, Slc25a13, Slc37a4
					2.34E-01	3.03E-01	2	9	2201	21447	22.22%	10.26%	0.09%	0.04%	glycolysis Jan 31	Gck, Hk1
MP:0005367	renal/urinary system phenotype	1.23E-01	3.14E-01	3.93E-01	3.14E-01	4.69E-01	2	11	2201	21447	18.18%	10.26%	0.09%	0.05%	shared jan 31	Aldoc, Gapdh
					1.25E-01	2.86E-01	1	19	150	21447	5.26%	0.70%	0.67%	0.09%	gluconeogenesis Jan 31	Pck1
					1.00E+00	1.00E+00	0	9	150	21447	0.00%	0.70%	0.00%	0.04%	glycolysis Jan 31	
MP:0009117	abnormal white fat cell morphology	1.25E-01	1.00E+00	1.25E-01	1.00E+00	1.00E+00	0	11	150	21447	0.00%	0.70%	0.00%	0.05%	shared jan 31	
					2.46E-01	3.83E-01	1	19	316	21447	5.26%	1.47%	0.32%	0.09%	gluconeogenesis Jan 31	G6pc3
					1.25E-01	2.01E-01	1	9	316	21447	11.11%	1.47%	0.32%	0.04%	glycolysis Jan 31	Pklr
MP:0002412	increased susceptibility to bacterial infection	1.25E-01	1.00E+00	1.25E-01	1.00E+00	1.00E+00	0	11	316	21447	0.00%	1.47%	0.00%	0.05%	shared jan 31	
					2.47E-01	3.83E-01	1	19	317	21447	5.26%	1.48%	0.32%	0.09%	gluconeogenesis Jan 31	G6pc3
					1.25E-01	2.01E-01	1	9	317	21447	11.11%	1.48%	0.32%	0.04%	glycolysis Jan 31	Gck
MP:0005215	abnormal pancreatic islet morphology	1.25E-01	1.00E+00	1.25E-01	1.00E+00	1.00E+00	0	11	317	21447	0.00%	1.48%	0.00%	0.05%	shared jan 31	
					1.25E-01	2.86E-01	2	19	697	21447	10.53%	3.25%	0.29%	0.09%	gluconeogenesis Jan 31	Got2, Mdh2
					2.57E-01	3.24E-01	1	9	697	21447	11.11%	3.25%	0.14%	0.04%	glycolysis Jan 31	Hk2
MP:0003984	embryonic growth retardation	1.25E-01	1.00E+00	1.25E-01	1.00E+00	1.00E+00	0	11	697	21447	0.00%	3.25%	0.00%	0.05%	shared jan 31	
					1.26E-01	2.86E-01	3	19	1411	21447	15.79%	6.58%	0.21%	0.09%	gluconeogenesis Jan 31	G6pc3, Got1, Slc37a4
					1.00E+00	1.00E+00	0	9	1411	21447	0.00%	6.58%	0.00%	0.04%	glycolysis Jan 31	
MP:0008250	abnormal myeloid leukocyte morphology	1.26E-01	1.00E+00	1.26E-01	1.00E+00	1.00E+00	0	11	1411	21447	0.00%	6.58%	0.00%	0.05%	shared jan 31	
					2.47E-01	3.83E-01	1	19	318	21447	5.26%	1.48%	0.31%	0.09%	gluconeogenesis Jan 31	G6pc3
					1.26E-01	2.01E-01	1	9	318	21447	11.11%	1.48%	0.31%	0.04%	glycolysis Jan 31	Gck
MP:0009165	abnormal endocrine pancreas morphology	1.26E-01	1.00E+00	1.26E-01	1.00E+00	1.00E+00	0	11	318	21447	0.00%	1.48%	0.00%	0.05%	shared jan 31	
					1.00E+00	1.00E+00	0	19	319	21447	0.00%	1.49%	0.00%	0.09%	gluconeogenesis Jan 31	
					1.26E-01	2.01E-01	1	9	319	21447	11.11%	1.49%	0.31%	0.04%	glycolysis Jan 31	Gck
MP:0003141	cardiac fibrosis	1.26E-01	1.00E+00	1.26E-01	1.00E+00	1.00E+00	0	11	319	21447	0.00%	1.49%	0.00%	0.05%	shared jan 31	
					1.27E-01	2.89E-01	1	19	153	21447	5.26%	0.71%	0.65%	0.09%	gluconeogenesis Jan 31	Slc25a12
					1.00E+00	1.00E+00	0	9	153	21447	0.00%	0.71%	0.00%	0.04%	glycolysis Jan 31	
MP:0001505	hunched posture	1.27E-01	1.00E+00	1.27E-01	1.00E+00	1.00E+00	0	11	153	21447	0.00%	0.71%	0.00%	0.05%	shared jan 31	
					1.00E+00	1.00E+00	0	19	323	21447	0.00%	1.51%	0.00%	0.09%	gluconeogenesis Jan 31	
					1.28E-01	2.03E-01	1	9	323	21447	11.11%	1.51%	0.31%	0.04%	glycolysis Jan 31	Pklr
MP:0002409	decreased susceptibility to infection	1.28E-01	1.00E+00	1.28E-01	1.00E+00	1.00E+00	0	11	323	21447	0.00%	1.51%	0.00%	0.05%	shared jan 31	
					1.28E-01	2.89E-01	1	19	154	21447	5.26%	0.72%	0.65%	0.09%	gluconeogenesis Jan 31	Pck1
					1.00E+00	1.00E+00	0	9	154	21447	0.00%	0.72%	0.00%	0.04%	glycolysis Jan 31	
MP:0006319	abnormal epididymal fat pad morphology	1.28E-01	1.00E+00	1.28E-01	1.00E+00	1.00E+00	0	11	154	21447	0.00%	0.72%	0.00%	0.05%	shared jan 31	
					1.30E-01	2.92E-01	1	19	156	21447	5.26%	0.73%	0.64%	0.09%	gluconeogenesis Jan 31	Slc37a4
					1.00E+00	1.00E+00	0	9	156	21447	0.00%	0.73%	0.00%	0.04%	glycolysis Jan 31	
MP:0001601	abnormal myelopoiesis	1.30E-01	1.00E+00	1.30E-01	1.00E+00	1.00E+00	0	11	156	21447	0.00%	0.73%	0.00%	0.05%	shared jan 31	
					2.14E-01	3.57E-01	1	19	270	21447	5.26%	1.26%	0.37%	0.09%	gluconeogenesis Jan 31	Pck2
					1.00E+00	1.00E+00	0	9	270	21447	0.00%	1.26%	0.00%	0.04%	glycolysis Jan 31	
MP:0002631	abnormal epididymis morphology	1.30E-01	1.00E+00	1.30E-01	1.30E-01	3.10E-01	1	11	270	21447	9.09%	1.26%	0.37%	0.05%	shared jan 31	Eno4
					1.31E-01	2.92E-01	2	19	714	21447	10.53%	3.33%	0.28%	0.09%	gluconeogenesis Jan 31	Pck2, Slc37a2
					2.63E-01	3.28E-01	1	9	714	21447	11.11%	3.33%	0.14%	0.04%	glycolysis Jan 31	Hk3
MP:0002339	abnormal lymph node morphology	1.31E-01	1.00E+00	1.31E-01	1.00E+00	1.00E+00	0	11	714	21447	0.00%	3.33%	0.00%	0.05%	shared jan 31	
					1.00E+00	1.00E+00	0	19	331	21447	0.00%	1.54%	0.00%	0.09%	gluconeogenesis Jan 31	
					1.31E-01	2.07E-01	1	9	331	21447	11.11%	1.54%	0.30%	0.04%	glycolysis Jan 31	Pfkm
MP:0003960	increased lean body mass	1.31E-01	1.00E+00	1.31E-01	1.00E+00	1.00E+00	0	11	331	21447	0.00%	1.54%	0.00%	0.05%	shared jan 31	
					1.32E-01	2.94E-01	1	19	159	21447	5.26%	0.74%	0.63%	0.09%	gluconeogenesis Jan 31	Pck1
					1.00E+00	1.00E+00	0	9	159	21447	0.00%	0.74%	0.00%	0.04%	glycolysis Jan 31	
MP:0005666	abnormal adipose tissue physiology	1.32E-01	1.00E+00	1.32E-01	1.00E+00	1.00E+00	0	11	159	21447	0.00%	0.74%	0.00%	0.05%	shared jan 31	
					1.33E-01	2.95E-01	1	19	161	21447	5.26%	0.75%	0.62%	0.09%	gluconeogenesis Jan 31	Pck1
					1.00E+00	1.00E+00	0	9	161	21447	0.00%	0.75%	0.00%	0.04%	glycolysis Jan 31	
MP:0005202	lethargy	1.33E-01	1.00E+00	1.33E-01	1.00E+00	1.00E+00	0	11	161	21447	0.00%	0.75%	0.00%	0.05%	shared jan 31	
					1.00E+00	1.00E+00	0	19	278	21447	0.00%	1.30%	0.00%	0.09%	gluconeogenesis Jan 31	
					1.00E+00	1.00E+00	0	9	278	21447	0.00%	1.30%	0.00%	0.04%	glycolysis Jan 31	
MP:0008892	abnormal sperm flagellum morphology	1.34E-01	1.00E+00	1.34E-01	1.34E-01	3.12E-01	1	11	278	21447	9.09%	1.30%	0.36%	0.05%	shared jan 31	Eno4
					1.00E+00	1.00E+00	0	19	278	21447	0.00%	1.30%	0.00%	0.09%	gluconeogenesis Jan 31	
					1.00E+00	1.00E+00	0	9	278	21447	0.00%	1.30%	0.00%	0.04%	glycolysis Jan 31	
MP:0008770	decreased survivor rate	1.34E-01	1.00E+00	1.34E-01	1.34E-01	3.12E-01	1	11	278	21447	9.09%	1.30%	0.36%	0.05%	shared jan 31	Gapdh
					1.34E-01	2.95E-01	1	19	162	21447	5.26%	0.76%	0.62%	0.09%	gluconeogenesis Jan 31	Slc25a12
					1.00E+00	1.00E+00	0	9	162	21447	0.00%	0.76%	0.00%	0.04%	glycolysis Jan 31	
MP:0006007	abnormal basal ganglion morphology	1.34E-01	1.00E+00	1.34E-01	1.00E+00	1.00E+00	0	11	162	21447	0.00%	0.76%	0.00%	0.05%	shared jan 31	
					1.35E-01	2.95E-01	1	19	163	21447	5.26%	0.76%	0.61%	0.09%	gluconeogenesis Jan 31	Pck1
					1.00E+00	1.00E+00	0	9	163	21447	0.00%	0.76%	0.00%	0.04%	glycolysis Jan 31	
MP:0020222	abnormal alertness	1.35E-01	1.00E+00	1.35E-01	1.00E+00	1.00E+00	0	11	163	21447	0.00%	0.76%	0.00%	0.05%	shared jan 31	
					1.35E-01	2.95E-01	1	19	163	21447	5.26%	0.76%	0.61%	0.09%	gluconeogenesis Jan 31	Pck1
					1.00E+00	1.00E+00	0	9	163	21447	0.00%	0.76%	0.00%	0.04%	glycolysis Jan 31	

MP:0020224	decreased alertness	1.35E-01	1.00E+00	1.35E-01	1.00E+00	1.00E+00	0	11	163	21447	0.00%	0.76%	0.00%	0.05%	shared jan 31	
					1.00E+00	1.00E+00	0	19	344	21447	0.00%	1.60%	0.00%	0.09%	gluconeogenesis Jan 31	
					1.35E-01	2.13E-01	1	9	344	21447	11.11%	1.60%	0.29%	0.04%	glycolysis Jan 31	Pfkm
MP:0000601	small liver	1.35E-01	1.00E+00	1.35E-01	1.00E+00	1.00E+00	0	11	344	21447	0.00%	1.60%	0.00%	0.05%	shared jan 31	
					2.25E-01	3.67E-01	1	19	285	21447	5.26%	1.33%	0.35%	0.09%	gluconeogenesis Jan 31	G6pc
					1.00E+00	1.00E+00	0	9	285	21447	0.00%	1.33%	0.00%	0.04%	glycolysis Jan 31	
MP:0008986	abnormal liver parenchyma morphology	1.37E-01	1.00E+00	1.37E-01	1.37E-01	3.17E-01	1	11	285	21447	9.09%	1.33%	0.35%	0.05%	shared jan 31	Aldob
					1.37E-01	2.99E-01	1	19	166	21447	5.26%	0.77%	0.60%	0.09%	gluconeogenesis Jan 31	G6pc
					1.00E+00	1.00E+00	0	9	166	21447	0.00%	0.77%	0.00%	0.04%	glycolysis Jan 31	
MP:0008019	increased liver tumor incidence	1.37E-01	1.00E+00	1.37E-01	1.00E+00	1.00E+00	0	11	166	21447	0.00%	0.77%	0.00%	0.05%	shared jan 31	
					7.66E-01	7.79E-01	1	19	1577	21447	5.26%	7.35%	0.06%	0.09%	gluconeogenesis Jan 31	G6pc2
					1.38E-01	2.14E-01	2	9	1577	21447	22.22%	7.35%	0.13%	0.04%	glycolysis Jan 31	Gck, Hk2
MP:0000350	abnormal cell proliferation	1.38E-01	1.00E+00	1.38E-01	1.00E+00	1.00E+00	0	11	1577	21447	0.00%	7.35%	0.00%	0.05%	shared jan 31	
					1.00E+00	1.00E+00	0	19	351	21447	0.00%	1.64%	0.00%	0.09%	gluconeogenesis Jan 31	
					1.38E-01	2.14E-01	1	9	351	21447	11.11%	1.64%	0.28%	0.04%	glycolysis Jan 31	Gck
MP:0002079	increased circulating insulin level	1.38E-01	1.00E+00	1.38E-01	1.00E+00	1.00E+00	0	11	351	21447	0.00%	1.64%	0.00%	0.05%	shared jan 31	
					1.38E-01	2.99E-01	1	19	167	21447	5.26%	0.78%	0.60%	0.09%	gluconeogenesis Jan 31	G6pc
					1.00E+00	1.00E+00	0	9	167	21447	0.00%	0.78%	0.00%	0.04%	glycolysis Jan 31	
MP:0010297	increased hepatobiliary system tumor incidence	1.38E-01	1.00E+00	1.38E-01	1.00E+00	1.00E+00	0	11	167	21447	0.00%	0.78%	0.00%	0.05%	shared jan 31	
					1.39E-01	3.00E-01	3	19	1478	21447	15.79%	6.89%	0.20%	0.09%	gluconeogenesis Jan 31	G6pc2, Slc37a2, Slc37a4
					1.00E+00	1.00E+00	0	9	1478	21447	0.00%	6.89%	0.00%	0.04%	glycolysis Jan 31	
MP:0002723	abnormal immune serum protein physiology	1.39E-01	1.00E+00	1.39E-01	5.44E-01	5.99E-01	1	11	1478	21447	9.09%	6.89%	0.07%	0.05%	shared jan 31	Pgam1
					1.40E-01	3.01E-01	1	19	169	21447	5.26%	0.79%	0.59%	0.09%	gluconeogenesis Jan 31	Slc25a12
					1.00E+00	1.00E+00	0	9	169	21447	0.00%	0.79%	0.00%	0.04%	glycolysis Jan 31	
MP:0001525	impaired balance	1.40E-01	1.00E+00	1.40E-01	1.00E+00	1.00E+00	0	11	169	21447	0.00%	0.79%	0.00%	0.05%	shared jan 31	
					1.00E+00	1.00E+00	0	19	356	21447	0.00%	1.66%	0.00%	0.09%	gluconeogenesis Jan 31	
					1.40E-01	2.16E-01	1	9	356	21447	11.11%	1.66%	0.28%	0.04%	glycolysis Jan 31	Pfkl
MP:0030572	abnormal pilosebaceous unit morphology	1.40E-01	1.00E+00	1.40E-01	1.00E+00	1.00E+00	0	11	356	21447	0.00%	1.66%	0.00%	0.05%	shared jan 31	
					1.00E+00	1.00E+00	0	19	292	21447	0.00%	1.36%	0.00%	0.09%	gluconeogenesis Jan 31	
					1.00E+00	1.00E+00	0	9	292	21447	0.00%	1.36%	0.00%	0.04%	glycolysis Jan 31	
MP:0008079	decreased CD8-positive, alpha-beta T cell number	1.40E-01	1.00E+00	1.40E-01	1.40E-01	3.22E-01	1	11	292	21447	9.09%	1.36%	0.34%	0.05%	shared jan 31	Pgam1
					1.00E+00	1.00E+00	0	19	359	21447	0.00%	1.67%	0.00%	0.09%	gluconeogenesis Jan 31	
					1.41E-01	2.16E-01	1	9	359	21447	11.11%	1.67%	0.28%	0.04%	glycolysis Jan 31	Gck
MP:0011088	neonatal lethality, incomplete penetrance	1.41E-01	1.00E+00	1.41E-01	1.00E+00	1.00E+00	0	11	359	21447	0.00%	1.67%	0.00%	0.05%	shared jan 31	
					1.42E-01	3.04E-01	1	19	172	21447	5.26%	0.80%	0.58%	0.09%	gluconeogenesis Jan 31	Pcx
					1.00E+00	1.00E+00	0	9	172	21447	0.00%	0.80%	0.00%	0.04%	glycolysis Jan 31	
MP:0004129	abnormal respiratory quotient	1.42E-01	1.00E+00	1.42E-01	1.00E+00	1.00E+00	0	11	172	21447	0.00%	0.80%	0.00%	0.05%	shared jan 31	
					1.00E+00	1.00E+00	0	19	362	21447	0.00%	1.69%	0.00%	0.09%	gluconeogenesis Jan 31	
					1.42E-01	2.16E-01	1	9	362	21447	11.11%	1.69%	0.28%	0.04%	glycolysis Jan 31	Pfkm
MP:0013330	abnormal male accessory sex gland morphology	1.42E-01	1.00E+00	1.42E-01	1.00E+00	1.00E+00	0	11	362	21447	0.00%	1.69%	0.00%	0.05%	shared jan 31	
					1.00E+00	1.00E+00	0	19	362	21447	0.00%	1.69%	0.00%	0.09%	gluconeogenesis Jan 31	
					1.42E-01	2.16E-01	1	9	362	21447	11.11%	1.69%	0.28%	0.04%	glycolysis Jan 31	Pfkm
MP:0008772	increased heart ventricle size	1.42E-01	1.00E+00	1.42E-01	1.00E+00	1.00E+00	0	11	362	21447	0.00%	1.69%	0.00%	0.05%	shared jan 31	
					3.19E-01	4.42E-01	4	19	3234	21447	21.05%	15.08%	0.12%	0.09%	gluconeogenesis Jan 31	Fbp2, Slc25a10, Slc37a1, Slc37a2
					1.43E-01	2.16E-01	3	9	3234	21447	33.33%	15.08%	0.09%	0.04%	glycolysis Jan 31	Hk3, Hkdc1, Pkm
MP:0005391	vision/eye phenotype	1.43E-01	5.11E-01	2.79E-01	5.11E-01	5.79E-01	2	11	3234	21447	18.18%	15.08%	0.06%	0.05%	shared jan 31	Aldoa, Gapdh
					1.44E-01	3.08E-01	2	19	759	21447	10.53%	3.54%	0.26%	0.09%	gluconeogenesis Jan 31	Fbp2, Slc37a2
					2.77E-01	3.39E-01	1	9	759	21447	11.11%	3.54%	0.13%	0.04%	glycolysis Jan 31	Hk3
MP:0001303	abnormal lens morphology	1.44E-01	3.27E-01	4.40E-01	3.27E-01	4.78E-01	1	11	759	21447	9.09%	3.54%	0.13%	0.05%	shared jan 31	Gapdh
					2.26E-01	3.68E-01	7	19	5741	21447	36.84%	26.77%	0.12%	0.09%	gluconeogenesis Jan 31	Fbp2, G6pc2, G6pc3, Got1, Got2, Slc37a2, Slc37a4
					2.00E-01	2.76E-01	4	9	5741	21447	44.44%	26.77%	0.07%	0.04%	glycolysis Jan 31	Gck, Hk1, Pfkm, Pklr
MP:0005397	hematopoietic system phenotype	1.45E-01	2.26E-01	6.41E-01	1.45E-01	3.26E-01	5	11	5741	21447	45.45%	26.77%	0.09%	0.05%	shared jan 31	Aldoa, Gapdh, Gpi1, Pgam1, Tpi1
					1.45E-01	3.08E-01	1	19	176	21447	5.26%	0.82%	0.57%	0.09%	gluconeogenesis Jan 31	Slc25a12
					1.00E+00	1.00E+00	0	9	176	21447	0.00%	0.82%	0.00%	0.04%	glycolysis Jan 31	
MP:0009748	abnormal behavioral response to addictive substance	1.45E-01	1.00E+00	1.45E-01	1.00E+00	1.00E+00	0	11	176	21447	0.00%	0.82%	0.00%	0.05%	shared jan 31	
					1.47E-01	3.10E-01	1	19	178	21447	5.26%	0.83%	0.56%	0.09%	gluconeogenesis Jan 31	G6pc2
					1.00E+00	1.00E+00	0	9	178	21447	0.00%	0.83%	0.00%	0.04%	glycolysis Jan 31	
MP:0008567	decreased interferon-gamma secretion	1.47E-01	1.00E+00	1.47E-01	1.00E+00	1.00E+00	0	11	178	21447	0.00%	0.83%	0.00%	0.05%	shared jan 31	
					1.48E-01	3.11E-01	2	19	771	21447	10.53%	3.59%	0.26%	0.09%	gluconeogenesis Jan 31	Got1, Slc37a4
					2.81E-01	3.43E-01	1	9	771	21447	11.11%	3.59%	0.13%	0.04%	glycolysis Jan 31	Pklr
MP:0002398	abnormal bone marrow cell morphology/development	1.48E-01	1.00E+00	1.48E-01	1.00E+00	1.00E+00	0	11	771	21447	0.00%	3.59%	0.00%	0.05%	shared jan 31	
					1.48E-01	3.11E-01	8	19	6144	21447	42.11%	28.65%	0.13%	0.09%	gluconeogenesis Jan 31	Fbp2, G6pc2, G6pc3, Got1, Got2, Pck2, Slc37a2, Slc37a4
					2.40E-01	3.09E-01	4	9	6144	21447	44.44%	28.65%	0.07%	0.04%	glycolysis Jan 31	Hk1, Hk3, Pfkm, Pklr
MP:0005387	immune system phenotype	1.48E-01	2.40E-01	6.18E-01	1.81E-01	3.73E-01	5	11	6144	21447	45.45%	28.65%	0.08%	0.05%	shared jan 31	Aldoa, Aldob, Gpi1, Pgam1, Tpi1
					1.50E-01	3.14E-01	1	19	183	21447	5.26%	0.85%	0.55%	0.09%	gluconeogenesis Jan 31	Slc25a12
					1.00E+00	1.00E+00	0	9	183	21447	0.00%	0.85%	0.00%	0.04%	glycolysis Jan 31	
MP:0011479	abnormal catecholamine level	1.50E-01	1.00E+00	1.50E-01	1.00E+00	1.00E+00	0	11	183	21447	0.00%	0.85%	0.00%	0.05%	shared jan 31	
					1.51E-01	3.15E-01	1	19	184	21447	5.26%	0.86%	0.54%	0.09%	gluconeogenesis Jan 31	Fbp2
					1.00E+00	1.00E+00	0	9	184	21447	0.00%	0.86%	0.00%	0.04%	glycolysis Jan 31	

				MP:0001529	abnormal vocalization	1.51E-01	1.00E+00	1.51E-01	1.00E+00	1.00E+00	0	11	184	21447	0.00%	0.86%	0.00%	0.05%	shared jan 31	
									2.47E-01	3.83E-01	1	19	318	21447	5.26%	1.48%	0.31%	0.09%	gluconeogenesis Jan 31	Slc37a4
									1.00E+00	1.00E+00	0	9	318	21447	0.00%	1.48%	0.00%	0.04%	glycolysis Jan 31	
				MP:0001876	decreased inflammatory response	1.52E-01	1.00E+00	1.52E-01	1.52E-01	3.34E-01	1	11	318	21447	9.09%	1.48%	0.31%	0.05%	shared jan 31	Pgam1
									1.52E-01	3.15E-01	1	19	185	21447	5.26%	0.86%	0.54%	0.09%	gluconeogenesis Jan 31	Slc25a12
									1.00E+00	1.00E+00	0	9	185	21447	0.00%	0.86%	0.00%	0.04%	glycolysis Jan 31	
				MP:0003674	oxidative stress	1.52E-01	1.00E+00	1.52E-01	1.00E+00	1.00E+00	0	11	185	21447	0.00%	0.86%	0.00%	0.05%	shared jan 31	
									2.48E-01	3.83E-01	1	19	319	21447	5.26%	1.49%	0.31%	0.09%	gluconeogenesis Jan 31	Slc37a2
									1.00E+00	1.00E+00	0	9	319	21447	0.00%	1.49%	0.00%	0.04%	glycolysis Jan 31	
				MP:0002699	abnormal vitreous body morphology	1.52E-01	1.00E+00	1.52E-01	1.52E-01	3.34E-01	1	11	319	21447	9.09%	1.49%	0.31%	0.05%	shared jan 31	Gapdh
									2.94E-01	4.22E-01	1	19	390	21447	5.26%	1.82%	0.26%	0.09%	gluconeogenesis Jan 31	Got1
									1.52E-01	2.28E-01	1	9	390	21447	11.11%	1.82%	0.26%	0.04%	glycolysis Jan 31	Gck
				MP:0010508	abnormal heart electrocardiography waveform feature	1.52E-01	1.00E+00	1.52E-01	1.00E+00	1.00E+00	0	11	390	21447	0.00%	1.82%	0.00%	0.05%	shared jan 31	
									8.19E-01	8.28E-01	2	19	3333	21447	10.53%	15.54%	0.06%	0.09%	gluconeogenesis Jan 31	G6pc3, Slc37a2
									1.53E-01	2.28E-01	3	9	3333	21447	33.33%	15.54%	0.09%	0.04%	glycolysis Jan 31	Gck, Hk3, Pfkm
				MP:0002163	abnormal gland morphology	1.53E-01	8.44E-01	1.81E-01	8.44E-01	8.63E-01	1	11	3333	21447	9.09%	15.54%	0.03%	0.05%	shared jan 31	Eno4
									1.53E-01	3.15E-01	1	19	186	21447	5.26%	0.87%	0.54%	0.09%	gluconeogenesis Jan 31	Slc37a2
									1.00E+00	1.00E+00	0	9	186	21447	0.00%	0.87%	0.00%	0.04%	glycolysis Jan 31	
				MP:0005088	increased acute inflammation	1.53E-01	1.00E+00	1.53E-01	1.00E+00	1.00E+00	0	11	186	21447	0.00%	0.87%	0.00%	0.05%	shared jan 31	
									1.00E+00	1.00E+00	0	19	321	21447	0.00%	1.50%	0.00%	0.09%	gluconeogenesis Jan 31	
									1.00E+00	1.00E+00	0	9	321	21447	0.00%	1.50%	0.00%	0.04%	glycolysis Jan 31	
				MP:0008075	decreased CD4-positive, alpha beta T cell number	1.53E-01	1.00E+00	1.53E-01	1.53E-01	3.34E-01	1	11	321	21447	9.09%	1.50%	0.31%	0.05%	shared jan 31	Pgam1
									1.55E-01	3.16E-01	3	19	1553	21447	15.79%	7.24%	0.19%	0.09%	gluconeogenesis Jan 31	G6pc, Pcx, Slc37a4
									4.92E-01	5.30E-01	1	9	1553	21447	11.11%	7.24%	0.06%	0.04%	glycolysis Jan 31	Hk1
				MP:0002135	abnormal kidney morphology	1.55E-01	5.63E-01	2.75E-01	5.63E-01	6.15E-01	1	11	1553	21447	9.09%	7.24%	0.06%	0.05%	shared jan 31	Aldoc
									1.56E-01	3.16E-01	3	19	1559	21447	15.79%	7.27%	0.19%	0.09%	gluconeogenesis Jan 31	G6pc3, Got1, Slc37a4
									1.00E+00	1.00E+00	0	9	1559	21447	0.00%	7.27%	0.00%	0.04%	glycolysis Jan 31	
				MP:0000221	decreased leukocyte cell number	1.56E-01	1.00E+00	1.56E-01	1.88E-01	3.77E-01	2	11	1559	21447	18.18%	7.27%	0.13%	0.05%	shared jan 31	Aldoa, Pgam1
									3.49E-01	4.59E-01	4	19	3373	21447	21.05%	15.73%	0.12%	0.09%	gluconeogenesis Jan 31	G6pc3, Pcx, Slc25a12, Slc37a4
									1.57E-01	2.34E-01	3	9	3373	21447	33.33%	15.73%	0.09%	0.04%	glycolysis Jan 31	Gck, Hk2, Pfkm
				MP:0005451	abnormal body composition	1.57E-01	5.35E-01	2.93E-01	5.35E-01	5.98E-01	2	11	3373	21447	18.18%	15.73%	0.06%	0.05%	shared jan 31	Gpi1, Tpi1
									1.57E-01	3.16E-01	1	19	192	21447	5.26%	0.90%	0.52%	0.09%	gluconeogenesis Jan 31	Slc25a12
									1.00E+00	1.00E+00	0	9	192	21447	0.00%	0.90%	0.00%	0.04%	glycolysis Jan 31	
				MP:0000947	convulsive seizures	1.57E-01	1.00E+00	1.57E-01	1.00E+00	1.00E+00	0	11	192	21447	0.00%	0.90%	0.00%	0.05%	shared jan 31	
									1.58E-01	3.16E-01	1	19	193	21447	5.26%	0.90%	0.52%	0.09%	gluconeogenesis Jan 31	G6pc
									1.00E+00	1.00E+00	0	9	193	21447	0.00%	0.90%	0.00%	0.04%	glycolysis Jan 31	
				MP:0020189	abnormal adenoma incidence	1.58E-01	1.00E+00	1.58E-01	1.00E+00	1.00E+00	0	11	193	21447	0.00%	0.90%	0.00%	0.05%	shared jan 31	
									1.58E-01	3.16E-01	1	19	193	21447	5.26%	0.90%	0.52%	0.09%	gluconeogenesis Jan 31	G6pc
									1.00E+00	1.00E+00	0	9	193	21447	0.00%	0.90%	0.00%	0.04%	glycolysis Jan 31	
				MP:0010383	increased adenoma incidence	1.58E-01	1.00E+00	1.58E-01	1.00E+00	1.00E+00	0	11	193	21447	0.00%	0.90%	0.00%	0.05%	shared jan 31	
									1.59E-01	3.16E-01	3	19	1573	21447	15.79%	7.33%	0.19%	0.09%	gluconeogenesis Jan 31	Pck1, Pcx, Slc37a1
									4.96E-01	5.33E-01	1	9	1573	21447	11.11%	7.33%	0.06%	0.04%	glycolysis Jan 31	Pfkm
				MP:0000003	abnormal adipose tissue morphology	1.59E-01	1.00E+00	1.59E-01	1.00E+00	1.00E+00	0	11	1573	21447	0.00%	7.33%	0.00%	0.05%	shared jan 31	
									1.59E-01	3.16E-01	1	19	195	21447	5.26%	0.91%	0.51%	0.09%	gluconeogenesis Jan 31	G6pc2
									1.00E+00	1.00E+00	0	9	195	21447	0.00%	0.91%	0.00%	0.04%	glycolysis Jan 31	
				MP:0005463	abnormal CD4-positive, alpha-beta T cell physiology	1.59E-01	1.00E+00	1.59E-01	1.00E+00	1.00E+00	0	11	195	21447	0.00%	0.91%	0.00%	0.05%	shared jan 31	
									1.60E-01	3.16E-01	2	19	810	21447	10.53%	3.78%	0.25%	0.09%	gluconeogenesis Jan 31	Got2, Mdh2
									2.93E-01	3.54E-01	1	9	810	21447	11.11%	3.78%	0.12%	0.04%	glycolysis Jan 31	Hk2
				MP:0010865	prenatal growth retardation	1.60E-01	1.00E+00	1.60E-01	1.00E+00	1.00E+00	0	11	810	21447	0.00%	3.78%	0.00%	0.05%	shared jan 31	
									1.60E-01	3.16E-01	1	19	196	21447	5.26%	0.91%	0.51%	0.09%	gluconeogenesis Jan 31	Pck1
									1.00E+00	1.00E+00	0	9	196	21447	0.00%	0.91%	0.00%	0.04%	glycolysis Jan 31	
				MP:0001781	abnormal white adipose tissue amount	1.60E-01	1.00E+00	1.60E-01	1.00E+00	1.00E+00	0	11	196	21447	0.00%	0.91%	0.00%	0.05%	shared jan 31	
									1.60E-01	3.16E-01	1	19	196	21447	5.26%	0.91%	0.51%	0.09%	gluconeogenesis Jan 31	Slc25a13
									1.00E+00	1.00E+00	0	9	196	21447	0.00%	0.91%	0.00%	0.04%	glycolysis Jan 31	
				MP:0001394	circling	1.60E-01	1.00E+00	1.60E-01	1.00E+00	1.00E+00	0	11	196	21447	0.00%	0.91%	0.00%	0.05%	shared jan 31	
									3.09E-01	4.36E-01	1	19	413	21447	5.26%	1.93%	0.24%	0.09%	gluconeogenesis Jan 31	G6pc3
									1.61E-01	2.39E-01	1	9	413	21447	11.11%	1.93%	0.24%	0.04%	glycolysis Jan 31	Pklr
				MP:0020186	abnormal susceptibility to bacterial infection	1.61E-01	1.00E+00	1.61E-01	1.00E+00	1.00E+00	0	11	413	21447	0.00%	1.93%	0.00%	0.05%	shared jan 31	
									1.61E-01	3.16E-01	1	19	197	21447	5.26%	0.92%	0.51%	0.09%	gluconeogenesis Jan 31	Slc25a12
									1.00E+00	1.00E+00	0	9	197	21447	0.00%	0.92%	0.00%	0.04%	glycolysis Jan 31	
				MP:0010954	abnormal cellular respiration	1.61E-01	1.00E+00	1.61E-01	1.00E+00	1.00E+00	0	11	197	21447	0.00%	0.92%	0.00%	0.05%	shared jan 31	
									1.62E-01	3.17E-01	1	19	198	21447	5.26%	0.92%	0.51%	0.09%	gluconeogenesis Jan 31	Pck1
									1.00E+00	1.00E+00	0	9	198	21447	0.00%	0.92%	0.00%	0.04%	glycolysis Jan 31	
				MP:0002971	abnormal brown adipose tissue morphology	1.62E-01	1.00E+00	1.62E-01	1.00E+00	1.00E+00	0	11	198	21447	0.00%	0.92%	0.00%	0.05%	shared jan 31	
									1.00E+00	1.00E+00	0	19	341	21447	0.00%	1.59%	0.00%	0.09%	gluconeogenesis Jan 31	
									1.00E+00	1.00E+00	0	9	341	21447	0.00%	1.59%	0.00%	0.04%	glycolysis Jan 31	
				MP:0001730	embryonic growth arrest	1.62E-01	1.00E+00	1.62E-01	1.62E-01	3.46E-01	1	11	341	21447	9.09%	1.59%	0.29%	0.05%	shared jan 31	Eno1
									1.62E-01	3.17E-01	1	19	199	21447	5.26%	0.93%	0.50%	0.09%	gluconeogenesis Jan 31	Slc25a13
									1.00E+00	1.00E+00	0	9	199	21447	0.00%	0.93%	0.00%	0.04%	glycolysis Jan 31	
				MP:0000436	abnormal head movements	1.62E-01	1.00E+00	1.62E-01	1.00E+00	1.00E+00	0	11	199	21447	0.00%	0.93%	0.00%	0.05%	shared jan 31	

					1.00E+00	1.00E+00	0	19	419	21447	0.00%	1.95%	0.00%	0.09%	gluconeogenesis Jan 31	
					1.63E-01	2.41E-01	1	9	419	21447	11.11%	1.95%	0.24%	0.04%	glycolysis Jan 31	Pfkm
MP:0000260	abnormal angiogenesis	1.63E-01	1.00E+00	1.63E-01	1.00E+00	1.00E+00	0	11	419	21447	0.00%	1.95%	0.00%	0.05%	shared jan 31	
					1.63E-01	3.17E-01	1	19	200	21447	5.26%	0.93%	0.50%	0.09%	gluconeogenesis Jan 31	Slc25a12
					1.00E+00	1.00E+00	0	9	200	21447	0.00%	0.93%	0.00%	0.04%	glycolysis Jan 31	
MP:0011380	enlarged brain ventricles	1.63E-01	1.00E+00	1.63E-01	1.00E+00	1.00E+00	0	11	200	21447	0.00%	0.93%	0.00%	0.05%	shared jan 31	
					3.15E-01	4.42E-01	1	19	423	21447	5.26%	1.97%	0.24%	0.09%	gluconeogenesis Jan 31	Got1
					1.64E-01	2.42E-01	1	9	423	21447	11.11%	1.97%	0.24%	0.04%	glycolysis Jan 31	Gck
MP:0003137	abnormal impulse conducting system conduction	1.64E-01	1.00E+00	1.64E-01	1.00E+00	1.00E+00	0	11	423	21447	0.00%	1.97%	0.00%	0.05%	shared jan 31	
					1.65E-01	3.17E-01	1	19	202	21447	5.26%	0.94%	0.50%	0.09%	gluconeogenesis Jan 31	Slc37a2
					1.00E+00	1.00E+00	0	9	202	21447	0.00%	0.94%	0.00%	0.04%	glycolysis Jan 31	
MP:0008704	abnormal interleukin-6 secretion	1.65E-01	1.00E+00	1.65E-01	1.00E+00	1.00E+00	0	11	202	21447	0.00%	0.94%	0.00%	0.05%	shared jan 31	
					1.65E-01	3.17E-01	1	19	202	21447	5.26%	0.94%	0.50%	0.09%	gluconeogenesis Jan 31	Pck1
					1.00E+00	1.00E+00	0	9	202	21447	0.00%	0.94%	0.00%	0.04%	glycolysis Jan 31	
MP:0005457	abnormal percent body fat/body weight	1.65E-01	1.00E+00	1.65E-01	1.00E+00	1.00E+00	0	11	202	21447	0.00%	0.94%	0.00%	0.05%	shared jan 31	
					1.00E+00	1.00E+00	0	19	427	21447	0.00%	1.99%	0.00%	0.09%	gluconeogenesis Jan 31	
					1.66E-01	2.43E-01	1	9	427	21447	11.11%	1.99%	0.23%	0.04%	glycolysis Jan 31	Pfkm
MP:0001625	cardiac hypertrophy	1.66E-01	1.00E+00	1.66E-01	1.00E+00	1.00E+00	0	11	427	21447	0.00%	1.99%	0.00%	0.05%	shared jan 31	
					1.66E-01	3.18E-01	1	19	204	21447	5.26%	0.95%	0.49%	0.09%	gluconeogenesis Jan 31	G6pc
					1.00E+00	1.00E+00	0	9	204	21447	0.00%	0.95%	0.00%	0.04%	glycolysis Jan 31	
MP:0000607	abnormal hepatocyte morphology	1.66E-01	1.00E+00	1.66E-01	1.00E+00	1.00E+00	0	11	204	21447	0.00%	0.95%	0.00%	0.05%	shared jan 31	
					1.00E+00	1.00E+00	0	19	355	21447	0.00%	1.66%	0.00%	0.09%	gluconeogenesis Jan 31	
					1.00E+00	1.00E+00	0	9	355	21447	0.00%	1.66%	0.00%	0.04%	glycolysis Jan 31	
MP:0013206	abnormal motile cilium morphology	1.68E-01	1.00E+00	1.68E-01	1.68E-01	3.55E-01	1	11	355	21447	9.09%	1.66%	0.28%	0.05%	shared jan 31	Eno4
					1.68E-01	3.21E-01	1	19	207	21447	5.26%	0.97%	0.48%	0.09%	gluconeogenesis Jan 31	Slc25a12
					1.00E+00	1.00E+00	0	9	207	21447	0.00%	0.97%	0.00%	0.04%	glycolysis Jan 31	
MP:0003186	abnormal redox activity	1.68E-01	1.00E+00	1.68E-01	1.00E+00	1.00E+00	0	11	207	21447	0.00%	0.97%	0.00%	0.05%	shared jan 31	
					1.70E-01	3.21E-01	1	19	209	21447	5.26%	0.97%	0.48%	0.09%	gluconeogenesis Jan 31	Slc25a12
					1.00E+00	1.00E+00	0	9	209	21447	0.00%	0.97%	0.00%	0.04%	glycolysis Jan 31	
MP:0013229	abnormal brain ventricle size	1.70E-01	1.00E+00	1.70E-01	1.00E+00	1.00E+00	0	11	209	21447	0.00%	0.97%	0.00%	0.05%	shared jan 31	
					1.70E-01	3.21E-01	1	19	209	21447	5.26%	0.97%	0.48%	0.09%	gluconeogenesis Jan 31	Slc25a12
					1.00E+00	1.00E+00	0	9	209	21447	0.00%	0.97%	0.00%	0.04%	glycolysis Jan 31	
MP:0000823	abnormal lateral ventricle morphology	1.70E-01	1.00E+00	1.70E-01	1.00E+00	1.00E+00	0	11	209	21447	0.00%	0.97%	0.00%	0.05%	shared jan 31	
					1.70E-01	3.21E-01	2	19	842	21447	10.53%	3.93%	0.24%	0.09%	gluconeogenesis Jan 31	Got2, Mdh2
					1.00E+00	1.00E+00	0	9	842	21447	0.00%	3.93%	0.00%	0.04%	glycolysis Jan 31	
MP:0002086	abnormal extraembryonic tissue morphology	1.70E-01	1.00E+00	1.70E-01	1.00E+00	1.00E+00	0	11	842	21447	0.00%	3.93%	0.00%	0.05%	shared jan 31	
					1.70E-01	3.21E-01	3	19	1624	21447	15.79%	7.57%	0.18%	0.09%	gluconeogenesis Jan 31	Pck1, Pcx, Slc37a1
					5.08E-01	5.42E-01	1	9	1624	21447	11.11%	7.57%	0.06%	0.04%	glycolysis Jan 31	Pfkm
MP:0005375	adipose tissue phenotype	1.70E-01	1.00E+00	1.70E-01	1.00E+00	1.00E+00	0	11	1624	21447	0.00%	7.57%	0.00%	0.05%	shared jan 31	
					1.71E-01	3.22E-01	4	19	2489	21447	21.05%	11.61%	0.16%	0.09%	gluconeogenesis Jan 31	Fbp2, G6pc3, Got1, Slc37a4
					1.00E+00	1.00E+00	0	9	2489	21447	0.00%	11.61%	0.00%	0.04%	glycolysis Jan 31	
MP:0000217	abnormal leukocyte cell number	1.71E-01	1.00E+00	1.71E-01	3.71E-01	4.99E-01	2	11	2489	21447	18.18%	11.61%	0.08%	0.05%	shared jan 31	Aldoa, Pgam1
					3.28E-01	4.47E-01	1	19	444	21447	5.26%	2.07%	0.23%	0.09%	gluconeogenesis Jan 31	Mdh1
					1.72E-01	2.51E-01	1	9	444	21447	11.11%	2.07%	0.23%	0.04%	glycolysis Jan 31	Pkm
MP:0011091	prenatal lethality, complete penetrance	1.72E-01	3.28E-01	5.23E-01	2.06E-01	3.90E-01	1	11	444	21447	9.09%	2.07%	0.23%	0.05%	shared jan 31	Gpi1
					3.32E-01	4.50E-01	1	19	450	21447	5.26%	2.10%	0.22%	0.09%	gluconeogenesis Jan 31	Pcx
					1.74E-01	2.53E-01	1	9	450	21447	11.11%	2.10%	0.22%	0.04%	glycolysis Jan 31	Gck
MP:0013277	abnormal fasting circulating glucose level	1.74E-01	1.00E+00	1.74E-01	1.00E+00	1.00E+00	0	11	450	21447	0.00%	2.10%	0.00%	0.05%	shared jan 31	
					1.74E-01	3.26E-01	1	19	215	21447	5.26%	1.00%	0.47%	0.09%	gluconeogenesis Jan 31	Slc37a4
					1.00E+00	1.00E+00	0	9	215	21447	0.00%	1.00%	0.00%	0.04%	glycolysis Jan 31	
MP:0000172	abnormal bone marrow cell number	1.74E-01	1.00E+00	1.74E-01	1.00E+00	1.00E+00	0	11	215	21447	0.00%	1.00%	0.00%	0.05%	shared jan 31	
					1.74E-01	3.26E-01	2	19	856	21447	10.53%	3.99%	0.23%	0.09%	gluconeogenesis Jan 31	G6pc, Slc37a4
					1.00E+00	1.00E+00	0	9	856	21447	0.00%	3.99%	0.00%	0.04%	glycolysis Jan 31	
MP:0003723	abnormal long bone morphology	1.74E-01	1.00E+00	1.74E-01	1.00E+00	1.00E+00	0	11	856	21447	0.00%	3.99%	0.00%	0.05%	shared jan 31	
					1.00E+00	1.00E+00	0	19	455	21447	0.00%	2.12%	0.00%	0.09%	gluconeogenesis Jan 31	
					1.76E-01	2.54E-01	1	9	455	21447	11.11%	2.12%	0.22%	0.04%	glycolysis Jan 31	Hk1
MP:0001926	female infertility	1.76E-01	1.00E+00	1.76E-01	1.00E+00	1.00E+00	0	11	455	21447	0.00%	2.12%	0.00%	0.05%	shared jan 31	
					1.77E-01	3.26E-01	1	19	218	21447	5.26%	1.02%	0.46%	0.09%	gluconeogenesis Jan 31	Slc25a13
					1.00E+00	1.00E+00	0	9	218	21447	0.00%	1.02%	0.00%	0.04%	glycolysis Jan 31	
MP:0002622	abnormal cochlear hair cell morphology	1.77E-01	1.00E+00	1.77E-01	1.00E+00	1.00E+00	0	11	218	21447	0.00%	1.02%	0.00%	0.05%	shared jan 31	
					1.77E-01	3.26E-01	2	19	864	21447	10.53%	4.03%	0.23%	0.09%	gluconeogenesis Jan 31	Got2, Mdh2
					3.09E-01	3.69E-01	1	9	864	21447	11.11%	4.03%	0.12%	0.04%	glycolysis Jan 31	Pfkm
MP:0002925	abnormal cardiovascular development	1.77E-01	1.00E+00	1.77E-01	1.00E+00	1.00E+00	0	11	864	21447	0.00%	4.03%	0.00%	0.05%	shared jan 31	
					1.77E-01	3.26E-01	1	19	219	21447	5.26%	1.02%	0.46%	0.09%	gluconeogenesis Jan 31	G6pc2
					1.00E+00	1.00E+00	0	9	219	21447	0.00%	1.02%	0.00%	0.04%	glycolysis Jan 31	
MP:0005095	decreased T cell proliferation	1.77E-01	1.00E+00	1.77E-01	1.00E+00	1.00E+00	0	11	219	21447	0.00%	1.02%	0.00%	0.05%	shared jan 31	
					2.91E-01	4.22E-01	1	19	385	21447	5.26%	1.80%	0.26%	0.09%	gluconeogenesis Jan 31	Slc37a2
					1.00E+00	1.00E+00	0	9	385	21447	0.00%	1.80%	0.00%	0.04%	glycolysis Jan 31	
MP:0002498	abnormal acute inflammation	1.81E-01	1.00E+00	1.81E-01	1.81E-01	3.73E-01	1	11	385	21447	9.09%	1.80%	0.26%	0.05%	shared jan 31	Pgam1
					1.81E-01	3.31E-01	3	19	1673	21447	15.79%	7.80%	0.18%	0.09%	gluconeogenesis Jan 31	Got2, Mdh1, Mdh2

									1.00E+00	1.00E+00	0	9	1673	21447	0.00%	7.80%	0.00%	0.04%	glycolysis Jan 31	
				MP:0014134	abnormal embryo morphology	1.81E-01	1.00E+00	1.81E-01	2.10E-01	3.91E-01	2	11	1673	21447	18.18%	7.80%	0.12%	0.05%	shared jan 31	Eno1, Gpi1
									5.67E-01	6.20E-01	5	19	5535	21447	26.32%	25.81%	0.09%	0.09%	gluconeogenesis Jan 31	G6pc, G6pc2, Pcx, Slc25a12, Slc37a4
									1.81E-01	2.60E-01	4	9	5535	21447	44.44%	25.81%	0.07%	0.04%	glycolysis Jan 31	Gck, Hk2, Hkdc1, Pfkm
				MP:0005384	cellular phenotype	1.81E-01	5.70E-01	3.18E-01	5.70E-01	6.17E-01	3	11	5535	21447	27.27%	25.81%	0.05%	0.05%	shared jan 31	Eno4, Gapdhs, Pgk2
									1.00E+00	1.00E+00	0	19	389	21447	0.00%	1.81%	0.00%	0.09%	gluconeogenesis Jan 31	
									1.00E+00	1.00E+00	0	9	389	21447	0.00%	1.81%	0.00%	0.04%	glycolysis Jan 31	
				MP:0001922	reduced male fertility	1.82E-01	1.00E+00	1.82E-01	1.82E-01	3.73E-01	1	11	389	21447	9.09%	1.81%	0.26%	0.05%	shared jan 31	Pgk2
									1.83E-01	3.31E-01	1	19	227	21447	5.26%	1.06%	0.44%	0.09%	gluconeogenesis Jan 31	Slc25a13
									1.00E+00	1.00E+00	0	9	227	21447	0.00%	1.06%	0.00%	0.04%	glycolysis Jan 31	
				MP:0003308	abnormal cochlear sensory epithelium morphology	1.83E-01	1.00E+00	1.83E-01	1.00E+00	1.00E+00	0	11	227	21447	0.00%	1.06%	0.00%	0.05%	shared jan 31	
									1.83E-01	3.31E-01	1	19	227	21447	5.26%	1.06%	0.44%	0.09%	gluconeogenesis Jan 31	Slc25a13
									1.00E+00	1.00E+00	0	9	227	21447	0.00%	1.06%	0.00%	0.04%	glycolysis Jan 31	
				MP:0000045	abnormal hair cell morphology	1.83E-01	1.00E+00	1.83E-01	1.00E+00	1.00E+00	0	11	227	21447	0.00%	1.06%	0.00%	0.05%	shared jan 31	
									1.83E-01	3.31E-01	1	19	227	21447	5.26%	1.06%	0.44%	0.09%	gluconeogenesis Jan 31	Slc25a13
									1.00E+00	1.00E+00	0	9	227	21447	0.00%	1.06%	0.00%	0.04%	glycolysis Jan 31	
				MP:0000042	abnormal organ of Corti morphology	1.83E-01	1.00E+00	1.83E-01	1.00E+00	1.00E+00	0	11	227	21447	0.00%	1.06%	0.00%	0.05%	shared jan 31	
									1.00E+00	1.00E+00	0	19	392	21447	0.00%	1.83%	0.00%	0.09%	gluconeogenesis Jan 31	
									1.00E+00	1.00E+00	0	9	392	21447	0.00%	1.83%	0.00%	0.04%	glycolysis Jan 31	
				MP:0001951	abnormal breathing pattern	1.84E-01	1.00E+00	1.84E-01	1.84E-01	3.73E-01	1	11	392	21447	9.09%	1.83%	0.26%	0.05%	shared jan 31	Aldoc
									3.50E-01	4.59E-01	1	19	481	21447	5.26%	2.24%	0.21%	0.09%	gluconeogenesis Jan 31	G6pc3
									1.85E-01	2.64E-01	1	9	481	21447	11.11%	2.24%	0.21%	0.04%	glycolysis Jan 31	Gck
				MP:0001944	abnormal pancreas morphology	1.85E-01	1.00E+00	1.85E-01	1.00E+00	1.00E+00	0	11	481	21447	0.00%	2.24%	0.00%	0.05%	shared jan 31	
									1.85E-01	3.33E-01	1	19	230	21447	5.26%	1.07%	0.43%	0.09%	gluconeogenesis Jan 31	Got2
									1.00E+00	1.00E+00	0	9	230	21447	0.00%	1.07%	0.00%	0.04%	glycolysis Jan 31	
				MP:0001718	abnormal visceral yolk sac morphology	1.85E-01	1.00E+00	1.85E-01	1.00E+00	1.00E+00	0	11	230	21447	0.00%	1.07%	0.00%	0.05%	shared jan 31	
									2.99E-01	4.25E-01	1	19	397	21447	5.26%	1.85%	0.25%	0.09%	gluconeogenesis Jan 31	G6pc3
									1.00E+00	1.00E+00	0	9	397	21447	0.00%	1.85%	0.00%	0.04%	glycolysis Jan 31	
				MP:0009763	increased sensitivity to induced morbidity/mortality	1.86E-01	1.00E+00	1.86E-01	1.86E-01	3.75E-01	1	11	397	21447	9.09%	1.85%	0.25%	0.05%	shared jan 31	Aldob
									1.87E-01	3.34E-01	8	19	6473	21447	42.11%	30.18%	0.12%	0.09%	gluconeogenesis Jan 31	Fbp2, G6pc, Pck1, Pcx, Slc25a10, Slc25a12, Slc25a13, Slc37a4
									9.61E-01	9.65E-01	1	9	6473	21447	11.11%	30.18%	0.02%	0.04%	glycolysis Jan 31	Pfkm
				MP:0004924	abnormal behavior	1.87E-01	9.61E-01	1.94E-01	8.89E-01	9.01E-01	2	11	6473	21447	18.18%	30.18%	0.03%	0.05%	shared jan 31	Aldoa, Aldoc
									1.87E-01	3.34E-01	1	19	232	21447	5.26%	1.08%	0.43%	0.09%	gluconeogenesis Jan 31	G6pc
									1.00E+00	1.00E+00	0	9	232	21447	0.00%	1.08%	0.00%	0.04%	glycolysis Jan 31	
				MP:0000164	abnormal cartilage development	1.87E-01	1.00E+00	1.87E-01	1.00E+00	1.00E+00	0	11	232	21447	0.00%	1.08%	0.00%	0.05%	shared jan 31	
									1.00E+00	1.00E+00	0	19	487	21447	0.00%	2.27%	0.00%	0.09%	gluconeogenesis Jan 31	
									1.87E-01	2.65E-01	1	9	487	21447	11.11%	2.27%	0.21%	0.04%	glycolysis Jan 31	Pfkm
				MP:0010025	decreased total body fat amount	1.87E-01	1.00E+00	1.87E-01	1.00E+00	1.00E+00	0	11	487	21447	0.00%	2.27%	0.00%	0.05%	shared jan 31	
									1.00E+00	1.00E+00	0	19	492	21447	0.00%	2.29%	0.00%	0.09%	gluconeogenesis Jan 31	
									1.89E-01	2.66E-01	1	9	492	21447	11.11%	2.29%	0.20%	0.04%	glycolysis Jan 31	Gck
				MP:0002968	increased circulating alkaline phosphatase level	1.89E-01	1.00E+00	1.89E-01	1.00E+00	1.00E+00	0	11	492	21447	0.00%	2.29%	0.00%	0.05%	shared jan 31	
									4.14E-01	5.08E-01	4	19	3671	21447	21.05%	17.12%	0.11%	0.09%	gluconeogenesis Jan 31	G6pc2, G6pc3, Pcx, Slc37a2
									1.89E-01	2.66E-01	3	9	3671	21447	33.33%	17.12%	0.08%	0.04%	glycolysis Jan 31	Gck, Hk3, Pfkm
				MP:0005379	endocrine/exocrine gland phenotype	1.89E-01	8.73E-01	2.16E-01	8.73E-01	8.87E-01	1	11	3671	21447	9.09%	17.12%	0.03%	0.05%	shared jan 31	Eno4
									1.90E-01	3.37E-01	1	19	236	21447	5.26%	1.10%	0.42%	0.09%	gluconeogenesis Jan 31	Slc25a12
									1.00E+00	1.00E+00	0	9	236	21447	0.00%	1.10%	0.00%	0.04%	glycolysis Jan 31	
				MP:0031391	increased locomotor activity	1.90E-01	1.00E+00	1.90E-01	1.00E+00	1.00E+00	0	11	236	21447	0.00%	1.10%	0.00%	0.05%	shared jan 31	
									1.90E-01	3.37E-01	1	19	237	21447	5.26%	1.11%	0.42%	0.09%	gluconeogenesis Jan 31	G6pc2
									1.00E+00	1.00E+00	0	9	237	21447	0.00%	1.11%	0.00%	0.04%	glycolysis Jan 31	
				MP:0003725	increased autoantibody level	1.90E-01	1.00E+00	1.90E-01	1.00E+00	1.00E+00	0	11	237	21447	0.00%	1.11%	0.00%	0.05%	shared jan 31	
									1.91E-01	3.38E-01	2	19	908	21447	10.53%	4.23%	0.22%	0.09%	gluconeogenesis Jan 31	Pcx, Slc25a10
									1.00E+00	1.00E+00	0	9	908	21447	0.00%	4.23%	0.00%	0.04%	glycolysis Jan 31	
				MP:0001515	abnormal grip strength	1.91E-01	1.00E+00	1.91E-01	1.00E+00	1.00E+00	0	11	908	21447	0.00%	4.23%	0.00%	0.05%	shared jan 31	
									1.00E+00	1.00E+00	0	19	410	21447	0.00%	1.91%	0.00%	0.09%	gluconeogenesis Jan 31	
									1.00E+00	1.00E+00	0	9	410	21447	0.00%	1.91%	0.00%	0.04%	glycolysis Jan 31	
				MP:0004852	decreased testis weight	1.91E-01	1.00E+00	1.91E-01	1.91E-01	3.77E-01	1	11	410	21447	9.09%	1.91%	0.24%	0.05%	shared jan 31	Eno4
									3.63E-01	4.68E-01	1	19	502	21447	5.26%	2.34%	0.20%	0.09%	gluconeogenesis Jan 31	G6pc3
									1.92E-01	2.70E-01	1	9	502	21447	11.11%	2.34%	0.20%	0.04%	glycolysis Jan 31	Pfkm
				MP:0001258	decreased body length	1.92E-01	3.63E-01	5.30E-01	2.29E-01	4.08E-01	1	11	502	21447	9.09%	2.34%	0.20%	0.05%	shared jan 31	Aldob
									1.00E+00	1.00E+00	0	19	414	21447	0.00%	1.93%	0.00%	0.09%	gluconeogenesis Jan 31	
									1.00E+00	1.00E+00	0	9	414	21447	0.00%	1.93%	0.00%	0.04%	glycolysis Jan 31	
				MP:0013202	abnormal cilium morphology	1.93E-01	1.00E+00	1.93E-01	1.93E-01	3.77E-01	1	11	414	21447	9.09%	1.93%	0.24%	0.05%	shared jan 31	Eno4
									3.65E-01	4.68E-01	1	19	506	21447	5.26%	2.36%	0.20%	0.09%	gluconeogenesis Jan 31	Pck2
									1.93E-01	2.70E-01	1	9	506	21447	11.11%	2.36%	0.20%	0.04%	glycolysis Jan 31	Hk3
				MP:0000702	enlarged lymph nodes	1.93E-01	1.00E+00	1.93E-01	1.00E+00	1.00E+00	0	11	506	21447	0.00%	2.36%	0.00%	0.05%	shared jan 31	
									1.94E-01	3.41E-01	3	19	1730	21447	15.79%	8.07%	0.17%	0.09%	gluconeogenesis Jan 31	G6pc, Pcx, Slc37a4
									5.31E-01	5.61E-01	1	9	1730	21447	11.11%	8.07%	0.06%	0.04%	glycolysis Jan 31	Hk1
				MP:0000516	abnormal renal/urinary system morphology	1.94E-01	6.04E-01	3.21E-01	6.04E-01	6.42E-01	1	11	1730	21447	9.09%	8.07%	0.06%	0.05%	shared jan 31	Aldoc
									1.94E-01	3.41E-01	2	19	918	21447	10.53%	4.28%	0.22%	0.09%	gluconeogenesis Jan 31	G6pc, Slc25a13

					3.26E-01	3.85E-01	1	9	918	21447	11.11%	4.28%	0.11%	0.04%	glycolysis Jan 31	Gck
MP:0005502	abnormal renal/urinary system physiology	1.94E-01	1.00E+00	1.94E-01	1.00E+00	1.00E+00	0	11	918	21447	0.00%	4.28%	0.00%	0.05%	shared jan 31	
					1.95E-01	3.41E-01	2	19	919	21447	10.53%	4.28%	0.22%	0.09%	gluconeogenesis Jan 31	Got2, Mdh2
					3.26E-01	3.85E-01	1	9	919	21447	11.11%	4.28%	0.11%	0.04%	glycolysis Jan 31	Hk2
MP:0000913	abnormal brain development	1.95E-01	1.00E+00	1.95E-01	1.00E+00	1.00E+00	0	11	919	21447	0.00%	4.28%	0.00%	0.05%	shared jan 31	
					1.95E-01	3.41E-01	1	19	244	21447	5.26%	1.14%	0.41%	0.09%	gluconeogenesis Jan 31	G6pc
					1.00E+00	1.00E+00	0	9	244	21447	0.00%	1.14%	0.00%	0.04%	glycolysis Jan 31	
MP:0002962	increased urine protein level	1.95E-01	1.00E+00	1.95E-01	1.00E+00	1.00E+00	0	11	244	21447	0.00%	1.14%	0.00%	0.05%	shared jan 31	
					1.00E+00	1.00E+00	0	19	421	21447	0.00%	1.96%	0.00%	0.09%	gluconeogenesis Jan 31	
					1.00E+00	1.00E+00	0	9	421	21447	0.00%	1.96%	0.00%	0.04%	glycolysis Jan 31	
MP:0012765	decreased alpha-beta T cell number	1.96E-01	1.00E+00	1.96E-01	1.96E-01	3.77E-01	1	11	421	21447	9.09%	1.96%	0.24%	0.05%	shared jan 31	Pgam1
					1.00E+00	1.00E+00	0	19	421	21447	0.00%	1.96%	0.00%	0.09%	gluconeogenesis Jan 31	
					1.00E+00	1.00E+00	0	9	421	21447	0.00%	1.96%	0.00%	0.04%	glycolysis Jan 31	
MP:0005578	teratozoospermia	1.96E-01	1.00E+00	1.96E-01	1.96E-01	3.77E-01	1	11	421	21447	9.09%	1.96%	0.24%	0.05%	shared jan 31	Eno4
					1.00E+00	1.00E+00	0	19	422	21447	0.00%	1.97%	0.00%	0.09%	gluconeogenesis Jan 31	
					1.00E+00	1.00E+00	0	9	422	21447	0.00%	1.97%	0.00%	0.04%	glycolysis Jan 31	
MP:0002687	oligozoospermia	1.96E-01	1.00E+00	1.96E-01	1.96E-01	3.77E-01	1	11	422	21447	9.09%	1.97%	0.24%	0.05%	shared jan 31	Eno4
					1.97E-01	3.42E-01	1	19	246	21447	5.26%	1.15%	0.41%	0.09%	gluconeogenesis Jan 31	Slc37a2
					1.00E+00	1.00E+00	0	9	246	21447	0.00%	1.15%	0.00%	0.04%	glycolysis Jan 31	
MP:0008556	abnormal tumor necrosis factor secretion	1.97E-01	1.00E+00	1.97E-01	1.00E+00	1.00E+00	0	11	246	21447	0.00%	1.15%	0.00%	0.05%	shared jan 31	
					3.71E-01	4.70E-01	1	19	517	21447	5.26%	2.41%	0.19%	0.09%	gluconeogenesis Jan 31	Slc37a2
					1.97E-01	2.73E-01	1	9	517	21447	11.11%	2.41%	0.19%	0.04%	glycolysis Jan 31	Hk3
MP:0001304	cataract	1.97E-01	3.71E-01	5.31E-01	2.35E-01	4.12E-01	1	11	517	21447	9.09%	2.41%	0.19%	0.05%	shared jan 31	Gapdh
					1.99E-01	3.43E-01	1	19	249	21447	5.26%	1.16%	0.40%	0.09%	gluconeogenesis Jan 31	Slc25a13
					1.00E+00	1.00E+00	0	9	249	21447	0.00%	1.16%	0.00%	0.04%	glycolysis Jan 31	
MP:0000972	abnormal mechanoreceptor morphology	1.99E-01	1.00E+00	1.99E-01	1.00E+00	1.00E+00	0	11	249	21447	0.00%	1.16%	0.00%	0.05%	shared jan 31	
					1.99E-01	3.43E-01	1	19	249	21447	5.26%	1.16%	0.40%	0.09%	gluconeogenesis Jan 31	Slc25a12
					1.00E+00	1.00E+00	0	9	249	21447	0.00%	1.16%	0.00%	0.04%	glycolysis Jan 31	
MP:0002574	increased vertical activity	1.99E-01	1.00E+00	1.99E-01	1.00E+00	1.00E+00	0	11	249	21447	0.00%	1.16%	0.00%	0.05%	shared jan 31	
					1.00E+00	1.00E+00	0	19	437	21447	0.00%	2.04%	0.00%	0.09%	gluconeogenesis Jan 31	
					1.00E+00	1.00E+00	0	9	437	21447	0.00%	2.04%	0.00%	0.04%	glycolysis Jan 31	
MP:0004850	abnormal testis weight	2.03E-01	1.00E+00	2.03E-01	2.03E-01	3.87E-01	1	11	437	21447	9.09%	2.04%	0.23%	0.05%	shared jan 31	Eno4
					2.04E-01	3.46E-01	1	19	256	21447	5.26%	1.19%	0.39%	0.09%	gluconeogenesis Jan 31	Slc25a13
					1.00E+00	1.00E+00	0	9	256	21447	0.00%	1.19%	0.00%	0.04%	glycolysis Jan 31	
MP:0003169	abnormal scala media morphology	2.04E-01	1.00E+00	2.04E-01	1.00E+00	1.00E+00	0	11	256	21447	0.00%	1.19%	0.00%	0.05%	shared jan 31	
					1.00E+00	1.00E+00	0	19	538	21447	0.00%	2.51%	0.00%	0.09%	gluconeogenesis Jan 31	
					2.04E-01	2.80E-01	1	9	538	21447	11.11%	2.51%	0.19%	0.04%	glycolysis Jan 31	Hk2
MP:0000849	abnormal cerebellum morphology	2.04E-01	1.00E+00	2.04E-01	1.00E+00	1.00E+00	0	11	538	21447	0.00%	2.51%	0.00%	0.05%	shared jan 31	
					2.05E-01	3.46E-01	1	19	257	21447	5.26%	1.20%	0.39%	0.09%	gluconeogenesis Jan 31	Slc25a13
					1.00E+00	1.00E+00	0	9	257	21447	0.00%	1.20%	0.00%	0.04%	glycolysis Jan 31	
MP:0004426	abnormal cochlear labyrinth morphology	2.05E-01	1.00E+00	2.05E-01	1.00E+00	1.00E+00	0	11	257	21447	0.00%	1.20%	0.00%	0.05%	shared jan 31	
					2.05E-01	3.46E-01	1	19	257	21447	5.26%	1.20%	0.39%	0.09%	gluconeogenesis Jan 31	Slc25a12
					1.00E+00	1.00E+00	0	9	257	21447	0.00%	1.20%	0.00%	0.04%	glycolysis Jan 31	
MP:0000920	abnormal myelination	2.05E-01	1.00E+00	2.05E-01	1.00E+00	1.00E+00	0	11	257	21447	0.00%	1.20%	0.00%	0.05%	shared jan 31	
					2.05E-01	3.46E-01	1	19	257	21447	5.26%	1.20%	0.39%	0.09%	gluconeogenesis Jan 31	Pck2
					1.00E+00	1.00E+00	0	9	257	21447	0.00%	1.20%	0.00%	0.04%	glycolysis Jan 31	
MP:0000470	abnormal stomach morphology	2.05E-01	1.00E+00	2.05E-01	1.00E+00	1.00E+00	0	11	257	21447	0.00%	1.20%	0.00%	0.05%	shared jan 31	
					1.00E+00	1.00E+00	0	19	451	21447	0.00%	2.10%	0.00%	0.09%	gluconeogenesis Jan 31	
					1.00E+00	1.00E+00	0	9	451	21447	0.00%	2.10%	0.00%	0.04%	glycolysis Jan 31	
MP:0008077	abnormal CD8-positive, alpha-beta T cell number	2.09E-01	1.00E+00	2.09E-01	2.09E-01	3.91E-01	1	11	451	21447	9.09%	2.10%	0.22%	0.05%	shared jan 31	Pgam1
					1.00E+00	1.00E+00	0	19	452	21447	0.00%	2.11%	0.00%	0.09%	gluconeogenesis Jan 31	
					1.00E+00	1.00E+00	0	9	452	21447	0.00%	2.11%	0.00%	0.04%	glycolysis Jan 31	
MP:0001364	decreased anxiety-related response	2.09E-01	1.00E+00	2.09E-01	2.09E-01	3.91E-01	1	11	452	21447	9.09%	2.11%	0.22%	0.05%	shared jan 31	Aldoa
					2.09E-01	3.52E-01	1	19	263	21447	5.26%	1.23%	0.38%	0.09%	gluconeogenesis Jan 31	Slc25a13
					1.00E+00	1.00E+00	0	9	263	21447	0.00%	1.23%	0.00%	0.04%	glycolysis Jan 31	
MP:0001388	abnormal stationary movement	2.09E-01	1.00E+00	2.09E-01	1.00E+00	1.00E+00	0	11	263	21447	0.00%	1.23%	0.00%	0.05%	shared jan 31	
					1.00E+00	1.00E+00	0	19	555	21447	0.00%	2.59%	0.00%	0.09%	gluconeogenesis Jan 31	
					2.10E-01	2.86E-01	1	9	555	21447	11.11%	2.59%	0.18%	0.04%	glycolysis Jan 31	Gck
MP:0000351	increased cell proliferation	2.10E-01	1.00E+00	2.10E-01	1.00E+00	1.00E+00	0	11	555	21447	0.00%	2.59%	0.00%	0.05%	shared jan 31	
					1.00E+00	1.00E+00	0	19	556	21447	0.00%	2.59%	0.00%	0.09%	gluconeogenesis Jan 31	
					2.11E-01	2.86E-01	1	9	556	21447	11.11%	2.59%	0.18%	0.04%	glycolysis Jan 31	Pfkm
MP:0002833	increased heart weight	2.11E-01	1.00E+00	2.11E-01	2.51E-01	4.29E-01	1	11	556	21447	9.09%	2.59%	0.18%	0.05%	shared jan 31	Gpi1
					2.47E-01	3.83E-01	9	19	7992	21447	47.37%	37.26%	0.11%	0.09%	gluconeogenesis Jan 31	G6pc, G6pc3, Got2, Mdh2, Pck1, Pcx, Slc25a12, Slc37a2, Slc37a4
					2.12E-01	2.87E-01	5	9	7992	21447	55.56%	37.26%	0.06%	0.04%	glycolysis Jan 31	Gck, Hk1, Hk2, Pfkm, Pklr
MP:0005378	growth/size/body region phenotype	2.12E-01	3.92E-01	5.41E-01	3.92E-01	5.07E-01	5	11	7992	21447	45.45%	37.26%	0.06%	0.05%	shared jan 31	Aldob, Eno1, Gapdh, Gpi1, Tpi1
					2.13E-01	3.57E-01	1	19	268	21447	5.26%	1.25%	0.37%	0.09%	gluconeogenesis Jan 31	G6pc
					1.00E+00	1.00E+00	0	9	268	21447	0.00%	1.25%	0.00%	0.04%	glycolysis Jan 31	
MP:0006315	abnormal urine protein level	2.13E-01	1.00E+00	2.13E-01	1.00E+00	1.00E+00	0	11	268	21447	0.00%	1.25%	0.00%	0.05%	shared jan 31	
					1.00E+00	1.00E+00	0	19	461	21447	0.00%	2.15%	0.00%	0.09%	gluconeogenesis Jan 31	

					1.00E+00	1.00E+00	0	9	461	21447	0.00%	2.15%	0.00%	0.04%	glycolysis Jan 31	
MP:0002145	abnormal T cell differentiation	2.13E-01	1.00E+00	2.13E-01	2.13E-01	3.94E-01	1	11	461	21447	9.09%	2.15%	0.22%	0.05%	shared jan 31	Pgam1
					2.13E-01	3.57E-01	1	19	269	21447	5.26%	1.25%	0.37%	0.09%	gluconeogenesis Jan 31	G6pc
					1.00E+00	1.00E+00	0	9	269	21447	0.00%	1.25%	0.00%	0.04%	glycolysis Jan 31	
MP:0008987	abnormal liver lobule morphology	2.13E-01	1.00E+00	2.13E-01	1.00E+00	1.00E+00	0	11	269	21447	0.00%	1.25%	0.00%	0.05%	shared jan 31	
					3.98E-01	4.95E-01	1	19	565	21447	5.26%	2.63%	0.18%	0.09%	gluconeogenesis Jan 31	Slc37a4
					2.14E-01	2.89E-01	1	9	565	21447	11.11%	2.63%	0.18%	0.04%	glycolysis Jan 31	Hk2
MP:0003091	abnormal cell migration	2.14E-01	1.00E+00	2.14E-01	1.00E+00	1.00E+00	0	11	565	21447	0.00%	2.63%	0.00%	0.05%	shared jan 31	
					1.00E+00	1.00E+00	0	19	571	21447	0.00%	2.66%	0.00%	0.09%	gluconeogenesis Jan 31	
					2.16E-01	2.89E-01	1	9	571	21447	11.11%	2.66%	0.18%	0.04%	glycolysis Jan 31	Pfkm
MP:0003921	abnormal heart left ventricle morphology	2.16E-01	1.00E+00	2.16E-01	2.57E-01	4.33E-01	1	11	571	21447	9.09%	2.66%	0.18%	0.05%	shared jan 31	Aldob
					4.02E-01	4.99E-01	1	19	573	21447	5.26%	2.67%	0.17%	0.09%	gluconeogenesis Jan 31	Pck2
					2.16E-01	2.89E-01	1	9	573	21447	11.11%	2.67%	0.17%	0.04%	glycolysis Jan 31	Hk3
MP:0000701	abnormal lymph node size	2.16E-01	1.00E+00	2.16E-01	1.00E+00	1.00E+00	0	11	573	21447	0.00%	2.67%	0.00%	0.05%	shared jan 31	
					2.17E-01	3.61E-01	1	19	274	21447	5.26%	1.28%	0.36%	0.09%	gluconeogenesis Jan 31	Slc25a13
					1.00E+00	1.00E+00	0	9	274	21447	0.00%	1.28%	0.00%	0.04%	glycolysis Jan 31	
MP:0000031	abnormal cochlea morphology	2.17E-01	1.00E+00	2.17E-01	1.00E+00	1.00E+00	0	11	274	21447	0.00%	1.28%	0.00%	0.05%	shared jan 31	
					1.00E+00	1.00E+00	0	19	576	21447	0.00%	2.69%	0.00%	0.09%	gluconeogenesis Jan 31	
					2.17E-01	2.89E-01	1	9	576	21447	11.11%	2.69%	0.17%	0.04%	glycolysis Jan 31	Hk2
MP:0000847	abnormal metencephalon morphology	2.17E-01	1.00E+00	2.17E-01	1.00E+00	1.00E+00	0	11	576	21447	0.00%	2.69%	0.00%	0.05%	shared jan 31	
					1.00E+00	1.00E+00	0	19	476	21447	0.00%	2.22%	0.00%	0.09%	gluconeogenesis Jan 31	
					1.00E+00	1.00E+00	0	9	476	21447	0.00%	2.22%	0.00%	0.04%	glycolysis Jan 31	
MP:0000063	decreased bone mineral density	2.19E-01	1.00E+00	2.19E-01	2.19E-01	4.03E-01	1	11	476	21447	9.09%	2.22%	0.21%	0.05%	shared jan 31	Aldob
					4.08E-01	5.05E-01	1	19	583	21447	5.26%	2.72%	0.17%	0.09%	gluconeogenesis Jan 31	G6pc3
					2.20E-01	2.91E-01	1	9	583	21447	11.11%	2.72%	0.17%	0.04%	glycolysis Jan 31	Pfkm
MP:0001256	abnormal body length	2.20E-01	4.08E-01	5.39E-01	2.62E-01	4.35E-01	1	11	583	21447	9.09%	2.72%	0.17%	0.05%	shared jan 31	Aldob
					2.20E-01	3.66E-01	3	19	1843	21447	15.79%	8.59%	0.16%	0.09%	gluconeogenesis Jan 31	G6pc2, Slc37a2, Slc37a4
					1.00E+00	1.00E+00	0	9	1843	21447	0.00%	8.59%	0.00%	0.04%	glycolysis Jan 31	
MP:0001845	abnormal inflammatory response	2.20E-01	1.00E+00	2.20E-01	2.43E-01	4.19E-01	2	11	1843	21447	18.18%	8.59%	0.11%	0.05%	shared jan 31	Aldob, Pgam1
					4.09E-01	5.06E-01	1	19	586	21447	5.26%	2.73%	0.17%	0.09%	gluconeogenesis Jan 31	G6pc3
					2.21E-01	2.91E-01	1	9	586	21447	11.11%	2.73%	0.17%	0.04%	glycolysis Jan 31	Pklr
MP:0002406	increased susceptibility to infection	2.21E-01	1.00E+00	2.21E-01	1.00E+00	1.00E+00	0	11	586	21447	0.00%	2.73%	0.00%	0.05%	shared jan 31	
					2.21E-01	3.66E-01	1	19	280	21447	5.26%	1.31%	0.36%	0.09%	gluconeogenesis Jan 31	Mdh1
					1.00E+00	1.00E+00	0	9	280	21447	0.00%	1.31%	0.00%	0.04%	glycolysis Jan 31	
MP:0020135	abnormal heart ventricle wall thickness	2.21E-01	1.00E+00	2.21E-01	1.00E+00	1.00E+00	0	11	280	21447	0.00%	1.31%	0.00%	0.05%	shared jan 31	
					2.25E-01	3.67E-01	1	19	285	21447	5.26%	1.33%	0.35%	0.09%	gluconeogenesis Jan 31	Pcx
					1.00E+00	1.00E+00	0	9	285	21447	0.00%	1.33%	0.00%	0.04%	glycolysis Jan 31	
MP:0006036	abnormal mitochondrial physiology	2.25E-01	1.00E+00	2.25E-01	1.00E+00	1.00E+00	0	11	285	21447	0.00%	1.33%	0.00%	0.05%	shared jan 31	
					2.25E-01	3.67E-01	3	19	1861	21447	15.79%	8.68%	0.16%	0.09%	gluconeogenesis Jan 31	G6pc2, Got1, Slc37a4
					1.00E+00	1.00E+00	0	9	1861	21447	0.00%	8.68%	0.00%	0.04%	glycolysis Jan 31	
MP:0002619	abnormal lymphocyte morphology	2.25E-01	1.00E+00	2.25E-01	6.32E-01	6.68E-01	1	11	1861	21447	9.09%	8.68%	0.05%	0.05%	shared jan 31	Pgam1
					2.25E-01	3.67E-01	1	19	286	21447	5.26%	1.33%	0.35%	0.09%	gluconeogenesis Jan 31	Slc25a10
					1.00E+00	1.00E+00	0	9	286	21447	0.00%	1.33%	0.00%	0.04%	glycolysis Jan 31	
MP:0001488	increased startle reflex	2.25E-01	1.00E+00	2.25E-01	1.00E+00	1.00E+00	0	11	286	21447	0.00%	1.33%	0.00%	0.05%	shared jan 31	
					3.58E-01	4.66E-01	1	19	494	21447	5.26%	2.30%	0.20%	0.09%	gluconeogenesis Jan 31	G6pc2
					1.00E+00	1.00E+00	0	9	494	21447	0.00%	2.30%	0.00%	0.04%	glycolysis Jan 31	
MP:0005010	abnormal CD8-positive, alpha beta T cell morphology	2.26E-01	1.00E+00	2.26E-01	2.26E-01	4.08E-01	1	11	494	21447	9.09%	2.30%	0.20%	0.05%	shared jan 31	Pgam1
					1.00E+00	1.00E+00	0	19	496	21447	0.00%	2.31%	0.00%	0.09%	gluconeogenesis Jan 31	
					1.00E+00	1.00E+00	0	9	496	21447	0.00%	2.31%	0.00%	0.04%	glycolysis Jan 31	
MP:0002327	abnormal respiratory function	2.27E-01	1.00E+00	2.27E-01	2.27E-01	4.08E-01	1	11	496	21447	9.09%	2.31%	0.20%	0.05%	shared jan 31	Aldoc
					3.60E-01	4.68E-01	1	19	498	21447	5.26%	2.32%	0.20%	0.09%	gluconeogenesis Jan 31	G6pc2
					1.00E+00	1.00E+00	0	9	498	21447	0.00%	2.32%	0.00%	0.04%	glycolysis Jan 31	
MP:0002425	abnormal susceptibility to autoimmune disorder	2.28E-01	1.00E+00	2.28E-01	2.28E-01	4.08E-01	1	11	498	21447	9.09%	2.32%	0.20%	0.05%	shared jan 31	Pgam1
					2.28E-01	3.69E-01	1	19	290	21447	5.26%	1.35%	0.34%	0.09%	gluconeogenesis Jan 31	Slc25a13
					1.00E+00	1.00E+00	0	9	290	21447	0.00%	1.35%	0.00%	0.04%	glycolysis Jan 31	
MP:0000035	abnormal membranous labyrinth morphology	2.28E-01	1.00E+00	2.28E-01	1.00E+00	1.00E+00	0	11	290	21447	0.00%	1.35%	0.00%	0.05%	shared jan 31	
					2.28E-01	3.69E-01	1	19	290	21447	5.26%	1.35%	0.34%	0.09%	gluconeogenesis Jan 31	Got1
					1.00E+00	1.00E+00	0	9	290	21447	0.00%	1.35%	0.00%	0.04%	glycolysis Jan 31	
MP:0005333	decreased heart rate	2.28E-01	1.00E+00	2.28E-01	1.00E+00	1.00E+00	0	11	290	21447	0.00%	1.35%	0.00%	0.05%	shared jan 31	
					2.29E-01	3.69E-01	1	19	291	21447	5.26%	1.36%	0.34%	0.09%	gluconeogenesis Jan 31	Slc25a13
					1.00E+00	1.00E+00	0	9	291	21447	0.00%	1.36%	0.00%	0.04%	glycolysis Jan 31	
MP:0011967	increased or absent threshold for auditory brainstem response	2.29E-01	1.00E+00	2.29E-01	1.00E+00	1.00E+00	0	11	291	21447	0.00%	1.36%	0.00%	0.05%	shared jan 31	
					1.00E+00	1.00E+00	0	19	610	21447	0.00%	2.84%	0.00%	0.09%	gluconeogenesis Jan 31	
					2.29E-01	2.98E-01	1	9	610	21447	11.11%	2.84%	0.16%	0.04%	glycolysis Jan 31	Gck
MP:0000202	abnormal circulating alkaline phosphatase level	2.29E-01	1.00E+00	2.29E-01	1.00E+00	1.00E+00	0	11	610	21447	0.00%	2.84%	0.00%	0.05%	shared jan 31	
					2.31E-01	3.72E-01	1	19	295	21447	5.26%	1.38%	0.34%	0.09%	gluconeogenesis Jan 31	G6pc2
					1.00E+00	1.00E+00	0	9	295	21447	0.00%	1.38%	0.00%	0.04%	glycolysis Jan 31	
MP:0005350	increased susceptibility to autoimmune disorder	2.31E-01	1.00E+00	2.31E-01	1.00E+00	1.00E+00	0	11	295	21447	0.00%	1.38%	0.00%	0.05%	shared jan 31	
					2.32E-01	3.72E-01	1	19	296	21447	5.26%	1.38%	0.34%	0.09%	gluconeogenesis Jan 31	Slc25a13
					1.00E+00	1.00E+00	0	9	296	21447	0.00%	1.38%	0.00%	0.04%	glycolysis Jan 31	

				MP:0006325	impaired hearing	2.32E-01	1.00E+00	2.32E-01	1.00E+00	1.00E+00	0	11	296	21447	0.00%	1.38%	0.00%	0.05%	shared jan 31	
									1.00E+00	1.00E+00	0	19	510	21447	0.00%	2.38%	0.00%	0.09%	gluconeogenesis Jan 31	
									1.00E+00	1.00E+00	0	9	510	21447	0.00%	2.38%	0.00%	0.04%	glycolysis Jan 31	
				MP:0008073	abnormal CD4-positive, alpha beta T cell number	2.33E-01	1.00E+00	2.33E-01	2.33E-01	4.12E-01	1	11	510	21447	9.09%	2.38%	0.20%	0.05%	shared jan 31	Pgam1
									2.33E-01	3.73E-01	2	19	1035	21447	10.53%	4.83%	0.19%	0.09%	gluconeogenesis Jan 31	Pcx, Slc25a10
									1.00E+00	1.00E+00	0	9	1035	21447	0.00%	4.83%	0.00%	0.04%	glycolysis Jan 31	
				MP:0004262	abnormal physical strength	2.33E-01	1.00E+00	2.33E-01	1.00E+00	1.00E+00	0	11	1035	21447	0.00%	4.83%	0.00%	0.05%	shared jan 31	
									2.34E-01	3.74E-01	2	19	1039	21447	10.53%	4.84%	0.19%	0.09%	gluconeogenesis Jan 31	Slc25a12, Slc25a13
									1.00E+00	1.00E+00	0	9	1039	21447	0.00%	4.84%	0.00%	0.04%	glycolysis Jan 31	
				MP:0003312	abnormal locomotor coordination	2.34E-01	1.00E+00	2.34E-01	1.00E+00	1.00E+00	0	11	1039	21447	0.00%	4.84%	0.00%	0.05%	shared jan 31	
									1.00E+00	1.00E+00	0	19	628	21447	0.00%	2.93%	0.00%	0.09%	gluconeogenesis Jan 31	
									2.35E-01	3.03E-01	1	9	628	21447	11.11%	2.93%	0.16%	0.04%	glycolysis Jan 31	Gck
				MP:0001260	increased body weight	2.35E-01	1.00E+00	2.35E-01	1.00E+00	1.00E+00	0	11	628	21447	0.00%	2.93%	0.00%	0.05%	shared jan 31	
									1.00E+00	1.00E+00	0	19	628	21447	0.00%	2.93%	0.00%	0.09%	gluconeogenesis Jan 31	
									2.35E-01	3.03E-01	1	9	628	21447	11.11%	2.93%	0.16%	0.04%	glycolysis Jan 31	Gck
				MP:0012323	increased total tissue mass	2.35E-01	1.00E+00	2.35E-01	1.00E+00	1.00E+00	0	11	628	21447	0.00%	2.93%	0.00%	0.05%	shared jan 31	
									3.71E-01	4.70E-01	1	19	516	21447	5.26%	2.41%	0.19%	0.09%	gluconeogenesis Jan 31	Pcx
									1.00E+00	1.00E+00	0	9	516	21447	0.00%	2.41%	0.00%	0.04%	glycolysis Jan 31	
				MP:0000609	abnormal liver physiology	2.35E-01	1.00E+00	2.35E-01	2.35E-01	4.12E-01	1	11	516	21447	9.09%	2.41%	0.19%	0.05%	shared jan 31	Aldob
									2.37E-01	3.77E-01	1	19	303	21447	5.26%	1.41%	0.33%	0.09%	gluconeogenesis Jan 31	Pck1
									1.00E+00	1.00E+00	0	9	303	21447	0.00%	1.41%	0.00%	0.04%	glycolysis Jan 31	
				MP:0009115	abnormal fat cell morphology	2.37E-01	1.00E+00	2.37E-01	1.00E+00	1.00E+00	0	11	303	21447	0.00%	1.41%	0.00%	0.05%	shared jan 31	
									1.00E+00	1.00E+00	0	19	649	21447	0.00%	3.03%	0.00%	0.09%	gluconeogenesis Jan 31	
									2.42E-01	3.09E-01	1	9	649	21447	11.11%	3.03%	0.15%	0.04%	glycolysis Jan 31	Hk2
				MP:0000841	abnormal hindbrain morphology	2.42E-01	1.00E+00	2.42E-01	1.00E+00	1.00E+00	0	11	649	21447	0.00%	3.03%	0.00%	0.05%	shared jan 31	
									4.42E-01	5.27E-01	1	19	649	21447	5.26%	3.03%	0.15%	0.09%	gluconeogenesis Jan 31	Pck1
									2.42E-01	3.09E-01	1	9	649	21447	11.11%	3.03%	0.15%	0.04%	glycolysis Jan 31	Hk1
				MP:0009931	abnormal skin appearance	2.42E-01	1.00E+00	2.42E-01	1.00E+00	1.00E+00	0	11	649	21447	0.00%	3.03%	0.00%	0.05%	shared jan 31	
									2.43E-01	3.83E-01	1	19	312	21447	5.26%	1.45%	0.32%	0.09%	gluconeogenesis Jan 31	Pck1
									1.00E+00	1.00E+00	0	9	312	21447	0.00%	1.45%	0.00%	0.04%	glycolysis Jan 31	
				MP:0005565	increased blood urea nitrogen level	2.43E-01	1.00E+00	2.43E-01	1.00E+00	1.00E+00	0	11	312	21447	0.00%	1.45%	0.00%	0.05%	shared jan 31	
									2.43E-01	3.83E-01	1	19	312	21447	5.26%	1.45%	0.32%	0.09%	gluconeogenesis Jan 31	Pck2
									1.00E+00	1.00E+00	0	9	312	21447	0.00%	1.45%	0.00%	0.04%	glycolysis Jan 31	
				MP:0005343	increased circulating aspartate transaminase level	2.43E-01	1.00E+00	2.43E-01	1.00E+00	1.00E+00	0	11	312	21447	0.00%	1.45%	0.00%	0.05%	shared jan 31	
									3.82E-01	4.80E-01	1	19	537	21447	5.26%	2.50%	0.19%	0.09%	gluconeogenesis Jan 31	G6pc3
									1.00E+00	1.00E+00	0	9	537	21447	0.00%	2.50%	0.00%	0.04%	glycolysis Jan 31	
				MP:0020148	abnormal sensitivity to induced morbidity/mortality	2.43E-01	1.00E+00	2.43E-01	2.43E-01	4.19E-01	1	11	537	21447	9.09%	2.50%	0.19%	0.05%	shared jan 31	Aldob
									2.44E-01	3.83E-01	1	19	313	21447	5.26%	1.46%	0.32%	0.09%	gluconeogenesis Jan 31	G6pc2
									1.00E+00	1.00E+00	0	9	313	21447	0.00%	1.46%	0.00%	0.04%	glycolysis Jan 31	
				MP:0008559	abnormal interferon-gamma secretion	2.44E-01	1.00E+00	2.44E-01	1.00E+00	1.00E+00	0	11	313	21447	0.00%	1.46%	0.00%	0.05%	shared jan 31	
									1.00E+00	1.00E+00	0	19	661	21447	0.00%	3.08%	0.00%	0.09%	gluconeogenesis Jan 31	
									2.46E-01	3.13E-01	1	9	661	21447	11.11%	3.08%	0.15%	0.04%	glycolysis Jan 31	Pfkm
				MP:0000759	abnormal skeletal muscle morphology	2.46E-01	1.00E+00	2.46E-01	1.00E+00	1.00E+00	0	11	661	21447	0.00%	3.08%	0.00%	0.05%	shared jan 31	
									2.46E-01	3.83E-01	1	19	316	21447	5.26%	1.47%	0.32%	0.09%	gluconeogenesis Jan 31	G6pc
									1.00E+00	1.00E+00	0	9	316	21447	0.00%	1.47%	0.00%	0.04%	glycolysis Jan 31	
				MP:0002703	abnormal renal tubule morphology	2.46E-01	1.00E+00	2.46E-01	1.00E+00	1.00E+00	0	11	316	21447	0.00%	1.47%	0.00%	0.05%	shared jan 31	
									2.50E-01	3.83E-01	1	19	322	21447	5.26%	1.50%	0.31%	0.09%	gluconeogenesis Jan 31	Got1
									1.00E+00	1.00E+00	0	9	322	21447	0.00%	1.50%	0.00%	0.04%	glycolysis Jan 31	
				MP:0002414	abnormal myeloblast morphology/development	2.50E-01	1.00E+00	2.50E-01	1.00E+00	1.00E+00	0	11	322	21447	0.00%	1.50%	0.00%	0.05%	shared jan 31	
									4.55E-01	5.35E-01	1	19	675	21447	5.26%	3.15%	0.15%	0.09%	gluconeogenesis Jan 31	Pcx
									2.50E-01	3.18E-01	1	9	675	21447	11.11%	3.15%	0.15%	0.04%	glycolysis Jan 31	Pfkm
				MP:0003948	abnormal gas homeostasis	2.50E-01	4.55E-01	5.49E-01	2.97E-01	4.60E-01	1	11	675	21447	9.09%	3.15%	0.15%	0.05%	shared jan 31	Pgam1
									3.95E-01	4.92E-01	1	19	559	21447	5.26%	2.61%	0.18%	0.09%	gluconeogenesis Jan 31	Pcx
									1.00E+00	1.00E+00	0	9	559	21447	0.00%	2.61%	0.00%	0.04%	glycolysis Jan 31	
				MP:0002139	abnormal hepatobiliary system physiology	2.52E-01	1.00E+00	2.52E-01	2.52E-01	4.29E-01	1	11	559	21447	9.09%	2.61%	0.18%	0.05%	shared jan 31	Aldob
									1.00E+00	1.00E+00	0	19	683	21447	0.00%	3.18%	0.00%	0.09%	gluconeogenesis Jan 31	
									2.53E-01	3.20E-01	1	9	683	21447	11.11%	3.18%	0.15%	0.04%	glycolysis Jan 31	Gck
				MP:0001264	increased body size	2.53E-01	1.00E+00	2.53E-01	1.00E+00	1.00E+00	0	11	683	21447	0.00%	3.18%	0.00%	0.05%	shared jan 31	
									4.60E-01	5.38E-01	1	19	684	21447	5.26%	3.19%	0.15%	0.09%	gluconeogenesis Jan 31	Pcx
									2.53E-01	3.20E-01	1	9	684	21447	11.11%	3.19%	0.15%	0.04%	glycolysis Jan 31	Pfkm
				MP:0003959	abnormal lean body mass	2.53E-01	1.00E+00	2.53E-01	1.00E+00	1.00E+00	0	11	684	21447	0.00%	3.19%	0.00%	0.05%	shared jan 31	
									1.00E+00	1.00E+00	0	19	1902	21447	0.00%	8.87%	0.00%	0.09%	gluconeogenesis Jan 31	
									1.00E+00	1.00E+00	0	9	1902	21447	0.00%	8.87%	0.00%	0.04%	glycolysis Jan 31	
				MP:0005388	respiratory system phenotype	2.55E-01	1.00E+00	2.55E-01	2.55E-01	4.31E-01	2	11	1902	21447	18.18%	8.87%	0.11%	0.05%	shared jan 31	Aldoc, Gpi1
									2.56E-01	3.90E-01	2	19	1103	21447	10.53%	5.14%	0.18%	0.09%	gluconeogenesis Jan 31	G6pc, Slc37a4
									1.00E+00	1.00E+00	0	9	1103	21447	0.00%	5.14%	0.00%	0.04%	glycolysis Jan 31	
				MP:0009250	abnormal appendicular skeleton morphology	2.56E-01	1.00E+00	2.56E-01	1.00E+00	1.00E+00	0	11	1103	21447	0.00%	5.14%	0.00%	0.05%	shared jan 31	
									1.00E+00	1.00E+00	0	19	698	21447	0.00%	3.25%	0.00%	0.09%	gluconeogenesis Jan 31	
									2.58E-01	3.24E-01	1	9	698	21447	11.11%	3.25%	0.14%	0.04%	glycolysis Jan 31	Pfkm
				MP:0004857	abnormal heart weight	2.58E-01	1.00E+00	2.58E-01	3.05E-01	4.64E-01	1	11	698	21447	9.09%	3.25%	0.14%	0.05%	shared jan 31	Gpi1

					2.59E-01	3.95E-01	1	19	336	21447	5.26%	1.57%	0.30%	0.09%	gluconeogenesis Jan 31	Slc25a13
					1.00E+00	1.00E+00	0	9	336	21447	0.00%	1.57%	0.00%	0.04%	glycolysis Jan 31	
MP:0000026	abnormal inner ear morphology	2.59E-01	1.00E+00	2.59E-01	1.00E+00	1.00E+00	0	11	336	21447	0.00%	1.57%	0.00%	0.05%	shared jan 31	
					2.63E-01	3.98E-01	1	19	342	21447	5.26%	1.59%	0.29%	0.09%	gluconeogenesis Jan 31	Slc25a12
					1.00E+00	1.00E+00	0	9	342	21447	0.00%	1.59%	0.00%	0.04%	glycolysis Jan 31	
MP:0001363	increased anxiety-related response	2.63E-01	1.00E+00	2.63E-01	1.00E+00	1.00E+00	0	11	342	21447	0.00%	1.59%	0.00%	0.05%	shared jan 31	
					4.11E-01	5.07E-01	1	19	589	21447	5.26%	2.75%	0.17%	0.09%	gluconeogenesis Jan 31	Slc37a2
					1.00E+00	1.00E+00	0	9	589	21447	0.00%	2.75%	0.00%	0.04%	glycolysis Jan 31	
MP:0008568	abnormal interleukin secretion	2.64E-01	1.00E+00	2.64E-01	2.64E-01	4.35E-01	1	11	589	21447	9.09%	2.75%	0.17%	0.05%	shared jan 31	Pgam1
					1.00E+00	1.00E+00	0	19	594	21447	0.00%	2.77%	0.00%	0.09%	gluconeogenesis Jan 31	
					1.00E+00	1.00E+00	0	9	594	21447	0.00%	2.77%	0.00%	0.04%	glycolysis Jan 31	
MP:0002432	abnormal CD4-positive, alpha beta T cell morphology	2.66E-01	1.00E+00	2.66E-01	2.66E-01	4.36E-01	1	11	594	21447	9.09%	2.77%	0.17%	0.05%	shared jan 31	Pgam1
					1.00E+00	1.00E+00	0	19	725	21447	0.00%	3.38%	0.00%	0.09%	gluconeogenesis Jan 31	
					2.66E-01	3.31E-01	1	9	725	21447	11.11%	3.38%	0.14%	0.04%	glycolysis Jan 31	Pfkm
MP:0004087	abnormal muscle fiber morphology	2.66E-01	1.00E+00	2.66E-01	1.00E+00	1.00E+00	0	11	725	21447	0.00%	3.38%	0.00%	0.05%	shared jan 31	
					1.00E+00	1.00E+00	0	19	731	21447	0.00%	3.41%	0.00%	0.09%	gluconeogenesis Jan 31	
					2.68E-01	3.32E-01	1	9	731	21447	11.11%	3.41%	0.14%	0.04%	glycolysis Jan 31	Hkdc1
MP:0005253	abnormal eye physiology	2.68E-01	1.00E+00	2.68E-01	1.00E+00	1.00E+00	0	11	731	21447	0.00%	3.41%	0.00%	0.05%	shared jan 31	
					3.64E-01	4.68E-01	3	19	2418	21447	15.79%	11.27%	0.12%	0.09%	gluconeogenesis Jan 31	G6pc3, Slc25a12, Slc37a4
					2.70E-01	3.32E-01	2	9	2418	21447	22.22%	11.27%	0.08%	0.04%	glycolysis Jan 31	Gck, Hk2
MP:0001262	decreased body weight	2.70E-01	3.64E-01	7.41E-01	3.57E-01	4.89E-01	2	11	2418	21447	18.18%	11.27%	0.08%	0.05%	shared jan 31	Gpi1, Tpi1
					3.64E-01	4.68E-01	3	19	2419	21447	15.79%	11.28%	0.12%	0.09%	gluconeogenesis Jan 31	G6pc3, Slc25a12, Slc37a4
					2.70E-01	3.32E-01	2	9	2419	21447	22.22%	11.28%	0.08%	0.04%	glycolysis Jan 31	Gck, Hk2
MP:0012322	decreased total tissue mass	2.70E-01	3.64E-01	7.41E-01	3.57E-01	4.89E-01	2	11	2419	21447	18.18%	11.28%	0.08%	0.05%	shared jan 31	Gpi1, Tpi1
					2.72E-01	4.08E-01	1	19	355	21447	5.26%	1.66%	0.28%	0.09%	gluconeogenesis Jan 31	Slc25a12
					1.00E+00	1.00E+00	0	9	355	21447	0.00%	1.66%	0.00%	0.04%	glycolysis Jan 31	
MP:0001504	abnormal posture	2.72E-01	1.00E+00	2.72E-01	1.00E+00	1.00E+00	0	11	355	21447	0.00%	1.66%	0.00%	0.05%	shared jan 31	
					2.73E-01	4.08E-01	1	19	356	21447	5.26%	1.66%	0.28%	0.09%	gluconeogenesis Jan 31	Pck1
					1.00E+00	1.00E+00	0	9	356	21447	0.00%	1.66%	0.00%	0.04%	glycolysis Jan 31	
MP:0000010	abnormal abdominal fat pad morphology	2.73E-01	1.00E+00	2.73E-01	1.00E+00	1.00E+00	0	11	356	21447	0.00%	1.66%	0.00%	0.05%	shared jan 31	
					2.74E-01	4.09E-01	1	19	358	21447	5.26%	1.67%	0.28%	0.09%	gluconeogenesis Jan 31	Slc37a4
					1.00E+00	1.00E+00	0	9	358	21447	0.00%	1.67%	0.00%	0.04%	glycolysis Jan 31	
MP:0002357	abnormal spleen white pulp morphology	2.74E-01	1.00E+00	2.74E-01	1.00E+00	1.00E+00	0	11	358	21447	0.00%	1.67%	0.00%	0.05%	shared jan 31	
					4.25E-01	5.14E-01	1	19	616	21447	5.26%	2.87%	0.16%	0.09%	gluconeogenesis Jan 31	Slc25a13
					1.00E+00	1.00E+00	0	9	616	21447	0.00%	2.87%	0.00%	0.04%	glycolysis Jan 31	
MP:0004738	abnormal auditory brainstem response	2.74E-01	1.00E+00	2.74E-01	2.74E-01	4.45E-01	1	11	616	21447	9.09%	2.87%	0.16%	0.05%	shared jan 31	Aldoc
					2.76E-01	4.10E-01	1	19	361	21447	5.26%	1.68%	0.28%	0.09%	gluconeogenesis Jan 31	Slc25a12
					1.00E+00	1.00E+00	0	9	361	21447	0.00%	1.68%	0.00%	0.04%	glycolysis Jan 31	
MP:0000822	abnormal brain ventricle morphology	2.76E-01	1.00E+00	2.76E-01	1.00E+00	1.00E+00	0	11	361	21447	0.00%	1.68%	0.00%	0.05%	shared jan 31	
					1.00E+00	1.00E+00	0	19	756	21447	0.00%	3.52%	0.00%	0.09%	gluconeogenesis Jan 31	
					2.76E-01	3.39E-01	1	9	756	21447	11.11%	3.52%	0.13%	0.04%	glycolysis Jan 31	Hk2
MP:0004811	abnormal neuron physiology	2.76E-01	1.00E+00	2.76E-01	1.00E+00	1.00E+00	0	11	756	21447	0.00%	3.52%	0.00%	0.05%	shared jan 31	
					2.77E-01	4.10E-01	1	19	363	21447	5.26%	1.69%	0.28%	0.09%	gluconeogenesis Jan 31	Pck2
					1.00E+00	1.00E+00	0	9	363	21447	0.00%	1.69%	0.00%	0.04%	glycolysis Jan 31	
MP:0000203	abnormal circulating aspartate transaminase level	2.77E-01	1.00E+00	2.77E-01	1.00E+00	1.00E+00	0	11	363	21447	0.00%	1.69%	0.00%	0.05%	shared jan 31	
					2.77E-01	4.10E-01	1	19	363	21447	5.26%	1.69%	0.28%	0.09%	gluconeogenesis Jan 31	Pcx
					1.00E+00	1.00E+00	0	9	363	21447	0.00%	1.69%	0.00%	0.04%	glycolysis Jan 31	
MP:0005292	improved glucose tolerance	2.77E-01	1.00E+00	2.77E-01	1.00E+00	1.00E+00	0	11	363	21447	0.00%	1.69%	0.00%	0.05%	shared jan 31	
					2.80E-01	4.12E-01	1	19	367	21447	5.26%	1.71%	0.27%	0.09%	gluconeogenesis Jan 31	Slc25a13
					1.00E+00	1.00E+00	0	9	367	21447	0.00%	1.71%	0.00%	0.04%	glycolysis Jan 31	
MP:0001408	stereotypic behavior	2.80E-01	1.00E+00	2.80E-01	1.00E+00	1.00E+00	0	11	367	21447	0.00%	1.71%	0.00%	0.05%	shared jan 31	
					2.80E-01	4.12E-01	2	19	1176	21447	10.53%	5.48%	0.17%	0.09%	gluconeogenesis Jan 31	G6pc3, Slc37a4
					1.00E+00	1.00E+00	0	9	1176	21447	0.00%	5.48%	0.00%	0.04%	glycolysis Jan 31	
MP:0008251	abnormal phagocyte morphology	2.80E-01	1.00E+00	2.80E-01	1.00E+00	1.00E+00	0	11	1176	21447	0.00%	5.48%	0.00%	0.05%	shared jan 31	
					2.80E-01	4.12E-01	1	19	368	21447	5.26%	1.72%	0.27%	0.09%	gluconeogenesis Jan 31	Pcx
					1.00E+00	1.00E+00	0	9	368	21447	0.00%	1.72%	0.00%	0.04%	glycolysis Jan 31	
MP:0003961	decreased lean body mass	2.80E-01	1.00E+00	2.80E-01	1.00E+00	1.00E+00	0	11	368	21447	0.00%	1.72%	0.00%	0.05%	shared jan 31	
					4.37E-01	5.23E-01	1	19	638	21447	5.26%	2.97%	0.16%	0.09%	gluconeogenesis Jan 31	Slc25a13
					1.00E+00	1.00E+00	0	9	638	21447	0.00%	2.97%	0.00%	0.04%	glycolysis Jan 31	
MP:0006335	abnormal hearing electrophysiology	2.83E-01	1.00E+00	2.83E-01	2.83E-01	4.57E-01	1	11	638	21447	9.09%	2.97%	0.16%	0.05%	shared jan 31	Aldoc
					2.85E-01	4.16E-01	1	19	375	21447	5.26%	1.75%	0.27%	0.09%	gluconeogenesis Jan 31	G6pc2
					1.00E+00	1.00E+00	0	9	375	21447	0.00%	1.75%	0.00%	0.04%	glycolysis Jan 31	
MP:0008555	abnormal interferon secretion	2.85E-01	1.00E+00	2.85E-01	1.00E+00	1.00E+00	0	11	375	21447	0.00%	1.75%	0.00%	0.05%	shared jan 31	
					2.86E-01	4.16E-01	1	19	376	21447	5.26%	1.75%	0.27%	0.09%	gluconeogenesis Jan 31	G6pc2
					1.00E+00	1.00E+00	0	9	376	21447	0.00%	1.75%	0.00%	0.04%	glycolysis Jan 31	
MP:0005094	abnormal T cell proliferation	2.86E-01	1.00E+00	2.86E-01	1.00E+00	1.00E+00	0	11	376	21447	0.00%	1.75%	0.00%	0.05%	shared jan 31	
					1.00E+00	1.00E+00	0	19	650	21447	0.00%	3.03%	0.00%	0.09%	gluconeogenesis Jan 31	
					1.00E+00	1.00E+00	0	9	650	21447	0.00%	3.03%	0.00%	0.04%	glycolysis Jan 31	
MP:0001698	decreased embryo size	2.87E-01	1.00E+00	2.87E-01	2.87E-01	4.57E-01	1	11	650	21447	9.09%	3.03%	0.15%	0.05%	shared jan 31	Eno1
					4.45E-01	5.27E-01	1	19	654	21447	5.26%	3.05%	0.15%	0.09%	gluconeogenesis Jan 31	G6pc2

					1.00E+00	1.00E+00	0	9	654	21447	0.00%	3.05%	0.00%	0.04%	glycolysis Jan 31	
MP:0001844	autoimmune response	2.89E-01	1.00E+00	2.89E-01	2.89E-01	4.57E-01	1	11	654	21447	9.09%	3.05%	0.15%	0.05%	shared jan 31	Pgam1
					5.14E-01	5.84E-01	1	19	798	21447	5.26%	3.72%	0.13%	0.09%	gluconeogenesis Jan 31	G6pc3
					2.89E-01	3.52E-01	1	9	798	21447	11.11%	3.72%	0.13%	0.04%	glycolysis Jan 31	Pklr
MP:0001657	abnormal induced morbidity/mortality	2.89E-01	5.14E-01	5.63E-01	3.41E-01	4.87E-01	1	11	798	21447	9.09%	3.72%	0.13%	0.05%	shared jan 31	Aldob
					4.46E-01	5.27E-01	1	19	656	21447	5.26%	3.06%	0.15%	0.09%	gluconeogenesis Jan 31	G6pc2
					1.00E+00	1.00E+00	0	9	656	21447	0.00%	3.06%	0.00%	0.04%	glycolysis Jan 31	
MP:0005005	abnormal self tolerance	2.90E-01	1.00E+00	2.90E-01	2.90E-01	4.57E-01	1	11	656	21447	9.09%	3.06%	0.15%	0.05%	shared jan 31	Pgam1
					5.19E-01	5.88E-01	1	19	809	21447	5.26%	3.77%	0.12%	0.09%	gluconeogenesis Jan 31	G6pc3
					2.93E-01	3.54E-01	1	9	809	21447	11.11%	3.77%	0.12%	0.04%	glycolysis Jan 31	Pklr
MP:0001793	abnormal susceptibility to infection	2.93E-01	1.00E+00	2.93E-01	1.00E+00	1.00E+00	0	11	809	21447	0.00%	3.77%	0.00%	0.05%	shared jan 31	
					2.93E-01	4.22E-01	1	19	388	21447	5.26%	1.81%	0.26%	0.09%	gluconeogenesis Jan 31	Pck1
					1.00E+00	1.00E+00	0	9	388	21447	0.00%	1.81%	0.00%	0.04%	glycolysis Jan 31	
MP:0005265	abnormal blood urea nitrogen level	2.93E-01	1.00E+00	2.93E-01	1.00E+00	1.00E+00	0	11	388	21447	0.00%	1.81%	0.00%	0.05%	shared jan 31	
					2.93E-01	4.22E-01	2	19	1216	21447	10.53%	5.67%	0.16%	0.09%	gluconeogenesis Jan 31	Got2, Mdh2
					4.09E-01	4.60E-01	1	9	1216	21447	11.11%	5.67%	0.08%	0.04%	glycolysis Jan 31	Hk2
MP:0002088	abnormal embryonic growth/weight/body size	2.93E-01	4.74E-01	6.19E-01	4.74E-01	5.55E-01	1	11	1216	21447	9.09%	5.67%	0.08%	0.05%	shared jan 31	Eno1
					2.94E-01	4.22E-01	1	19	389	21447	5.26%	1.81%	0.26%	0.09%	gluconeogenesis Jan 31	G6pc
					1.00E+00	1.00E+00	0	9	389	21447	0.00%	1.81%	0.00%	0.04%	glycolysis Jan 31	
MP:0005325	abnormal renal glomerulus morphology	2.94E-01	1.00E+00	2.94E-01	1.00E+00	1.00E+00	0	11	389	21447	0.00%	1.81%	0.00%	0.05%	shared jan 31	
					1.00E+00	1.00E+00	0	19	670	21447	0.00%	3.12%	0.00%	0.09%	gluconeogenesis Jan 31	
					1.00E+00	1.00E+00	0	9	670	21447	0.00%	3.12%	0.00%	0.04%	glycolysis Jan 31	
MP:0002673	abnormal sperm number	2.95E-01	1.00E+00	2.95E-01	2.95E-01	4.60E-01	1	11	670	21447	9.09%	3.12%	0.15%	0.05%	shared jan 31	Eno4
					2.96E-01	4.24E-01	4	19	3125	21447	21.05%	14.57%	0.13%	0.09%	gluconeogenesis Jan 31	G6pc3, Pcx, Slc25a12, Slc37a4
					3.86E-01	4.39E-01	2	9	3125	21447	22.22%	14.57%	0.06%	0.04%	glycolysis Jan 31	Gck, Hk2
MP:0001259	abnormal body weight	2.96E-01	4.91E-01	6.03E-01	4.91E-01	5.67E-01	2	11	3125	21447	18.18%	14.57%	0.06%	0.05%	shared jan 31	Gpi1, Tpi1
					1.00E+00	1.00E+00	0	19	822	21447	0.00%	3.83%	0.00%	0.09%	gluconeogenesis Jan 31	
					2.97E-01	3.58E-01	1	9	822	21447	11.11%	3.83%	0.12%	0.04%	glycolysis Jan 31	Pfkm
MP:0010630	abnormal cardiac muscle tissue morphology	2.97E-01	1.00E+00	2.97E-01	1.00E+00	1.00E+00	0	11	822	21447	0.00%	3.83%	0.00%	0.05%	shared jan 31	
					2.97E-01	4.24E-01	2	19	1228	21447	10.53%	5.73%	0.16%	0.09%	gluconeogenesis Jan 31	Got1, Slc37a4
					1.00E+00	1.00E+00	0	9	1228	21447	0.00%	5.73%	0.00%	0.04%	glycolysis Jan 31	
MP:0005016	decreased lymphocyte cell number	2.97E-01	1.00E+00	2.97E-01	4.77E-01	5.57E-01	1	11	1228	21447	9.09%	5.73%	0.08%	0.05%	shared jan 31	Pgam1
					1.00E+00	1.00E+00	0	19	678	21447	0.00%	3.16%	0.00%	0.09%	gluconeogenesis Jan 31	
					1.00E+00	1.00E+00	0	9	678	21447	0.00%	3.16%	0.00%	0.04%	glycolysis Jan 31	
MP:0012763	abnormal alpha-beta T cell number	2.98E-01	1.00E+00	2.98E-01	2.98E-01	4.60E-01	1	11	678	21447	9.09%	3.16%	0.15%	0.05%	shared jan 31	Pgam1
					4.57E-01	5.36E-01	1	19	679	21447	5.26%	3.17%	0.15%	0.09%	gluconeogenesis Jan 31	G6pc2
					1.00E+00	1.00E+00	0	9	679	21447	0.00%	3.17%	0.00%	0.04%	glycolysis Jan 31	
MP:0005000	abnormal immune tolerance	2.98E-01	1.00E+00	2.98E-01	2.98E-01	4.60E-01	1	11	679	21447	9.09%	3.17%	0.15%	0.05%	shared jan 31	Pgam1
					1.00E+00	1.00E+00	0	19	691	21447	0.00%	3.22%	0.00%	0.09%	gluconeogenesis Jan 31	
					1.00E+00	1.00E+00	0	9	691	21447	0.00%	3.22%	0.00%	0.04%	glycolysis Jan 31	
MP:0010119	abnormal bone mineral density	3.03E-01	1.00E+00	3.03E-01	3.03E-01	4.64E-01	1	11	691	21447	9.09%	3.22%	0.14%	0.05%	shared jan 31	Aldob
					3.03E-01	4.31E-01	2	19	1246	21447	10.53%	5.81%	0.16%	0.09%	gluconeogenesis Jan 31	Slc25a12, Slc25a13
					1.00E+00	1.00E+00	0	9	1246	21447	0.00%	5.81%	0.00%	0.04%	glycolysis Jan 31	
MP:0001516	abnormal motor coordination/balance	3.03E-01	1.00E+00	3.03E-01	1.00E+00	1.00E+00	0	11	1246	21447	0.00%	5.81%	0.00%	0.05%	shared jan 31	
					1.00E+00	1.00E+00	0	19	694	21447	0.00%	3.24%	0.00%	0.09%	gluconeogenesis Jan 31	
					1.00E+00	1.00E+00	0	9	694	21447	0.00%	3.24%	0.00%	0.04%	glycolysis Jan 31	
MP:0004901	decreased male germ cell number	3.04E-01	1.00E+00	3.04E-01	3.04E-01	4.64E-01	1	11	694	21447	9.09%	3.24%	0.14%	0.05%	shared jan 31	Eno4
					3.05E-01	4.31E-01	1	19	406	21447	5.26%	1.89%	0.25%	0.09%	gluconeogenesis Jan 31	Pcx
					1.00E+00	1.00E+00	0	9	406	21447	0.00%	1.89%	0.00%	0.04%	glycolysis Jan 31	
MP:0010124	decreased bone mineral content	3.05E-01	1.00E+00	3.05E-01	1.00E+00	1.00E+00	0	11	406	21447	0.00%	1.89%	0.00%	0.05%	shared jan 31	
					5.37E-01	6.02E-01	1	19	851	21447	5.26%	3.97%	0.12%	0.09%	gluconeogenesis Jan 31	G6pc3
					3.05E-01	3.67E-01	1	9	851	21447	11.11%	3.97%	0.12%	0.04%	glycolysis Jan 31	Pklr
MP:0005025	abnormal response to infection	3.05E-01	1.00E+00	3.05E-01	1.00E+00	1.00E+00	0	11	851	21447	0.00%	3.97%	0.00%	0.05%	shared jan 31	
					4.20E-01	5.08E-01	3	19	2639	21447	15.79%	12.30%	0.11%	0.09%	gluconeogenesis Jan 31	Got2, Mdh1, Slc37a1
					3.06E-01	3.67E-01	2	9	2639	21447	22.22%	12.30%	0.08%	0.04%	glycolysis Jan 31	Gck, Pfkm
MP:0000266	abnormal heart morphology	3.06E-01	4.20E-01	7.29E-01	4.00E-01	5.12E-01	2	11	2639	21447	18.18%	12.30%	0.08%	0.05%	shared jan 31	Aldob, Gpi1
					3.08E-01	4.35E-01	2	19	1259	21447	10.53%	5.87%	0.16%	0.09%	gluconeogenesis Jan 31	Got2, Mdh2
					4.20E-01	4.71E-01	1	9	1259	21447	11.11%	5.87%	0.08%	0.04%	glycolysis Jan 31	Hk2
MP:0001672	abnormal embryo development	3.08E-01	4.86E-01	6.33E-01	4.86E-01	5.63E-01	1	11	1259	21447	9.09%	5.87%	0.08%	0.05%	shared jan 31	Eno1
					5.42E-01	6.06E-01	1	19	863	21447	5.26%	4.02%	0.12%	0.09%	gluconeogenesis Jan 31	G6pc3
					3.09E-01	3.69E-01	1	9	863	21447	11.11%	4.02%	0.12%	0.04%	glycolysis Jan 31	Pklr
MP:0020000	abnormal response to antigen	3.09E-01	1.00E+00	3.09E-01	1.00E+00	1.00E+00	0	11	863	21447	0.00%	4.02%	0.00%	0.05%	shared jan 31	
					1.00E+00	1.00E+00	0	19	715	21447	0.00%	3.33%	0.00%	0.09%	gluconeogenesis Jan 31	
					1.00E+00	1.00E+00	0	9	715	21447	0.00%	3.33%	0.00%	0.04%	glycolysis Jan 31	
MP:0001943	abnormal respiration	3.11E-01	1.00E+00	3.11E-01	3.11E-01	4.69E-01	1	11	715	21447	9.09%	3.33%	0.14%	0.05%	shared jan 31	Aldoc
					3.13E-01	4.41E-01	1	19	420	21447	5.26%	1.96%	0.24%	0.09%	gluconeogenesis Jan 31	G6pc
					1.00E+00	1.00E+00	0	9	420	21447	0.00%	1.96%	0.00%	0.04%	glycolysis Jan 31	
MP:0002827	abnormal renal corpuscle morphology	3.13E-01	1.00E+00	3.13E-01	1.00E+00	1.00E+00	0	11	420	21447	0.00%	1.96%	0.00%	0.05%	shared jan 31	
					4.78E-01	5.51E-01	1	19	721	21447	5.26%	3.36%	0.14%	0.09%	gluconeogenesis Jan 31	Slc25a13
					1.00E+00	1.00E+00	0	9	721	21447	0.00%	3.36%	0.00%	0.04%	glycolysis Jan 31	

				MP:0001963	abnormal hearing physiology	3.14E-01	1.00E+00	3.14E-01	3.14E-01	4.69E-01	1	11	721	21447	9.09%	3.36%	0.14%	0.05%	shared jan 31	Aldoc
									3.16E-01	4.42E-01	1	19	424	21447	5.26%	1.98%	0.24%	0.09%	gluconeogenesis Jan 31	Pck1
									1.00E+00	1.00E+00	0	9	424	21447	0.00%	1.98%	0.00%	0.04%	glycolysis Jan 31	
				MP:0002970	abnormal white adipose tissue morphology	3.16E-01	1.00E+00	3.16E-01	1.00E+00	1.00E+00	0	11	424	21447	0.00%	1.98%	0.00%	0.05%	shared jan 31	
									3.18E-01	4.42E-01	6	19	5280	21447	31.58%	24.62%	0.11%	0.09%	gluconeogenesis Jan 31	G6pc, Got2, Mdh2, Slc25a12, Slc25a13, Slc37a4
									9.21E-01	9.28E-01	1	9	5280	21447	11.11%	24.62%	0.02%	0.04%	glycolysis Jan 31	Hk2
				MP:0003631	nervous system phenotype	3.18E-01	9.55E-01	3.33E-01	9.55E-01	9.58E-01	1	11	5280	21447	9.09%	24.62%	0.02%	0.05%	shared jan 31	Tpi1
									3.18E-01	4.42E-01	1	19	428	21447	5.26%	2.00%	0.23%	0.09%	gluconeogenesis Jan 31	G6pc3
									1.00E+00	1.00E+00	0	9	428	21447	0.00%	2.00%	0.00%	0.04%	glycolysis Jan 31	
				MP:0005179	decreased circulating cholesterol level	3.18E-01	1.00E+00	3.18E-01	1.00E+00	1.00E+00	0	11	428	21447	0.00%	2.00%	0.00%	0.05%	shared jan 31	
									3.20E-01	4.42E-01	2	19	1294	21447	10.53%	6.03%	0.15%	0.09%	gluconeogenesis Jan 31	Pck2, Slc25a10
									4.29E-01	4.80E-01	1	9	1294	21447	11.11%	6.03%	0.08%	0.04%	glycolysis Jan 31	Gck
				MP:0001570	abnormal circulating enzyme level	3.20E-01	1.00E+00	3.20E-01	1.00E+00	1.00E+00	0	11	1294	21447	0.00%	6.03%	0.00%	0.05%	shared jan 31	
									3.20E-01	4.42E-01	1	19	430	21447	5.26%	2.00%	0.23%	0.09%	gluconeogenesis Jan 31	G6pc2
									1.00E+00	1.00E+00	0	9	430	21447	0.00%	2.00%	0.00%	0.04%	glycolysis Jan 31	
				MP:0001828	abnormal T cell activation	3.20E-01	1.00E+00	3.20E-01	1.00E+00	1.00E+00	0	11	430	21447	0.00%	2.00%	0.00%	0.05%	shared jan 31	
									3.21E-01	4.43E-01	1	19	433	21447	5.26%	2.02%	0.23%	0.09%	gluconeogenesis Jan 31	G6pc
									1.00E+00	1.00E+00	0	9	433	21447	0.00%	2.02%	0.00%	0.04%	glycolysis Jan 31	
				MP:0002989	small kidney	3.21E-01	1.00E+00	3.21E-01	1.00E+00	1.00E+00	0	11	433	21447	0.00%	2.02%	0.00%	0.05%	shared jan 31	
									3.22E-01	4.43E-01	1	19	434	21447	5.26%	2.02%	0.23%	0.09%	gluconeogenesis Jan 31	Pck1
									1.00E+00	1.00E+00	0	9	434	21447	0.00%	2.02%	0.00%	0.04%	glycolysis Jan 31	
				MP:0005311	abnormal circulating amino acid level	3.22E-01	1.00E+00	3.22E-01	1.00E+00	1.00E+00	0	11	434	21447	0.00%	2.02%	0.00%	0.05%	shared jan 31	
									6.99E-01	7.21E-01	3	19	3905	21447	15.79%	18.21%	0.08%	0.09%	gluconeogenesis Jan 31	G6pc2, Pck2, Slc37a2
									5.08E-01	5.42E-01	2	9	3905	21447	22.22%	18.21%	0.05%	0.04%	glycolysis Jan 31	Hk1, Pfkm
				MP:0005389	reproductive system phenotype	3.23E-01	6.99E-01	4.62E-01	3.23E-01	4.76E-01	3	11	3905	21447	27.27%	18.21%	0.08%	0.05%	shared jan 31	Eno4, Gapdhs, Pgk2
									1.00E+00	1.00E+00	0	19	748	21447	0.00%	3.49%	0.00%	0.09%	gluconeogenesis Jan 31	
									1.00E+00	1.00E+00	0	9	748	21447	0.00%	3.49%	0.00%	0.04%	glycolysis Jan 31	
				MP:0002209	decreased germ cell number	3.23E-01	1.00E+00	3.23E-01	3.23E-01	4.76E-01	1	11	748	21447	9.09%	3.49%	0.13%	0.05%	shared jan 31	Eno4
									1.00E+00	1.00E+00	0	19	749	21447	0.00%	3.49%	0.00%	0.09%	gluconeogenesis Jan 31	
									1.00E+00	1.00E+00	0	9	749	21447	0.00%	3.49%	0.00%	0.04%	glycolysis Jan 31	
				MP:0005018	decreased T cell number	3.24E-01	1.00E+00	3.24E-01	3.24E-01	4.76E-01	1	11	749	21447	9.09%	3.49%	0.13%	0.05%	shared jan 31	Pgam1
									1.00E+00	1.00E+00	0	19	917	21447	0.00%	4.28%	0.00%	0.09%	gluconeogenesis Jan 31	
									3.25E-01	3.85E-01	1	9	917	21447	11.11%	4.28%	0.11%	0.04%	glycolysis Jan 31	Gck
				MP:0006207	embryonic lethality during organogenesis	3.25E-01	1.00E+00	3.25E-01	1.00E+00	1.00E+00	0	11	917	21447	0.00%	4.28%	0.00%	0.05%	shared jan 31	
									3.26E-01	4.46E-01	1	19	441	21447	5.26%	2.06%	0.23%	0.09%	gluconeogenesis Jan 31	Pck1
									1.00E+00	1.00E+00	0	9	441	21447	0.00%	2.06%	0.00%	0.04%	glycolysis Jan 31	
				MP:0005334	abnormal fat pad morphology	3.26E-01	1.00E+00	3.26E-01	1.00E+00	1.00E+00	0	11	441	21447	0.00%	2.06%	0.00%	0.05%	shared jan 31	
									3.26E-01	4.46E-01	1	19	441	21447	5.26%	2.06%	0.23%	0.09%	gluconeogenesis Jan 31	Slc25a12
									1.00E+00	1.00E+00	0	9	441	21447	0.00%	2.06%	0.00%	0.04%	glycolysis Jan 31	
				MP:0001513	limb grasping	3.26E-01	1.00E+00	3.26E-01	1.00E+00	1.00E+00	0	11	441	21447	0.00%	2.06%	0.00%	0.05%	shared jan 31	
									1.00E+00	1.00E+00	0	19	760	21447	0.00%	3.54%	0.00%	0.09%	gluconeogenesis Jan 31	
									1.00E+00	1.00E+00	0	9	760	21447	0.00%	3.54%	0.00%	0.04%	glycolysis Jan 31	
				MP:0002401	abnormal lymphopoiesis	3.28E-01	1.00E+00	3.28E-01	3.28E-01	4.78E-01	1	11	760	21447	9.09%	3.54%	0.13%	0.05%	shared jan 31	Pgam1
									5.01E-01	5.74E-01	1	19	771	21447	5.26%	3.59%	0.13%	0.09%	gluconeogenesis Jan 31	Slc25a13
									1.00E+00	1.00E+00	0	9	771	21447	0.00%	3.59%	0.00%	0.04%	glycolysis Jan 31	
				MP:0003878	abnormal ear physiology	3.32E-01	1.00E+00	3.32E-01	3.32E-01	4.81E-01	1	11	771	21447	9.09%	3.59%	0.13%	0.05%	shared jan 31	Aldoc
									5.73E-01	6.23E-01	1	19	940	21447	5.26%	4.38%	0.11%	0.09%	gluconeogenesis Jan 31	Slc37a2
									3.32E-01	3.90E-01	1	9	940	21447	11.11%	4.38%	0.11%	0.04%	glycolysis Jan 31	Pfkm
				MP:0004849	abnormal testis size	3.32E-01	5.73E-01	5.79E-01	3.89E-01	5.07E-01	1	11	940	21447	9.09%	4.38%	0.11%	0.05%	shared jan 31	Eno4
									3.33E-01	4.51E-01	1	19	452	21447	5.26%	2.11%	0.22%	0.09%	gluconeogenesis Jan 31	Pck1
									1.00E+00	1.00E+00	0	9	452	21447	0.00%	2.11%	0.00%	0.04%	glycolysis Jan 31	
				MP:0009644	uremia	3.33E-01	1.00E+00	3.33E-01	1.00E+00	1.00E+00	0	11	452	21447	0.00%	2.11%	0.00%	0.05%	shared jan 31	
									1.00E+00	1.00E+00	0	19	946	21447	0.00%	4.41%	0.00%	0.09%	gluconeogenesis Jan 31	
									3.34E-01	3.91E-01	1	9	946	21447	11.11%	4.41%	0.11%	0.04%	glycolysis Jan 31	Hk1
				MP:0003699	abnormal female reproductive system physiology	3.34E-01	1.00E+00	3.34E-01	1.00E+00	1.00E+00	0	11	946	21447	0.00%	4.41%	0.00%	0.05%	shared jan 31	
									3.34E-01	4.51E-01	1	19	454	21447	5.26%	2.12%	0.22%	0.09%	gluconeogenesis Jan 31	Slc25a12
									1.00E+00	1.00E+00	0	9	454	21447	0.00%	2.12%	0.00%	0.04%	glycolysis Jan 31	
				MP:0020167	abnormal vertical activity	3.34E-01	1.00E+00	3.34E-01	1.00E+00	1.00E+00	0	11	454	21447	0.00%	2.12%	0.00%	0.05%	shared jan 31	
									5.05E-01	5.76E-01	1	19	779	21447	5.26%	3.63%	0.13%	0.09%	gluconeogenesis Jan 31	Slc25a12
									1.00E+00	1.00E+00	0	9	779	21447	0.00%	3.63%	0.00%	0.04%	glycolysis Jan 31	
				MP:0001362	abnormal anxiety-related response	3.34E-01	1.00E+00	3.34E-01	3.34E-01	4.83E-01	1	11	779	21447	9.09%	3.63%	0.13%	0.05%	shared jan 31	Aldoa
									3.35E-01	4.51E-01	1	19	455	21447	5.26%	2.12%	0.22%	0.09%	gluconeogenesis Jan 31	G6pc
									1.00E+00	1.00E+00	0	9	455	21447	0.00%	2.12%	0.00%	0.04%	glycolysis Jan 31	
				MP:0000521	abnormal kidney cortex morphology	3.35E-01	1.00E+00	3.35E-01	1.00E+00	1.00E+00	0	11	455	21447	0.00%	2.12%	0.00%	0.05%	shared jan 31	
									1.00E+00	1.00E+00	0	19	955	21447	0.00%	4.45%	0.00%	0.09%	gluconeogenesis Jan 31	
									3.36E-01	3.93E-01	1	9	955	21447	11.11%	4.45%	0.10%	0.04%	glycolysis Jan 31	Gck
				MP:0002058	neonatal lethality	3.36E-01	1.00E+00	3.36E-01	1.00E+00	1.00E+00	0	11	955	21447	0.00%	4.45%	0.00%	0.05%	shared jan 31	
									3.37E-01	4.52E-01	1	19	458	21447	5.26%	2.14%	0.22%	0.09%	gluconeogenesis Jan 31	G6pc3
									1.00E+00	1.00E+00	0	9	458	21447	0.00%	2.14%	0.00%	0.04%	glycolysis Jan 31	
				MP:0003983	decreased cholesterol level	3.37E-01	1.00E+00	3.37E-01	1.00E+00	1.00E+00	0	11	458	21447	0.00%	2.14%	0.00%	0.05%	shared jan 31	

									3.37E-01	4.52E-01	1	19	458	21447	5.26%	2.14%	0.22%	0.09%	gluconeogenesis Jan 31	G6pc3
									1.00E+00	1.00E+00	0	9	458	21447	0.00%	2.14%	0.00%	0.04%	glycolysis Jan 31	
				MP:0012225	decreased sterol level	3.37E-01	1.00E+00	3.37E-01	1.00E+00	1.00E+00	0	11	458	21447	0.00%	2.14%	0.00%	0.05%	shared jan 31	
									1.00E+00	1.00E+00	0	19	795	21447	0.00%	3.71%	0.00%	0.09%	gluconeogenesis Jan 31	
									1.00E+00	1.00E+00	0	9	795	21447	0.00%	3.71%	0.00%	0.04%	glycolysis Jan 31	
				MP:0011086	postnatal lethality, incomplete penetrance	3.40E-01	1.00E+00	3.40E-01	3.40E-01	4.87E-01	1	11	795	21447	9.09%	3.71%	0.13%	0.05%	shared jan 31	Gpi1
									3.45E-01	4.57E-01	3	19	2343	21447	15.79%	10.92%	0.13%	0.09%	gluconeogenesis Jan 31	Got2, Mdh1, Mdh2
									6.47E-01	6.65E-01	1	9	2343	21447	11.11%	10.92%	0.04%	0.04%	glycolysis Jan 31	Hk2
				MP:0005380	embryo phenotype	3.42E-01	6.47E-01	5.29E-01	3.42E-01	4.87E-01	2	11	2343	21447	18.18%	10.92%	0.09%	0.05%	shared jan 31	Eno1, Gpi1
									3.43E-01	4.57E-01	3	19	2335	21447	15.79%	10.89%	0.13%	0.09%	gluconeogenesis Jan 31	G6pc2, Got1, Slc37a4
									1.00E+00	1.00E+00	0	9	2335	21447	0.00%	10.89%	0.00%	0.04%	glycolysis Jan 31	
				MP:0008247	abnormal mononuclear cell morphology	3.43E-01	1.00E+00	3.43E-01	7.19E-01	7.52E-01	1	11	2335	21447	9.09%	10.89%	0.04%	0.05%	shared jan 31	Pgam1
									3.44E-01	4.57E-01	1	19	470	21447	5.26%	2.19%	0.21%	0.09%	gluconeogenesis Jan 31	G6pc
									1.00E+00	1.00E+00	0	9	470	21447	0.00%	2.19%	0.00%	0.04%	glycolysis Jan 31	
				MP:0002932	abnormal joint morphology	3.44E-01	1.00E+00	3.44E-01	1.00E+00	1.00E+00	0	11	470	21447	0.00%	2.19%	0.00%	0.05%	shared jan 31	
									3.44E-01	4.57E-01	3	19	2342	21447	15.79%	10.92%	0.13%	0.09%	gluconeogenesis Jan 31	Got2, Mdh2, Slc25a12
									6.47E-01	6.65E-01	1	9	2342	21447	11.11%	10.92%	0.04%	0.04%	glycolysis Jan 31	Hk2
				MP:0002152	abnormal brain morphology	3.44E-01	1.00E+00	3.44E-01	1.00E+00	1.00E+00	0	11	2342	21447	0.00%	10.92%	0.00%	0.05%	shared jan 31	
									3.46E-01	4.57E-01	1	19	473	21447	5.26%	2.21%	0.21%	0.09%	gluconeogenesis Jan 31	Got1
									1.00E+00	1.00E+00	0	9	473	21447	0.00%	2.21%	0.00%	0.04%	glycolysis Jan 31	
				MP:0001629	abnormal heart rate	3.46E-01	1.00E+00	3.46E-01	1.00E+00	1.00E+00	0	11	473	21447	0.00%	2.21%	0.00%	0.05%	shared jan 31	
									1.00E+00	1.00E+00	0	19	811	21447	0.00%	3.78%	0.00%	0.09%	gluconeogenesis Jan 31	
									1.00E+00	1.00E+00	0	9	811	21447	0.00%	3.78%	0.00%	0.04%	glycolysis Jan 31	
				MP:0001175	abnormal lung morphology	3.46E-01	1.00E+00	3.46E-01	3.46E-01	4.88E-01	1	11	811	21447	9.09%	3.78%	0.12%	0.05%	shared jan 31	Gpi1
									3.47E-01	4.57E-01	1	19	475	21447	5.26%	2.21%	0.21%	0.09%	gluconeogenesis Jan 31	Slc37a2
									1.00E+00	1.00E+00	0	9	475	21447	0.00%	2.21%	0.00%	0.04%	glycolysis Jan 31	
				MP:0002451	abnormal macrophage physiology	3.47E-01	1.00E+00	3.47E-01	1.00E+00	1.00E+00	0	11	475	21447	0.00%	2.21%	0.00%	0.05%	shared jan 31	
									3.49E-01	4.59E-01	1	19	479	21447	5.26%	2.23%	0.21%	0.09%	gluconeogenesis Jan 31	Slc25a12
									1.00E+00	1.00E+00	0	9	479	21447	0.00%	2.23%	0.00%	0.04%	glycolysis Jan 31	
				MP:0009745	abnormal behavioral response to xenobiotic	3.49E-01	1.00E+00	3.49E-01	1.00E+00	1.00E+00	0	11	479	21447	0.00%	2.23%	0.00%	0.05%	shared jan 31	
									5.25E-01	5.94E-01	1	19	824	21447	5.26%	3.84%	0.12%	0.09%	gluconeogenesis Jan 31	G6pc2
									1.00E+00	1.00E+00	0	9	824	21447	0.00%	3.84%	0.00%	0.04%	glycolysis Jan 31	
				MP:0002435	abnormal effector T cell morphology	3.50E-01	1.00E+00	3.50E-01	3.50E-01	4.88E-01	1	11	824	21447	9.09%	3.84%	0.12%	0.05%	shared jan 31	Pgam1
									4.86E-01	5.58E-01	3	19	2909	21447	15.79%	13.56%	0.10%	0.09%	gluconeogenesis Jan 31	G6pc3, Slc25a12, Slc37a4
									3.50E-01	4.07E-01	2	9	2909	21447	22.22%	13.56%	0.07%	0.04%	glycolysis Jan 31	Gck, Hk2
				MP:0012321	abnormal total tissue mass	3.50E-01	4.86E-01	7.20E-01	4.51E-01	5.46E-01	2	11	2909	21447	18.18%	13.56%	0.07%	0.05%	shared jan 31	Gpi1, Tpi1
									1.00E+00	1.00E+00	0	19	838	21447	0.00%	3.91%	0.00%	0.09%	gluconeogenesis Jan 31	
									1.00E+00	1.00E+00	0	9	838	21447	0.00%	3.91%	0.00%	0.04%	glycolysis Jan 31	
				MP:0001124	abnormal gametes	3.55E-01	1.00E+00	3.55E-01	3.55E-01	4.89E-01	1	11	838	21447	9.09%	3.91%	0.12%	0.05%	shared jan 31	Eno4
									5.33E-01	6.00E-01	1	19	843	21447	5.26%	3.93%	0.12%	0.09%	gluconeogenesis Jan 31	G6pc2
									1.00E+00	1.00E+00	0	9	843	21447	0.00%	3.93%	0.00%	0.04%	glycolysis Jan 31	
				MP:0012762	abnormal alpha-beta T cell morphology	3.57E-01	1.00E+00	3.57E-01	3.57E-01	4.89E-01	1	11	843	21447	9.09%	3.93%	0.12%	0.05%	shared jan 31	Pgam1
									3.57E-01	4.66E-01	1	19	493	21447	5.26%	2.30%	0.20%	0.09%	gluconeogenesis Jan 31	Slc25a12
									1.00E+00	1.00E+00	0	9	493	21447	0.00%	2.30%	0.00%	0.04%	glycolysis Jan 31	
				MP:0002200	abnormal brain ventricular system morphology	3.57E-01	1.00E+00	3.57E-01	1.00E+00	1.00E+00	0	11	493	21447	0.00%	2.30%	0.00%	0.05%	shared jan 31	
									1.00E+00	1.00E+00	0	19	850	21447	0.00%	3.96%	0.00%	0.09%	gluconeogenesis Jan 31	
									1.00E+00	1.00E+00	0	9	850	21447	0.00%	3.96%	0.00%	0.04%	glycolysis Jan 31	
				MP:0001147	small testis	3.59E-01	1.00E+00	3.59E-01	3.59E-01	4.90E-01	1	11	850	21447	9.09%	3.96%	0.12%	0.05%	shared jan 31	Eno4
									3.65E-01	4.68E-01	1	19	506	21447	5.26%	2.36%	0.20%	0.09%	gluconeogenesis Jan 31	Slc25a12
									1.00E+00	1.00E+00	0	9	506	21447	0.00%	2.36%	0.00%	0.04%	glycolysis Jan 31	
				MP:0000745	tremors	3.65E-01	1.00E+00	3.65E-01	1.00E+00	1.00E+00	0	11	506	21447	0.00%	2.36%	0.00%	0.05%	shared jan 31	
									3.66E-01	4.69E-01	2	19	1435	21447	10.53%	6.69%	0.14%	0.09%	gluconeogenesis Jan 31	Got2, Mdh2
									4.64E-01	5.08E-01	1	9	1435	21447	11.11%	6.69%	0.07%	0.04%	glycolysis Jan 31	Hk2
				MP:0004196	abnormal prenatal growth/weight/body size	3.66E-01	5.33E-01	6.87E-01	5.33E-01	5.98E-01	1	11	1435	21447	9.09%	6.69%	0.07%	0.05%	shared jan 31	Eno1
									3.68E-01	4.70E-01	2	19	1441	21447	10.53%	6.72%	0.14%	0.09%	gluconeogenesis Jan 31	Slc25a10, Slc25a12
									1.00E+00	1.00E+00	0	9	1441	21447	0.00%	6.72%	0.00%	0.04%	glycolysis Jan 31	
				MP:0001961	abnormal reflex	3.68E-01	1.00E+00	3.68E-01	1.00E+00	1.00E+00	0	11	1441	21447	0.00%	6.72%	0.00%	0.05%	shared jan 31	
									3.70E-01	4.70E-01	2	19	1445	21447	10.53%	6.74%	0.14%	0.09%	gluconeogenesis Jan 31	Pck2, Slc37a2
									4.66E-01	5.09E-01	1	9	1445	21447	11.11%	6.74%	0.07%	0.04%	glycolysis Jan 31	Pfkm
				MP:0009205	abnormal internal male genitalia morphology	3.70E-01	5.36E-01	6.90E-01	5.36E-01	5.98E-01	1	11	1445	21447	9.09%	6.74%	0.07%	0.05%	shared jan 31	Eno4
									3.71E-01	4.70E-01	2	19	1450	21447	10.53%	6.76%	0.14%	0.09%	gluconeogenesis Jan 31	Pcx, Slc37a4
									1.00E+00	1.00E+00	0	9	1450	21447	0.00%	6.76%	0.00%	0.04%	glycolysis Jan 31	
				MP:0003795	abnormal bone structure	3.71E-01	1.00E+00	3.71E-01	5.37E-01	5.98E-01	1	11	1450	21447	9.09%	6.76%	0.07%	0.05%	shared jan 31	Aldob
									3.71E-01	4.70E-01	2	19	1450	21447	10.53%	6.76%	0.14%	0.09%	gluconeogenesis Jan 31	Got2, Mdh2
									4.67E-01	5.09E-01	1	9	1450	21447	11.11%	6.76%	0.07%	0.04%	glycolysis Jan 31	Hk2
				MP:0003861	abnormal nervous system development	3.71E-01	1.00E+00	3.71E-01	1.00E+00	1.00E+00	0	11	1450	21447	0.00%	6.76%	0.00%	0.05%	shared jan 31	
									5.54E-01	6.09E-01	1	19	891	21447	5.26%	4.15%	0.11%	0.09%	gluconeogenesis Jan 31	Slc37a4
									1.00E+00	1.00E+00	0	9	891	21447	0.00%	4.15%	0.00%	0.04%	glycolysis Jan 31	
				MP:0005460	abnormal leukopoiesis	3.73E-01	1.00E+00	3.73E-01	3.73E-01	5.00E-01	1	11	891	21447	9.09%	4.15%	0.11%	0.05%	shared jan 31	Pgam1
									3.78E-01	4.76E-01	2	19	1471	21447	10.53%	6.86%	0.14%	0.09%	gluconeogenesis Jan 31	Pck2, Slc37a2

					4.72E-01	5.12E-01	1	9	1471	21447	11.11%	6.86%	0.07%	0.04%	glycolysis Jan 31	Pfkm
MP:0009198	abnormal male genitalia morphology	3.78E-01	5.42E-01	6.97E-01	5.42E-01	5.99E-01	1	11	1471	21447	9.09%	6.86%	0.07%	0.05%	shared jan 31	Eno4
					3.78E-01	4.76E-01	2	19	1471	21447	10.53%	6.86%	0.14%	0.09%	gluconeogenesis Jan 31	Pck2, Slc37a2
					4.72E-01	5.12E-01	1	9	1471	21447	11.11%	6.86%	0.07%	0.04%	glycolysis Jan 31	Pfkm
MP:0001145	abnormal male reproductive system morphology	3.78E-01	5.42E-01	6.97E-01	5.42E-01	5.99E-01	1	11	1471	21447	9.09%	6.86%	0.07%	0.05%	shared jan 31	Eno4
					5.62E-01	6.16E-01	1	19	911	21447	5.26%	4.25%	0.11%	0.09%	gluconeogenesis Jan 31	Mdh2
					1.00E+00	1.00E+00	0	9	911	21447	0.00%	4.25%	0.00%	0.04%	glycolysis Jan 31	
MP:0001697	abnormal embryo size	3.80E-01	1.00E+00	3.80E-01	3.80E-01	5.07E-01	1	11	911	21447	9.09%	4.25%	0.11%	0.05%	shared jan 31	Eno1
					6.35E-01	6.77E-01	1	19	1109	21447	5.26%	5.17%	0.09%	0.09%	gluconeogenesis Jan 31	Slc37a2
					3.80E-01	4.35E-01	1	9	1109	21447	11.11%	5.17%	0.09%	0.04%	glycolysis Jan 31	Pfkm
MP:0001146	abnormal testis morphology	3.80E-01	6.35E-01	5.98E-01	4.42E-01	5.46E-01	1	11	1109	21447	9.09%	5.17%	0.09%	0.05%	shared jan 31	Eno4
					3.82E-01	4.80E-01	1	19	537	21447	5.26%	2.50%	0.19%	0.09%	gluconeogenesis Jan 31	Got1
					1.00E+00	1.00E+00	0	9	537	21447	0.00%	2.50%	0.00%	0.04%	glycolysis Jan 31	
MP:0004085	abnormal heartbeat	3.82E-01	1.00E+00	3.82E-01	1.00E+00	1.00E+00	0	11	537	21447	0.00%	2.50%	0.00%	0.05%	shared jan 31	
					5.67E-01	6.20E-01	1	19	925	21447	5.26%	4.31%	0.11%	0.09%	gluconeogenesis Jan 31	Slc37a4
					1.00E+00	1.00E+00	0	9	925	21447	0.00%	4.31%	0.00%	0.04%	glycolysis Jan 31	
MP:0020981	abnormal immune system development	3.84E-01	1.00E+00	3.84E-01	3.84E-01	5.07E-01	1	11	925	21447	9.09%	4.31%	0.11%	0.05%	shared jan 31	Pgam1
					1.00E+00	1.00E+00	0	19	926	21447	0.00%	4.32%	0.00%	0.09%	gluconeogenesis Jan 31	
					1.00E+00	1.00E+00	0	9	926	21447	0.00%	4.32%	0.00%	0.04%	glycolysis Jan 31	
MP:0001116	small gonad	3.85E-01	1.00E+00	3.85E-01	3.85E-01	5.07E-01	1	11	926	21447	9.09%	4.32%	0.11%	0.05%	shared jan 31	Eno4
					3.85E-01	4.82E-01	1	19	542	21447	5.26%	2.53%	0.18%	0.09%	gluconeogenesis Jan 31	Pck1
					1.00E+00	1.00E+00	0	9	542	21447	0.00%	2.53%	0.00%	0.04%	glycolysis Jan 31	
MP:0005449	abnormal food intake	3.85E-01	1.00E+00	3.85E-01	1.00E+00	1.00E+00	0	11	542	21447	0.00%	2.53%	0.00%	0.05%	shared jan 31	
					1.00E+00	1.00E+00	0	19	1130	21447	0.00%	5.27%	0.00%	0.09%	gluconeogenesis Jan 31	
					3.86E-01	4.39E-01	1	9	1130	21447	11.11%	5.27%	0.09%	0.04%	glycolysis Jan 31	Pfkm
MP:0013558	abnormal exocrine gland morphology	3.86E-01	1.00E+00	3.86E-01	1.00E+00	1.00E+00	0	11	1130	21447	0.00%	5.27%	0.00%	0.05%	shared jan 31	
					1.00E+00	1.00E+00	0	19	930	21447	0.00%	4.34%	0.00%	0.09%	gluconeogenesis Jan 31	
					1.00E+00	1.00E+00	0	9	930	21447	0.00%	4.34%	0.00%	0.04%	glycolysis Jan 31	
MP:0006362	abnormal male germ cell morphology	3.86E-01	1.00E+00	3.86E-01	3.86E-01	5.07E-01	1	11	930	21447	9.09%	4.34%	0.11%	0.05%	shared jan 31	Eno4
					5.70E-01	6.20E-01	1	19	931	21447	5.26%	4.34%	0.11%	0.09%	gluconeogenesis Jan 31	Slc25a12
					1.00E+00	1.00E+00	0	9	931	21447	0.00%	4.34%	0.00%	0.04%	glycolysis Jan 31	
MP:0002065	abnormal fear/anxiety-related behavior	3.86E-01	1.00E+00	3.86E-01	3.86E-01	5.07E-01	1	11	931	21447	9.09%	4.34%	0.11%	0.05%	shared jan 31	Aldoa
					6.45E-01	6.86E-01	1	19	1138	21447	5.26%	5.31%	0.09%	0.09%	gluconeogenesis Jan 31	Mdh1
					3.88E-01	4.40E-01	1	9	1138	21447	11.11%	5.31%	0.09%	0.04%	glycolysis Jan 31	Pfkm
MP:0005294	abnormal heart ventricle morphology	3.88E-01	6.45E-01	6.01E-01	4.51E-01	5.46E-01	1	11	1138	21447	9.09%	5.31%	0.09%	0.05%	shared jan 31	Aldob
					1.00E+00	1.00E+00	0	19	950	21447	0.00%	4.43%	0.00%	0.09%	gluconeogenesis Jan 31	
					1.00E+00	1.00E+00	0	9	950	21447	0.00%	4.43%	0.00%	0.04%	glycolysis Jan 31	
MP:0002211	abnormal primary sex determination	3.93E-01	1.00E+00	3.93E-01	3.93E-01	5.07E-01	1	11	950	21447	9.09%	4.43%	0.11%	0.05%	shared jan 31	Eno4
					3.94E-01	4.92E-01	3	19	2537	21447	15.79%	11.83%	0.12%	0.09%	gluconeogenesis Jan 31	Pck1, Slc25a12, Slc37a4
					6.78E-01	6.90E-01	1	9	2537	21447	11.11%	11.83%	0.04%	0.04%	glycolysis Jan 31	Gck
MP:0010832	lethality during fetal growth through weaning	3.94E-01	7.50E-01	5.26E-01	7.50E-01	7.79E-01	1	11	2537	21447	9.09%	11.83%	0.04%	0.05%	shared jan 31	Gpi1
					1.00E+00	1.00E+00	0	19	972	21447	0.00%	4.53%	0.00%	0.09%	gluconeogenesis Jan 31	
					1.00E+00	1.00E+00	0	9	972	21447	0.00%	4.53%	0.00%	0.04%	glycolysis Jan 31	
MP:0001921	reduced fertility	4.00E-01	1.00E+00	4.00E-01	4.00E-01	5.12E-01	1	11	972	21447	9.09%	4.53%	0.10%	0.05%	shared jan 31	Pgk2
					1.00E+00	1.00E+00	0	19	999	21447	0.00%	4.66%	0.00%	0.09%	gluconeogenesis Jan 31	
					1.00E+00	1.00E+00	0	9	999	21447	0.00%	4.66%	0.00%	0.04%	glycolysis Jan 31	
MP:0002210	abnormal sex determination	4.08E-01	1.00E+00	4.08E-01	4.08E-01	5.21E-01	1	11	999	21447	9.09%	4.66%	0.10%	0.05%	shared jan 31	Eno4
					4.15E-01	5.08E-01	2	19	1586	21447	10.53%	7.39%	0.13%	0.09%	gluconeogenesis Jan 31	G6pc2, Slc37a2
					1.00E+00	1.00E+00	0	9	1586	21447	0.00%	7.39%	0.00%	0.04%	glycolysis Jan 31	
MP:0001846	increased inflammatory response	4.15E-01	1.00E+00	4.15E-01	5.71E-01	6.17E-01	1	11	1586	21447	9.09%	7.39%	0.06%	0.05%	shared jan 31	Aldob
					4.15E-01	5.08E-01	4	19	3678	21447	21.05%	17.15%	0.11%	0.09%	gluconeogenesis Jan 31	G6pc2, G6pc3, Slc37a2, Slc37a4
					8.16E-01	8.26E-01	1	9	3678	21447	11.11%	17.15%	0.03%	0.04%	glycolysis Jan 31	Pklr
MP:0001790	abnormal immune system physiology	4.15E-01	8.16E-01	5.09E-01	5.86E-01	6.28E-01	2	11	3678	21447	18.18%	17.15%	0.05%	0.05%	shared jan 31	Aldob, Pgam1
					4.16E-01	5.08E-01	1	19	598	21447	5.26%	2.79%	0.17%	0.09%	gluconeogenesis Jan 31	Slc25a13
					1.00E+00	1.00E+00	0	9	598	21447	0.00%	2.79%	0.00%	0.04%	glycolysis Jan 31	
MP:0002102	abnormal ear morphology	4.16E-01	1.00E+00	4.16E-01	1.00E+00	1.00E+00	0	11	598	21447	0.00%	2.79%	0.00%	0.05%	shared jan 31	
					4.17E-01	5.08E-01	1	19	600	21447	5.26%	2.80%	0.17%	0.09%	gluconeogenesis Jan 31	Slc25a10
					1.00E+00	1.00E+00	0	9	600	21447	0.00%	2.80%	0.00%	0.04%	glycolysis Jan 31	
MP:0001486	abnormal startle reflex	4.17E-01	1.00E+00	4.17E-01	1.00E+00	1.00E+00	0	11	600	21447	0.00%	2.80%	0.00%	0.05%	shared jan 31	
					1.00E+00	1.00E+00	0	19	1032	21447	0.00%	4.81%	0.00%	0.09%	gluconeogenesis Jan 31	
					1.00E+00	1.00E+00	0	9	1032	21447	0.00%	4.81%	0.00%	0.04%	glycolysis Jan 31	
MP:0002208	abnormal germ cell morphology	4.19E-01	1.00E+00	4.19E-01	4.19E-01	5.30E-01	1	11	1032	21447	9.09%	4.81%	0.10%	0.05%	shared jan 31	Eno4
					4.19E-01	5.08E-01	5	19	4764	21447	26.32%	22.21%	0.10%	0.09%	gluconeogenesis Jan 31	Pck1, Pcx, Slc25a10, Slc25a12, Slc25a13
					1.00E+00	1.00E+00	0	9	4764	21447	0.00%	22.21%	0.00%	0.04%	glycolysis Jan 31	
MP:0002066	abnormal motor capabilities/coordination/movement	4.19E-01	1.00E+00	4.19E-01	9.37E-01	9.43E-01	1	11	4764	21447	9.09%	22.21%	0.02%	0.05%	shared jan 31	Aldoc
					1.00E+00	1.00E+00	0	19	1033	21447	0.00%	4.82%	0.00%	0.09%	gluconeogenesis Jan 31	
					1.00E+00	1.00E+00	0	9	1033	21447	0.00%	4.82%	0.00%	0.04%	glycolysis Jan 31	
MP:0001156	abnormal spermatogenesis	4.19E-01	1.00E+00	4.19E-01	4.19E-01	5.30E-01	1	11	1033	21447	9.09%	4.82%	0.10%	0.05%	shared jan 31	Eno4
					4.20E-01	5.08E-01	1	19	605	21447	5.26%	2.82%	0.17%	0.09%	gluconeogenesis Jan 31	G6pc
					1.00E+00	1.00E+00	0	9	605	21447	0.00%	2.82%	0.00%	0.04%	glycolysis Jan 31	

				MP:0003881	abnormal nephron morphology	4.20E-01	1.00E+00	4.20E-01	1.00E+00	1.00E+00	0	11	605	21447	0.00%	2.82%	0.00%	0.05%	shared jan 31	
									4.20E-01	5.08E-01	1	19	605	21447	5.26%	2.82%	0.17%	0.09%	gluconeogenesis Jan 31	G6pc3
									1.00E+00	1.00E+00	0	9	605	21447	0.00%	2.82%	0.00%	0.04%	glycolysis Jan 31	
				MP:0000322	increased granulocyte number	4.20E-01	1.00E+00	4.20E-01	1.00E+00	1.00E+00	0	11	605	21447	0.00%	2.82%	0.00%	0.05%	shared jan 31	
									1.00E+00	1.00E+00	0	19	1067	21447	0.00%	4.98%	0.00%	0.09%	gluconeogenesis Jan 31	
									1.00E+00	1.00E+00	0	9	1067	21447	0.00%	4.98%	0.00%	0.04%	glycolysis Jan 31	
				MP:0006387	abnormal T cell number	4.30E-01	1.00E+00	4.30E-01	4.30E-01	5.39E-01	1	11	1067	21447	9.09%	4.98%	0.09%	0.05%	shared jan 31	Pgam1
									4.35E-01	5.22E-01	1	19	634	21447	5.26%	2.96%	0.16%	0.09%	gluconeogenesis Jan 31	Pcx
									1.00E+00	1.00E+00	0	9	634	21447	0.00%	2.96%	0.00%	0.04%	glycolysis Jan 31	
				MP:0010122	abnormal bone mineral content	4.35E-01	1.00E+00	4.35E-01	1.00E+00	1.00E+00	0	11	634	21447	0.00%	2.96%	0.00%	0.05%	shared jan 31	
									6.28E-01	6.71E-01	1	19	1086	21447	5.26%	5.06%	0.09%	0.09%	gluconeogenesis Jan 31	Mdh2
									1.00E+00	1.00E+00	0	9	1086	21447	0.00%	5.06%	0.00%	0.04%	glycolysis Jan 31	
				MP:0010866	abnormal prenatal body size	4.35E-01	1.00E+00	4.35E-01	4.35E-01	5.43E-01	1	11	1086	21447	9.09%	5.06%	0.09%	0.05%	shared jan 31	Eno1
									1.00E+00	1.00E+00	0	19	1088	21447	0.00%	5.07%	0.00%	0.09%	gluconeogenesis Jan 31	
									1.00E+00	1.00E+00	0	9	1088	21447	0.00%	5.07%	0.00%	0.04%	glycolysis Jan 31	
				MP:0002133	abnormal respiratory system physiology	4.36E-01	1.00E+00	4.36E-01	4.36E-01	5.43E-01	1	11	1088	21447	9.09%	5.07%	0.09%	0.05%	shared jan 31	Aldoc
									4.40E-01	5.27E-01	2	19	1665	21447	10.53%	7.76%	0.12%	0.09%	gluconeogenesis Jan 31	Slc25a10, Slc25a12
									1.00E+00	1.00E+00	0	9	1665	21447	0.00%	7.76%	0.00%	0.04%	glycolysis Jan 31	
				MP:0002067	abnormal sensory capabilities/reflexes/nociception	4.40E-01	1.00E+00	4.40E-01	1.00E+00	1.00E+00	0	11	1665	21447	0.00%	7.76%	0.00%	0.05%	shared jan 31	
									7.07E-01	7.27E-01	1	19	1342	21447	5.26%	6.26%	0.07%	0.09%	gluconeogenesis Jan 31	Slc37a2
									4.41E-01	4.89E-01	1	9	1342	21447	11.11%	6.26%	0.07%	0.04%	glycolysis Jan 31	Pfkm
				MP:0013325	abnormal male reproductive gland morphology	4.41E-01	7.07E-01	6.24E-01	5.09E-01	5.79E-01	1	11	1342	21447	9.09%	6.26%	0.07%	0.05%	shared jan 31	Eno4
									4.43E-01	5.27E-01	1	19	650	21447	5.26%	3.03%	0.15%	0.09%	gluconeogenesis Jan 31	Slc25a12
									1.00E+00	1.00E+00	0	9	650	21447	0.00%	3.03%	0.00%	0.04%	glycolysis Jan 31	
				MP:0001405	impaired coordination	4.43E-01	1.00E+00	4.43E-01	1.00E+00	1.00E+00	0	11	650	21447	0.00%	3.03%	0.00%	0.05%	shared jan 31	
									1.00E+00	1.00E+00	0	19	1350	21447	0.00%	6.29%	0.00%	0.09%	gluconeogenesis Jan 31	
									4.43E-01	4.90E-01	1	9	1350	21447	11.11%	6.29%	0.07%	0.04%	glycolysis Jan 31	Pfkm
				MP:0000274	enlarged heart	4.43E-01	1.00E+00	4.43E-01	5.11E-01	5.79E-01	1	11	1350	21447	9.09%	6.29%	0.07%	0.05%	shared jan 31	Gpi1
									4.44E-01	5.27E-01	3	19	2737	21447	15.79%	12.76%	0.11%	0.09%	gluconeogenesis Jan 31	G6pc, Slc25a12, Slc37a4
									7.07E-01	7.18E-01	1	9	2737	21447	11.11%	12.76%	0.04%	0.04%	glycolysis Jan 31	Hk2
				MP:0003633	abnormal nervous system physiology	4.44E-01	1.00E+00	4.44E-01	1.00E+00	1.00E+00	0	11	2737	21447	0.00%	12.76%	0.00%	0.05%	shared jan 31	
									4.49E-01	5.30E-01	2	19	1694	21447	10.53%	7.90%	0.12%	0.09%	gluconeogenesis Jan 31	Got1, Slc37a4
									1.00E+00	1.00E+00	0	9	1694	21447	0.00%	7.90%	0.00%	0.04%	glycolysis Jan 31	
				MP:0000717	abnormal lymphocyte cell number	4.49E-01	1.00E+00	4.49E-01	5.96E-01	6.36E-01	1	11	1694	21447	9.09%	7.90%	0.06%	0.05%	shared jan 31	Pgam1
									1.00E+00	1.00E+00	0	19	1382	21447	0.00%	6.44%	0.00%	0.09%	gluconeogenesis Jan 31	
									4.51E-01	4.98E-01	1	9	1382	21447	11.11%	6.44%	0.07%	0.04%	glycolysis Jan 31	Gck
				MP:0002081	perinatal lethality	4.51E-01	1.00E+00	4.51E-01	1.00E+00	1.00E+00	0	11	1382	21447	0.00%	6.44%	0.00%	0.05%	shared jan 31	
									4.54E-01	5.34E-01	2	19	1708	21447	10.53%	7.96%	0.12%	0.09%	gluconeogenesis Jan 31	Fbp2, G6pc3
									1.00E+00	1.00E+00	0	9	1708	21447	0.00%	7.96%	0.00%	0.04%	glycolysis Jan 31	
				MP:0000218	increased leukocyte cell number	4.54E-01	1.00E+00	4.54E-01	1.00E+00	1.00E+00	0	11	1708	21447	0.00%	7.96%	0.00%	0.05%	shared jan 31	
									1.00E+00	1.00E+00	0	19	1420	21447	0.00%	6.62%	0.00%	0.09%	gluconeogenesis Jan 31	
									4.60E-01	5.05E-01	1	9	1420	21447	11.11%	6.62%	0.07%	0.04%	glycolysis Jan 31	Pfkm
				MP:0002106	abnormal muscle physiology	4.60E-01	1.00E+00	4.60E-01	1.00E+00	1.00E+00	0	11	1420	21447	0.00%	6.62%	0.00%	0.05%	shared jan 31	
									4.61E-01	5.38E-01	1	19	686	21447	5.26%	3.20%	0.15%	0.09%	gluconeogenesis Jan 31	Got2
									1.00E+00	1.00E+00	0	9	686	21447	0.00%	3.20%	0.00%	0.04%	glycolysis Jan 31	
				MP:0001914	hemorrhage	4.61E-01	1.00E+00	4.61E-01	1.00E+00	1.00E+00	0	11	686	21447	0.00%	3.20%	0.00%	0.05%	shared jan 31	
									6.60E-01	6.92E-01	1	19	1182	21447	5.26%	5.51%	0.08%	0.09%	gluconeogenesis Jan 31	G6pc2
									1.00E+00	1.00E+00	0	9	1182	21447	0.00%	5.51%	0.00%	0.04%	glycolysis Jan 31	
				MP:0008037	abnormal T cell morphology	4.64E-01	1.00E+00	4.64E-01	4.64E-01	5.54E-01	1	11	1182	21447	9.09%	5.51%	0.08%	0.05%	shared jan 31	Pgam1
									4.66E-01	5.42E-01	1	19	697	21447	5.26%	3.25%	0.14%	0.09%	gluconeogenesis Jan 31	Slc25a12
									1.00E+00	1.00E+00	0	9	697	21447	0.00%	3.25%	0.00%	0.04%	glycolysis Jan 31	
				MP:0001406	abnormal gait	4.66E-01	1.00E+00	4.66E-01	1.00E+00	1.00E+00	0	11	697	21447	0.00%	3.25%	0.00%	0.05%	shared jan 31	
									4.68E-01	5.43E-01	2	19	1754	21447	10.53%	8.18%	0.11%	0.09%	gluconeogenesis Jan 31	Slc25a10, Slc25a12
									1.00E+00	1.00E+00	0	9	1754	21447	0.00%	8.18%	0.00%	0.04%	glycolysis Jan 31	
				MP:0003492	abnormal involuntary movement	4.68E-01	1.00E+00	4.68E-01	1.00E+00	1.00E+00	0	11	1754	21447	0.00%	8.18%	0.00%	0.05%	shared jan 31	
									6.64E-01	6.96E-01	1	19	1196	21447	5.26%	5.58%	0.08%	0.09%	gluconeogenesis Jan 31	Slc25a13
									1.00E+00	1.00E+00	0	9	1196	21447	0.00%	5.58%	0.00%	0.04%	glycolysis Jan 31	
				MP:0005377	hearing/vestibular/ear phenotype	4.68E-01	1.00E+00	4.68E-01	4.68E-01	5.54E-01	1	11	1196	21447	9.09%	5.58%	0.08%	0.05%	shared jan 31	Aldoc
									4.69E-01	5.43E-01	1	19	702	21447	5.26%	3.27%	0.14%	0.09%	gluconeogenesis Jan 31	Pck1
									1.00E+00	1.00E+00	0	9	702	21447	0.00%	3.27%	0.00%	0.04%	glycolysis Jan 31	
				MP:0001431	abnormal eating behavior	4.69E-01	1.00E+00	4.69E-01	1.00E+00	1.00E+00	0	11	702	21447	0.00%	3.27%	0.00%	0.05%	shared jan 31	
									1.00E+00	1.00E+00	0	19	1217	21447	0.00%	5.67%	0.00%	0.09%	gluconeogenesis Jan 31	
									1.00E+00	1.00E+00	0	9	1217	21447	0.00%	5.67%	0.00%	0.04%	glycolysis Jan 31	
				MP:0001929	abnormal gametogenesis	4.74E-01	1.00E+00	4.74E-01	4.74E-01	5.55E-01	1	11	1217	21447	9.09%	5.67%	0.08%	0.05%	shared jan 31	Eno4
									4.78E-01	5.51E-01	2	19	1787	21447	10.53%	8.33%	0.11%	0.09%	gluconeogenesis Jan 31	Got2, Mdh1
									1.00E+00	1.00E+00	0	9	1787	21447	0.00%	8.33%	0.00%	0.04%	glycolysis Jan 31	
				MP:0000428	abnormal craniofacial morphology	4.78E-01	1.00E+00	4.78E-01	1.00E+00	1.00E+00	0	11	1787	21447	0.00%	8.33%	0.00%	0.05%	shared jan 31	
									4.78E-01	5.51E-01	4	19	3970	21447	21.05%	18.51%	0.10%	0.09%	gluconeogenesis Jan 31	Got2, Mdh2, Slc25a12, Slc25a13
									8.42E-01	8.50E-01	1	9	3970	21447	11.11%	18.51%	0.03%	0.04%	glycolysis Jan 31	Hk2
				MP:0003632	abnormal nervous system morphology	4.78E-01	1.00E+00	4.78E-01	1.00E+00	1.00E+00	0	11	3970	21447	0.00%	18.51%	0.00%	0.05%	shared jan 31	

					1.00E+00	1.00E+00	0	19	1250	21447	0.00%	5.83%	0.00%	0.09%	gluconeogenesis Jan 31	
					1.00E+00	1.00E+00	0	9	1250	21447	0.00%	5.83%	0.00%	0.04%	glycolysis Jan 31	
MP:0002132	abnormal respiratory system morphology	4.84E-01	1.00E+00	4.84E-01	4.84E-01	5.62E-01	1	11	1250	21447	9.09%	5.83%	0.08%	0.05%	shared jan 31	Gpi1
					4.85E-01	5.58E-01	2	19	1812	21447	10.53%	8.45%	0.11%	0.09%	gluconeogenesis Jan 31	Got2, Mdh1
					1.00E+00	1.00E+00	0	9	1812	21447	0.00%	8.45%	0.00%	0.04%	glycolysis Jan 31	
MP:0005382	craniofacial phenotype	4.85E-01	1.00E+00	4.85E-01	1.00E+00	1.00E+00	0	11	1812	21447	0.00%	8.45%	0.00%	0.05%	shared jan 31	
					6.91E-01	7.15E-01	1	19	1284	21447	5.26%	5.99%	0.08%	0.09%	gluconeogenesis Jan 31	Slc25a12
					1.00E+00	1.00E+00	0	9	1284	21447	0.00%	5.99%	0.00%	0.04%	glycolysis Jan 31	
MP:0001399	hyperactivity	4.93E-01	1.00E+00	4.93E-01	4.93E-01	5.67E-01	1	11	1284	21447	9.09%	5.99%	0.08%	0.05%	shared jan 31	Aldoc
					7.69E-01	7.81E-01	1	19	1590	21447	5.26%	7.41%	0.06%	0.09%	gluconeogenesis Jan 31	Slc37a2
					5.00E-01	5.36E-01	1	9	1590	21447	11.11%	7.41%	0.06%	0.04%	glycolysis Jan 31	Pfkm
MP:0000653	abnormal sex gland morphology	5.00E-01	7.69E-01	6.51E-01	5.72E-01	6.17E-01	1	11	1590	21447	9.09%	7.41%	0.06%	0.05%	shared jan 31	Eno4
					5.02E-01	5.74E-01	1	19	773	21447	5.26%	3.60%	0.13%	0.09%	gluconeogenesis Jan 31	G6pc2
					1.00E+00	1.00E+00	0	9	773	21447	0.00%	3.60%	0.00%	0.04%	glycolysis Jan 31	
MP:0002444	abnormal T cell physiology	5.02E-01	1.00E+00	5.02E-01	1.00E+00	1.00E+00	0	11	773	21447	0.00%	3.60%	0.00%	0.05%	shared jan 31	
					1.00E+00	1.00E+00	0	19	1653	21447	0.00%	7.71%	0.00%	0.09%	gluconeogenesis Jan 31	
					5.14E-01	5.47E-01	1	9	1653	21447	11.11%	7.71%	0.06%	0.04%	glycolysis Jan 31	Pfkm
MP:0005406	abnormal heart size	5.14E-01	1.00E+00	5.14E-01	5.86E-01	6.28E-01	1	11	1653	21447	9.09%	7.71%	0.06%	0.05%	shared jan 31	Gpi1
					1.00E+00	1.00E+00	0	19	1679	21447	0.00%	7.83%	0.00%	0.09%	gluconeogenesis Jan 31	
					5.20E-01	5.50E-01	1	9	1679	21447	11.11%	7.83%	0.06%	0.04%	glycolysis Jan 31	Pfkm
MP:0002108	abnormal muscle morphology	5.20E-01	1.00E+00	5.20E-01	1.00E+00	1.00E+00	0	11	1679	21447	0.00%	7.83%	0.00%	0.05%	shared jan 31	
					5.33E-01	6.00E-01	3	19	3103	21447	15.79%	14.47%	0.10%	0.09%	gluconeogenesis Jan 31	Pck1, Slc25a12, Slc25a13
					1.00E+00	1.00E+00	0	9	3103	21447	0.00%	14.47%	0.00%	0.04%	glycolysis Jan 31	
MP:0003491	abnormal voluntary movement	5.33E-01	1.00E+00	5.33E-01	8.21E-01	8.45E-01	1	11	3103	21447	9.09%	14.47%	0.03%	0.05%	shared jan 31	Aldoc
					5.34E-01	6.00E-01	2	19	1978	21447	10.53%	9.22%	0.10%	0.09%	gluconeogenesis Jan 31	Pck2, Slc25a10
					5.81E-01	6.08E-01	1	9	1978	21447	11.11%	9.22%	0.05%	0.04%	glycolysis Jan 31	Gck
MP:0005416	abnormal circulating protein level	5.34E-01	1.00E+00	5.34E-01	1.00E+00	1.00E+00	0	11	1978	21447	0.00%	9.22%	0.00%	0.05%	shared jan 31	
					5.42E-01	6.06E-01	1	19	864	21447	5.26%	4.03%	0.12%	0.09%	gluconeogenesis Jan 31	Slc25a13
					1.00E+00	1.00E+00	0	9	864	21447	0.00%	4.03%	0.00%	0.04%	glycolysis Jan 31	
MP:0000959	abnormal somatic sensory system morphology	5.42E-01	1.00E+00	5.42E-01	1.00E+00	1.00E+00	0	11	864	21447	0.00%	4.03%	0.00%	0.05%	shared jan 31	
					5.44E-01	6.06E-01	1	19	867	21447	5.26%	4.04%	0.12%	0.09%	gluconeogenesis Jan 31	G6pc
					1.00E+00	1.00E+00	0	9	867	21447	0.00%	4.04%	0.00%	0.04%	glycolysis Jan 31	
MP:0010273	increased classified tumor incidence	5.44E-01	1.00E+00	5.44E-01	1.00E+00	1.00E+00	0	11	867	21447	0.00%	4.04%	0.00%	0.05%	shared jan 31	
					8.10E-01	8.21E-01	1	19	1793	21447	5.26%	8.36%	0.06%	0.09%	gluconeogenesis Jan 31	G6pc
					5.44E-01	5.73E-01	1	9	1793	21447	11.11%	8.36%	0.06%	0.04%	glycolysis Jan 31	Pfkm
MP:0002083	premature death	5.44E-01	1.00E+00	5.44E-01	1.00E+00	1.00E+00	0	11	1793	21447	0.00%	8.36%	0.00%	0.05%	shared jan 31	
					5.47E-01	6.06E-01	1	19	874	21447	5.26%	4.08%	0.11%	0.09%	gluconeogenesis Jan 31	G6pc
					1.00E+00	1.00E+00	0	9	874	21447	0.00%	4.08%	0.00%	0.04%	glycolysis Jan 31	
MP:0020188	abnormal classified tumor incidence	5.47E-01	1.00E+00	5.47E-01	1.00E+00	1.00E+00	0	11	874	21447	0.00%	4.08%	0.00%	0.05%	shared jan 31	
					5.48E-01	6.06E-01	1	19	877	21447	5.26%	4.09%	0.11%	0.09%	gluconeogenesis Jan 31	Pck1
					1.00E+00	1.00E+00	0	9	877	21447	0.00%	4.09%	0.00%	0.04%	glycolysis Jan 31	
MP:0002069	abnormal consumption behavior	5.48E-01	1.00E+00	5.48E-01	1.00E+00	1.00E+00	0	11	877	21447	0.00%	4.09%	0.00%	0.05%	shared jan 31	
					5.52E-01	6.08E-01	1	19	886	21447	5.26%	4.13%	0.11%	0.09%	gluconeogenesis Jan 31	G6pc2
					1.00E+00	1.00E+00	0	9	886	21447	0.00%	4.13%	0.00%	0.04%	glycolysis Jan 31	
MP:0000352	decreased cell proliferation	5.52E-01	1.00E+00	5.52E-01	1.00E+00	1.00E+00	0	11	886	21447	0.00%	4.13%	0.00%	0.05%	shared jan 31	
					1.00E+00	1.00E+00	0	19	1840	21447	0.00%	8.58%	0.00%	0.09%	gluconeogenesis Jan 31	
					5.54E-01	5.82E-01	1	9	1840	21447	11.11%	8.58%	0.05%	0.04%	glycolysis Jan 31	Gck
MP:0000358	abnormal cell morphology	5.54E-01	1.00E+00	5.54E-01	6.27E-01	6.65E-01	1	11	1840	21447	9.09%	8.58%	0.05%	0.05%	shared jan 31	Eno4
					5.60E-01	6.15E-01	1	19	906	21447	5.26%	4.22%	0.11%	0.09%	gluconeogenesis Jan 31	Got2
					1.00E+00	1.00E+00	0	9	906	21447	0.00%	4.22%	0.00%	0.04%	glycolysis Jan 31	
MP:0002128	abnormal blood circulation	5.60E-01	1.00E+00	5.60E-01	1.00E+00	1.00E+00	0	11	906	21447	0.00%	4.22%	0.00%	0.05%	shared jan 31	
					8.30E-01	8.39E-01	1	19	1911	21447	5.26%	8.91%	0.05%	0.09%	gluconeogenesis Jan 31	Slc25a13
					5.68E-01	5.96E-01	1	9	1911	21447	11.11%	8.91%	0.05%	0.04%	glycolysis Jan 31	Pfkl
MP:0010678	abnormal skin adnexa morphology	5.68E-01	1.00E+00	5.68E-01	1.00E+00	1.00E+00	0	11	1911	21447	0.00%	8.91%	0.00%	0.05%	shared jan 31	
					7.66E-01	7.79E-01	1	19	1577	21447	5.26%	7.35%	0.06%	0.09%	gluconeogenesis Jan 31	Slc25a12
					1.00E+00	1.00E+00	0	9	1577	21447	0.00%	7.35%	0.00%	0.04%	glycolysis Jan 31	
MP:0002572	abnormal emotion/affect behavior	5.68E-01	1.00E+00	5.68E-01	5.68E-01	6.17E-01	1	11	1577	21447	9.09%	7.35%	0.06%	0.05%	shared jan 31	Aldoa
					5.80E-01	6.29E-01	3	19	3313	21447	15.79%	15.45%	0.09%	0.09%	gluconeogenesis Jan 31	G6pc, Pcx, Slc37a4
					1.00E+00	1.00E+00	0	9	3313	21447	0.00%	15.45%	0.00%	0.04%	glycolysis Jan 31	
MP:0005508	abnormal skeleton morphology	5.80E-01	1.00E+00	5.80E-01	8.42E-01	8.63E-01	1	11	3313	21447	9.09%	15.45%	0.03%	0.05%	shared jan 31	Aldob
					5.85E-01	6.33E-01	1	19	970	21447	5.26%	4.52%	0.10%	0.09%	gluconeogenesis Jan 31	G6pc
					1.00E+00	1.00E+00	0	9	970	21447	0.00%	4.52%	0.00%	0.04%	glycolysis Jan 31	
MP:0010274	increased organ/body region tumor incidence	5.85E-01	1.00E+00	5.85E-01	1.00E+00	1.00E+00	0	11	970	21447	0.00%	4.52%	0.00%	0.05%	shared jan 31	
					5.86E-01	6.33E-01	2	19	2168	21447	10.53%	10.11%	0.09%	0.09%	gluconeogenesis Jan 31	Pck2, Slc25a10
					6.17E-01	6.38E-01	1	9	2168	21447	11.11%	10.11%	0.05%	0.04%	glycolysis Jan 31	Gck
MP:0008469	abnormal protein level	5.86E-01	1.00E+00	5.86E-01	1.00E+00	1.00E+00	0	11	2168	21447	0.00%	10.11%	0.00%	0.05%	shared jan 31	
					5.89E-01	6.35E-01	1	19	981	21447	5.26%	4.57%	0.10%	0.09%	gluconeogenesis Jan 31	G6pc
					1.00E+00	1.00E+00	0	9	981	21447	0.00%	4.57%	0.00%	0.04%	glycolysis Jan 31	
MP:0013152	abnormal organ/body region tumor incidence	5.89E-01	1.00E+00	5.89E-01	1.00E+00	1.00E+00	0	11	981	21447	0.00%	4.57%	0.00%	0.05%	shared jan 31	
					8.50E-01	8.56E-01	1	19	2040	21447	5.26%	9.51%	0.05%	0.09%	gluconeogenesis Jan 31	G6pc

					5.93E-01	6.19E-01	1	9	2040	21447	11.11%	9.51%	0.05%	0.04%	glycolysis Jan 31	Pfkm
MP:0000313	abnormal cell death	5.93E-01	1.00E+00	5.93E-01	1.00E+00	1.00E+00	0	11	2040	21447	0.00%	9.51%	0.00%	0.05%	shared jan 31	
					6.15E-01	6.59E-01	2	19	2279	21447	10.53%	10.63%	0.09%	0.09%	gluconeogenesis Jan 31	Slc25a12, Slc25a13
					1.00E+00	1.00E+00	0	9	2279	21447	0.00%	10.63%	0.00%	0.04%	glycolysis Jan 31	
MP:0001392	abnormal locomotor behavior	6.15E-01	1.00E+00	6.15E-01	1.00E+00	1.00E+00	0	11	2279	21447	0.00%	10.63%	0.00%	0.05%	shared jan 31	
					6.26E-01	6.70E-01	1	19	1082	21447	5.26%	5.04%	0.09%	0.09%	gluconeogenesis Jan 31	Slc25a12
					1.00E+00	1.00E+00	0	9	1082	21447	0.00%	5.04%	0.00%	0.04%	glycolysis Jan 31	
MP:0000787	abnormal telencephalon morphology	6.26E-01	1.00E+00	6.26E-01	1.00E+00	1.00E+00	0	11	1082	21447	0.00%	5.04%	0.00%	0.05%	shared jan 31	
					6.32E-01	6.74E-01	3	19	3557	21447	15.79%	16.59%	0.08%	0.09%	gluconeogenesis Jan 31	G6pc, Pcx, Slc37a4
					1.00E+00	1.00E+00	0	9	3557	21447	0.00%	16.59%	0.00%	0.04%	glycolysis Jan 31	
MP:0005390	skeleton phenotype	6.32E-01	1.00E+00	6.32E-01	8.64E-01	8.81E-01	1	11	3557	21447	9.09%	16.59%	0.03%	0.05%	shared jan 31	Aldob
					1.00E+00	1.00E+00	0	19	2355	21447	0.00%	10.98%	0.00%	0.09%	gluconeogenesis Jan 31	
					6.49E-01	6.65E-01	1	9	2355	21447	11.11%	10.98%	0.04%	0.04%	glycolysis Jan 31	Pfkm
MP:0005369	muscle phenotype	6.49E-01	1.00E+00	6.49E-01	1.00E+00	1.00E+00	0	11	2355	21447	0.00%	10.98%	0.00%	0.05%	shared jan 31	
					6.50E-01	6.89E-01	1	19	1152	21447	5.26%	5.37%	0.09%	0.09%	gluconeogenesis Jan 31	G6pc
					1.00E+00	1.00E+00	0	9	1152	21447	0.00%	5.37%	0.00%	0.04%	glycolysis Jan 31	
MP:0002020	increased tumor incidence	6.50E-01	1.00E+00	6.50E-01	1.00E+00	1.00E+00	0	11	1152	21447	0.00%	5.37%	0.00%	0.05%	shared jan 31	
					6.51E-01	6.90E-01	1	19	1156	21447	5.26%	5.39%	0.09%	0.09%	gluconeogenesis Jan 31	Pck1
					1.00E+00	1.00E+00	0	9	1156	21447	0.00%	5.39%	0.00%	0.04%	glycolysis Jan 31	
MP:0031392	hypoactivity	6.51E-01	1.00E+00	6.51E-01	1.00E+00	1.00E+00	0	11	1156	21447	0.00%	5.39%	0.00%	0.05%	shared jan 31	
					6.54E-01	6.91E-01	2	19	2441	21447	10.53%	11.38%	0.08%	0.09%	gluconeogenesis Jan 31	Pck2, Slc37a2
					6.63E-01	6.78E-01	1	9	2441	21447	11.11%	11.38%	0.04%	0.04%	glycolysis Jan 31	Pfkm
MP:0002160	abnormal reproductive system morphology	6.54E-01	7.35E-01	8.89E-01	7.35E-01	7.67E-01	1	11	2441	21447	9.09%	11.38%	0.04%	0.05%	shared jan 31	Eno4
					6.57E-01	6.92E-01	1	19	1173	21447	5.26%	5.47%	0.09%	0.09%	gluconeogenesis Jan 31	Slc25a12
					1.00E+00	1.00E+00	0	9	1173	21447	0.00%	5.47%	0.00%	0.04%	glycolysis Jan 31	
MP:0003635	abnormal synaptic transmission	6.57E-01	1.00E+00	6.57E-01	1.00E+00	1.00E+00	0	11	1173	21447	0.00%	5.47%	0.00%	0.05%	shared jan 31	
					6.60E-01	6.92E-01	1	19	1182	21447	5.26%	5.51%	0.08%	0.09%	gluconeogenesis Jan 31	Slc25a12
					1.00E+00	1.00E+00	0	9	1182	21447	0.00%	5.51%	0.00%	0.04%	glycolysis Jan 31	
MP:0021009	abnormal synaptic physiology	6.60E-01	1.00E+00	6.60E-01	1.00E+00	1.00E+00	0	11	1182	21447	0.00%	5.51%	0.00%	0.05%	shared jan 31	
					6.69E-01	6.99E-01	2	19	2508	21447	10.53%	11.69%	0.08%	0.09%	gluconeogenesis Jan 31	Got1, Got2
					6.74E-01	6.87E-01	1	9	2508	21447	11.11%	11.69%	0.04%	0.04%	glycolysis Jan 31	Gck
MP:0001544	abnormal cardiovascular system physiology	6.69E-01	1.00E+00	6.69E-01	1.00E+00	1.00E+00	0	11	2508	21447	0.00%	11.69%	0.00%	0.05%	shared jan 31	
					6.74E-01	7.02E-01	1	19	1227	21447	5.26%	5.72%	0.08%	0.09%	gluconeogenesis Jan 31	Slc37a2
					1.00E+00	1.00E+00	0	9	1227	21447	0.00%	5.72%	0.00%	0.04%	glycolysis Jan 31	
MP:0002452	abnormal professional antigen presenting cell physiology	6.74E-01	1.00E+00	6.74E-01	1.00E+00	1.00E+00	0	11	1227	21447	0.00%	5.72%	0.00%	0.05%	shared jan 31	
					6.88E-01	7.15E-01	1	19	1275	21447	5.26%	5.94%	0.08%	0.09%	gluconeogenesis Jan 31	Slc25a13
					1.00E+00	1.00E+00	0	9	1275	21447	0.00%	5.94%	0.00%	0.04%	glycolysis Jan 31	
MP:0000367	abnormal coat/ hair morphology	6.88E-01	1.00E+00	6.88E-01	1.00E+00	1.00E+00	0	11	1275	21447	0.00%	5.94%	0.00%	0.05%	shared jan 31	
					6.89E-01	7.15E-01	1	19	1278	21447	5.26%	5.96%	0.08%	0.09%	gluconeogenesis Jan 31	G6pc
					1.00E+00	1.00E+00	0	9	1278	21447	0.00%	5.96%	0.00%	0.04%	glycolysis Jan 31	
MP:0002019	abnormal tumor incidence	6.89E-01	1.00E+00	6.89E-01	1.00E+00	1.00E+00	0	11	1278	21447	0.00%	5.96%	0.00%	0.05%	shared jan 31	
					6.91E-01	7.15E-01	1	19	1285	21447	5.26%	5.99%	0.08%	0.09%	gluconeogenesis Jan 31	Slc25a13
					1.00E+00	1.00E+00	0	9	1285	21447	0.00%	5.99%	0.00%	0.04%	glycolysis Jan 31	
MP:0002752	abnormal somatic nervous system morphology	6.91E-01	1.00E+00	6.91E-01	1.00E+00	1.00E+00	0	11	1285	21447	0.00%	5.99%	0.00%	0.05%	shared jan 31	
					6.95E-01	7.18E-01	1	19	1300	21447	5.26%	6.06%	0.08%	0.09%	gluconeogenesis Jan 31	G6pc
					1.00E+00	1.00E+00	0	9	1300	21447	0.00%	6.06%	0.00%	0.04%	glycolysis Jan 31	
MP:0002166	abnormal tumor susceptibility	6.95E-01	1.00E+00	6.95E-01	1.00E+00	1.00E+00	0	11	1300	21447	0.00%	6.06%	0.00%	0.05%	shared jan 31	
					7.04E-01	7.25E-01	1	19	1332	21447	5.26%	6.21%	0.08%	0.09%	gluconeogenesis Jan 31	G6pc2
					1.00E+00	1.00E+00	0	9	1332	21447	0.00%	6.21%	0.00%	0.04%	glycolysis Jan 31	
MP:0003945	abnormal lymphocyte physiology	7.04E-01	1.00E+00	7.04E-01	1.00E+00	1.00E+00	0	11	1332	21447	0.00%	6.21%	0.00%	0.05%	shared jan 31	
					7.41E-01	7.57E-01	1	19	1470	21447	5.26%	6.85%	0.07%	0.09%	gluconeogenesis Jan 31	Pck2
					1.00E+00	1.00E+00	0	9	1470	21447	0.00%	6.85%	0.00%	0.04%	glycolysis Jan 31	
MP:0000462	abnormal digestive system morphology	7.41E-01	1.00E+00	7.41E-01	1.00E+00	1.00E+00	0	11	1470	21447	0.00%	6.85%	0.00%	0.05%	shared jan 31	
					7.53E-01	7.69E-01	1	19	1522	21447	5.26%	7.10%	0.07%	0.09%	gluconeogenesis Jan 31	G6pc
					1.00E+00	1.00E+00	0	9	1522	21447	0.00%	7.10%	0.00%	0.04%	glycolysis Jan 31	
MP:0002006	neoplasm	7.53E-01	1.00E+00	7.53E-01	1.00E+00	1.00E+00	0	11	1522	21447	0.00%	7.10%	0.00%	0.05%	shared jan 31	
					8.34E-01	8.42E-01	1	19	1936	21447	5.26%	9.03%	0.05%	0.09%	gluconeogenesis Jan 31	Pck2
					1.00E+00	1.00E+00	0	9	1936	21447	0.00%	9.03%	0.00%	0.04%	glycolysis Jan 31	
MP:0005381	digestive/alimentary phenotype	8.34E-01	1.00E+00	8.34E-01	1.00E+00	1.00E+00	0	11	1936	21447	0.00%	9.03%	0.00%	0.05%	shared jan 31	
					8.64E-01	8.68E-01	1	19	2134	21447	5.26%	9.95%	0.05%	0.09%	gluconeogenesis Jan 31	Slc25a12
					1.00E+00	1.00E+00	0	9	2134	21447	0.00%	9.95%	0.00%	0.04%	glycolysis Jan 31	
MP:0002882	abnormal neuron morphology	8.64E-01	1.00E+00	8.64E-01	1.00E+00	1.00E+00	0	11	2134	21447	0.00%	9.95%	0.00%	0.05%	shared jan 31	
					1.00E+00	1.00E+00	19	19	2144	21447	100.00%	100.00%	0.09%	0.09%	gluconeogenesis Jan 31	Fbp1, Fbp2, G6pc, G6pc2, G6pc3, Got1, Got2, Mdh1, Mdh2, Pck1, Pck2, Pcx, Slc25a10, Slc25a11, Slc25a12, Slc25a13, Slc37a1, Slc37a2, Slc37a4
					1.00E+00	1.00E+00	9	9	2144	21447	100.00%	100.00%	0.04%	0.04%	glycolysis Jan 31	Gck, Hk1, Hk2, Hk3, Hkdc1, Pfkl, Pfkm, Pklr, Pkm
MP:0000001	mammalian phenotype	1.00E+00	1.00E+00	1.00E+00	1.00E+00	1.00E+00	11	11	2144	21447	100.00%	100.00%	0.05%	0.05%	shared jan 31	Aldoa, Aldob, Aldoc, Eno1, Eno4, Gapdh, Gapdhs, Gpi1, Pgam1, Pgk2, Tpi1
