# Supplementary material for: Identification of Biochemical Pathways Responsible for Distinct Phenotypes Using Gene Ontology Causal Activity Models
Source: bioRxiv. 2023 May 25:2023.05.22.541760. Preprint. [Version 2] doi: 10.1101/2023.05.22.541760 (PMC10245817; doi:10.1101/2023.05.22.541760)
Supplement: Supplementary file 2 [file media-2.pdf]

Genes Used			TermID	Term	P	Q	k	n	K	N	k/n	K/N	k/K	n/N	Qset	Symbols
**glycolysis**			MP:0002640	reticulocytosis	7.85E-06	1.94E-03	3	9	99	21447	33.33%	0.46%	3.03%	0.04%	Unique to glycolysis	Hk1, Pfkm, Pklr
Gck (MGI:1270854)			MP:0011913	abnormal reticulocyte cell number	1.17E-05	1.94E-03	3	9	113	21447	33.33%	0.53%	2.65%	0.04%	Unique to glycolysis	Hk1, Pfkm, Pklr
Hk1 (MGI:96103)			MP:0002424	abnormal reticulocyte morphology	1.40E-05	1.94E-03	3	9	120	21447	33.33%	0.56%	2.50%	0.04%	Unique to glycolysis	Hk1, Pfkm, Pklr
Hk2 (MGI:1315197)			MP:0031085	increased erythrocyte osmotic fragility	2.96E-05	3.08E-03	2	9	20	21447	22.22%	0.09%	10.00%	0.04%	Unique to glycolysis	Pfkm, Pklr
Hk3 (MGI:2670962)			MP:0005344	increased circulating bilirubin level	5.53E-05	4.60E-03	3	9	190	21447	33.33%	0.89%	1.58%	0.04%	Unique to glycolysis	Hk1, Pfkm, Pklr
Hkdc1 (MGI:2384910)			MP:0001569	abnormal circulating bilirubin level	6.73E-05	4.67E-03	3	9	203	21447	33.33%	0.95%	1.48%	0.04%	Unique to glycolysis	Hk1, Pfkm, Pklr
Pfkm (MGI:97548)			MP:0003657	abnormal erythrocyte osmotic lysis	8.21E-05	4.88E-03	2	9	33	21447	22.22%	0.15%	6.06%	0.04%	Unique to glycolysis	Pfkm, Pklr
Pfkp (MGI:1891833)			MP:0010163	hemolysis	1.09E-04	5.68E-03	2	9	38	21447	22.22%	0.18%	5.26%	0.04%	Unique to glycolysis	Pfkm, Pklr
Pfkl (MGI:97547)			MP:0001585	hemolytic anemia	1.34E-04	6.18E-03	2	9	42	21447	22.22%	0.20%	4.76%	0.04%	Unique to glycolysis	Hk1, Pklr
Pkm (MGI:97591)			MP:0000208	decreased hematocrit	3.04E-04	1.11E-02	3	9	338	21447	33.33%	1.58%	0.89%	0.04%	Unique to glycolysis	Hk1, Pfkm, Pklr
Pklr (MGI:97604)			MP:0005584	abnormal enzyme/coenzyme activity	3.06E-04	1.11E-02	3	9	339	21447	33.33%	1.58%	0.88%	0.04%	Unique to glycolysis	Gck, Pfkl, Pklr
			MP:0003656	abnormal erythrocyte physiology	3.21E-04	1.11E-02	2	9	65	21447	22.22%	0.30%	3.08%	0.04%	Unique to glycolysis	Pfkm, Pklr
	not annotated to a phenotype		MP:0000874	irregular external granule cell layer thickness	4.20E-04	1.34E-02	1	9	1	21447	11.11%	0.00%	100.00%	0.04%	Unique to glycolysis	Hk2
			MP:0013663	increased myeloid cell number	4.69E-04	1.39E-02	3	9	392	21447	33.33%	1.83%	0.77%	0.04%	Unique to glycolysis	Hk1, Pfkm, Pklr
			MP:0005563	abnormal hemoglobin content	6.57E-04	1.74E-02	3	9	440	21447	33.33%	2.05%	0.68%	0.04%	Unique to glycolysis	Gck, Hk1, Pklr
			MP:0005266	abnormal metabolism	6.69E-04	1.74E-02	4	9	1085	21447	44.44%	5.06%	0.37%	0.04%	Unique to glycolysis	Gck, Pfkl, Pfkm, Pklr
			MP:0002596	abnormal hematocrit	7.34E-04	1.80E-02	3	9	457	21447	33.33%	2.13%	0.66%	0.04%	Unique to glycolysis	Hk1, Pfkm, Pklr
			MP:0011604	abnormal glucokinase activity	8.39E-04	1.84E-02	1	9	2	21447	11.11%	0.01%	50.00%	0.04%	Unique to glycolysis	Gck
			MP:0011606	decreased glucokinase activity	8.39E-04	1.84E-02	1	9	2	21447	11.11%	0.01%	50.00%	0.04%	Unique to glycolysis	Gck
			MP:0005293	impaired glucose tolerance	1.34E-03	2.78E-02	3	9	562	21447	33.33%	2.62%	0.53%	0.04%	Unique to glycolysis	Gck, Hkdc1, Pfkm
			MP:0003059	decreased insulin secretion	1.90E-03	3.76E-02	2	9	159	21447	22.22%	0.74%	1.26%	0.04%	Unique to glycolysis	Gck, Pfkm
			MP:0000245	abnormal erythropoiesis	2.00E-03	3.77E-02	2	9	163	21447	22.22%	0.76%	1.23%	0.04%	Unique to glycolysis	Hk1, Pklr
			MP:0005437	abnormal glycogen level	2.27E-03	4.10E-02	2	9	174	21447	22.22%	0.81%	1.15%	0.04%	Unique to glycolysis	Gck, Pfkm
			MP:0002814	hyperchromasia	2.52E-03	4.36E-02	1	9	6	21447	11.11%	0.03%	16.67%	0.04%	Unique to glycolysis	Hk1
			MP:0005438	abnormal glycogen homeostasis	2.75E-03	4.52E-02	2	9	192	21447	22.22%	0.90%	1.04%	0.04%	Unique to glycolysis	Gck, Pfkm
			MP:0000598	abnormal liver morphology	2.88E-03	4.52E-02	4	9	1602	21447	44.44%	7.47%	0.25%	0.04%	Unique to glycolysis	Gck, Hk1, Hkdc1, Pfkm
			MP:0006353	increased glycosylated hemoglobin level	2.93E-03	4.52E-02	1	9	7	21447	11.11%	0.03%	14.29%	0.04%	Unique to glycolysis	Gck
			MP:0002447	abnormal erythrocyte morphology	3.06E-03	4.55E-02	4	9	1629	21447	44.44%	7.60%	0.25%	0.04%	Unique to glycolysis	Gck, Hk1, Pfkm, Pklr
			MP:0013659	abnormal erythroid lineage cell morphology	3.57E-03	4.65E-02	4	9	1699	21447	44.44%	7.92%	0.24%	0.04%	Unique to glycolysis	Gck, Hk1, Pfkm, Pklr
			MP:0003564	abnormal insulin secretion	3.66E-03	4.65E-02	2	9	222	21447	22.22%	1.04%	0.90%	0.04%	Unique to glycolysis	Gck, Pfkm
			MP:0014195	abnormal endocrine pancreas secretion	3.76E-03	4.65E-02	2	9	225	21447	22.22%	1.05%	0.89%	0.04%	Unique to glycolysis	Gck, Pfkm
			MP:0006351	abnormal glycosylated hemoglobin level	3.77E-03	4.65E-02	1	9	9	21447	11.11%	0.04%	11.11%	0.04%	Unique to glycolysis	Gck
			MP:0002694	abnormal pancreas secretion	4.06E-03	4.65E-02	2	9	234	21447	22.22%	1.09%	0.85%	0.04%	Unique to glycolysis	Gck, Pfkm
			MP:0001770	abnormal iron level	4.13E-03	4.65E-02	2	9	236	21447	22.22%	1.10%	0.85%	0.04%	Unique to glycolysis	Hk1, Pklr
			MP:0002138	abnormal hepatobiliary system morphology	4.16E-03	4.65E-02	4	9	1771	21447	44.44%	8.26%	0.23%	0.04%	Unique to glycolysis	Gck, Hk1, Hkdc1, Pfkm
			MP:0005637	abnormal iron homeostasis	4.33E-03	4.65E-02	2	9	242	21447	22.22%	1.13%	0.83%	0.04%	Unique to glycolysis	Hk1, Pklr
			MP:0031318	enhanced skeletal muscle regeneration	4.61E-03	4.65E-02	1	9	11	21447	11.11%	0.05%	9.09%	0.04%	Unique to glycolysis	Pfkm
			MP:0003562	abnormal pancreatic beta cell physiology	4.76E-03	4.65E-02	2	9	254	21447	22.22%	1.18%	0.79%	0.04%	Unique to glycolysis	Gck, Pfkm
			MP:0013661	abnormal myeloid cell number	4.90E-03	4.65E-02	3	9	886	21447	33.33%	4.13%	0.34%	0.04%	Unique to glycolysis	Hk1, Pfkm, Pklr
			MP:0010147	abnormal endocrine pancreas physiology	4.91E-03	4.65E-02	2	9	258	21447	22.22%	1.20%	0.78%	0.04%	Unique to glycolysis	Gck, Pfkm
			MP:0002590	increased mean corpuscular volume	4.91E-03	4.65E-02	2	9	258	21447	22.22%	1.20%	0.78%	0.04%	Unique to glycolysis	Hk1, Pklr
			MP:0005291	abnormal glucose tolerance	4.91E-03	4.65E-02	3	9	887	21447	33.33%	4.14%	0.34%	0.04%	Unique to glycolysis	Gck, Hkdc1, Pfkm
			MP:0010034	abnormal erythrocyte clearance	5.03E-03	4.65E-02	1	9	12	21447	11.11%	0.06%	8.33%	0.04%	Unique to glycolysis	Pklr
			MP:0013404	decreased circulating lactate level	5.03E-03	4.65E-02	1	9	12	21447	11.11%	0.06%	8.33%	0.04%	Unique to glycolysis	Pfkm
			MP:0010035	increased erythrocyte clearance	5.03E-03	4.65E-02	1	9	12	21447	11.11%	0.06%	8.33%	0.04%	Unique to glycolysis	Pklr
			MP:0005639	hemosiderosis	5.44E-03	4.82E-02	1	9	13	21447	11.11%	0.06%	7.69%	0.04%	Unique to glycolysis	Pklr
			MP:0004952	increased spleen weight	5.48E-03	4.82E-02	2	9	273	21447	22.22%	1.27%	0.73%	0.04%	Unique to glycolysis	Hk1, Pfkm
			MP:0002221	abnormal lymph organ size	5.63E-03	4.82E-02	4	9	1924	21447	44.44%	8.97%	0.21%	0.04%	Unique to glycolysis	Hk1, Hk3, Pfkm, Pklr
			MP:0001588	abnormal hemoglobin	5.68E-03	4.82E-02	3	9	934	21447	33.33%	4.35%	0.32%	0.04%	Unique to glycolysis	Gck, Hk1, Pklr
			MP:0003731	abnormal retina outer nuclear layer morphology	6.00E-03	4.93E-02	2	9	286	21447	22.22%	1.33%	0.70%	0.04%	Unique to glycolysis	Hkdc1, Pkm
			MP:0008859	abnormal hair cycle catagen phase	6.28E-03	4.93E-02	1	9	15	21447	11.11%	0.07%	6.67%	0.04%	Unique to glycolysis	Pfkl
			MP:0013161	enhanced muscle regeneration	6.28E-03	4.93E-02	1	9	15	21447	11.11%	0.07%	6.67%	0.04%	Unique to glycolysis	Pfkm
			MP:0010375	increased kidney iron level	6.28E-03	4.93E-02	1	9	15	21447	11.11%	0.07%	6.67%	0.04%	Unique to glycolysis	Hk1

			MP:0005370	liver/biliary system phenotype	6.49E-03	5.00E-02	4	9	2002	21447	44.44%	9.33%	0.20%	0.04%	Unique to glycolysis	Gck, Hk1, Hkdc1, Pfkm
			MP:0008742	abnormal kidney iron level	6.70E-03	5.06E-02	1	9	16	21447	11.11%	0.07%	6.25%	0.04%	Unique to glycolysis	Hk1
			MP:0000691	enlarged spleen	7.03E-03	5.21E-02	3	9	1008	21447	33.33%	4.70%	0.30%	0.04%	Unique to glycolysis	Hk1, Pfkm, Pklr
			MP:0002874	decreased hemoglobin content	7.14E-03	5.21E-02	2	9	313	21447	22.22%	1.46%	0.64%	0.04%	Unique to glycolysis	Hk1, Pklr
			MP:0002727	decreased circulating insulin level	7.45E-03	5.22E-02	2	9	320	21447	22.22%	1.49%	0.62%	0.04%	Unique to glycolysis	Gck, Pfkm
			MP:0010751	decreased susceptibility to parasitic infection induced morbidity/mortality	7.53E-03	5.22E-02	1	9	18	21447	11.11%	0.08%	5.56%	0.04%	Unique to glycolysis	Pklr
			MP:0011819	increased pancreatic beta cell proliferation	7.53E-03	5.22E-02	1	9	18	21447	11.11%	0.08%	5.56%	0.04%	Unique to glycolysis	Gck
			MP:0009977	abnormal cerebellar granule cell migration	8.36E-03	5.70E-02	1	9	20	21447	11.11%	0.09%	5.00%	0.04%	Unique to glycolysis	Hk2
			MP:0002875	decreased erythrocyte cell number	1.04E-02	6.79E-02	2	9	380	21447	22.22%	1.77%	0.53%	0.04%	Unique to glycolysis	Hk1, Pklr
			MP:0010401	increased skeletal muscle glycogen level	1.04E-02	6.79E-02	1	9	25	21447	11.11%	0.12%	4.00%	0.04%	Unique to glycolysis	Pfkm
			MP:0009414	skeletal muscle fiber necrosis	1.04E-02	6.79E-02	1	9	25	21447	11.11%	0.12%	4.00%	0.04%	Unique to glycolysis	Pfkm
			MP:0001577	anemia	1.09E-02	6.98E-02	2	9	390	21447	22.22%	1.82%	0.51%	0.04%	Unique to glycolysis	Hk1, Pklr
			MP:0004951	abnormal spleen weight	1.12E-02	7.01E-02	2	9	396	21447	22.22%	1.85%	0.51%	0.04%	Unique to glycolysis	Hk1, Pfkm
			MP:0013707	abnormal hematopoietic precursor cell morphology	1.13E-02	7.01E-02	2	9	397	21447	22.22%	1.85%	0.50%	0.04%	Unique to glycolysis	Pfkm, Pklr
			MP:0010768	mortality/aging	1.17E-02	7.09E-02	7	9	7559	21447	77.78%	35.25%	0.09%	0.04%	Unique to glycolysis	Gck, Hk2, Hkdc1, Pfkl, Pfkm, Pklr, Pkm
			MP:0002693	abnormal pancreas physiology	1.20E-02	7.09E-02	2	9	410	21447	22.22%	1.91%	0.49%	0.04%	Unique to glycolysis	Gck, Pfkm
			MP:0002722	abnormal immune system organ morphology	1.21E-02	7.09E-02	4	9	2381	21447	44.44%	11.10%	0.17%	0.04%	Unique to glycolysis	Hk1, Hk3, Pfkm, Pklr
			MP:0014071	increased cardiac muscle glycogen level	1.21E-02	7.09E-02	1	9	29	21447	11.11%	0.14%	3.45%	0.04%	Unique to glycolysis	Pfkm
			MP:0010087	increased circulating fructosamine level	1.25E-02	7.23E-02	1	9	30	21447	11.11%	0.14%	3.33%	0.04%	Unique to glycolysis	Gck
			MP:0013328	visceromegaly	1.28E-02	7.27E-02	3	9	1253	21447	33.33%	5.84%	0.24%	0.04%	Unique to glycolysis	Hk1, Pfkm, Pklr
			MP:0003413	hair follicle degeneration	1.29E-02	7.27E-02	1	9	31	21447	11.11%	0.14%	3.23%	0.04%	Unique to glycolysis	Pfkl
			MP:0009412	skeletal muscle fiber degeneration	1.34E-02	7.28E-02	1	9	32	21447	11.11%	0.15%	3.12%	0.04%	Unique to glycolysis	Pfkm
			MP:0014070	abnormal cardiac muscle glycogen level	1.38E-02	7.28E-02	1	9	33	21447	11.11%	0.15%	3.03%	0.04%	Unique to glycolysis	Pfkm
			MP:0011178	increased erythroblast number	1.38E-02	7.28E-02	1	9	33	21447	11.11%	0.15%	3.03%	0.04%	Unique to glycolysis	Pklr
			MP:0009113	increased pancreatic beta cell mass	1.38E-02	7.28E-02	1	9	33	21447	11.11%	0.15%	3.03%	0.04%	Unique to glycolysis	Gck
			MP:0010770	preweaning lethality	1.41E-02	7.28E-02	6	9	5731	21447	66.67%	26.72%	0.10%	0.04%	Unique to glycolysis	Gck, Hk2, Hkdc1, Pfkl, Pfkm, Pkm
			MP:0011093	embryonic lethality at implantation, complete penetrance	1.42E-02	7.28E-02	1	9	34	21447	11.11%	0.16%	2.94%	0.04%	Unique to glycolysis	Pkm
			MP:0011628	increased mitochondrial number	1.42E-02	7.28E-02	1	9	34	21447	11.11%	0.16%	2.94%	0.04%	Unique to glycolysis	Gck
			MP:0013561	abnormal endocrine gland physiology	1.56E-02	7.90E-02	2	9	470	21447	22.22%	2.19%	0.43%	0.04%	Unique to glycolysis	Gck, Pfkm
			MP:0013403	abnormal circulating lactate level	1.58E-02	7.91E-02	1	9	38	21447	11.11%	0.18%	2.63%	0.04%	Unique to glycolysis	Pfkm
			MP:0002944	increased lactate dehydrogenase level	1.63E-02	7.91E-02	1	9	39	21447	11.11%	0.18%	2.56%	0.04%	Unique to glycolysis	Pfkm
			MP:0005097	polychromatophilia	1.63E-02	7.91E-02	1	9	39	21447	11.11%	0.18%	2.56%	0.04%	Unique to glycolysis	Hk1
			MP:0005039	hypoxia	1.67E-02	7.91E-02	1	9	40	21447	11.11%	0.19%	2.50%	0.04%	Unique to glycolysis	Pfkm
			MP:0000226	abnormal mean corpuscular volume	1.71E-02	7.91E-02	2	9	493	21447	22.22%	2.30%	0.41%	0.04%	Unique to glycolysis	Hk1, Pklr
			MP:0014069	abnormal skeletal muscle glycogen level	1.71E-02	7.91E-02	1	9	41	21447	11.11%	0.19%	2.44%	0.04%	Unique to glycolysis	Pfkm
			MP:0002224	abnormal spleen size	1.73E-02	7.91E-02	3	9	1400	21447	33.33%	6.53%	0.21%	0.04%	Unique to glycolysis	Hk1, Pfkm, Pklr
			MP:0008807	increased liver iron level	1.75E-02	7.91E-02	1	9	42	21447	11.11%	0.20%	2.38%	0.04%	Unique to glycolysis	Hk1
			MP:0001759	increased urine glucose level	1.75E-02	7.91E-02	1	9	42	21447	11.11%	0.20%	2.38%	0.04%	Unique to glycolysis	Gck
			MP:0003852	skeletal muscle necrosis	1.75E-02	7.91E-02	1	9	42	21447	11.11%	0.20%	2.38%	0.04%	Unique to glycolysis	Pfkm
			MP:0011818	abnormal pancreatic beta cell proliferation	1.79E-02	7.92E-02	1	9	43	21447	11.11%	0.20%	2.33%	0.04%	Unique to glycolysis	Gck
			MP:0031116	microvesicular hepatic steatosis	1.79E-02	7.92E-02	1	9	43	21447	11.11%	0.20%	2.33%	0.04%	Unique to glycolysis	Gck
			MP:0001586	abnormal erythrocyte cell number	1.82E-02	7.96E-02	2	9	510	21447	22.22%	2.38%	0.39%	0.04%	Unique to glycolysis	Hk1, Pklr
			MP:0011177	abnormal erythroblast number	1.87E-02	8.03E-02	1	9	45	21447	11.11%	0.21%	2.22%	0.04%	Unique to glycolysis	Pklr
			MP:0008527	embryonic lethality at implantation	1.87E-02	8.03E-02	1	9	45	21447	11.11%	0.21%	2.22%	0.04%	Unique to glycolysis	Pkm
			MP:0001758	abnormal urine glucose level	1.96E-02	8.22E-02	1	9	47	21447	11.11%	0.22%	2.13%	0.04%	Unique to glycolysis	Gck
			MP:0030970	ketosis	1.96E-02	8.22E-02	1	9	47	21447	11.11%	0.22%	2.13%	0.04%	Unique to glycolysis	Gck
			MP:0006069	abnormal retina neuronal layer morphology	2.01E-02	8.37E-02	2	9	538	21447	22.22%	2.51%	0.37%	0.04%	Unique to glycolysis	Hkdc1, Pkm
			MP:0009415	skeletal muscle degeneration	2.04E-02	8.39E-02	1	9	49	21447	11.11%	0.23%	2.04%	0.04%	Unique to glycolysis	Pfkm
			MP:0013237	abnormal skeletal muscle regeneration	2.08E-02	8.48E-02	1	9	50	21447	11.11%	0.23%	2.00%	0.04%	Unique to glycolysis	Pfkm
			MP:0002768	small adrenal glands	2.12E-02	8.56E-02	1	9	51	21447	11.11%	0.24%	1.96%	0.04%	Unique to glycolysis	Hk3

			MP:0008738	abnormal liver iron level	2.20E-02	8.81E-02	1	9	53	21447	11.11%	0.25%	1.89%	0.04%	Unique to glycolysis	Hk1
			MP:0011084	lethality at weaning, incomplete penetrance	2.24E-02	8.88E-02	1	9	54	21447	11.11%	0.25%	1.85%	0.04%	Unique to glycolysis	Pfkm
			MP:0011176	abnormal erythroblast morphology	2.28E-02	8.88E-02	1	9	55	21447	11.11%	0.26%	1.82%	0.04%	Unique to glycolysis	Pklr
			MP:0009172	small pancreatic islets	2.28E-02	8.88E-02	1	9	55	21447	11.11%	0.26%	1.82%	0.04%	Unique to glycolysis	Gck
			MP:0008511	decreased retina inner nuclear layer thickness	2.37E-02	9.12E-02	1	9	57	21447	11.11%	0.27%	1.75%	0.04%	Unique to glycolysis	Hkdc1
			MP:0003135	increased erythroid progenitor cell number	2.45E-02	9.26E-02	1	9	59	21447	11.11%	0.28%	1.69%	0.04%	Unique to glycolysis	Pklr
			MP:0009395	increased nucleated erythrocyte cell number	2.45E-02	9.26E-02	1	9	59	21447	11.11%	0.28%	1.69%	0.04%	Unique to glycolysis	Pklr
			MP:0011996	abnormal retina inner nuclear layer thickness	2.53E-02	9.37E-02	1	9	61	21447	11.11%	0.28%	1.64%	0.04%	Unique to glycolysis	Hkdc1
			MP:0003727	abnormal retina layer morphology	2.57E-02	9.37E-02	2	9	613	21447	22.22%	2.86%	0.33%	0.04%	Unique to glycolysis	Hkdc1, Pkm
			MP:0012397	abnormal nucleated erythrocyte cell number	2.57E-02	9.37E-02	1	9	62	21447	11.11%	0.29%	1.61%	0.04%	Unique to glycolysis	Pklr
			MP:0013662	decreased myeloid cell number	2.61E-02	9.37E-02	2	9	618	21447	22.22%	2.88%	0.32%	0.04%	Unique to glycolysis	Hk1, Pklr
			MP:0003926	decreased cellular glucose uptake	2.61E-02	9.37E-02	1	9	63	21447	11.11%	0.29%	1.59%	0.04%	Unique to glycolysis	Hkdc1
			MP:0009114	decreased pancreatic beta cell mass	2.61E-02	9.37E-02	1	9	63	21447	11.11%	0.29%	1.59%	0.04%	Unique to glycolysis	Gck
			MP:0008518	retina outer nuclear layer degeneration	2.65E-02	9.44E-02	1	9	64	21447	11.11%	0.30%	1.56%	0.04%	Unique to glycolysis	Hkdc1
			MP:0014068	abnormal muscle glycogen level	2.70E-02	9.49E-02	1	9	65	21447	11.11%	0.30%	1.54%	0.04%	Unique to glycolysis	Pfkm
			MP:0013658	abnormal myeloid cell morphology	2.73E-02	9.49E-02	4	9	3016	21447	44.44%	14.06%	0.13%	0.04%	Unique to glycolysis	Gck, Hk1, Pfkm, Pklr
			MP:0001560	abnormal circulating insulin level	2.74E-02	9.49E-02	2	9	634	21447	22.22%	2.96%	0.32%	0.04%	Unique to glycolysis	Gck, Pfkm
			MP:0002943	abnormal lactate dehydrogenase level	2.78E-02	9.55E-02	1	9	67	21447	11.11%	0.31%	1.49%	0.04%	Unique to glycolysis	Pfkm
			MP:0003896	prolonged PR interval	2.86E-02	9.75E-02	1	9	69	21447	11.11%	0.32%	1.45%	0.04%	Unique to glycolysis	Gck
			MP:0005559	increased circulating glucose level	2.90E-02	9.77E-02	2	9	654	21447	22.22%	3.05%	0.31%	0.04%	Unique to glycolysis	Gck, Pfkm
			MP:0010398	decreased liver glycogen level	2.94E-02	9.77E-02	1	9	71	21447	11.11%	0.33%	1.41%	0.04%	Unique to glycolysis	Gck
			MP:0008476	increased spleen red pulp amount	2.94E-02	9.77E-02	1	9	71	21447	11.11%	0.33%	1.41%	0.04%	Unique to glycolysis	Hk1
			MP:0000750	abnormal muscle regeneration	2.98E-02	9.77E-02	1	9	72	21447	11.11%	0.34%	1.39%	0.04%	Unique to glycolysis	Pfkm
			MP:0003058	increased insulin secretion	2.98E-02	9.77E-02	1	9	72	21447	11.11%	0.34%	1.39%	0.04%	Unique to glycolysis	Gck
			MP:0002060	abnormal skin morphology	3.06E-02	9.91E-02	3	9	1735	21447	33.33%	8.09%	0.17%	0.04%	Unique to glycolysis	Hk1, Hk3, Pfkm
			MP:0000689	abnormal spleen morphology	3.07E-02	9.91E-02	3	9	1737	21447	33.33%	8.10%	0.17%	0.04%	Unique to glycolysis	Hk1, Pfkm, Pklr
			MP:0002642	anisocytosis	3.10E-02	9.93E-02	1	9	75	21447	11.11%	0.35%	1.33%	0.04%	Unique to glycolysis	Hk1
			MP:0010769	abnormal survival	3.16E-02	1.00E-01	6	9	6733	21447	66.67%	31.39%	0.09%	0.04%	Unique to glycolysis	Gck, Hk2, Hkdc1, Pfkl, Pfkm, Pkm
			MP:0013657	abnormal blood cell morphology	3.20E-02	1.00E-01	4	9	3161	21447	44.44%	14.74%	0.13%	0.04%	Unique to glycolysis	Gck, Hk1, Pfkm, Pklr
			MP:0010771	integument phenotype	3.21E-02	1.00E-01	4	9	3165	21447	44.44%	14.76%	0.13%	0.04%	Unique to glycolysis	Hk1, Hk3, Pfkl, Pfkm
			MP:0004809	increased hematopoietic stem cell number	3.31E-02	1.02E-01	1	9	80	21447	11.11%	0.37%	1.25%	0.04%	Unique to glycolysis	Pfkm
			MP:0012143	decreased a-wave amplitude	3.35E-02	1.02E-01	1	9	81	21447	11.11%	0.38%	1.23%	0.04%	Unique to glycolysis	Hkdc1
			MP:0005026	decreased susceptibility to parasitic infection	3.35E-02	1.02E-01	1	9	81	21447	11.11%	0.38%	1.23%	0.04%	Unique to glycolysis	Pklr
			MP:0000872	abnormal cerebellum external granule cell layer morphology	3.39E-02	1.03E-01	1	9	82	21447	11.11%	0.38%	1.22%	0.04%	Unique to glycolysis	Hk2
			MP:0012030	abnormal a-wave amplitude	3.43E-02	1.03E-01	1	9	83	21447	11.11%	0.39%	1.20%	0.04%	Unique to glycolysis	Hkdc1
			MP:0011635	abnormal mitochondrial crista morphology	3.43E-02	1.03E-01	1	9	83	21447	11.11%	0.39%	1.20%	0.04%	Unique to glycolysis	Gck
			MP:0012034	abnormal a-wave shape	3.47E-02	1.03E-01	1	9	84	21447	11.11%	0.39%	1.19%	0.04%	Unique to glycolysis	Hkdc1
			MP:0002416	abnormal proerythroblast morphology	3.55E-02	1.05E-01	1	9	86	21447	11.11%	0.40%	1.16%	0.04%	Unique to glycolysis	Pklr
			MP:0011634	abnormal mitochondrial inner membrane morphology	3.63E-02	1.06E-01	1	9	88	21447	11.11%	0.41%	1.14%	0.04%	Unique to glycolysis	Gck
			MP:0009112	abnormal pancreatic beta cell mass	3.67E-02	1.07E-01	1	9	89	21447	11.11%	0.41%	1.12%	0.04%	Unique to glycolysis	Gck
			MP:0005440	increased glycogen level	3.76E-02	1.08E-01	1	9	91	21447	11.11%	0.42%	1.10%	0.04%	Unique to glycolysis	Pfkm
			MP:0005439	decreased glycogen level	3.80E-02	1.09E-01	1	9	92	21447	11.11%	0.43%	1.09%	0.04%	Unique to glycolysis	Gck
			MP:0000749	muscle degeneration	3.84E-02	1.09E-01	1	9	93	21447	11.11%	0.43%	1.08%	0.04%	Unique to glycolysis	Pfkm
			MP:0001265	decreased body size	3.88E-02	1.10E-01	4	9	3351	21447	44.44%	15.62%	0.12%	0.04%	Unique to glycolysis	Gck, Hk1, Hk2, Pfkm
			MP:0010519	atrioventricular block	3.92E-02	1.10E-01	1	9	95	21447	11.11%	0.44%	1.05%	0.04%	Unique to glycolysis	Gck
			MP:0000187	abnormal triglyceride level	3.93E-02	1.10E-01	2	9	771	21447	22.22%	3.59%	0.26%	0.04%	Unique to glycolysis	Gck, Hkdc1
			MP:0011097	embryonic lethality between somite formation and embryo turning, complete penetrance	3.96E-02	1.10E-01	1	9	96	21447	11.11%	0.45%	1.04%	0.04%	Unique to glycolysis	Hk2
			MP:0012144	decreased b-wave amplitude	4.04E-02	1.11E-01	1	9	98	21447	11.11%	0.46%	1.02%	0.04%	Unique to glycolysis	Hkdc1
			MP:0012106	impaired exercise endurance	4.08E-02	1.11E-01	1	9	99	21447	11.11%	0.46%	1.01%	0.04%	Unique to glycolysis	Pfkm
			MP:0000427	abnormal hair cycle	4.12E-02	1.11E-01	1	9	100	21447	11.11%	0.47%	1.00%	0.04%	Unique to glycolysis	Pfkl

			MP:0010392	prolonged QRS complex duration	4.12E-02	1.11E-01	1	9	100	21447	11.11%	0.47%	1.00%	0.04%	Unique to glycolysis	Gck
			MP:0012031	abnormal b-wave amplitude	4.16E-02	1.11E-01	1	9	101	21447	11.11%	0.47%	0.99%	0.04%	Unique to glycolysis	Hkdc1
			MP:0012035	abnormal b-wave shape	4.16E-02	1.11E-01	1	9	101	21447	11.11%	0.47%	0.99%	0.04%	Unique to glycolysis	Hkdc1
			MP:0010636	bundle branch block	4.20E-02	1.11E-01	1	9	102	21447	11.11%	0.48%	0.98%	0.04%	Unique to glycolysis	Gck
			MP:0010560	intraventricular block	4.20E-02	1.11E-01	1	9	102	21447	11.11%	0.48%	0.98%	0.04%	Unique to glycolysis	Gck
			MP:0011100	preweaning lethality, complete penetrance	4.27E-02	1.11E-01	3	9	1972	21447	33.33%	9.19%	0.15%	0.04%	Unique to glycolysis	Hkdc1, Pfkl, Pkm
			MP:0003901	abnormal PR interval	4.28E-02	1.11E-01	1	9	104	21447	11.11%	0.48%	0.96%	0.04%	Unique to glycolysis	Gck
			MP:0011181	increased hematopoietic cell number	4.31E-02	1.11E-01	3	9	1978	21447	33.33%	9.22%	0.15%	0.04%	Unique to glycolysis	Hk1, Pfkm, Pklr
			MP:0009786	decreased susceptibility to infection induced morbidity/mortality	4.36E-02	1.12E-01	1	9	106	21447	11.11%	0.49%	0.94%	0.04%	Unique to glycolysis	Pklr
			MP:0008569	lethality at weaning	4.40E-02	1.12E-01	1	9	107	21447	11.11%	0.50%	0.93%	0.04%	Unique to glycolysis	Pfkm
			MP:0009788	increased susceptibility to bacterial infection induced morbidity/mortality	4.44E-02	1.13E-01	1	9	108	21447	11.11%	0.50%	0.93%	0.04%	Unique to glycolysis	Pklr
			MP:0004948	abnormal neuronal precursor proliferation	4.52E-02	1.13E-01	1	9	110	21447	11.11%	0.51%	0.91%	0.04%	Unique to glycolysis	Hk2
			MP:0002899	fatigue	4.52E-02	1.13E-01	1	9	110	21447	11.11%	0.51%	0.91%	0.04%	Unique to glycolysis	Pfkm
			MP:0012777	abnormal liver glycogen level	4.56E-02	1.14E-01	1	9	111	21447	11.11%	0.52%	0.90%	0.04%	Unique to glycolysis	Gck
			MP:0010086	abnormal circulating fructosamine level	4.68E-02	1.16E-01	1	9	114	21447	11.11%	0.53%	0.88%	0.04%	Unique to glycolysis	Gck
			MP:0009356	decreased liver triglyceride level	4.72E-02	1.16E-01	1	9	115	21447	11.11%	0.54%	0.87%	0.04%	Unique to glycolysis	Hkdc1
			MP:0006206	embryonic lethality between somite formation and embryo turning	4.76E-02	1.16E-01	1	9	116	21447	11.11%	0.54%	0.86%	0.04%	Unique to glycolysis	Hk2
			MP:0001157	small seminal vesicle	4.80E-02	1.16E-01	1	9	117	21447	11.11%	0.55%	0.85%	0.04%	Unique to glycolysis	Pfkm
			MP:0002078	abnormal glucose homeostasis	4.81E-02	1.16E-01	3	9	2065	21447	33.33%	9.63%	0.15%	0.04%	Unique to glycolysis	Gck, Hkdc1, Pfkm
			MP:0009404	centrally nucleated skeletal muscle fibers	4.89E-02	1.17E-01	1	9	119	21447	11.11%	0.55%	0.84%	0.04%	Unique to glycolysis	Pfkm
			MP:0011925	abnormal heart echocardiography feature	5.01E-02	1.20E-01	1	9	122	21447	11.11%	0.57%	0.82%	0.04%	Unique to glycolysis	Gck
			MP:0012029	abnormal electroretinogram waveform feature	5.09E-02	1.21E-01	1	9	124	21447	11.11%	0.58%	0.81%	0.04%	Unique to glycolysis	Hkdc1
			MP:0009170	abnormal pancreatic islet size	5.21E-02	1.23E-01	1	9	127	21447	11.11%	0.59%	0.79%	0.04%	Unique to glycolysis	Gck
			MP:0002332	abnormal exercise endurance	5.25E-02	1.23E-01	1	9	128	21447	11.11%	0.60%	0.78%	0.04%	Unique to glycolysis	Pfkm
			MP:0003925	abnormal cellular glucose uptake	5.33E-02	1.24E-01	1	9	130	21447	11.11%	0.61%	0.77%	0.04%	Unique to glycolysis	Hkdc1
			MP:0006141	abnormal atrioventricular node conduction	5.41E-02	1.26E-01	1	9	132	21447	11.11%	0.62%	0.76%	0.04%	Unique to glycolysis	Gck
			MP:0002164	abnormal gland physiology	5.54E-02	1.27E-01	2	9	932	21447	22.22%	4.35%	0.21%	0.04%	Unique to glycolysis	Gck, Pfkm
			MP:0006009	abnormal neuronal migration	5.57E-02	1.27E-01	1	9	136	21447	11.11%	0.63%	0.74%	0.04%	Unique to glycolysis	Hk2
			MP:0006410	abnormal common myeloid progenitor cell morphology	5.61E-02	1.27E-01	1	9	137	21447	11.11%	0.64%	0.73%	0.04%	Unique to glycolysis	Pklr
			MP:0008801	abnormal erythroid progenitor cell morphology	5.65E-02	1.27E-01	1	9	138	21447	11.11%	0.64%	0.72%	0.04%	Unique to glycolysis	Pklr
			MP:0003898	abnormal QRS complex	5.69E-02	1.27E-01	1	9	139	21447	11.11%	0.65%	0.72%	0.04%	Unique to glycolysis	Gck
			MP:0000189	hypoglycemia	5.69E-02	1.27E-01	1	9	139	21447	11.11%	0.65%	0.72%	0.04%	Unique to glycolysis	Gck
			MP:0003733	abnormal retina inner nuclear layer morphology	5.72E-02	1.27E-01	1	9	140	21447	11.11%	0.65%	0.71%	0.04%	Unique to glycolysis	Hkdc1
			MP:0001554	increased circulating free fatty acids level	5.72E-02	1.27E-01	1	9	140	21447	11.11%	0.65%	0.71%	0.04%	Unique to glycolysis	Gck
			MP:0002625	heart left ventricle hypertrophy	5.84E-02	1.29E-01	1	9	143	21447	11.11%	0.67%	0.70%	0.04%	Unique to glycolysis	Pfkm
			MP:0009967	abnormal neuron proliferation	5.96E-02	1.31E-01	1	9	146	21447	11.11%	0.68%	0.68%	0.04%	Unique to glycolysis	Hk2
			MP:0001148	enlarged testis	5.96E-02	1.31E-01	1	9	146	21447	11.11%	0.68%	0.68%	0.04%	Unique to glycolysis	Pfkm
			MP:0008762	embryonic lethality	6.17E-02	1.34E-01	3	9	2280	21447	33.33%	10.63%	0.13%	0.04%	Unique to glycolysis	Gck, Hk2, Pkm
			MP:0000854	abnormal cerebellum development	6.24E-02	1.35E-01	1	9	153	21447	11.11%	0.71%	0.65%	0.04%	Unique to glycolysis	Hk2
			MP:0002356	abnormal spleen red pulp morphology	6.24E-02	1.35E-01	1	9	153	21447	11.11%	0.71%	0.65%	0.04%	Unique to glycolysis	Hk1
			MP:0000192	abnormal mineral level	6.55E-02	1.40E-01	2	9	1023	21447	22.22%	4.77%	0.20%	0.04%	Unique to glycolysis	Hk1, Pklr
			MP:0011110	preweaning lethality, incomplete penetrance	6.57E-02	1.40E-01	2	9	1025	21447	22.22%	4.78%	0.20%	0.04%	Unique to glycolysis	Gck, Pfkm
			MP:0005636	abnormal mineral homeostasis	6.66E-02	1.41E-01	2	9	1033	21447	22.22%	4.82%	0.19%	0.04%	Unique to glycolysis	Hk1, Pklr
			MP:0008515	thin retina outer nuclear layer	6.72E-02	1.42E-01	1	9	165	21447	11.11%	0.77%	0.61%	0.04%	Unique to glycolysis	Hkdc1
			MP:0011997	abnormal retina outer nuclear layer thickness	6.76E-02	1.42E-01	1	9	166	21447	11.11%	0.77%	0.60%	0.04%	Unique to glycolysis	Hkdc1
			MP:0004808	abnormal hematopoietic stem cell morphology	6.87E-02	1.44E-01	1	9	169	21447	11.11%	0.79%	0.59%	0.04%	Unique to glycolysis	Pfkm
			MP:0010559	heart block	6.91E-02	1.44E-01	1	9	170	21447	11.11%	0.79%	0.59%	0.04%	Unique to glycolysis	Gck
			MP:0004907	abnormal seminal vesicle size	7.03E-02	1.46E-01	1	9	173	21447	11.11%	0.81%	0.58%	0.04%	Unique to glycolysis	Pfkm

			MP:0003956	abnormal body size	7.29E-02	1.50E-01	4	9	4073	21447	44.44%	18.99%	0.10%	0.04%	Unique to glycolysis	Gck, Hk1, Hk2, Pfkm
			MP:0020184	abnormal susceptibility to parasitic infection	7.38E-02	1.51E-01	1	9	182	21447	11.11%	0.85%	0.55%	0.04%	Unique to glycolysis	Pklr
			MP:0000685	abnormal immune system morphology	7.51E-02	1.53E-01	4	9	4113	21447	44.44%	19.18%	0.10%	0.04%	Unique to glycolysis	Hk1, Hk3, Pfkm, Pklr
			MP:0000639	abnormal adrenal gland morphology	7.54E-02	1.53E-01	1	9	186	21447	11.11%	0.87%	0.54%	0.04%	Unique to glycolysis	Hk3
			MP:0002169	no abnormal phenotype detected	7.63E-02	1.54E-01	3	9	2484	21447	33.33%	11.58%	0.12%	0.04%	Unique to glycolysis	Hk2, Pklr, Pkm
			MP:0002873	normal phenotype	7.65E-02	1.54E-01	3	9	2487	21447	33.33%	11.60%	0.12%	0.04%	Unique to glycolysis	Hk2, Pklr, Pkm
			MP:0005281	increased fatty acids level	7.85E-02	1.56E-01	1	9	194	21447	11.11%	0.90%	0.52%	0.04%	Unique to glycolysis	Gck
			MP:0005217	abnormal pancreatic beta cell morphology	7.89E-02	1.56E-01	1	9	195	21447	11.11%	0.91%	0.51%	0.04%	Unique to glycolysis	Gck
			MP:0001559	hyperglycemia	7.89E-02	1.56E-01	1	9	195	21447	11.11%	0.91%	0.51%	0.04%	Unique to glycolysis	Gck
			MP:0009787	increased susceptibility to infection induced morbidity/mortality	7.93E-02	1.56E-01	1	9	196	21447	11.11%	0.91%	0.51%	0.04%	Unique to glycolysis	Pklr
			MP:0013278	decreased fasting circulating glucose level	7.97E-02	1.56E-01	1	9	197	21447	11.11%	0.92%	0.51%	0.04%	Unique to glycolysis	Gck
			MP:0013292	embryonic lethality prior to organogenesis	8.00E-02	1.56E-01	2	9	1146	21447	22.22%	5.34%	0.17%	0.04%	Unique to glycolysis	Hk2, Pkm
			MP:0005418	abnormal circulating hormone level	8.24E-02	1.60E-01	2	9	1166	21447	22.22%	5.44%	0.17%	0.04%	Unique to glycolysis	Gck, Pfkm
			MP:0005376	homeostasis/metabolism phenotype	8.27E-02	1.60E-01	6	9	8239	21447	66.67%	38.42%	0.07%	0.04%	Unique to glycolysis	Gck, Hk1, Hkdc1, Pfkl, Pfkm, Pklr
			MP:0002123	abnormal definitive hematopoiesis	8.33E-02	1.60E-01	2	9	1173	21447	22.22%	5.47%	0.17%	0.04%	Unique to glycolysis	Hk1, Pklr
			MP:0001325	abnormal retina morphology	8.46E-02	1.62E-01	2	9	1183	21447	22.22%	5.52%	0.17%	0.04%	Unique to glycolysis	Hkdc1, Pkm
			MP:0013656	abnormal hematopoietic cell morphology	8.48E-02	1.62E-01	4	9	4277	21447	44.44%	19.94%	0.09%	0.04%	Unique to glycolysis	Gck, Hk1, Pfkm, Pklr
			MP:0031094	organomegaly	8.55E-02	1.62E-01	3	9	2603	21447	33.33%	12.14%	0.12%	0.04%	Unique to glycolysis	Hk1, Pfkm, Pklr
			MP:0001765	abnormal ion homeostasis	8.62E-02	1.63E-01	2	9	1196	21447	22.22%	5.58%	0.17%	0.04%	Unique to glycolysis	Hk1, Pklr
			MP:0013693	abnormal hemopoiesis	8.67E-02	1.63E-01	2	9	1200	21447	22.22%	5.60%	0.17%	0.04%	Unique to glycolysis	Hk1, Pklr
			MP:0006108	abnormal hindbrain development	8.71E-02	1.63E-01	1	9	216	21447	11.11%	1.01%	0.46%	0.04%	Unique to glycolysis	Hk2
			MP:0002864	abnormal ocular fundus morphology	8.83E-02	1.65E-01	2	9	1213	21447	22.22%	5.66%	0.16%	0.04%	Unique to glycolysis	Hkdc1, Pkm
			MP:0010579	increased heart left ventricle size	9.40E-02	1.75E-01	1	9	234	21447	11.11%	1.09%	0.43%	0.04%	Unique to glycolysis	Pfkm
			MP:0005562	decreased mean corpuscular hemoglobin	9.67E-02	1.79E-01	1	9	241	21447	11.11%	1.12%	0.41%	0.04%	Unique to glycolysis	Hk1
			MP:0012778	abnormal liver triglyceride level	9.86E-02	1.82E-01	1	9	246	21447	11.11%	1.15%	0.41%	0.04%	Unique to glycolysis	Hkdc1
			MP:0003953	abnormal hormone level	9.99E-02	1.82E-01	2	9	1303	21447	22.22%	6.08%	0.15%	0.04%	Unique to glycolysis	Gck, Pfkm
			MP:0003717	pallor	1.01E-01	1.82E-01	1	9	251	21447	11.11%	1.17%	0.40%	0.04%	Unique to glycolysis	Hk1
			MP:0013293	embryonic lethality prior to tooth bud stage	1.01E-01	1.82E-01	2	9	1308	21447	22.22%	6.10%	0.15%	0.04%	Unique to glycolysis	Hk2, Pkm
			MP:0000188	abnormal circulating glucose level	1.01E-01	1.82E-01	2	9	1309	21447	22.22%	6.10%	0.15%	0.04%	Unique to glycolysis	Gck, Pfkm
			MP:0013279	increased fasting circulating glucose level	1.03E-01	1.84E-01	1	9	257	21447	11.11%	1.20%	0.39%	0.04%	Unique to glycolysis	Gck
			MP:0005331	insulin resistance	1.03E-01	1.84E-01	1	9	257	21447	11.11%	1.20%	0.39%	0.04%	Unique to glycolysis	Gck
			MP:0001553	abnormal circulating free fatty acids level	1.05E-01	1.87E-01	1	9	262	21447	11.11%	1.22%	0.38%	0.04%	Unique to glycolysis	Gck
			MP:0013560	abnormal endocrine gland morphology	1.07E-01	1.90E-01	2	9	1355	21447	22.22%	6.32%	0.15%	0.04%	Unique to glycolysis	Gck, Hk3
			MP:0002429	abnormal blood cell morphology/development	1.09E-01	1.91E-01	2	9	1369	21447	22.22%	6.38%	0.15%	0.04%	Unique to glycolysis	Hk1, Pklr
			MP:0010680	abnormal skin adnexa physiology	1.09E-01	1.91E-01	1	9	273	21447	11.11%	1.27%	0.37%	0.04%	Unique to glycolysis	Pfkl
			MP:0000377	abnormal hair follicle morphology	1.09E-01	1.91E-01	1	9	274	21447	11.11%	1.28%	0.36%	0.04%	Unique to glycolysis	Pfkl
			MP:0006035	abnormal mitochondrial morphology	1.09E-01	1.91E-01	1	9	274	21447	11.11%	1.28%	0.36%	0.04%	Unique to glycolysis	Gck
			MP:0009785	abnormal susceptibility to infection induced morbidity/mortality	1.10E-01	1.91E-01	1	9	276	21447	11.11%	1.29%	0.36%	0.04%	Unique to glycolysis	Pklr
			MP:0010067	increased red blood cell distribution width	1.10E-01	1.91E-01	1	9	276	21447	11.11%	1.29%	0.36%	0.04%	Unique to glycolysis	Hk1
			MP:0003728	abnormal retina photoreceptor layer morphology	1.11E-01	1.91E-01	1	9	278	21447	11.11%	1.30%	0.36%	0.04%	Unique to glycolysis	Hkdc1
			MP:0010578	abnormal heart left ventricle size	1.12E-01	1.91E-01	1	9	281	21447	11.11%	1.31%	0.36%	0.04%	Unique to glycolysis	Pfkm
			MP:0002092	abnormal eye morphology	1.12E-01	1.91E-01	3	9	2916	21447	33.33%	13.60%	0.10%	0.04%	Unique to glycolysis	Hk3, Hkdc1, Pkm
			MP:0005319	abnormal enzyme/coenzyme level	1.12E-01	1.91E-01	2	9	1395	21447	22.22%	6.50%	0.14%	0.04%	Unique to glycolysis	Gck, Pfkm
			MP:0010358	abnormal free fatty acids level	1.13E-01	1.91E-01	1	9	283	21447	11.11%	1.32%	0.35%	0.04%	Unique to glycolysis	Gck
			MP:0011108	embryonic lethality during organogenesis, incomplete penetrance	1.13E-01	1.91E-01	1	9	284	21447	11.11%	1.32%	0.35%	0.04%	Unique to glycolysis	Gck
			MP:0002089	abnormal postnatal growth/weight/body size	1.13E-01	1.91E-01	4	9	4703	21447	44.44%	21.93%	0.09%	0.04%	Unique to glycolysis	Gck, Hk1, Hk2, Pfkm
			MP:0002059	abnormal seminal vesicle morphology	1.16E-01	1.95E-01	1	9	293	21447	11.11%	1.37%	0.34%	0.04%	Unique to glycolysis	Pfkm

			MP:0001574	abnormal oxygen level	1.18E-01	1.97E-01	1	9	298	21447	11.11%	1.39%	0.34%	0.04%	Unique to glycolysis	Pfkm
			MP:0003084	abnormal skeletal muscle fiber morphology	1.19E-01	1.97E-01	1	9	300	21447	11.11%	1.40%	0.33%	0.04%	Unique to glycolysis	Pfkm
			MP:0005195	abnormal posterior eye segment morphology	1.19E-01	1.97E-01	2	9	1447	21447	22.22%	6.75%	0.14%	0.04%	Unique to glycolysis	Hkdc1, Pkm
			MP:0002628	hepatic steatosis	1.19E-01	1.97E-01	1	9	301	21447	11.11%	1.40%	0.33%	0.04%	Unique to glycolysis	Gck
			MP:0011180	abnormal hematopoietic cell number	1.21E-01	1.98E-01	3	9	3011	21447	33.33%	14.04%	0.10%	0.04%	Unique to glycolysis	Hk1, Pfkm, Pklr
			MP:0000316	cellular necrosis	1.21E-01	1.98E-01	1	9	305	21447	11.11%	1.42%	0.33%	0.04%	Unique to glycolysis	Pfkm
			MP:0002080	prenatal lethality	1.22E-01	1.99E-01	3	9	3020	21447	33.33%	14.08%	0.10%	0.04%	Unique to glycolysis	Gck, Hk2, Pkm
			MP:0010066	abnormal red blood cell distribution width	1.23E-01	2.00E-01	1	9	311	21447	11.11%	1.45%	0.32%	0.04%	Unique to glycolysis	Hk1
			MP:0005621	abnormal cell physiology	1.23E-01	2.00E-01	4	9	4840	21447	44.44%	22.57%	0.08%	0.04%	Unique to glycolysis	Gck, Hk2, Hkdc1, Pfkm
			MP:0002412	increased susceptibility to bacterial infection	1.25E-01	2.01E-01	1	9	316	21447	11.11%	1.47%	0.32%	0.04%	Unique to glycolysis	Pklr
			MP:0005215	abnormal pancreatic islet morphology	1.25E-01	2.01E-01	1	9	317	21447	11.11%	1.48%	0.32%	0.04%	Unique to glycolysis	Gck
			MP:0009165	abnormal endocrine pancreas morphology	1.26E-01	2.01E-01	1	9	318	21447	11.11%	1.48%	0.31%	0.04%	Unique to glycolysis	Gck
			MP:0003141	cardiac fibrosis	1.26E-01	2.01E-01	1	9	319	21447	11.11%	1.49%	0.31%	0.04%	Unique to glycolysis	Gck
			MP:0002409	decreased susceptibility to infection	1.28E-01	2.03E-01	1	9	323	21447	11.11%	1.51%	0.31%	0.04%	Unique to glycolysis	Pklr
			MP:0003960	increased lean body mass	1.31E-01	2.07E-01	1	9	331	21447	11.11%	1.54%	0.30%	0.04%	Unique to glycolysis	Pfkm
			MP:0001552	increased circulating triglyceride level	1.33E-01	2.09E-01	1	9	336	21447	11.11%	1.57%	0.30%	0.04%	Unique to glycolysis	Gck
			MP:0000601	small liver	1.35E-01	2.13E-01	1	9	344	21447	11.11%	1.60%	0.29%	0.04%	Unique to glycolysis	Pfkm
			MP:0005280	abnormal fatty acids level	1.38E-01	2.14E-01	1	9	350	21447	11.11%	1.63%	0.29%	0.04%	Unique to glycolysis	Gck
			MP:0000350	abnormal cell proliferation	1.38E-01	2.14E-01	2	9	1577	21447	22.22%	7.35%	0.13%	0.04%	Unique to glycolysis	Gck, Hk2
			MP:0002079	increased circulating insulin level	1.38E-01	2.14E-01	1	9	351	21447	11.11%	1.64%	0.28%	0.04%	Unique to glycolysis	Gck
			MP:0030572	abnormal pilosebaceous unit morphology	1.40E-01	2.16E-01	1	9	356	21447	11.11%	1.66%	0.28%	0.04%	Unique to glycolysis	Pfkl
			MP:0001764	abnormal homeostasis	1.40E-01	2.16E-01	5	9	7085	21447	55.56%	33.03%	0.07%	0.04%	Unique to glycolysis	Gck, Hk1, Hkdc1, Pfkm, Pklr
			MP:0011088	neonatal lethality, incomplete penetrance	1.41E-01	2.16E-01	1	9	359	21447	11.11%	1.67%	0.28%	0.04%	Unique to glycolysis	Gck
			MP:0013330	abnormal male accessory sex gland morphology	1.42E-01	2.16E-01	1	9	362	21447	11.11%	1.69%	0.28%	0.04%	Unique to glycolysis	Pfkm
			MP:0008772	increased heart ventricle size	1.42E-01	2.16E-01	1	9	362	21447	11.11%	1.69%	0.28%	0.04%	Unique to glycolysis	Pfkm
			MP:0005391	vision/eye phenotype	1.43E-01	2.16E-01	3	9	3234	21447	33.33%	15.08%	0.09%	0.04%	Unique to glycolysis	Hk3, Hkdc1, Pkm
			MP:0002396	abnormal hematopoietic system morphology/development	1.45E-01	2.20E-01	4	9	5119	21447	44.44%	23.87%	0.08%	0.04%	Unique to glycolysis	Gck, Hk1, Pfkm, Pklr
			MP:0005318	decreased triglyceride level	1.46E-01	2.20E-01	1	9	373	21447	11.11%	1.74%	0.27%	0.04%	Unique to glycolysis	Hkdc1
			MP:0010508	abnormal heart electrocardiography waveform feature	1.52E-01	2.28E-01	1	9	390	21447	11.11%	1.82%	0.26%	0.04%	Unique to glycolysis	Gck
			MP:0002163	abnormal gland morphology	1.53E-01	2.28E-01	3	9	3333	21447	33.33%	15.54%	0.09%	0.04%	Unique to glycolysis	Gck, Hk3, Pfkm
			MP:0005451	abnormal body composition	1.57E-01	2.34E-01	3	9	3373	21447	33.33%	15.73%	0.09%	0.04%	Unique to glycolysis	Gck, Hk2, Pfkm
			MP:0020186	abnormal susceptibility to bacterial infection	1.61E-01	2.39E-01	1	9	413	21447	11.11%	1.93%	0.24%	0.04%	Unique to glycolysis	Pklr
			MP:0000260	abnormal angiogenesis	1.63E-01	2.41E-01	1	9	419	21447	11.11%	1.95%	0.24%	0.04%	Unique to glycolysis	Pfkm
			MP:0003137	abnormal impulse conducting system conduction	1.64E-01	2.42E-01	1	9	423	21447	11.11%	1.97%	0.24%	0.04%	Unique to glycolysis	Gck
			MP:0009642	abnormal blood homeostasis	1.65E-01	2.42E-01	4	9	5352	21447	44.44%	24.95%	0.07%	0.04%	Unique to glycolysis	Gck, Hk1, Pfkm, Pklr
			MP:0001625	cardiac hypertrophy	1.66E-01	2.43E-01	1	9	427	21447	11.11%	1.99%	0.23%	0.04%	Unique to glycolysis	Pfkm
			MP:0011091	prenatal lethality, complete penetrance	1.72E-01	2.51E-01	1	9	444	21447	11.11%	2.07%	0.23%	0.04%	Unique to glycolysis	Pkm
			MP:0013277	abnormal fasting circulating glucose level	1.74E-01	2.53E-01	1	9	450	21447	11.11%	2.10%	0.22%	0.04%	Unique to glycolysis	Gck
			MP:0001926	female infertility	1.76E-01	2.54E-01	1	9	455	21447	11.11%	2.12%	0.22%	0.04%	Unique to glycolysis	Hk1
			MP:0001547	abnormal lipid level	1.76E-01	2.55E-01	2	9	1834	21447	22.22%	8.55%	0.11%	0.04%	Unique to glycolysis	Gck, Hkdc1
			MP:0005317	increased triglyceride level	1.78E-01	2.56E-01	1	9	462	21447	11.11%	2.15%	0.22%	0.04%	Unique to glycolysis	Gck
			MP:0005384	cellular phenotype	1.81E-01	2.60E-01	4	9	5535	21447	44.44%	25.81%	0.07%	0.04%	Unique to glycolysis	Gck, Hk2, Hkdc1, Pfkm
			MP:0001944	abnormal pancreas morphology	1.85E-01	2.64E-01	1	9	481	21447	11.11%	2.24%	0.21%	0.04%	Unique to glycolysis	Gck
			MP:0001589	abnormal mean corpuscular hemoglobin	1.86E-01	2.65E-01	1	9	484	21447	11.11%	2.26%	0.21%	0.04%	Unique to glycolysis	Hk1
			MP:0010025	decreased total body fat amount	1.87E-01	2.65E-01	1	9	487	21447	11.11%	2.27%	0.21%	0.04%	Unique to glycolysis	Pfkm
			MP:0002968	increased circulating alkaline phosphatase level	1.89E-01	2.66E-01	1	9	492	21447	11.11%	2.29%	0.20%	0.04%	Unique to glycolysis	Gck
			MP:0005379	endocrine/exocrine gland phenotype	1.89E-01	2.66E-01	3	9	3671	21447	33.33%	17.12%	0.08%	0.04%	Unique to glycolysis	Gck, Hk3, Pfkm
			MP:0001258	decreased body length	1.92E-01	2.70E-01	1	9	502	21447	11.11%	2.34%	0.20%	0.04%	Unique to glycolysis	Pfkm
			MP:0000702	enlarged lymph nodes	1.93E-01	2.70E-01	1	9	506	21447	11.11%	2.36%	0.20%	0.04%	Unique to glycolysis	Hk3

			MP:0012382	abnormal blood cell physiology	1.94E-01	2.70E-01	2	9	1946	21447	22.22%	9.07%	0.10%	0.04%	Unique to glycolysis	Pfkm, Pklr
			MP:0009850	embryonic lethality between implantation and placentation	1.96E-01	2.72E-01	1	9	513	21447	11.11%	2.39%	0.19%	0.04%	Unique to glycolysis	Hk2
			MP:0002118	abnormal lipid homeostasis	1.96E-01	2.72E-01	2	9	1962	21447	22.22%	9.15%	0.10%	0.04%	Unique to glycolysis	Gck, Hkdc1
			MP:0001304	cataract	1.97E-01	2.73E-01	1	9	517	21447	11.11%	2.41%	0.19%	0.04%	Unique to glycolysis	Hk3
			MP:0005397	hematopoietic system phenotype	2.00E-01	2.76E-01	4	9	5741	21447	44.44%	26.77%	0.07%	0.04%	Unique to glycolysis	Gck, Hk1, Pfkm, Pklr
			MP:0011182	decreased hematopoietic cell number	2.04E-01	2.80E-01	2	9	2013	21447	22.22%	9.39%	0.10%	0.04%	Unique to glycolysis	Hk1, Pklr
			MP:0000849	abnormal cerebellum morphology	2.04E-01	2.80E-01	1	9	538	21447	11.11%	2.51%	0.19%	0.04%	Unique to glycolysis	Hk2
			MP:0000351	increased cell proliferation	2.10E-01	2.86E-01	1	9	555	21447	11.11%	2.59%	0.18%	0.04%	Unique to glycolysis	Gck
			MP:0002833	increased heart weight	2.11E-01	2.86E-01	1	9	556	21447	11.11%	2.59%	0.18%	0.04%	Unique to glycolysis	Pfkm
			MP:0005378	growth/size/body region phenotype	2.12E-01	2.87E-01	5	9	7992	21447	55.56%	37.26%	0.06%	0.04%	Unique to glycolysis	Gck, Hk1, Hk2, Pfkm, Pklr
			MP:0003091	abnormal cell migration	2.14E-01	2.89E-01	1	9	565	21447	11.11%	2.63%	0.18%	0.04%	Unique to glycolysis	Hk2
			MP:0001545	abnormal hematopoietic system physiology	2.15E-01	2.89E-01	2	9	2083	21447	22.22%	9.71%	0.10%	0.04%	Unique to glycolysis	Pfkm, Pklr
			MP:0003921	abnormal heart left ventricle morphology	2.16E-01	2.89E-01	1	9	571	21447	11.11%	2.66%	0.18%	0.04%	Unique to glycolysis	Pfkm
			MP:0000701	abnormal lymph node size	2.16E-01	2.89E-01	1	9	573	21447	11.11%	2.67%	0.17%	0.04%	Unique to glycolysis	Hk3
			MP:0000847	abnormal metencephalon morphology	2.17E-01	2.89E-01	1	9	576	21447	11.11%	2.69%	0.17%	0.04%	Unique to glycolysis	Hk2
			MP:0000259	abnormal vascular development	2.18E-01	2.89E-01	1	9	577	21447	11.11%	2.69%	0.17%	0.04%	Unique to glycolysis	Pfkm
			MP:0001256	abnormal body length	2.20E-01	2.91E-01	1	9	583	21447	11.11%	2.72%	0.17%	0.04%	Unique to glycolysis	Pfkm
			MP:0002406	increased susceptibility to infection	2.21E-01	2.91E-01	1	9	586	21447	11.11%	2.73%	0.17%	0.04%	Unique to glycolysis	Pklr
			MP:0011085	postnatal lethality, complete penetrance	2.21E-01	2.91E-01	1	9	586	21447	11.11%	2.73%	0.17%	0.04%	Unique to glycolysis	Gck
			MP:0009643	abnormal urine homeostasis	2.22E-01	2.91E-01	1	9	590	21447	11.11%	2.75%	0.17%	0.04%	Unique to glycolysis	Gck
			MP:0011969	abnormal circulating triglyceride level	2.22E-01	2.91E-01	1	9	591	21447	11.11%	2.76%	0.17%	0.04%	Unique to glycolysis	Gck
			MP:0000202	abnormal circulating alkaline phosphatase level	2.29E-01	2.98E-01	1	9	610	21447	11.11%	2.84%	0.16%	0.04%	Unique to glycolysis	Gck
			MP:0005367	renal/urinary system phenotype	2.34E-01	3.03E-01	2	9	2201	21447	22.22%	10.26%	0.09%	0.04%	Unique to glycolysis	Gck, Hk1
			MP:0001260	increased body weight	2.35E-01	3.03E-01	1	9	628	21447	11.11%	2.93%	0.16%	0.04%	Unique to glycolysis	Gck
			MP:0012323	increased total tissue mass	2.35E-01	3.03E-01	1	9	628	21447	11.11%	2.93%	0.16%	0.04%	Unique to glycolysis	Gck
			MP:0005387	immune system phenotype	2.40E-01	3.09E-01	4	9	6144	21447	44.44%	28.65%	0.07%	0.04%	Unique to glycolysis	Hk1, Hk3, Pfkm, Pklr
			MP:0000841	abnormal hindbrain morphology	2.42E-01	3.09E-01	1	9	649	21447	11.11%	3.03%	0.15%	0.04%	Unique to glycolysis	Hk2
			MP:0009931	abnormal skin appearance	2.42E-01	3.09E-01	1	9	649	21447	11.11%	3.03%	0.15%	0.04%	Unique to glycolysis	Hk1
			MP:0000759	abnormal skeletal muscle morphology	2.46E-01	3.13E-01	1	9	661	21447	11.11%	3.08%	0.15%	0.04%	Unique to glycolysis	Pfkm
			MP:0003948	abnormal gas homeostasis	2.50E-01	3.18E-01	1	9	675	21447	11.11%	3.15%	0.15%	0.04%	Unique to glycolysis	Pfkm
			MP:0001264	increased body size	2.53E-01	3.20E-01	1	9	683	21447	11.11%	3.18%	0.15%	0.04%	Unique to glycolysis	Gck
			MP:0003959	abnormal lean body mass	2.53E-01	3.20E-01	1	9	684	21447	11.11%	3.19%	0.15%	0.04%	Unique to glycolysis	Pfkm
			MP:0003984	embryonic growth retardation	2.57E-01	3.24E-01	1	9	697	21447	11.11%	3.25%	0.14%	0.04%	Unique to glycolysis	Hk2
			MP:0004857	abnormal heart weight	2.58E-01	3.24E-01	1	9	698	21447	11.11%	3.25%	0.14%	0.04%	Unique to glycolysis	Pfkm
			MP:0004848	abnormal liver size	2.60E-01	3.26E-01	1	9	705	21447	11.11%	3.29%	0.14%	0.04%	Unique to glycolysis	Pfkm
			MP:0002339	abnormal lymph node morphology	2.63E-01	3.28E-01	1	9	714	21447	11.11%	3.33%	0.14%	0.04%	Unique to glycolysis	Hk3
			MP:0005560	decreased circulating glucose level	2.64E-01	3.29E-01	1	9	719	21447	11.11%	3.35%	0.14%	0.04%	Unique to glycolysis	Gck
			MP:0004087	abnormal muscle fiber morphology	2.66E-01	3.31E-01	1	9	725	21447	11.11%	3.38%	0.14%	0.04%	Unique to glycolysis	Pfkm
			MP:0005253	abnormal eye physiology	2.68E-01	3.32E-01	1	9	731	21447	11.11%	3.41%	0.14%	0.04%	Unique to glycolysis	Hkdc1
			MP:0001262	decreased body weight	2.70E-01	3.32E-01	2	9	2418	21447	22.22%	11.27%	0.08%	0.04%	Unique to glycolysis	Gck, Hk2
			MP:0012322	decreased total tissue mass	2.70E-01	3.32E-01	2	9	2419	21447	22.22%	11.28%	0.08%	0.04%	Unique to glycolysis	Gck, Hk2
			MP:0004811	abnormal neuron physiology	2.76E-01	3.39E-01	1	9	756	21447	11.11%	3.52%	0.13%	0.04%	Unique to glycolysis	Hk2
			MP:0001303	abnormal lens morphology	2.77E-01	3.39E-01	1	9	759	21447	11.11%	3.54%	0.13%	0.04%	Unique to glycolysis	Hk3
			MP:0002398	abnormal bone marrow cell morphology/development	2.81E-01	3.43E-01	1	9	771	21447	11.11%	3.59%	0.13%	0.04%	Unique to glycolysis	Pklr
			MP:0001657	abnormal induced morbidity/mortality	2.89E-01	3.52E-01	1	9	798	21447	11.11%	3.72%	0.13%	0.04%	Unique to glycolysis	Pklr
			MP:0001793	abnormal susceptibility to infection	2.93E-01	3.54E-01	1	9	809	21447	11.11%	3.77%	0.12%	0.04%	Unique to glycolysis	Pklr
			MP:0010865	prenatal growth retardation	2.93E-01	3.54E-01	1	9	810	21447	11.11%	3.78%	0.12%	0.04%	Unique to glycolysis	Hk2
			MP:0010630	abnormal cardiac muscle tissue morphology	2.97E-01	3.58E-01	1	9	822	21447	11.11%	3.83%	0.12%	0.04%	Unique to glycolysis	Pfkm
			MP:0005025	abnormal response to infection	3.05E-01	3.67E-01	1	9	851	21447	11.11%	3.97%	0.12%	0.04%	Unique to glycolysis	Pklr
			MP:0000266	abnormal heart morphology	3.06E-01	3.67E-01	2	9	2639	21447	22.22%	12.30%	0.08%	0.04%	Unique to glycolysis	Gck, Pfkm
			MP:0020000	abnormal response to antigen	3.09E-01	3.69E-01	1	9	863	21447	11.11%	4.02%	0.12%	0.04%	Unique to glycolysis	Pklr
			MP:0002925	abnormal cardiovascular development	3.09E-01	3.69E-01	1	9	864	21447	11.11%	4.03%	0.12%	0.04%	Unique to glycolysis	Pfkm
			MP:0006207	embryonic lethality during organogenesis	3.25E-01	3.85E-01	1	9	917	21447	11.11%	4.28%	0.11%	0.04%	Unique to glycolysis	Gck

			MP:0005502	abnormal renal/urinary system physiology	3.26E-01	3.85E-01	1	9	918	21447	11.11%	4.28%	0.11%	0.04%	Unique to glycolysis	Gck
			MP:0000913	abnormal brain development	3.26E-01	3.85E-01	1	9	919	21447	11.11%	4.28%	0.11%	0.04%	Unique to glycolysis	Hk2
			MP:0020846	abnormal cell motility	3.29E-01	3.88E-01	1	9	930	21447	11.11%	4.34%	0.11%	0.04%	Unique to glycolysis	Hk2
			MP:0004849	abnormal testis size	3.32E-01	3.90E-01	1	9	940	21447	11.11%	4.38%	0.11%	0.04%	Unique to glycolysis	Pfkm
			MP:0003699	abnormal female reproductive system physiology	3.34E-01	3.91E-01	1	9	946	21447	11.11%	4.41%	0.11%	0.04%	Unique to glycolysis	Hk1
			MP:0002058	neonatal lethality	3.36E-01	3.93E-01	1	9	955	21447	11.11%	4.45%	0.10%	0.04%	Unique to glycolysis	Gck
			MP:0000180	abnormal circulating cholesterol level	3.45E-01	4.02E-01	1	9	984	21447	11.11%	4.59%	0.10%	0.04%	Unique to glycolysis	Gck
			MP:0012321	abnormal total tissue mass	3.50E-01	4.07E-01	2	9	2909	21447	22.22%	13.56%	0.07%	0.04%	Unique to glycolysis	Gck, Hk2
			MP:0003947	abnormal cholesterol level	3.64E-01	4.22E-01	1	9	1052	21447	11.11%	4.91%	0.10%	0.04%	Unique to glycolysis	Gck
			MP:0001732	postnatal growth retardation	3.69E-01	4.26E-01	1	9	1068	21447	11.11%	4.98%	0.09%	0.04%	Unique to glycolysis	Gck
			MP:0012224	abnormal sterol level	3.69E-01	4.26E-01	1	9	1071	21447	11.11%	4.99%	0.09%	0.04%	Unique to glycolysis	Gck
			MP:0005278	abnormal cholesterol homeostasis	3.77E-01	4.34E-01	1	9	1100	21447	11.11%	5.13%	0.09%	0.04%	Unique to glycolysis	Gck
			MP:0001146	abnormal testis morphology	3.80E-01	4.35E-01	1	9	1109	21447	11.11%	5.17%	0.09%	0.04%	Unique to glycolysis	Pfkm
			MP:0001259	abnormal body weight	3.86E-01	4.39E-01	2	9	3125	21447	22.22%	14.57%	0.06%	0.04%	Unique to glycolysis	Gck, Hk2
			MP:0013558	abnormal exocrine gland morphology	3.86E-01	4.39E-01	1	9	1130	21447	11.11%	5.27%	0.09%	0.04%	Unique to glycolysis	Pfkm
			MP:0005452	abnormal adipose tissue amount	3.86E-01	4.39E-01	1	9	1132	21447	11.11%	5.28%	0.09%	0.04%	Unique to glycolysis	Pfkm
			MP:0005294	abnormal heart ventricle morphology	3.88E-01	4.40E-01	1	9	1138	21447	11.11%	5.31%	0.09%	0.04%	Unique to glycolysis	Pfkm
			MP:0001731	abnormal postnatal growth	3.89E-01	4.40E-01	1	9	1144	21447	11.11%	5.33%	0.09%	0.04%	Unique to glycolysis	Gck
			MP:0005193	abnormal anterior eye segment morphology	4.03E-01	4.55E-01	1	9	1196	21447	11.11%	5.58%	0.08%	0.04%	Unique to glycolysis	Hk3
			MP:0002088	abnormal embryonic growth/weight/body size	4.09E-01	4.60E-01	1	9	1216	21447	11.11%	5.67%	0.08%	0.04%	Unique to glycolysis	Hk2
			MP:0001672	abnormal embryo development	4.20E-01	4.71E-01	1	9	1259	21447	11.11%	5.87%	0.08%	0.04%	Unique to glycolysis	Hk2
			MP:0001570	abnormal circulating enzyme level	4.29E-01	4.80E-01	1	9	1294	21447	11.11%	6.03%	0.08%	0.04%	Unique to glycolysis	Gck
			MP:0001924	infertility	4.37E-01	4.87E-01	1	9	1326	21447	11.11%	6.18%	0.08%	0.04%	Unique to glycolysis	Hk1
			MP:0002127	abnormal cardiovascular system morphology	4.40E-01	4.89E-01	2	9	3466	21447	22.22%	16.16%	0.06%	0.04%	Unique to glycolysis	Gck, Pfkm
			MP:0013325	abnormal male reproductive gland morphology	4.41E-01	4.89E-01	1	9	1342	21447	11.11%	6.26%	0.07%	0.04%	Unique to glycolysis	Pfkm
			MP:0000274	enlarged heart	4.43E-01	4.90E-01	1	9	1350	21447	11.11%	6.29%	0.07%	0.04%	Unique to glycolysis	Pfkm
			MP:0002081	perinatal lethality	4.51E-01	4.98E-01	1	9	1382	21447	11.11%	6.44%	0.07%	0.04%	Unique to glycolysis	Gck
			MP:0002082	postnatal lethality	4.53E-01	4.99E-01	1	9	1392	21447	11.11%	6.49%	0.07%	0.04%	Unique to glycolysis	Gck
			MP:0002106	abnormal muscle physiology	4.60E-01	5.05E-01	1	9	1420	21447	11.11%	6.62%	0.07%	0.04%	Unique to glycolysis	Pfkm
			MP:0004196	abnormal prenatal growth/weight/body size	4.64E-01	5.08E-01	1	9	1435	21447	11.11%	6.69%	0.07%	0.04%	Unique to glycolysis	Hk2
			MP:0009205	abnormal internal male genitalia morphology	4.66E-01	5.09E-01	1	9	1445	21447	11.11%	6.74%	0.07%	0.04%	Unique to glycolysis	Pfkm
			MP:0003861	abnormal nervous system development	4.67E-01	5.09E-01	1	9	1450	21447	11.11%	6.76%	0.07%	0.04%	Unique to glycolysis	Hk2
			MP:0009198	abnormal male genitalia morphology	4.72E-01	5.12E-01	1	9	1471	21447	11.11%	6.86%	0.07%	0.04%	Unique to glycolysis	Pfkm
			MP:0001145	abnormal male reproductive system morphology	4.72E-01	5.12E-01	1	9	1471	21447	11.11%	6.86%	0.07%	0.04%	Unique to glycolysis	Pfkm
			MP:0003949	abnormal circulating lipid level	4.87E-01	5.26E-01	1	9	1532	21447	11.11%	7.14%	0.07%	0.04%	Unique to glycolysis	Gck
			MP:0002135	abnormal kidney morphology	4.92E-01	5.30E-01	1	9	1553	21447	11.11%	7.24%	0.06%	0.04%	Unique to glycolysis	Hk1
			MP:0000003	abnormal adipose tissue morphology	4.96E-01	5.33E-01	1	9	1573	21447	11.11%	7.33%	0.06%	0.04%	Unique to glycolysis	Pfkm
			MP:0000653	abnormal sex gland morphology	5.00E-01	5.36E-01	1	9	1590	21447	11.11%	7.41%	0.06%	0.04%	Unique to glycolysis	Pfkm
			MP:0005375	adipose tissue phenotype	5.08E-01	5.42E-01	1	9	1624	21447	11.11%	7.57%	0.06%	0.04%	Unique to glycolysis	Pfkm
			MP:0005389	reproductive system phenotype	5.08E-01	5.42E-01	2	9	3905	21447	22.22%	18.21%	0.05%	0.04%	Unique to glycolysis	Hk1, Pfkm
			MP:0005406	abnormal heart size	5.14E-01	5.47E-01	1	9	1653	21447	11.11%	7.71%	0.06%	0.04%	Unique to glycolysis	Pfkm
			MP:0001614	abnormal blood vessel morphology	5.18E-01	5.50E-01	1	9	1672	21447	11.11%	7.80%	0.06%	0.04%	Unique to glycolysis	Pfkm
			MP:0002108	abnormal muscle morphology	5.20E-01	5.50E-01	1	9	1679	21447	11.11%	7.83%	0.06%	0.04%	Unique to glycolysis	Pfkm
			MP:0000516	abnormal renal/urinary system morphology	5.31E-01	5.61E-01	1	9	1730	21447	11.11%	8.07%	0.06%	0.04%	Unique to glycolysis	Hk1
			MP:0002083	premature death	5.44E-01	5.73E-01	1	9	1793	21447	11.11%	8.36%	0.06%	0.04%	Unique to glycolysis	Pfkm
			MP:0000358	abnormal cell morphology	5.54E-01	5.82E-01	1	9	1840	21447	11.11%	8.58%	0.05%	0.04%	Unique to glycolysis	Gck
			MP:0010678	abnormal skin adnexa morphology	5.68E-01	5.96E-01	1	9	1911	21447	11.11%	8.91%	0.05%	0.04%	Unique to glycolysis	Pfkl
			MP:0005416	abnormal circulating protein level	5.81E-01	6.08E-01	1	9	1978	21447	11.11%	9.22%	0.05%	0.04%	Unique to glycolysis	Gck
			MP:0000313	abnormal cell death	5.93E-01	6.19E-01	1	9	2040	21447	11.11%	9.51%	0.05%	0.04%	Unique to glycolysis	Pfkm
			MP:0002161	abnormal fertility/fecundity	6.00E-01	6.23E-01	1	9	2073	21447	11.11%	9.67%	0.05%	0.04%	Unique to glycolysis	Hk1
			MP:0005385	cardiovascular system phenotype	6.02E-01	6.24E-01	2	9	4565	21447	22.22%	21.29%	0.04%	0.04%	Unique to glycolysis	Gck, Pfkm
			MP:0008469	abnormal protein level	6.17E-01	6.38E-01	1	9	2168	21447	11.11%	10.11%	0.05%	0.04%	Unique to glycolysis	Gck
			MP:0002152	abnormal brain morphology	6.47E-01	6.65E-01	1	9	2342	21447	11.11%	10.92%	0.04%	0.04%	Unique to glycolysis	Hk2

			MP:0005380	embryo phenotype	6.47E-01	6.65E-01	1	9	2343	21447	11.11%	10.92%	0.04%	0.04%	Unique to glycolysis	Hk2
			MP:0001919	abnormal reproductive system physiology	6.48E-01	6.65E-01	1	9	2348	21447	11.11%	10.95%	0.04%	0.04%	Unique to glycolysis	Hk1
			MP:0005369	muscle phenotype	6.49E-01	6.65E-01	1	9	2355	21447	11.11%	10.98%	0.04%	0.04%	Unique to glycolysis	Pfkm
			MP:0002160	abnormal reproductive system morphology	6.63E-01	6.78E-01	1	9	2441	21447	11.11%	11.38%	0.04%	0.04%	Unique to glycolysis	Pfkm
			MP:0001544	abnormal cardiovascular system physiology	6.74E-01	6.87E-01	1	9	2508	21447	11.11%	11.69%	0.04%	0.04%	Unique to glycolysis	Gck
			MP:0010832	lethality during fetal growth through weaning	6.78E-01	6.90E-01	1	9	2537	21447	11.11%	11.83%	0.04%	0.04%	Unique to glycolysis	Gck
			MP:0003633	abnormal nervous system physiology	7.07E-01	7.18E-01	1	9	2737	21447	11.11%	12.76%	0.04%	0.04%	Unique to glycolysis	Hk2
			MP:0001790	abnormal immune system physiology	8.16E-01	8.26E-01	1	9	3678	21447	11.11%	17.15%	0.03%	0.04%	Unique to glycolysis	Pklr
			MP:0003632	abnormal nervous system morphology	8.42E-01	8.50E-01	1	9	3970	21447	11.11%	18.51%	0.03%	0.04%	Unique to glycolysis	Hk2
			MP:0003631	nervous system phenotype	9.21E-01	9.28E-01	1	9	5280	21447	11.11%	24.62%	0.02%	0.04%	Unique to glycolysis	Hk2
			MP:0004924	abnormal behavior	9.61E-01	9.65E-01	1	9	6473	21447	11.11%	30.18%	0.02%	0.04%	Unique to glycolysis	Pfkm
			MP:0005386	behavior/neurological phenotype	9.67E-01	9.69E-01	1	9	6756	21447	11.11%	31.50%	0.01%	0.04%	Unique to glycolysis	Pfkm
			MP:0000001	mammalian phenotype	1.00E+00	1.00E+00	9	9	21447	21447	100.00%	100.00%	0.04%	0.04%	Unique to glycolysis	Gck, Hk1, Hk2, Hk3, Hkdc1, Pfkl, Pfkm, Pklr, Pkm
