# Supplementary material for: Identification of Biochemical Pathways Responsible for Distinct Phenotypes Using Gene Ontology Causal Activity Models
Source: bioRxiv. 2023 May 25:2023.05.22.541760. Preprint. [Version 2] doi: 10.1101/2023.05.22.541760 (PMC10245817; doi:10.1101/2023.05.22.541760)
Supplement: Supplementary file 3 [file media-3.pdf]

Genes Used			TermID	Term	P	Q	k	n	K	N	k/n	K/N	k/K	n/N	Qset	Symbols
**glycolysis**	**pyruvate->lactate**		MP:0002640	reticulocytosis	5.61E-07	2.10E-04	4	15	99	21447	26.67%	0.46%	4.04%	0.07%	glycolysis to lactate	Hk1, Ldha, Pfkm, Pklr
Gck (MGI:1270854)	Slc16a3 (MGI:1933438)		MP:0011913	abnormal reticulocyte cell number	9.54E-07	2.10E-04	4	15	113	21447	26.67%	0.53%	3.54%	0.07%	glycolysis to lactate	Hk1, Ldha, Pfkm, Pklr
Hk1 (MGI:96103)	Slc16a1 (MGI:106013)		MP:0002424	abnormal reticulocyte morphology	1.21E-06	2.10E-04	4	15	120	21447	26.67%	0.56%	3.33%	0.07%	glycolysis to lactate	Hk1, Ldha, Pfkm, Pklr
Hk2 (MGI:1315197)	Slc16a8 (MGI:1929519)		MP:0001585	hemolytic anemia	3.13E-06	4.06E-04	3	15	42	21447	20.00%	0.20%	7.14%	0.07%	glycolysis to lactate	Hk1, Ldha, Pklr
Hk3 (MGI:2670962)	Ldhc (MGI:96764)		MP:0005344	increased circulating bilirubin level	7.55E-06	7.85E-04	4	15	190	21447	26.67%	0.89%	2.11%	0.07%	glycolysis to lactate	Hk1, Ldha, Pfkm, Pklr
Hkdc1 (MGI:2384910)	Ldhb (MGI:96763)		MP:0001569	abnormal circulating bilirubin level	9.80E-06	8.49E-04	4	15	203	21447	26.67%	0.95%	1.97%	0.07%	glycolysis to lactate	Hk1, Ldha, Pfkm, Pklr
Pfkm (MGI:97548)	Ldha (MGI:96759)		MP:0005266	abnormal metabolism	5.58E-05	4.14E-03	6	15	1085	21447	40.00%	5.06%	0.55%	0.07%	glycolysis to lactate	Gck, Ldha, Pfkl, Pfkm, Pklr, Slc16a3
Pfkp (MGI:1891833)			MP:0000208	decreased hematocrit	7.21E-05	4.21E-03	4	15	338	21447	26.67%	1.58%	1.18%	0.07%	glycolysis to lactate	Hk1, Ldha, Pfkm, Pklr
Pfkl (MGI:97547)			MP:0005584	abnormal enzyme/coenzyme activity	7.29E-05	4.21E-03	4	15	339	21447	26.67%	1.58%	1.18%	0.07%	glycolysis to lactate	Gck, Ldha, Pfkl, Pklr
Pkm (MGI:97591)			MP:0031085	increased erythrocyte osmotic fragility	8.61E-05	4.48E-03	2	15	20	21447	13.33%	0.09%	10.00%	0.07%	glycolysis to lactate	Pfkm, Pklr
Pklr (MGI:97604)			MP:0013663	increased myeloid cell number	1.28E-04	6.05E-03	4	15	392	21447	26.67%	1.83%	1.02%	0.07%	glycolysis to lactate	Hk1, Ldha, Pfkm, Pklr
			MP:0005563	abnormal hemoglobin content	1.99E-04	8.64E-03	4	15	440	21447	26.67%	2.05%	0.91%	0.07%	glycolysis to lactate	Gck, Hk1, Ldha, Pklr
	not annotated to a phenotype		MP:0002596	abnormal hematocrit	2.30E-04	8.84E-03	4	15	457	21447	26.67%	2.13%	0.88%	0.07%	glycolysis to lactate	Hk1, Ldha, Pfkm, Pklr
			MP:0003657	abnormal erythrocyte osmotic lysis	2.38E-04	8.84E-03	2	15	33	21447	13.33%	0.15%	6.06%	0.07%	glycolysis to lactate	Pfkm, Pklr
			MP:0010163	hemolysis	3.16E-04	1.10E-02	2	15	38	21447	13.33%	0.18%	5.26%	0.07%	glycolysis to lactate	Pfkm, Pklr
			MP:0000598	abnormal liver morphology	4.78E-04	1.55E-02	6	15	1602	21447	40.00%	7.47%	0.37%	0.07%	glycolysis to lactate	Gck, Hk1, Hkdc1, Ldha, Pfkm, Slc16a1
			MP:0000874	irregular external granule cell layer thickness	6.99E-04	2.03E-02	1	15	1	21447	6.67%	0.00%	100.00%	0.07%	glycolysis to lactate	Hk2
			MP:0002590	increased mean corpuscular volume	7.03E-04	2.03E-02	3	15	258	21447	20.00%	1.20%	1.16%	0.07%	glycolysis to lactate	Hk1, Ldha, Pklr
			MP:0002138	abnormal hepatobiliary system morphology	8.19E-04	2.15E-02	6	15	1771	21447	40.00%	8.26%	0.34%	0.07%	glycolysis to lactate	Gck, Hk1, Hkdc1, Ldha, Pfkm, Slc16a1
			MP:0004952	increased spleen weight	8.29E-04	2.15E-02	3	15	273	21447	20.00%	1.27%	1.10%	0.07%	glycolysis to lactate	Hk1, Ldha, Pfkm
			MP:0003656	abnormal erythrocyte physiology	9.26E-04	2.29E-02	2	15	65	21447	13.33%	0.30%	3.08%	0.07%	glycolysis to lactate	Pfkm, Pklr
			MP:0002943	abnormal lactate dehydrogenase level	9.83E-04	2.32E-02	2	15	67	21447	13.33%	0.31%	2.99%	0.07%	glycolysis to lactate	Ldha, Pfkm
			MP:0002874	decreased hemoglobin content	1.23E-03	2.69E-02	3	15	313	21447	20.00%	1.46%	0.96%	0.07%	glycolysis to lactate	Hk1, Ldha, Pklr
			MP:0002942	decreased circulating alanine transaminase level	1.26E-03	2.69E-02	2	15	76	21447	13.33%	0.35%	2.63%	0.07%	glycolysis to lactate	Ldha, Slc16a1
			MP:0002727	decreased circulating insulin level	1.31E-03	2.69E-02	3	15	320	21447	20.00%	1.49%	0.94%	0.07%	glycolysis to lactate	Gck, Pfkm, Slc16a1
			MP:0011604	abnormal glucokinase activity	1.40E-03	2.69E-02	1	15	2	21447	6.67%	0.01%	50.00%	0.07%	glycolysis to lactate	Gck
			MP:0011606	decreased glucokinase activity	1.40E-03	2.69E-02	1	15	2	21447	6.67%	0.01%	50.00%	0.07%	glycolysis to lactate	Gck
			MP:0005370	liver/biliary system phenotype	1.56E-03	2.91E-02	6	15	2002	21447	40.00%	9.33%	0.30%	0.07%	glycolysis to lactate	Gck, Hk1, Hkdc1, Ldha, Pfkm, Slc16a1
			MP:0012106	impaired exercise endurance	2.13E-03	3.71E-02	2	15	99	21447	13.33%	0.46%	2.02%	0.07%	glycolysis to lactate	Pfkm, Slc16a3
			MP:0002875	decreased erythrocyte cell number	2.14E-03	3.71E-02	3	15	380	21447	20.00%	1.77%	0.79%	0.07%	glycolysis to lactate	Hk1, Ldha, Pklr
			MP:0001577	anemia	2.31E-03	3.82E-02	3	15	390	21447	20.00%	1.82%	0.77%	0.07%	glycolysis to lactate	Hk1, Ldha, Pklr
			MP:0004951	abnormal spleen weight	2.41E-03	3.82E-02	3	15	396	21447	20.00%	1.85%	0.76%	0.07%	glycolysis to lactate	Hk1, Ldha, Pfkm
			MP:0013707	abnormal hematopoietic precursor cell morphology	2.43E-03	3.82E-02	3	15	397	21447	20.00%	1.85%	0.76%	0.07%	glycolysis to lactate	Ldha, Pfkm, Pklr
			MP:0002899	fatigue	2.62E-03	3.98E-02	2	15	110	21447	13.33%	0.51%	1.82%	0.07%	glycolysis to lactate	Pfkm, Slc16a3
			MP:0013661	abnormal myeloid cell number	2.74E-03	3.98E-02	4	15	886	21447	26.67%	4.13%	0.45%	0.07%	glycolysis to lactate	Hk1, Ldha, Pfkm, Pklr
			MP:0005291	abnormal glucose tolerance	2.75E-03	3.98E-02	4	15	887	21447	26.67%	4.14%	0.45%	0.07%	glycolysis to lactate	Gck, Hkdc1, Pfkm, Slc16a1
			MP:0001588	abnormal hemoglobin	3.32E-03	4.66E-02	4	15	934	21447	26.67%	4.35%	0.43%	0.07%	glycolysis to lactate	Gck, Hk1, Ldha, Pklr
			MP:0002332	abnormal exercise endurance	3.53E-03	4.83E-02	2	15	128	21447	13.33%	0.60%	1.56%	0.07%	glycolysis to lactate	Pfkm, Slc16a3
			MP:0002447	abnormal erythrocyte morphology	3.96E-03	5.24E-02	5	15	1629	21447	33.33%	7.60%	0.31%	0.07%	glycolysis to lactate	Gck, Hk1, Ldha, Pfkm, Pklr
			MP:0006410	abnormal common myeloid progenitor cell morphology	4.03E-03	5.24E-02	2	15	137	21447	13.33%	0.64%	1.46%	0.07%	glycolysis to lactate	Ldha, Pklr
			MP:0002814	hyperchromasia	4.19E-03	5.31E-02	1	15	6	21447	6.67%	0.03%	16.67%	0.07%	glycolysis to lactate	Hk1
			MP:0000691	enlarged spleen	4.37E-03	5.41E-02	4	15	1008	21447	26.67%	4.70%	0.40%	0.07%	glycolysis to lactate	Hk1, Ldha, Pfkm, Pklr
			MP:0000226	abnormal mean corpuscular volume	4.47E-03	5.41E-02	3	15	493	21447	20.00%	2.30%	0.61%	0.07%	glycolysis to lactate	Hk1, Ldha, Pklr
			MP:0013659	abnormal erythroid lineage cell morphology	4.75E-03	5.56E-02	5	15	1699	21447	33.33%	7.92%	0.29%	0.07%	glycolysis to lactate	Gck, Hk1, Ldha, Pfkm, Pklr
			MP:0006353	increased glycosylated hemoglobin level	4.89E-03	5.56E-02	1	15	7	21447	6.67%	0.03%	14.29%	0.07%	glycolysis to lactate	Gck
			MP:0001586	abnormal erythrocyte cell number	4.91E-03	5.56E-02	3	15	510	21447	20.00%	2.38%	0.59%	0.07%	glycolysis to lactate	Hk1, Ldha, Pklr
			MP:0003059	decreased insulin secretion	5.38E-03	5.96E-02	2	15	159	21447	13.33%	0.74%	1.26%	0.07%	glycolysis to lactate	Gck, Pfkm
			MP:0000245	abnormal erythropoiesis	5.65E-03	6.12E-02	2	15	163	21447	13.33%	0.76%	1.23%	0.07%	glycolysis to lactate	Hk1, Pklr
			MP:0006351	abnormal glycosylated hemoglobin level	6.28E-03	6.56E-02	1	15	9	21447	6.67%	0.04%	11.11%	0.07%	glycolysis to lactate	Gck

			MP:0005437	abnormal glycogen level	6.41E-03	6.56E-02	2	15	174	21447	13.33%	0.81%	1.15%	0.07%	glycolysis to lactate	Gck, Pfkm
			MP:0005293	impaired glucose tolerance	6.44E-03	6.56E-02	3	15	562	21447	20.00%	2.62%	0.53%	0.07%	glycolysis to lactate	Gck, Hkdc1, Pfkm
			MP:0031318	enhanced skeletal muscle regeneration	7.67E-03	7.50E-02	1	15	11	21447	6.67%	0.05%	9.09%	0.07%	glycolysis to lactate	Pfkm
			MP:0005438	abnormal glycogen homeostasis	7.75E-03	7.50E-02	2	15	192	21447	13.33%	0.90%	1.04%	0.07%	glycolysis to lactate	Gck, Pfkm
			MP:0002221	abnormal lymph organ size	8.07E-03	7.50E-02	5	15	1924	21447	33.33%	8.97%	0.26%	0.07%	glycolysis to lactate	Hk1, Hk3, Ldha, Pfkm, Pklr
			MP:0010034	abnormal erythrocyte clearance	8.36E-03	7.50E-02	1	15	12	21447	6.67%	0.06%	8.33%	0.07%	glycolysis to lactate	Pklr
			MP:0013404	decreased circulating lactate level	8.36E-03	7.50E-02	1	15	12	21447	6.67%	0.06%	8.33%	0.07%	glycolysis to lactate	Pfkm
			MP:0010035	increased erythrocyte clearance	8.36E-03	7.50E-02	1	15	12	21447	6.67%	0.06%	8.33%	0.07%	glycolysis to lactate	Pklr
			MP:0013662	decreased myeloid cell number	8.36E-03	7.50E-02	3	15	618	21447	20.00%	2.88%	0.49%	0.07%	glycolysis to lactate	Hk1, Ldha, Pklr
			MP:0001560	abnormal circulating insulin level	8.97E-03	7.85E-02	3	15	634	21447	20.00%	2.96%	0.47%	0.07%	glycolysis to lactate	Gck, Pfkm, Slc16a1
			MP:0005639	hemosiderosis	9.06E-03	7.85E-02	1	15	13	21447	6.67%	0.06%	7.69%	0.07%	glycolysis to lactate	Pklr
			MP:0013328	visceromegaly	9.42E-03	8.03E-02	4	15	1253	21447	26.67%	5.84%	0.32%	0.07%	glycolysis to lactate	Hk1, Ldha, Pfkm, Pklr
			MP:0003564	abnormal insulin secretion	1.02E-02	8.28E-02	2	15	222	21447	13.33%	1.04%	0.90%	0.07%	glycolysis to lactate	Gck, Pfkm
			MP:0008859	abnormal hair cycle catagen phase	1.04E-02	8.28E-02	1	15	15	21447	6.67%	0.07%	6.67%	0.07%	glycolysis to lactate	Pfkl
			MP:0013161	enhanced muscle regeneration	1.04E-02	8.28E-02	1	15	15	21447	6.67%	0.07%	6.67%	0.07%	glycolysis to lactate	Pfkm
			MP:0010375	increased kidney iron level	1.04E-02	8.28E-02	1	15	15	21447	6.67%	0.07%	6.67%	0.07%	glycolysis to lactate	Hk1
			MP:0014195	abnormal endocrine pancreas secretion	1.05E-02	8.28E-02	2	15	225	21447	13.33%	1.05%	0.89%	0.07%	glycolysis to lactate	Gck, Pfkm
			MP:0008742	abnormal kidney iron level	1.11E-02	8.64E-02	1	15	16	21447	6.67%	0.07%	6.25%	0.07%	glycolysis to lactate	Hk1
			MP:0002694	abnormal pancreas secretion	1.13E-02	8.67E-02	2	15	234	21447	13.33%	1.09%	0.85%	0.07%	glycolysis to lactate	Gck, Pfkm
			MP:0001770	abnormal iron level	1.15E-02	8.68E-02	2	15	236	21447	13.33%	1.10%	0.85%	0.07%	glycolysis to lactate	Hk1, Pklr
			MP:0004848	abnormal liver size	1.20E-02	8.85E-02	3	15	705	21447	20.00%	3.29%	0.43%	0.07%	glycolysis to lactate	Ldha, Pfkm, Slc16a1
			MP:0005637	abnormal iron homeostasis	1.21E-02	8.85E-02	2	15	242	21447	13.33%	1.13%	0.83%	0.07%	glycolysis to lactate	Hk1, Pklr
			MP:0010751	decreased susceptibility to parasitic infection induced morbidity/mortality	1.25E-02	8.92E-02	1	15	18	21447	6.67%	0.08%	5.56%	0.07%	glycolysis to lactate	Pklr
			MP:0011819	increased pancreatic beta cell proliferation	1.25E-02	8.92E-02	1	15	18	21447	6.67%	0.08%	5.56%	0.07%	glycolysis to lactate	Gck
			MP:0003562	abnormal pancreatic beta cell physiology	1.33E-02	9.27E-02	2	15	254	21447	13.33%	1.18%	0.79%	0.07%	glycolysis to lactate	Gck, Pfkm
			MP:0005319	abnormal enzyme/coenzyme level	1.36E-02	9.27E-02	4	15	1395	21447	26.67%	6.50%	0.29%	0.07%	glycolysis to lactate	Gck, Ldha, Pfkm, Slc16a1
			MP:0010147	abnormal endocrine pancreas physiology	1.37E-02	9.27E-02	2	15	258	21447	13.33%	1.20%	0.78%	0.07%	glycolysis to lactate	Gck, Pfkm
			MP:0002224	abnormal spleen size	1.38E-02	9.27E-02	4	15	1400	21447	26.67%	6.53%	0.29%	0.07%	glycolysis to lactate	Hk1, Ldha, Pfkm, Pklr
			MP:0009977	abnormal cerebellar granule cell migration	1.39E-02	9.27E-02	1	15	20	21447	6.67%	0.09%	5.00%	0.07%	glycolysis to lactate	Hk2
			MP:0000187	abnormal triglyceride level	1.52E-02	1.00E-01	3	15	771	21447	20.00%	3.59%	0.39%	0.07%	glycolysis to lactate	Gck, Hkdc1, Slc16a1
			MP:0011108	embryonic lethality during organogenesis, incomplete penetrance	1.64E-02	1.06E-01	2	15	284	21447	13.33%	1.32%	0.70%	0.07%	glycolysis to lactate	Gck, Ldha
			MP:0003731	abnormal retina outer nuclear layer morphology	1.66E-02	1.07E-01	2	15	286	21447	13.33%	1.33%	0.70%	0.07%	glycolysis to lactate	Hkdc1, Pkm
			MP:0010401	increased skeletal muscle glycogen level	1.73E-02	1.09E-01	1	15	25	21447	6.67%	0.12%	4.00%	0.07%	glycolysis to lactate	Pfkm
			MP:0009414	skeletal muscle fiber necrosis	1.73E-02	1.09E-01	1	15	25	21447	6.67%	0.12%	4.00%	0.07%	glycolysis to lactate	Pfkm
			MP:0005571	decreased lactate dehydrogenase level	1.87E-02	1.16E-01	1	15	27	21447	6.67%	0.13%	3.70%	0.07%	glycolysis to lactate	Ldha
			MP:0002722	abnormal immune system organ morphology	1.94E-02	1.19E-01	5	15	2381	21447	33.33%	11.10%	0.21%	0.07%	glycolysis to lactate	Hk1, Hk3, Ldha, Pfkm, Pklr
			MP:0014071	increased cardiac muscle glycogen level	2.01E-02	1.21E-01	1	15	29	21447	6.67%	0.14%	3.45%	0.07%	glycolysis to lactate	Pfkm
			MP:0001265	decreased body size	2.03E-02	1.21E-01	6	15	3351	21447	40.00%	15.62%	0.18%	0.07%	glycolysis to lactate	Gck, Hk1, Hk2, Ldha, Pfkm, Slc16a1
			MP:0010087	increased circulating fructosamine level	2.08E-02	1.23E-01	1	15	30	21447	6.67%	0.14%	3.33%	0.07%	glycolysis to lactate	Gck
			MP:0003413	hair follicle degeneration	2.15E-02	1.25E-01	1	15	31	21447	6.67%	0.14%	3.23%	0.07%	glycolysis to lactate	Pfkl
			MP:0009412	skeletal muscle fiber degeneration	2.22E-02	1.26E-01	1	15	32	21447	6.67%	0.15%	3.12%	0.07%	glycolysis to lactate	Pfkm
			MP:0004847	abnormal liver weight	2.27E-02	1.26E-01	2	15	338	21447	13.33%	1.58%	0.59%	0.07%	glycolysis to lactate	Ldha, Slc16a1
			MP:0014070	abnormal cardiac muscle glycogen level	2.28E-02	1.26E-01	1	15	33	21447	6.67%	0.15%	3.03%	0.07%	glycolysis to lactate	Pfkm
			MP:0011178	increased erythroblast number	2.28E-02	1.26E-01	1	15	33	21447	6.67%	0.15%	3.03%	0.07%	glycolysis to lactate	Pklr
			MP:0009113	increased pancreatic beta cell mass	2.28E-02	1.26E-01	1	15	33	21447	6.67%	0.15%	3.03%	0.07%	glycolysis to lactate	Gck
			MP:0000601	small liver	2.35E-02	1.26E-01	2	15	344	21447	13.33%	1.60%	0.58%	0.07%	glycolysis to lactate	Pfkm, Slc16a1

			MP:0011093	embryonic lethality at implantation, complete penetrance	2.35E-02	1.26E-01	1	15	34	21447	6.67%	0.16%	2.94%	0.07%	glycolysis to lactate	Pkm
			MP:0011628	increased mitochondrial number	2.35E-02	1.26E-01	1	15	34	21447	6.67%	0.16%	2.94%	0.07%	glycolysis to lactate	Gck
			MP:0002164	abnormal gland physiology	2.52E-02	1.34E-01	3	15	932	21447	20.00%	4.35%	0.32%	0.07%	glycolysis to lactate	Gck, Ldhc, Pfkm
			MP:0010770	preweaning lethality	2.57E-02	1.35E-01	8	15	5731	21447	53.33%	26.72%	0.14%	0.07%	glycolysis to lactate	Gck, Hk2, Hkdc1, Ldha, Pfkl, Pfkm, Pkm, Slc16a1
			MP:0013403	abnormal circulating lactate level	2.63E-02	1.37E-01	1	15	38	21447	6.67%	0.18%	2.63%	0.07%	glycolysis to lactate	Pfkm
			MP:0002944	increased lactate dehydrogenase level	2.69E-02	1.37E-01	1	15	39	21447	6.67%	0.18%	2.56%	0.07%	glycolysis to lactate	Pfkm
			MP:0005097	polychromatophilia	2.69E-02	1.37E-01	1	15	39	21447	6.67%	0.18%	2.56%	0.07%	glycolysis to lactate	Hk1
			MP:0005318	decreased triglyceride level	2.73E-02	1.38E-01	2	15	373	21447	13.33%	1.74%	0.54%	0.07%	glycolysis to lactate	Hkdc1, Slc16a1
			MP:0005039	hypoxia	2.76E-02	1.38E-01	1	15	40	21447	6.67%	0.19%	2.50%	0.07%	glycolysis to lactate	Pfkm
			MP:0014069	abnormal skeletal muscle glycogen level	2.83E-02	1.38E-01	1	15	41	21447	6.67%	0.19%	2.44%	0.07%	glycolysis to lactate	Pfkm
			MP:0000689	abnormal spleen morphology	2.84E-02	1.38E-01	4	15	1737	21447	26.67%	8.10%	0.23%	0.07%	glycolysis to lactate	Hk1, Ldha, Pfkm, Pklr
			MP:0008807	increased liver iron level	2.90E-02	1.38E-01	1	15	42	21447	6.67%	0.20%	2.38%	0.07%	glycolysis to lactate	Hk1
			MP:0001759	increased urine glucose level	2.90E-02	1.38E-01	1	15	42	21447	6.67%	0.20%	2.38%	0.07%	glycolysis to lactate	Gck
			MP:0003852	skeletal muscle necrosis	2.90E-02	1.38E-01	1	15	42	21447	6.67%	0.20%	2.38%	0.07%	glycolysis to lactate	Pfkm
			MP:0011818	abnormal pancreatic beta cell proliferation	2.97E-02	1.39E-01	1	15	43	21447	6.67%	0.20%	2.33%	0.07%	glycolysis to lactate	Gck
			MP:0031116	microvesicular hepatic steatosis	2.97E-02	1.39E-01	1	15	43	21447	6.67%	0.20%	2.33%	0.07%	glycolysis to lactate	Gck
			MP:0011177	abnormal erythroblast number	3.10E-02	1.43E-01	1	15	45	21447	6.67%	0.21%	2.22%	0.07%	glycolysis to lactate	Pklr
			MP:0008527	embryonic lethality at implantation	3.10E-02	1.43E-01	1	15	45	21447	6.67%	0.21%	2.22%	0.07%	glycolysis to lactate	Pkm
			MP:0011110	preweaning lethality, incomplete penetrance	3.22E-02	1.44E-01	3	15	1025	21447	20.00%	4.78%	0.29%	0.07%	glycolysis to lactate	Gck, Ldha, Pfkm
			MP:0001758	abnormal urine glucose level	3.24E-02	1.44E-01	1	15	47	21447	6.67%	0.22%	2.13%	0.07%	glycolysis to lactate	Gck
			MP:0030970	ketosis	3.24E-02	1.44E-01	1	15	47	21447	6.67%	0.22%	2.13%	0.07%	glycolysis to lactate	Gck
			MP:0002693	abnormal pancreas physiology	3.25E-02	1.44E-01	2	15	410	21447	13.33%	1.91%	0.49%	0.07%	glycolysis to lactate	Gck, Pfkm
			MP:0002801	abnormal long term object recognition memory	3.31E-02	1.44E-01	1	15	48	21447	6.67%	0.22%	2.08%	0.07%	glycolysis to lactate	Slc16a3
			MP:0008963	increased carbon dioxide production	3.31E-02	1.44E-01	1	15	48	21447	6.67%	0.22%	2.08%	0.07%	glycolysis to lactate	Slc16a1
			MP:0009415	skeletal muscle degeneration	3.37E-02	1.46E-01	1	15	49	21447	6.67%	0.23%	2.04%	0.07%	glycolysis to lactate	Pfkm
			MP:0001547	abnormal lipid level	3.39E-02	1.46E-01	4	15	1834	21447	26.67%	8.55%	0.22%	0.07%	glycolysis to lactate	Gck, Hkdc1, Ldha, Slc16a1
			MP:0013237	abnormal skeletal muscle regeneration	3.44E-02	1.47E-01	1	15	50	21447	6.67%	0.23%	2.00%	0.07%	glycolysis to lactate	Pfkm
			MP:0002768	small adrenal glands	3.51E-02	1.48E-01	1	15	51	21447	6.67%	0.24%	1.96%	0.07%	glycolysis to lactate	Hk3
			MP:0008738	abnormal liver iron level	3.64E-02	1.53E-01	1	15	53	21447	6.67%	0.25%	1.89%	0.07%	glycolysis to lactate	Hk1
			MP:0011084	lethality at weaning, incomplete penetrance	3.71E-02	1.54E-01	1	15	54	21447	6.67%	0.25%	1.85%	0.07%	glycolysis to lactate	Pfkm
			MP:0011091	prenatal lethality, complete penetrance	3.76E-02	1.54E-01	2	15	444	21447	13.33%	2.07%	0.45%	0.07%	glycolysis to lactate	Pkm, Slc16a1
			MP:0011176	abnormal erythroblast morphology	3.78E-02	1.54E-01	1	15	55	21447	6.67%	0.26%	1.82%	0.07%	glycolysis to lactate	Pklr
			MP:0009172	small pancreatic islets	3.78E-02	1.54E-01	1	15	55	21447	6.67%	0.26%	1.82%	0.07%	glycolysis to lactate	Gck
			MP:0008511	decreased retina inner nuclear layer thickness	3.91E-02	1.57E-01	1	15	57	21447	6.67%	0.27%	1.75%	0.07%	glycolysis to lactate	Hkdc1
			MP:0003135	increased erythroid progenitor cell number	4.05E-02	1.57E-01	1	15	59	21447	6.67%	0.28%	1.69%	0.07%	glycolysis to lactate	Pklr
			MP:0009395	increased nucleated erythrocyte cell number	4.05E-02	1.57E-01	1	15	59	21447	6.67%	0.28%	1.69%	0.07%	glycolysis to lactate	Pklr
			MP:0001573	abnormal circulating alanine transaminase level	4.06E-02	1.57E-01	2	15	463	21447	13.33%	2.16%	0.43%	0.07%	glycolysis to lactate	Ldha, Slc16a1
			MP:0005452	abnormal adipose tissue amount	4.15E-02	1.57E-01	3	15	1132	21447	20.00%	5.28%	0.27%	0.07%	glycolysis to lactate	Ldha, Pfkm, Slc16a1
			MP:0013561	abnormal endocrine gland physiology	4.17E-02	1.57E-01	2	15	470	21447	13.33%	2.19%	0.43%	0.07%	glycolysis to lactate	Gck, Pfkm
			MP:0011996	abnormal retina inner nuclear layer thickness	4.18E-02	1.57E-01	1	15	61	21447	6.67%	0.28%	1.64%	0.07%	glycolysis to lactate	Hkdc1
			MP:0002118	abnormal lipid homeostasis	4.20E-02	1.57E-01	4	15	1962	21447	26.67%	9.15%	0.20%	0.07%	glycolysis to lactate	Gck, Hkdc1, Ldha, Slc16a1
			MP:0012397	abnormal nucleated erythrocyte cell number	4.25E-02	1.57E-01	1	15	62	21447	6.67%	0.29%	1.61%	0.07%	glycolysis to lactate	Pklr
			MP:0011100	preweaning lethality, complete penetrance	4.27E-02	1.57E-01	4	15	1972	21447	26.67%	9.19%	0.20%	0.07%	glycolysis to lactate	Hkdc1, Ldha, Pfkl, Pkm
			MP:0011181	increased hematopoietic cell number	4.31E-02	1.57E-01	4	15	1978	21447	26.67%	9.22%	0.20%	0.07%	glycolysis to lactate	Hk1, Ldha, Pfkm, Pklr
			MP:0008962	abnormal carbon dioxide production	4.32E-02	1.57E-01	1	15	63	21447	6.67%	0.29%	1.59%	0.07%	glycolysis to lactate	Slc16a1
			MP:0003926	decreased cellular glucose uptake	4.32E-02	1.57E-01	1	15	63	21447	6.67%	0.29%	1.59%	0.07%	glycolysis to lactate	Hkdc1

			MP:0009114	decreased pancreatic beta cell mass	4.32E-02	1.57E-01	1	15	63	21447	6.67%	0.29%	1.59%	0.07%	glycolysis to lactate	Gck
			MP:0005630	increased lung weight	4.32E-02	1.57E-01	1	15	63	21447	6.67%	0.29%	1.59%	0.07%	glycolysis to lactate	Ldha
			MP:0008518	retina outer nuclear layer degeneration	4.39E-02	1.57E-01	1	15	64	21447	6.67%	0.30%	1.56%	0.07%	glycolysis to lactate	Hkdc1
			MP:0010768	mortality/aging	4.40E-02	1.57E-01	9	15	7559	21447	60.00%	35.25%	0.12%	0.07%	glycolysis to lactate	Gck, Hk2, Hkdc1, Ldha, Pfkl, Pfkm, Pklr, Pkm, Slc16a1
			MP:0010025	decreased total body fat amount	4.44E-02	1.57E-01	2	15	487	21447	13.33%	2.27%	0.41%	0.07%	glycolysis to lactate	Ldha, Pfkm
			MP:0014068	abnormal muscle glycogen level	4.45E-02	1.57E-01	1	15	65	21447	6.67%	0.30%	1.54%	0.07%	glycolysis to lactate	Pfkm
			MP:0005418	abnormal circulating hormone level	4.47E-02	1.57E-01	3	15	1166	21447	20.00%	5.44%	0.26%	0.07%	glycolysis to lactate	Gck, Pfkm, Slc16a1
			MP:0002123	abnormal definitive hematopoiesis	4.53E-02	1.58E-01	3	15	1173	21447	20.00%	5.47%	0.26%	0.07%	glycolysis to lactate	Hk1, Ldha, Pklr
			MP:0001258	decreased body length	4.69E-02	1.59E-01	2	15	502	21447	13.33%	2.34%	0.40%	0.07%	glycolysis to lactate	Pfkm, Slc16a1
			MP:0003896	prolonged PR interval	4.72E-02	1.59E-01	1	15	69	21447	6.67%	0.32%	1.45%	0.07%	glycolysis to lactate	Gck
			MP:0013693	abnormal hemopoiesis	4.80E-02	1.59E-01	3	15	1200	21447	20.00%	5.60%	0.25%	0.07%	glycolysis to lactate	Hk1, Ldha, Pklr
			MP:0010398	decreased liver glycogen level	4.85E-02	1.59E-01	1	15	71	21447	6.67%	0.33%	1.41%	0.07%	glycolysis to lactate	Gck
			MP:0010378	increased respiratory quotient	4.85E-02	1.59E-01	1	15	71	21447	6.67%	0.33%	1.41%	0.07%	glycolysis to lactate	Slc16a1
			MP:0008476	increased spleen red pulp amount	4.85E-02	1.59E-01	1	15	71	21447	6.67%	0.33%	1.41%	0.07%	glycolysis to lactate	Hk1
			MP:0013658	abnormal myeloid cell morphology	4.86E-02	1.59E-01	5	15	3016	21447	33.33%	14.06%	0.17%	0.07%	glycolysis to lactate	Gck, Hk1, Ldha, Pfkm, Pklr
			MP:0002080	prenatal lethality	4.88E-02	1.59E-01	5	15	3020	21447	33.33%	14.08%	0.17%	0.07%	glycolysis to lactate	Gck, Hk2, Ldha, Pkm, Slc16a1
			MP:0003956	abnormal body size	4.89E-02	1.59E-01	6	15	4073	21447	40.00%	18.99%	0.15%	0.07%	glycolysis to lactate	Gck, Hk1, Hk2, Ldha, Pfkm, Slc16a1
			MP:0000750	abnormal muscle regeneration	4.92E-02	1.59E-01	1	15	72	21447	6.67%	0.34%	1.39%	0.07%	glycolysis to lactate	Pfkm
			MP:0003058	increased insulin secretion	4.92E-02	1.59E-01	1	15	72	21447	6.67%	0.34%	1.39%	0.07%	glycolysis to lactate	Gck
			MP:0002078	abnormal glucose homeostasis	4.93E-02	1.59E-01	4	15	2065	21447	26.67%	9.63%	0.19%	0.07%	glycolysis to lactate	Gck, Hkdc1, Pfkm, Slc16a1
			MP:0002642	anisocytosis	5.12E-02	1.64E-01	1	15	75	21447	6.67%	0.35%	1.33%	0.07%	glycolysis to lactate	Hk1
			MP:0004884	abnormal testis physiology	5.25E-02	1.68E-01	1	15	77	21447	6.67%	0.36%	1.30%	0.07%	glycolysis to lactate	Ldhc
			MP:0006069	abnormal retina neuronal layer morphology	5.32E-02	1.69E-01	2	15	538	21447	13.33%	2.51%	0.37%	0.07%	glycolysis to lactate	Hkdc1, Pkm
			MP:0004809	increased hematopoietic stem cell number	5.45E-02	1.71E-01	1	15	80	21447	6.67%	0.37%	1.25%	0.07%	glycolysis to lactate	Pfkm
			MP:0012143	decreased a-wave amplitude	5.52E-02	1.71E-01	1	15	81	21447	6.67%	0.38%	1.23%	0.07%	glycolysis to lactate	Hkdc1
			MP:0005026	decreased susceptibility to parasitic infection	5.52E-02	1.71E-01	1	15	81	21447	6.67%	0.38%	1.23%	0.07%	glycolysis to lactate	Pklr
			MP:0000872	abnormal cerebellum external granule cell layer morphology	5.59E-02	1.71E-01	1	15	82	21447	6.67%	0.38%	1.22%	0.07%	glycolysis to lactate	Hk2
			MP:0002833	increased heart weight	5.64E-02	1.71E-01	2	15	556	21447	13.33%	2.59%	0.36%	0.07%	glycolysis to lactate	Ldha, Pfkm
			MP:0012030	abnormal a-wave amplitude	5.65E-02	1.71E-01	1	15	83	21447	6.67%	0.39%	1.20%	0.07%	glycolysis to lactate	Hkdc1
			MP:0011635	abnormal mitochondrial crista morphology	5.65E-02	1.71E-01	1	15	83	21447	6.67%	0.39%	1.20%	0.07%	glycolysis to lactate	Gck
			MP:0012034	abnormal a-wave shape	5.72E-02	1.71E-01	1	15	84	21447	6.67%	0.39%	1.19%	0.07%	glycolysis to lactate	Hkdc1
			MP:0031268	decreased susceptibility to diet-induced hepatic steatosis	5.72E-02	1.71E-01	1	15	84	21447	6.67%	0.39%	1.19%	0.07%	glycolysis to lactate	Slc16a1
			MP:0013657	abnormal blood cell morphology	5.78E-02	1.71E-01	5	15	3161	21447	33.33%	14.74%	0.16%	0.07%	glycolysis to lactate	Gck, Hk1, Ldha, Pfkm, Pklr
			MP:0001666	abnormal intestinal absorption	5.78E-02	1.71E-01	1	15	85	21447	6.67%	0.40%	1.18%	0.07%	glycolysis to lactate	Slc16a1
			MP:0001570	abnormal circulating enzyme level	5.79E-02	1.71E-01	3	15	1294	21447	20.00%	6.03%	0.23%	0.07%	glycolysis to lactate	Gck, Ldha, Slc16a1
			MP:0002416	abnormal proerythroblast morphology	5.85E-02	1.72E-01	1	15	86	21447	6.67%	0.40%	1.16%	0.07%	glycolysis to lactate	Pklr
			MP:0003953	abnormal hormone level	5.88E-02	1.72E-01	3	15	1303	21447	20.00%	6.08%	0.23%	0.07%	glycolysis to lactate	Gck, Pfkm, Slc16a1
			MP:0004882	enlarged lung	5.92E-02	1.72E-01	1	15	87	21447	6.67%	0.41%	1.15%	0.07%	glycolysis to lactate	Ldha
			MP:0011634	abnormal mitochondrial inner membrane morphology	5.98E-02	1.72E-01	1	15	88	21447	6.67%	0.41%	1.14%	0.07%	glycolysis to lactate	Gck
			MP:0006358	absent pinna reflex	5.98E-02	1.72E-01	1	15	88	21447	6.67%	0.41%	1.14%	0.07%	glycolysis to lactate	Ldhb
			MP:0009112	abnormal pancreatic beta cell mass	6.05E-02	1.73E-01	1	15	89	21447	6.67%	0.41%	1.12%	0.07%	glycolysis to lactate	Gck
			MP:0001256	abnormal body length	6.13E-02	1.74E-01	2	15	583	21447	13.33%	2.72%	0.34%	0.07%	glycolysis to lactate	Pfkm, Slc16a1
			MP:0005440	increased glycogen level	6.18E-02	1.74E-01	1	15	91	21447	6.67%	0.42%	1.10%	0.07%	glycolysis to lactate	Pfkm
			MP:0011085	postnatal lethality, complete penetrance	6.19E-02	1.74E-01	2	15	586	21447	13.33%	2.73%	0.34%	0.07%	glycolysis to lactate	Gck, Ldha
			MP:0005439	decreased glycogen level	6.25E-02	1.74E-01	1	15	92	21447	6.67%	0.43%	1.09%	0.07%	glycolysis to lactate	Gck
			MP:0011969	abnormal circulating triglyceride level	6.28E-02	1.74E-01	2	15	591	21447	13.33%	2.76%	0.34%	0.07%	glycolysis to lactate	Gck, Slc16a1
			MP:0000749	muscle degeneration	6.31E-02	1.74E-01	1	15	93	21447	6.67%	0.43%	1.08%	0.07%	glycolysis to lactate	Pfkm
			MP:0010769	abnormal survival	6.41E-02	1.74E-01	8	15	6733	21447	53.33%	31.39%	0.12%	0.07%	glycolysis to lactate	Gck, Hk2, Hkdc1, Ldha, Pfkl, Pfkm, Pkm, Slc16a1
			MP:0010519	atrioventricular block	6.44E-02	1.74E-01	1	15	95	21447	6.67%	0.44%	1.05%	0.07%	glycolysis to lactate	Gck
			MP:0005629	abnormal lung weight	6.51E-02	1.74E-01	1	15	96	21447	6.67%	0.45%	1.04%	0.07%	glycolysis to lactate	Ldha

			MP:0011097	embryonic lethality between somite formation and embryo turning, complete penetrance	6.51E-02	1.74E-01	1	15	96	21447	6.67%	0.45%	1.04%	0.07%	glycolysis to lactate	Hk2
			MP:0002429	abnormal blood cell morphology/development	6.64E-02	1.74E-01	3	15	1369	21447	20.00%	6.38%	0.22%	0.07%	glycolysis to lactate	Hk1, Ldha, Pklr
			MP:0012144	decreased b-wave amplitude	6.64E-02	1.74E-01	1	15	98	21447	6.67%	0.46%	1.02%	0.07%	glycolysis to lactate	Hkdc1
			MP:0000202	abnormal circulating alkaline phosphatase level	6.64E-02	1.74E-01	2	15	610	21447	13.33%	2.84%	0.33%	0.07%	glycolysis to lactate	Gck, Ldha
			MP:0008762	embryonic lethality	6.69E-02	1.74E-01	4	15	2280	21447	26.67%	10.63%	0.18%	0.07%	glycolysis to lactate	Gck, Hk2, Ldha, Pkm
			MP:0003727	abnormal retina layer morphology	6.70E-02	1.74E-01	2	15	613	21447	13.33%	2.86%	0.33%	0.07%	glycolysis to lactate	Hkdc1, Pkm
			MP:0002310	decreased susceptibility to hepatic steatosis	6.71E-02	1.74E-01	1	15	99	21447	6.67%	0.46%	1.01%	0.07%	glycolysis to lactate	Slc16a1
			MP:0000427	abnormal hair cycle	6.77E-02	1.74E-01	1	15	100	21447	6.67%	0.47%	1.00%	0.07%	glycolysis to lactate	Pfkl
			MP:0010392	prolonged QRS complex duration	6.77E-02	1.74E-01	1	15	100	21447	6.67%	0.47%	1.00%	0.07%	glycolysis to lactate	Gck
			MP:0012031	abnormal b-wave amplitude	6.84E-02	1.74E-01	1	15	101	21447	6.67%	0.47%	0.99%	0.07%	glycolysis to lactate	Hkdc1
			MP:0012035	abnormal b-wave shape	6.84E-02	1.74E-01	1	15	101	21447	6.67%	0.47%	0.99%	0.07%	glycolysis to lactate	Hkdc1
			MP:0013327	abnormal male reproductive gland physiology	6.84E-02	1.74E-01	1	15	101	21447	6.67%	0.47%	0.99%	0.07%	glycolysis to lactate	Ldhc
			MP:0001485	abnormal pinna reflex	6.84E-02	1.74E-01	1	15	101	21447	6.67%	0.47%	0.99%	0.07%	glycolysis to lactate	Ldhb
			MP:0010636	bundle branch block	6.90E-02	1.74E-01	1	15	102	21447	6.67%	0.48%	0.98%	0.07%	glycolysis to lactate	Gck
			MP:0010560	intraventricular block	6.90E-02	1.74E-01	1	15	102	21447	6.67%	0.48%	0.98%	0.07%	glycolysis to lactate	Gck
			MP:0005459	decreased percent body fat/body weight	6.97E-02	1.75E-01	1	15	103	21447	6.67%	0.48%	0.97%	0.07%	glycolysis to lactate	Slc16a1
			MP:0003901	abnormal PR interval	7.03E-02	1.76E-01	1	15	104	21447	6.67%	0.48%	0.96%	0.07%	glycolysis to lactate	Gck
			MP:0009786	decreased susceptibility to infection induced morbidity/mortality	7.16E-02	1.78E-01	1	15	106	21447	6.67%	0.49%	0.94%	0.07%	glycolysis to lactate	Pklr
			MP:0008569	lethality at weaning	7.23E-02	1.78E-01	1	15	107	21447	6.67%	0.50%	0.93%	0.07%	glycolysis to lactate	Pfkm
			MP:0001458	abnormal object recognition memory	7.30E-02	1.78E-01	1	15	108	21447	6.67%	0.50%	0.93%	0.07%	glycolysis to lactate	Slc16a3
			MP:0012307	impaired spatial learning	7.30E-02	1.78E-01	1	15	108	21447	6.67%	0.50%	0.93%	0.07%	glycolysis to lactate	Slc16a3
			MP:0009788	increased susceptibility to bacterial infection induced morbidity/mortality	7.30E-02	1.78E-01	1	15	108	21447	6.67%	0.50%	0.93%	0.07%	glycolysis to lactate	Pklr
			MP:0004948	abnormal neuronal precursor proliferation	7.43E-02	1.80E-01	1	15	110	21447	6.67%	0.51%	0.91%	0.07%	glycolysis to lactate	Hk2
			MP:0005376	homeostasis/metabolism phenotype	7.48E-02	1.80E-01	9	15	8239	21447	60.00%	38.42%	0.11%	0.07%	glycolysis to lactate	Gck, Hk1, Hkdc1, Ldha, Pfkl, Pfkm, Pklr, Slc16a1, Slc16a3
			MP:0012777	abnormal liver glycogen level	7.49E-02	1.80E-01	1	15	111	21447	6.67%	0.52%	0.90%	0.07%	glycolysis to lactate	Gck
			MP:0005559	increased circulating glucose level	7.50E-02	1.80E-01	2	15	654	21447	13.33%	3.05%	0.31%	0.07%	glycolysis to lactate	Gck, Pfkm
			MP:0001783	decreased white adipose tissue amount	7.56E-02	1.80E-01	1	15	112	21447	6.67%	0.52%	0.89%	0.07%	glycolysis to lactate	Slc16a1
			MP:0010086	abnormal circulating fructosamine level	7.69E-02	1.82E-01	1	15	114	21447	6.67%	0.53%	0.88%	0.07%	glycolysis to lactate	Gck
			MP:0009356	decreased liver triglyceride level	7.75E-02	1.83E-01	1	15	115	21447	6.67%	0.54%	0.87%	0.07%	glycolysis to lactate	Hkdc1
			MP:0002966	decreased circulating alkaline phosphatase level	7.82E-02	1.83E-01	1	15	116	21447	6.67%	0.54%	0.86%	0.07%	glycolysis to lactate	Ldha
			MP:0006206	embryonic lethality between somite formation and embryo turning	7.82E-02	1.83E-01	1	15	116	21447	6.67%	0.54%	0.86%	0.07%	glycolysis to lactate	Hk2
			MP:0001157	small seminal vesicle	7.88E-02	1.83E-01	1	15	117	21447	6.67%	0.55%	0.85%	0.07%	glycolysis to lactate	Pfkm
			MP:0000693	spleen hyperplasia	7.88E-02	1.83E-01	1	15	117	21447	6.67%	0.55%	0.85%	0.07%	glycolysis to lactate	Ldha
			MP:0003948	abnormal gas homeostasis	7.93E-02	1.83E-01	2	15	675	21447	13.33%	3.15%	0.30%	0.07%	glycolysis to lactate	Pfkm, Slc16a1
			MP:0009404	centrally nucleated skeletal muscle fibers	8.01E-02	1.84E-01	1	15	119	21447	6.67%	0.55%	0.84%	0.07%	glycolysis to lactate	Pfkm
			MP:0011925	abnormal heart echocardiography feature	8.20E-02	1.87E-01	1	15	122	21447	6.67%	0.57%	0.82%	0.07%	glycolysis to lactate	Gck
			MP:0001053	abnormal neuromuscular synapse morphology	8.20E-02	1.87E-01	1	15	122	21447	6.67%	0.57%	0.82%	0.07%	glycolysis to lactate	Slc16a3
			MP:0012029	abnormal electroretinogram waveform feature	8.33E-02	1.88E-01	1	15	124	21447	6.67%	0.58%	0.81%	0.07%	glycolysis to lactate	Hkdc1
			MP:0001051	abnormal somatic motor system morphology	8.33E-02	1.88E-01	1	15	124	21447	6.67%	0.58%	0.81%	0.07%	glycolysis to lactate	Slc16a3
			MP:0004857	abnormal heart weight	8.40E-02	1.89E-01	2	15	698	21447	13.33%	3.25%	0.29%	0.07%	glycolysis to lactate	Ldha, Pfkm
			MP:0009170	abnormal pancreatic islet size	8.53E-02	1.91E-01	1	15	127	21447	6.67%	0.59%	0.79%	0.07%	glycolysis to lactate	Gck
			MP:0002169	no abnormal phenotype detected	8.64E-02	1.92E-01	4	15	2484	21447	26.67%	11.58%	0.16%	0.07%	glycolysis to lactate	Hk2, Ldha, Pklr, Pkm
			MP:0002873	normal phenotype	8.67E-02	1.92E-01	4	15	2487	21447	26.67%	11.60%	0.16%	0.07%	glycolysis to lactate	Hk2, Ldha, Pklr, Pkm
			MP:0003949	abnormal circulating lipid level	8.68E-02	1.92E-01	3	15	1532	21447	20.00%	7.14%	0.20%	0.07%	glycolysis to lactate	Gck, Ldha, Slc16a1

			MP:0003925	abnormal cellular glucose uptake	8.72E-02	1.92E-01	1	15	130	21447	6.67%	0.61%	0.77%	0.07%	glycolysis to lactate	Hkdc1
			MP:0006141	abnormal atrioventricular node conduction	8.85E-02	1.94E-01	1	15	132	21447	6.67%	0.62%	0.76%	0.07%	glycolysis to lactate	Gck
			MP:0005668	decreased circulating leptin level	8.91E-02	1.94E-01	1	15	133	21447	6.67%	0.62%	0.75%	0.07%	glycolysis to lactate	Slc16a1
			MP:0002089	abnormal postnatal growth/weight/body size	8.92E-02	1.94E-01	6	15	4703	21447	40.00%	21.93%	0.13%	0.07%	glycolysis to lactate	Gck, Hk1, Hk2, Ldha, Pfkm, Slc16a1
			MP:0001664	abnormal digestion	8.98E-02	1.94E-01	1	15	134	21447	6.67%	0.62%	0.75%	0.07%	glycolysis to lactate	Slc16a1
			MP:0005253	abnormal eye physiology	9.09E-02	1.95E-01	2	15	731	21447	13.33%	3.41%	0.27%	0.07%	glycolysis to lactate	Hkdc1, Slc16a8
			MP:0006009	abnormal neuronal migration	9.10E-02	1.95E-01	1	15	136	21447	6.67%	0.63%	0.74%	0.07%	glycolysis to lactate	Hk2
			MP:0008801	abnormal erythroid progenitor cell morphology	9.23E-02	1.95E-01	1	15	138	21447	6.67%	0.64%	0.72%	0.07%	glycolysis to lactate	Pklr
			MP:0003402	decreased liver weight	9.23E-02	1.95E-01	1	15	138	21447	6.67%	0.64%	0.72%	0.07%	glycolysis to lactate	Slc16a1
			MP:0000003	abnormal adipose tissue morphology	9.24E-02	1.95E-01	3	15	1573	21447	20.00%	7.33%	0.19%	0.07%	glycolysis to lactate	Ldha, Pfkm, Slc16a1
			MP:0003898	abnormal QRS complex	9.30E-02	1.95E-01	1	15	139	21447	6.67%	0.65%	0.72%	0.07%	glycolysis to lactate	Gck
			MP:0000189	hypoglycemia	9.30E-02	1.95E-01	1	15	139	21447	6.67%	0.65%	0.72%	0.07%	glycolysis to lactate	Gck
			MP:0003733	abnormal retina inner nuclear layer morphology	9.36E-02	1.95E-01	1	15	140	21447	6.67%	0.65%	0.71%	0.07%	glycolysis to lactate	Hkdc1
			MP:0001554	increased circulating free fatty acids level	9.36E-02	1.95E-01	1	15	140	21447	6.67%	0.65%	0.71%	0.07%	glycolysis to lactate	Gck
			MP:0002625	heart left ventricle hypertrophy	9.55E-02	1.99E-01	1	15	143	21447	6.67%	0.67%	0.70%	0.07%	glycolysis to lactate	Pfkm
			MP:0009967	abnormal neuron proliferation	9.74E-02	2.01E-01	1	15	146	21447	6.67%	0.68%	0.68%	0.07%	glycolysis to lactate	Hk2
			MP:0001148	enlarged testis	9.74E-02	2.01E-01	1	15	146	21447	6.67%	0.68%	0.68%	0.07%	glycolysis to lactate	Pfkm
			MP:0031094	organomegaly	9.90E-02	2.03E-01	4	15	2603	21447	26.67%	12.14%	0.15%	0.07%	glycolysis to lactate	Hk1, Ldha, Pfkm, Pklr
			MP:0005375	adipose tissue phenotype	9.95E-02	2.03E-01	3	15	1624	21447	20.00%	7.57%	0.18%	0.07%	glycolysis to lactate	Ldha, Pfkm, Slc16a1
			MP:0002398	abnormal bone marrow cell morphology/development	9.95E-02	2.03E-01	2	15	771	21447	13.33%	3.59%	0.26%	0.07%	glycolysis to lactate	Ldha, Pklr
			MP:0000854	abnormal cerebellum development	1.02E-01	2.05E-01	1	15	153	21447	6.67%	0.71%	0.65%	0.07%	glycolysis to lactate	Hk2
			MP:0005647	abnormal sex gland physiology	1.02E-01	2.05E-01	1	15	153	21447	6.67%	0.71%	0.65%	0.07%	glycolysis to lactate	Ldhc
			MP:0002356	abnormal spleen red pulp morphology	1.02E-01	2.05E-01	1	15	153	21447	6.67%	0.71%	0.65%	0.07%	glycolysis to lactate	Hk1
			MP:0005289	increased oxygen consumption	1.02E-01	2.05E-01	1	15	153	21447	6.67%	0.71%	0.65%	0.07%	glycolysis to lactate	Slc16a1
			MP:0011940	decreased food intake	1.04E-01	2.09E-01	1	15	157	21447	6.67%	0.73%	0.64%	0.07%	glycolysis to lactate	Slc16a1
			MP:0005402	abnormal action potential	1.09E-01	2.17E-01	1	15	164	21447	6.67%	0.76%	0.61%	0.07%	glycolysis to lactate	Slc16a3
			MP:0008515	thin retina outer nuclear layer	1.09E-01	2.17E-01	1	15	165	21447	6.67%	0.77%	0.61%	0.07%	glycolysis to lactate	Hkdc1
			MP:0011997	abnormal retina outer nuclear layer thickness	1.10E-01	2.18E-01	1	15	166	21447	6.67%	0.77%	0.60%	0.07%	glycolysis to lactate	Hkdc1
			MP:0004808	abnormal hematopoietic stem cell morphology	1.12E-01	2.20E-01	1	15	169	21447	6.67%	0.79%	0.59%	0.07%	glycolysis to lactate	Pfkm
			MP:0012315	impaired learning	1.12E-01	2.20E-01	1	15	169	21447	6.67%	0.79%	0.59%	0.07%	glycolysis to lactate	Slc16a3
			MP:0010559	heart block	1.13E-01	2.20E-01	1	15	170	21447	6.67%	0.79%	0.59%	0.07%	glycolysis to lactate	Gck
			MP:0004129	abnormal respiratory quotient	1.14E-01	2.22E-01	1	15	172	21447	6.67%	0.80%	0.58%	0.07%	glycolysis to lactate	Slc16a1
			MP:0004907	abnormal seminal vesicle size	1.14E-01	2.22E-01	1	15	173	21447	6.67%	0.81%	0.58%	0.07%	glycolysis to lactate	Pfkm
			MP:0002060	abnormal skin morphology	1.16E-01	2.24E-01	3	15	1735	21447	20.00%	8.09%	0.17%	0.07%	glycolysis to lactate	Hk1, Hk3, Pfkm
			MP:0020184	abnormal susceptibility to parasitic infection	1.20E-01	2.31E-01	1	15	182	21447	6.67%	0.85%	0.55%	0.07%	glycolysis to lactate	Pklr
			MP:0005659	decreased susceptibility to diet-induced obesity	1.21E-01	2.33E-01	1	15	184	21447	6.67%	0.86%	0.54%	0.07%	glycolysis to lactate	Slc16a1
			MP:0000639	abnormal adrenal gland morphology	1.23E-01	2.34E-01	1	15	186	21447	6.67%	0.87%	0.54%	0.07%	glycolysis to lactate	Hk3
			MP:0005281	increased fatty acids level	1.27E-01	2.42E-01	1	15	194	21447	6.67%	0.90%	0.52%	0.07%	glycolysis to lactate	Gck
			MP:0005217	abnormal pancreatic beta cell morphology	1.28E-01	2.42E-01	1	15	195	21447	6.67%	0.91%	0.51%	0.07%	glycolysis to lactate	Gck
			MP:0001559	hyperglycemia	1.28E-01	2.42E-01	1	15	195	21447	6.67%	0.91%	0.51%	0.07%	glycolysis to lactate	Gck
			MP:0001781	abnormal white adipose tissue amount	1.29E-01	2.42E-01	1	15	196	21447	6.67%	0.91%	0.51%	0.07%	glycolysis to lactate	Slc16a1
			MP:0009787	increased susceptibility to infection induced morbidity/mortality	1.29E-01	2.42E-01	1	15	196	21447	6.67%	0.91%	0.51%	0.07%	glycolysis to lactate	Pklr
			MP:0013278	decreased fasting circulating glucose level	1.29E-01	2.42E-01	1	15	197	21447	6.67%	0.92%	0.51%	0.07%	glycolysis to lactate	Gck
			MP:0000242	impaired fertilization	1.31E-01	2.44E-01	1	15	200	21447	6.67%	0.93%	0.50%	0.07%	glycolysis to lactate	Ldhc
			MP:0005457	abnormal percent body fat/body weight	1.32E-01	2.46E-01	1	15	202	21447	6.67%	0.94%	0.50%	0.07%	glycolysis to lactate	Slc16a1
			MP:0006207	embryonic lethality during organogenesis	1.33E-01	2.46E-01	2	15	917	21447	13.33%	4.28%	0.22%	0.07%	glycolysis to lactate	Gck, Ldha

			MP:0002981	increased liver weight	1.35E-01	2.50E-01	1	15	207	21447	6.67%	0.97%	0.48%	0.07%	glycolysis to lactate	Ldha
			MP:0020846	abnormal cell motility	1.36E-01	2.50E-01	2	15	930	21447	13.33%	4.34%	0.22%	0.07%	glycolysis to lactate	Hk2, Ldhc
			MP:0005410	abnormal fertilization	1.40E-01	2.57E-01	1	15	215	21447	6.67%	1.00%	0.47%	0.07%	glycolysis to lactate	Ldhc
			MP:0006108	abnormal hindbrain development	1.41E-01	2.57E-01	1	15	216	21447	6.67%	1.01%	0.46%	0.07%	glycolysis to lactate	Hk2
			MP:0010182	decreased susceptibility to weight gain	1.42E-01	2.58E-01	1	15	218	21447	6.67%	1.02%	0.46%	0.07%	glycolysis to lactate	Slc16a1
			MP:0000685	abnormal immune system morphology	1.44E-01	2.60E-01	5	15	4113	21447	33.33%	19.18%	0.12%	0.07%	glycolysis to lactate	Hk1, Hk3, Ldha, Pfkm, Pklr
			MP:0009642	abnormal blood homeostasis	1.47E-01	2.66E-01	6	15	5352	21447	40.00%	24.95%	0.11%	0.07%	glycolysis to lactate	Gck, Hk1, Ldha, Pfkm, Pklr, Slc16a1
			MP:0004881	abnormal lung size	1.48E-01	2.66E-01	1	15	227	21447	6.67%	1.06%	0.44%	0.07%	glycolysis to lactate	Ldha
			MP:0011180	abnormal hematopoietic cell number	1.49E-01	2.66E-01	4	15	3011	21447	26.67%	14.04%	0.13%	0.07%	glycolysis to lactate	Hk1, Ldha, Pfkm, Pklr
			MP:0000180	abnormal circulating cholesterol level	1.49E-01	2.66E-01	2	15	984	21447	13.33%	4.59%	0.20%	0.07%	glycolysis to lactate	Gck, Ldha
			MP:0005288	abnormal oxygen consumption	1.51E-01	2.68E-01	1	15	233	21447	6.67%	1.09%	0.43%	0.07%	glycolysis to lactate	Slc16a1
			MP:0004889	increased energy expenditure	1.52E-01	2.68E-01	1	15	234	21447	6.67%	1.09%	0.43%	0.07%	glycolysis to lactate	Slc16a1
			MP:0010579	increased heart left ventricle size	1.52E-01	2.68E-01	1	15	234	21447	6.67%	1.09%	0.43%	0.07%	glycolysis to lactate	Pfkm
			MP:0002891	increased insulin sensitivity	1.54E-01	2.72E-01	1	15	238	21447	6.67%	1.11%	0.42%	0.07%	glycolysis to lactate	Slc16a1
			MP:0005416	abnormal circulating protein level	1.55E-01	2.72E-01	3	15	1978	21447	20.00%	9.22%	0.15%	0.07%	glycolysis to lactate	Gck, Ldha, Slc16a1
			MP:0000186	decreased circulating HDL cholesterol level	1.55E-01	2.72E-01	1	15	240	21447	6.67%	1.12%	0.42%	0.07%	glycolysis to lactate	Ldha
			MP:0005562	decreased mean corpuscular hemoglobin	1.56E-01	2.72E-01	1	15	241	21447	6.67%	1.12%	0.41%	0.07%	glycolysis to lactate	Hk1
			MP:0000192	abnormal mineral level	1.59E-01	2.76E-01	2	15	1023	21447	13.33%	4.77%	0.20%	0.07%	glycolysis to lactate	Hk1, Pklr
			MP:0012778	abnormal liver triglyceride level	1.59E-01	2.76E-01	1	15	246	21447	6.67%	1.15%	0.41%	0.07%	glycolysis to lactate	Hkdc1
			MP:0011182	decreased hematopoietic cell number	1.61E-01	2.77E-01	3	15	2013	21447	20.00%	9.39%	0.15%	0.07%	glycolysis to lactate	Hk1, Ldha, Pklr
			MP:0005636	abnormal mineral homeostasis	1.61E-01	2.77E-01	2	15	1033	21447	13.33%	4.82%	0.19%	0.07%	glycolysis to lactate	Hk1, Pklr
			MP:0003717	pallor	1.62E-01	2.78E-01	1	15	251	21447	6.67%	1.17%	0.40%	0.07%	glycolysis to lactate	Hk1
			MP:0013656	abnormal hematopoietic cell morphology	1.63E-01	2.78E-01	5	15	4277	21447	33.33%	19.94%	0.12%	0.07%	glycolysis to lactate	Gck, Hk1, Ldha, Pfkm, Pklr
			MP:0013279	increased fasting circulating glucose level	1.65E-01	2.81E-01	1	15	257	21447	6.67%	1.20%	0.39%	0.07%	glycolysis to lactate	Gck
			MP:0005331	insulin resistance	1.65E-01	2.81E-01	1	15	257	21447	6.67%	1.20%	0.39%	0.07%	glycolysis to lactate	Gck
			MP:0003947	abnormal cholesterol level	1.66E-01	2.81E-01	2	15	1052	21447	13.33%	4.91%	0.19%	0.07%	glycolysis to lactate	Gck, Ldha
			MP:0005667	abnormal circulating leptin level	1.67E-01	2.82E-01	1	15	260	21447	6.67%	1.21%	0.38%	0.07%	glycolysis to lactate	Slc16a1
			MP:0001553	abnormal circulating free fatty acids level	1.68E-01	2.83E-01	1	15	262	21447	6.67%	1.22%	0.38%	0.07%	glycolysis to lactate	Gck
			MP:0010771	integument phenotype	1.70E-01	2.85E-01	4	15	3165	21447	26.67%	14.76%	0.13%	0.07%	glycolysis to lactate	Hk1, Hk3, Pfkl, Pfkm
			MP:0012224	abnormal sterol level	1.71E-01	2.85E-01	2	15	1071	21447	13.33%	4.99%	0.19%	0.07%	glycolysis to lactate	Gck, Ldha
			MP:0010680	abnormal skin adnexa physiology	1.75E-01	2.90E-01	1	15	273	21447	6.67%	1.27%	0.37%	0.07%	glycolysis to lactate	Pfkl
			MP:0000377	abnormal hair follicle morphology	1.75E-01	2.90E-01	1	15	274	21447	6.67%	1.28%	0.36%	0.07%	glycolysis to lactate	Pfkl
			MP:0006035	abnormal mitochondrial morphology	1.75E-01	2.90E-01	1	15	274	21447	6.67%	1.28%	0.36%	0.07%	glycolysis to lactate	Gck
			MP:0009785	abnormal susceptibility to infection induced morbidity/mortality	1.77E-01	2.90E-01	1	15	276	21447	6.67%	1.29%	0.36%	0.07%	glycolysis to lactate	Pklr
			MP:0010067	increased red blood cell distribution width	1.77E-01	2.90E-01	1	15	276	21447	6.67%	1.29%	0.36%	0.07%	glycolysis to lactate	Hk1
			MP:0003728	abnormal retina photoreceptor layer morphology	1.78E-01	2.90E-01	1	15	278	21447	6.67%	1.30%	0.36%	0.07%	glycolysis to lactate	Hkdc1
			MP:0009538	abnormal synapse morphology	1.78E-01	2.90E-01	1	15	278	21447	6.67%	1.30%	0.36%	0.07%	glycolysis to lactate	Slc16a3
			MP:0005278	abnormal cholesterol homeostasis	1.78E-01	2.90E-01	2	15	1100	21447	13.33%	5.13%	0.18%	0.07%	glycolysis to lactate	Gck, Ldha
			MP:0001468	abnormal temporal memory	1.78E-01	2.90E-01	1	15	279	21447	6.67%	1.30%	0.36%	0.07%	glycolysis to lactate	Slc16a3
			MP:0010578	abnormal heart left ventricle size	1.80E-01	2.90E-01	1	15	281	21447	6.67%	1.31%	0.36%	0.07%	glycolysis to lactate	Pfkm
			MP:0005391	vision/eye phenotype	1.80E-01	2.90E-01	4	15	3234	21447	26.67%	15.08%	0.12%	0.07%	glycolysis to lactate	Hk3, Hkdc1, Pkm, Slc16a8
			MP:0010358	abnormal free fatty acids level	1.81E-01	2.90E-01	1	15	283	21447	6.67%	1.32%	0.35%	0.07%	glycolysis to lactate	Gck
			MP:0002644	decreased circulating triglyceride level	1.81E-01	2.90E-01	1	15	283	21447	6.67%	1.32%	0.35%	0.07%	glycolysis to lactate	Slc16a1
			MP:0005551	abnormal eye electrophysiology	1.83E-01	2.93E-01	1	15	287	21447	6.67%	1.34%	0.35%	0.07%	glycolysis to lactate	Slc16a8
			MP:0002059	abnormal seminal vesicle morphology	1.86E-01	2.97E-01	1	15	293	21447	6.67%	1.37%	0.34%	0.07%	glycolysis to lactate	Pfkm
			MP:0008469	abnormal protein level	1.88E-01	2.99E-01	3	15	2168	21447	20.00%	10.11%	0.14%	0.07%	glycolysis to lactate	Gck, Ldha, Slc16a1
			MP:0001574	abnormal oxygen level	1.89E-01	3.00E-01	1	15	298	21447	6.67%	1.39%	0.34%	0.07%	glycolysis to lactate	Pfkm
			MP:0013292	embryonic lethality prior to organogenesis	1.90E-01	3.00E-01	2	15	1146	21447	13.33%	5.34%	0.17%	0.07%	glycolysis to lactate	Hk2, Pkm

			MP:0003084	abnormal skeletal muscle fiber morphology	1.91E-01	3.00E-01	1	15	300	21447	6.67%	1.40%	0.33%	0.07%	glycolysis to lactate	Pfkm
			MP:0002628	hepatic steatosis	1.91E-01	3.00E-01	1	15	301	21447	6.67%	1.40%	0.33%	0.07%	glycolysis to lactate	Gck
			MP:0000316	cellular necrosis	1.93E-01	3.03E-01	1	15	305	21447	6.67%	1.42%	0.33%	0.07%	glycolysis to lactate	Pfkm
			MP:0001764	abnormal homeostasis	1.96E-01	3.06E-01	7	15	7085	21447	46.67%	33.03%	0.10%	0.07%	glycolysis to lactate	Gck, Hk1, Hkdc1, Ldha, Pfkm, Pklr, Slc16a1
			MP:0010066	abnormal red blood cell distribution width	1.97E-01	3.06E-01	1	15	311	21447	6.67%	1.45%	0.32%	0.07%	glycolysis to lactate	Hk1
			MP:0005404	abnormal axon morphology	1.99E-01	3.08E-01	1	15	314	21447	6.67%	1.46%	0.32%	0.07%	glycolysis to lactate	Slc16a3
			MP:0001325	abnormal retina morphology	1.99E-01	3.08E-01	2	15	1183	21447	13.33%	5.52%	0.17%	0.07%	glycolysis to lactate	Hkdc1, Pkm
			MP:0002412	increased susceptibility to bacterial infection	2.00E-01	3.08E-01	1	15	316	21447	6.67%	1.47%	0.32%	0.07%	glycolysis to lactate	Pklr
			MP:0005215	abnormal pancreatic islet morphology	2.00E-01	3.08E-01	1	15	317	21447	6.67%	1.48%	0.32%	0.07%	glycolysis to lactate	Gck
			MP:0009165	abnormal endocrine pancreas morphology	2.01E-01	3.08E-01	1	15	318	21447	6.67%	1.48%	0.31%	0.07%	glycolysis to lactate	Gck
			MP:0003141	cardiac fibrosis	2.01E-01	3.08E-01	1	15	319	21447	6.67%	1.49%	0.31%	0.07%	glycolysis to lactate	Gck
			MP:0001765	abnormal ion homeostasis	2.03E-01	3.09E-01	2	15	1196	21447	13.33%	5.58%	0.17%	0.07%	glycolysis to lactate	Hk1, Pklr
			MP:0002409	decreased susceptibility to infection	2.04E-01	3.10E-01	1	15	323	21447	6.67%	1.51%	0.31%	0.07%	glycolysis to lactate	Pklr
			MP:0002864	abnormal ocular fundus morphology	2.07E-01	3.14E-01	2	15	1213	21447	13.33%	5.66%	0.16%	0.07%	glycolysis to lactate	Hkdc1, Pkm
			MP:0002675	asthenozoospermia	2.08E-01	3.14E-01	1	15	331	21447	6.67%	1.54%	0.30%	0.07%	glycolysis to lactate	Ldhc
			MP:0003960	increased lean body mass	2.08E-01	3.14E-01	1	15	331	21447	6.67%	1.54%	0.30%	0.07%	glycolysis to lactate	Pfkm
			MP:0001552	increased circulating triglyceride level	2.11E-01	3.17E-01	1	15	336	21447	6.67%	1.57%	0.30%	0.07%	glycolysis to lactate	Gck
			MP:0005280	abnormal fatty acids level	2.19E-01	3.28E-01	1	15	350	21447	6.67%	1.63%	0.29%	0.07%	glycolysis to lactate	Gck
			MP:0002079	increased circulating insulin level	2.19E-01	3.28E-01	1	15	351	21447	6.67%	1.64%	0.28%	0.07%	glycolysis to lactate	Gck
			MP:0001463	abnormal spatial learning	2.20E-01	3.28E-01	1	15	353	21447	6.67%	1.65%	0.28%	0.07%	glycolysis to lactate	Slc16a3
			MP:0030572	abnormal pilosebaceous unit morphology	2.22E-01	3.30E-01	1	15	356	21447	6.67%	1.66%	0.28%	0.07%	glycolysis to lactate	Pfkl
			MP:0005450	abnormal energy expenditure	2.23E-01	3.30E-01	1	15	357	21447	6.67%	1.66%	0.28%	0.07%	glycolysis to lactate	Slc16a1
			MP:0011088	neonatal lethality, incomplete penetrance	2.24E-01	3.31E-01	1	15	359	21447	6.67%	1.67%	0.28%	0.07%	glycolysis to lactate	Gck
			MP:0013330	abnormal male accessory sex gland morphology	2.25E-01	3.31E-01	1	15	362	21447	6.67%	1.69%	0.28%	0.07%	glycolysis to lactate	Pfkm
			MP:0008772	increased heart ventricle size	2.25E-01	3.31E-01	1	15	362	21447	6.67%	1.69%	0.28%	0.07%	glycolysis to lactate	Pfkm
			MP:0005292	improved glucose tolerance	2.26E-01	3.31E-01	1	15	363	21447	6.67%	1.69%	0.28%	0.07%	glycolysis to lactate	Slc16a1
			MP:0005448	abnormal energy homeostasis	2.28E-01	3.32E-01	1	15	367	21447	6.67%	1.71%	0.27%	0.07%	glycolysis to lactate	Slc16a1
			MP:0011117	abnormal susceptibility to weight gain	2.28E-01	3.32E-01	1	15	367	21447	6.67%	1.71%	0.27%	0.07%	glycolysis to lactate	Slc16a1
			MP:0013293	embryonic lethality prior to tooth bud stage	2.32E-01	3.36E-01	2	15	1308	21447	13.33%	6.10%	0.15%	0.07%	glycolysis to lactate	Hk2, Pkm
			MP:0000188	abnormal circulating glucose level	2.32E-01	3.36E-01	2	15	1309	21447	13.33%	6.10%	0.15%	0.07%	glycolysis to lactate	Gck, Pfkm
			MP:0000599	enlarged liver	2.34E-01	3.37E-01	1	15	377	21447	6.67%	1.76%	0.27%	0.07%	glycolysis to lactate	Ldha
			MP:0000219	increased neutrophil cell number	2.35E-01	3.38E-01	1	15	379	21447	6.67%	1.77%	0.26%	0.07%	glycolysis to lactate	Ldha
			MP:0005621	abnormal cell physiology	2.36E-01	3.38E-01	5	15	4840	21447	33.33%	22.57%	0.10%	0.07%	glycolysis to lactate	Gck, Hk2, Hkdc1, Ldhc, Pfkm
			MP:0002674	abnormal sperm motility	2.36E-01	3.38E-01	1	15	382	21447	6.67%	1.78%	0.26%	0.07%	glycolysis to lactate	Ldhc
			MP:0001924	infertility	2.37E-01	3.38E-01	2	15	1326	21447	13.33%	6.18%	0.15%	0.07%	glycolysis to lactate	Hk1, Ldhc
			MP:0001922	reduced male fertility	2.40E-01	3.42E-01	1	15	389	21447	6.67%	1.81%	0.26%	0.07%	glycolysis to lactate	Ldhc
			MP:0010508	abnormal heart electrocardiography waveform feature	2.41E-01	3.42E-01	1	15	390	21447	6.67%	1.82%	0.26%	0.07%	glycolysis to lactate	Gck
			MP:0000274	enlarged heart	2.43E-01	3.44E-01	2	15	1350	21447	13.33%	6.29%	0.15%	0.07%	glycolysis to lactate	Ldha, Pfkm
			MP:0013560	abnormal endocrine gland morphology	2.44E-01	3.45E-01	2	15	1355	21447	13.33%	6.32%	0.15%	0.07%	glycolysis to lactate	Gck, Hk3
			MP:0005379	endocrine/exocrine gland phenotype	2.47E-01	3.48E-01	4	15	3671	21447	26.67%	17.12%	0.11%	0.07%	glycolysis to lactate	Gck, Hk3, Ldhc, Pfkm
			MP:0002272	abnormal nervous system electrophysiology	2.53E-01	3.55E-01	1	15	412	21447	6.67%	1.92%	0.24%	0.07%	glycolysis to lactate	Slc16a3
			MP:0020186	abnormal susceptibility to bacterial infection	2.53E-01	3.55E-01	1	15	413	21447	6.67%	1.93%	0.24%	0.07%	glycolysis to lactate	Pklr
			MP:0002082	postnatal lethality	2.54E-01	3.55E-01	2	15	1392	21447	13.33%	6.49%	0.14%	0.07%	glycolysis to lactate	Gck, Ldha
			MP:0000260	abnormal angiogenesis	2.56E-01	3.57E-01	1	15	419	21447	6.67%	1.95%	0.24%	0.07%	glycolysis to lactate	Pfkm
			MP:0003137	abnormal impulse conducting system conduction	2.58E-01	3.58E-01	1	15	423	21447	6.67%	1.97%	0.24%	0.07%	glycolysis to lactate	Gck
			MP:0004543	abnormal sperm physiology	2.59E-01	3.58E-01	1	15	424	21447	6.67%	1.98%	0.24%	0.07%	glycolysis to lactate	Ldhc

			MP:0002970	abnormal white adipose tissue morphology	2.59E-01	3.58E-01	1	15	424	21447	6.67%	1.98%	0.24%	0.07%	glycolysis to lactate	Slc16a1
			MP:0001625	cardiac hypertrophy	2.60E-01	3.59E-01	1	15	427	21447	6.67%	1.99%	0.23%	0.07%	glycolysis to lactate	Pfkm
			MP:0005179	decreased circulating cholesterol level	2.61E-01	3.59E-01	1	15	428	21447	6.67%	2.00%	0.23%	0.07%	glycolysis to lactate	Ldha
			MP:0005195	abnormal posterior eye segment morphology	2.69E-01	3.69E-01	2	15	1447	21447	13.33%	6.75%	0.14%	0.07%	glycolysis to lactate	Hkdc1, Pkm
			MP:0013277	abnormal fasting circulating glucose level	2.73E-01	3.73E-01	1	15	450	21447	6.67%	2.10%	0.22%	0.07%	glycolysis to lactate	Gck
			MP:0001926	female infertility	2.75E-01	3.75E-01	1	15	455	21447	6.67%	2.12%	0.22%	0.07%	glycolysis to lactate	Hk1
			MP:0003983	decreased cholesterol level	2.77E-01	3.75E-01	1	15	458	21447	6.67%	2.14%	0.22%	0.07%	glycolysis to lactate	Ldha
			MP:0012225	decreased sterol level	2.77E-01	3.75E-01	1	15	458	21447	6.67%	2.14%	0.22%	0.07%	glycolysis to lactate	Ldha
			MP:0002396	abnormal hematopoietic system morphology/development	2.77E-01	3.75E-01	5	15	5119	21447	33.33%	23.87%	0.10%	0.07%	glycolysis to lactate	Gck, Hk1, Ldha, Pfkm, Pklr
			MP:0000266	abnormal heart morphology	2.78E-01	3.76E-01	3	15	2639	21447	20.00%	12.30%	0.11%	0.07%	glycolysis to lactate	Gck, Ldha, Pfkm
			MP:0005317	increased triglyceride level	2.79E-01	3.76E-01	1	15	462	21447	6.67%	2.15%	0.22%	0.07%	glycolysis to lactate	Gck
			MP:0001944	abnormal pancreas morphology	2.88E-01	3.88E-01	1	15	481	21447	6.67%	2.24%	0.21%	0.07%	glycolysis to lactate	Gck
			MP:0001589	abnormal mean corpuscular hemoglobin	2.90E-01	3.89E-01	1	15	484	21447	6.67%	2.26%	0.21%	0.07%	glycolysis to lactate	Hk1
			MP:0002968	increased circulating alkaline phosphatase level	2.94E-01	3.93E-01	1	15	492	21447	6.67%	2.29%	0.20%	0.07%	glycolysis to lactate	Gck
			MP:0000702	enlarged lymph nodes	3.01E-01	4.01E-01	1	15	506	21447	6.67%	2.36%	0.20%	0.07%	glycolysis to lactate	Hk3
			MP:0000350	abnormal cell proliferation	3.03E-01	4.03E-01	2	15	1577	21447	13.33%	7.35%	0.13%	0.07%	glycolysis to lactate	Gck, Hk2
			MP:0009850	embryonic lethality between implantation and placentation	3.05E-01	4.04E-01	1	15	513	21447	6.67%	2.39%	0.19%	0.07%	glycolysis to lactate	Hk2
			MP:0001304	cataract	3.07E-01	4.06E-01	1	15	517	21447	6.67%	2.41%	0.19%	0.07%	glycolysis to lactate	Hk3
			MP:0005378	growth/size/body region phenotype	3.08E-01	4.06E-01	7	15	7992	21447	46.67%	37.26%	0.09%	0.07%	glycolysis to lactate	Gck, Hk1, Hk2, Ldha, Pfkm, Pklr, Slc16a1
			MP:0012440	abnormal neutrophil cell number	3.12E-01	4.11E-01	1	15	528	21447	6.67%	2.46%	0.19%	0.07%	glycolysis to lactate	Ldha
			MP:0000849	abnormal cerebellum morphology	3.17E-01	4.16E-01	1	15	538	21447	6.67%	2.51%	0.19%	0.07%	glycolysis to lactate	Hk2
			MP:0000184	abnormal circulating HDL cholesterol level	3.17E-01	4.16E-01	1	15	539	21447	6.67%	2.51%	0.19%	0.07%	glycolysis to lactate	Ldha
			MP:0005449	abnormal food intake	3.19E-01	4.17E-01	1	15	542	21447	6.67%	2.53%	0.18%	0.07%	glycolysis to lactate	Slc16a1
			MP:0005065	abnormal neutrophil morphology	3.21E-01	4.18E-01	1	15	546	21447	6.67%	2.55%	0.18%	0.07%	glycolysis to lactate	Ldha
			MP:0005406	abnormal heart size	3.24E-01	4.21E-01	2	15	1653	21447	13.33%	7.71%	0.12%	0.07%	glycolysis to lactate	Ldha, Pfkm
			MP:0000351	increased cell proliferation	3.25E-01	4.22E-01	1	15	555	21447	6.67%	2.59%	0.18%	0.07%	glycolysis to lactate	Gck
			MP:0003091	abnormal cell migration	3.30E-01	4.27E-01	1	15	565	21447	6.67%	2.63%	0.18%	0.07%	glycolysis to lactate	Hk2
			MP:0003921	abnormal heart left ventricle morphology	3.33E-01	4.29E-01	1	15	571	21447	6.67%	2.66%	0.18%	0.07%	glycolysis to lactate	Pfkm
			MP:0000701	abnormal lymph node size	3.34E-01	4.29E-01	1	15	573	21447	6.67%	2.67%	0.17%	0.07%	glycolysis to lactate	Hk3
			MP:0002092	abnormal eye morphology	3.34E-01	4.29E-01	3	15	2916	21447	20.00%	13.60%	0.10%	0.07%	glycolysis to lactate	Hk3, Hkdc1, Pkm
			MP:0000847	abnormal metencephalon morphology	3.35E-01	4.29E-01	1	15	576	21447	6.67%	2.69%	0.17%	0.07%	glycolysis to lactate	Hk2
			MP:0000259	abnormal vascular development	3.36E-01	4.29E-01	1	15	577	21447	6.67%	2.69%	0.17%	0.07%	glycolysis to lactate	Pfkm
			MP:0002406	increased susceptibility to infection	3.40E-01	4.33E-01	1	15	586	21447	6.67%	2.73%	0.17%	0.07%	glycolysis to lactate	Pklr
			MP:0005384	cellular phenotype	3.40E-01	4.33E-01	5	15	5535	21447	33.33%	25.81%	0.09%	0.07%	glycolysis to lactate	Gck, Hk2, Hkdc1, Ldhc, Pfkm
			MP:0009643	abnormal urine homeostasis	3.42E-01	4.34E-01	1	15	590	21447	6.67%	2.75%	0.17%	0.07%	glycolysis to lactate	Gck
			MP:0000322	increased granulocyte number	3.49E-01	4.42E-01	1	15	605	21447	6.67%	2.82%	0.17%	0.07%	glycolysis to lactate	Ldha
			MP:0010330	abnormal circulating lipoprotein level	3.59E-01	4.51E-01	1	15	626	21447	6.67%	2.92%	0.16%	0.07%	glycolysis to lactate	Ldha
			MP:0010329	abnormal lipoprotein level	3.59E-01	4.51E-01	1	15	626	21447	6.67%	2.92%	0.16%	0.07%	glycolysis to lactate	Ldha
			MP:0001260	increased body weight	3.60E-01	4.51E-01	1	15	628	21447	6.67%	2.93%	0.16%	0.07%	glycolysis to lactate	Gck
			MP:0012323	increased total tissue mass	3.60E-01	4.51E-01	1	15	628	21447	6.67%	2.93%	0.16%	0.07%	glycolysis to lactate	Gck
			MP:0000841	abnormal hindbrain morphology	3.69E-01	4.61E-01	1	15	649	21447	6.67%	3.03%	0.15%	0.07%	glycolysis to lactate	Hk2
			MP:0009931	abnormal skin appearance	3.69E-01	4.61E-01	1	15	649	21447	6.67%	3.03%	0.15%	0.07%	glycolysis to lactate	Hk1
			MP:0008415	abnormal neurite morphology	3.71E-01	4.62E-01	1	15	653	21447	6.67%	3.04%	0.15%	0.07%	glycolysis to lactate	Slc16a3
			MP:0005397	hematopoietic system phenotype	3.73E-01	4.63E-01	5	15	5741	21447	33.33%	26.77%	0.09%	0.07%	glycolysis to lactate	Gck, Hk1, Ldha, Pfkm, Pklr
			MP:0000759	abnormal skeletal muscle morphology	3.75E-01	4.64E-01	1	15	661	21447	6.67%	3.08%	0.15%	0.07%	glycolysis to lactate	Pfkm
			MP:0001259	abnormal body weight	3.77E-01	4.66E-01	3	15	3125	21447	20.00%	14.57%	0.10%	0.07%	glycolysis to lactate	Gck, Hk2, Slc16a1
			MP:0001264	increased body size	3.85E-01	4.73E-01	1	15	683	21447	6.67%	3.18%	0.15%	0.07%	glycolysis to lactate	Gck
			MP:0003959	abnormal lean body mass	3.85E-01	4.73E-01	1	15	684	21447	6.67%	3.19%	0.15%	0.07%	glycolysis to lactate	Pfkm
			MP:0003984	embryonic growth retardation	3.91E-01	4.79E-01	1	15	697	21447	6.67%	3.25%	0.14%	0.07%	glycolysis to lactate	Hk2
			MP:0001431	abnormal eating behavior	3.93E-01	4.81E-01	1	15	702	21447	6.67%	3.27%	0.14%	0.07%	glycolysis to lactate	Slc16a1

			MP:0002339	abnormal lymph node morphology	3.98E-01	4.86E-01	1	15	714	21447	6.67%	3.33%	0.14%	0.07%	glycolysis to lactate	Hk3
			MP:0005560	decreased circulating glucose level	4.01E-01	4.87E-01	1	15	719	21447	6.67%	3.35%	0.14%	0.07%	glycolysis to lactate	Gck
			MP:0012382	abnormal blood cell physiology	4.01E-01	4.87E-01	2	15	1946	21447	13.33%	9.07%	0.10%	0.07%	glycolysis to lactate	Pfkm, Pklr
			MP:0004087	abnormal muscle fiber morphology	4.03E-01	4.89E-01	1	15	725	21447	6.67%	3.38%	0.14%	0.07%	glycolysis to lactate	Pfkm
			MP:0011098	embryonic lethality during organogenesis, complete penetrance	4.07E-01	4.92E-01	1	15	734	21447	6.67%	3.42%	0.14%	0.07%	glycolysis to lactate	Ldha
			MP:0004811	abnormal neuron physiology	4.16E-01	5.02E-01	1	15	756	21447	6.67%	3.52%	0.13%	0.07%	glycolysis to lactate	Hk2
			MP:0001303	abnormal lens morphology	4.18E-01	5.03E-01	1	15	759	21447	6.67%	3.54%	0.13%	0.07%	glycolysis to lactate	Hk3
			MP:0002163	abnormal gland morphology	4.19E-01	5.04E-01	3	15	3333	21447	20.00%	15.54%	0.09%	0.07%	glycolysis to lactate	Gck, Hk3, Pfkm
			MP:0005451	abnormal body composition	4.27E-01	5.12E-01	3	15	3373	21447	20.00%	15.73%	0.09%	0.07%	glycolysis to lactate	Gck, Hk2, Pfkm
			MP:0002161	abnormal fertility/fecundity	4.33E-01	5.17E-01	2	15	2073	21447	13.33%	9.67%	0.10%	0.07%	glycolysis to lactate	Hk1, Ldhc
			MP:0001657	abnormal induced morbidity/mortality	4.34E-01	5.17E-01	1	15	798	21447	6.67%	3.72%	0.13%	0.07%	glycolysis to lactate	Pklr
			MP:0001545	abnormal hematopoietic system physiology	4.36E-01	5.17E-01	2	15	2083	21447	13.33%	9.71%	0.10%	0.07%	glycolysis to lactate	Pfkm, Pklr
			MP:0001663	abnormal digestive system physiology	4.38E-01	5.17E-01	1	15	807	21447	6.67%	3.76%	0.12%	0.07%	glycolysis to lactate	Slc16a1
			MP:0005387	immune system phenotype	4.38E-01	5.17E-01	5	15	6144	21447	33.33%	28.65%	0.08%	0.07%	glycolysis to lactate	Hk1, Hk3, Ldha, Pfkm, Pklr
			MP:0001793	abnormal susceptibility to infection	4.38E-01	5.17E-01	1	15	809	21447	6.67%	3.77%	0.12%	0.07%	glycolysis to lactate	Pklr
			MP:0010865	prenatal growth retardation	4.39E-01	5.17E-01	1	15	810	21447	6.67%	3.78%	0.12%	0.07%	glycolysis to lactate	Hk2
			MP:0001175	abnormal lung morphology	4.39E-01	5.17E-01	1	15	811	21447	6.67%	3.78%	0.12%	0.07%	glycolysis to lactate	Ldha
			MP:0020201	abnormal granulocyte number	4.42E-01	5.18E-01	1	15	817	21447	6.67%	3.81%	0.12%	0.07%	glycolysis to lactate	Ldha
			MP:0010630	abnormal cardiac muscle tissue morphology	4.44E-01	5.20E-01	1	15	822	21447	6.67%	3.83%	0.12%	0.07%	glycolysis to lactate	Pfkm
			MP:0002127	abnormal cardiovascular system morphology	4.46E-01	5.21E-01	3	15	3466	21447	20.00%	16.16%	0.09%	0.07%	glycolysis to lactate	Gck, Ldha, Pfkm
			MP:0005025	abnormal response to infection	4.55E-01	5.31E-01	1	15	851	21447	6.67%	3.97%	0.12%	0.07%	glycolysis to lactate	Pklr
			MP:0002441	abnormal granulocyte morphology	4.57E-01	5.32E-01	1	15	856	21447	6.67%	3.99%	0.12%	0.07%	glycolysis to lactate	Ldha
			MP:0020000	abnormal response to antigen	4.60E-01	5.33E-01	1	15	863	21447	6.67%	4.02%	0.12%	0.07%	glycolysis to lactate	Pklr
			MP:0002925	abnormal cardiovascular development	4.60E-01	5.33E-01	1	15	864	21447	6.67%	4.03%	0.12%	0.07%	glycolysis to lactate	Pfkm
			MP:0005367	renal/urinary system phenotype	4.65E-01	5.37E-01	2	15	2201	21447	13.33%	10.26%	0.09%	0.07%	glycolysis to lactate	Gck, Hk1
			MP:0002069	abnormal consumption behavior	4.66E-01	5.37E-01	1	15	877	21447	6.67%	4.09%	0.11%	0.07%	glycolysis to lactate	Slc16a1
			MP:0005502	abnormal renal/urinary system physiology	4.81E-01	5.53E-01	1	15	918	21447	6.67%	4.28%	0.11%	0.07%	glycolysis to lactate	Gck
			MP:0000913	abnormal brain development	4.82E-01	5.53E-01	1	15	919	21447	6.67%	4.28%	0.11%	0.07%	glycolysis to lactate	Hk2
			MP:0004849	abnormal testis size	4.90E-01	5.61E-01	1	15	940	21447	6.67%	4.38%	0.11%	0.07%	glycolysis to lactate	Pfkm
			MP:0003699	abnormal female reproductive system physiology	4.92E-01	5.62E-01	1	15	946	21447	6.67%	4.41%	0.11%	0.07%	glycolysis to lactate	Hk1
			MP:0002058	neonatal lethality	4.95E-01	5.65E-01	1	15	955	21447	6.67%	4.45%	0.10%	0.07%	glycolysis to lactate	Gck
			MP:0001919	abnormal reproductive system physiology	5.00E-01	5.69E-01	2	15	2348	21447	13.33%	10.95%	0.09%	0.07%	glycolysis to lactate	Hk1, Ldhc
			MP:0001921	reduced fertility	5.01E-01	5.69E-01	1	15	972	21447	6.67%	4.53%	0.10%	0.07%	glycolysis to lactate	Ldhc
			MP:0005369	muscle phenotype	5.02E-01	5.69E-01	2	15	2355	21447	13.33%	10.98%	0.08%	0.07%	glycolysis to lactate	Pfkm, Slc16a3
			MP:0001925	male infertility	5.16E-01	5.82E-01	1	15	1013	21447	6.67%	4.72%	0.10%	0.07%	glycolysis to lactate	Ldhc
			MP:0001262	decreased body weight	5.17E-01	5.82E-01	2	15	2418	21447	13.33%	11.27%	0.08%	0.07%	glycolysis to lactate	Gck, Hk2
			MP:0012322	decreased total tissue mass	5.17E-01	5.82E-01	2	15	2419	21447	13.33%	11.28%	0.08%	0.07%	glycolysis to lactate	Gck, Hk2
			MP:0005389	reproductive system phenotype	5.32E-01	5.97E-01	3	15	3905	21447	20.00%	18.21%	0.08%	0.07%	glycolysis to lactate	Hk1, Ldhc, Pfkm
			MP:0001732	postnatal growth retardation	5.35E-01	6.00E-01	1	15	1068	21447	6.67%	4.98%	0.09%	0.07%	glycolysis to lactate	Gck
			MP:0010832	lethality during fetal growth through weaning	5.44E-01	6.09E-01	2	15	2537	21447	13.33%	11.83%	0.08%	0.07%	glycolysis to lactate	Gck, Ldha
			MP:0001146	abnormal testis morphology	5.49E-01	6.13E-01	1	15	1109	21447	6.67%	5.17%	0.09%	0.07%	glycolysis to lactate	Pfkm
			MP:0013558	abnormal exocrine gland morphology	5.56E-01	6.19E-01	1	15	1130	21447	6.67%	5.27%	0.09%	0.07%	glycolysis to lactate	Pfkm
			MP:0005294	abnormal heart ventricle morphology	5.59E-01	6.21E-01	1	15	1138	21447	6.67%	5.31%	0.09%	0.07%	glycolysis to lactate	Pfkm
			MP:0001731	abnormal postnatal growth	5.61E-01	6.22E-01	1	15	1144	21447	6.67%	5.33%	0.09%	0.07%	glycolysis to lactate	Gck
			MP:0008251	abnormal phagocyte morphology	5.71E-01	6.32E-01	1	15	1176	21447	6.67%	5.48%	0.09%	0.07%	glycolysis to lactate	Ldha
			MP:0005193	abnormal anterior eye segment morphology	5.77E-01	6.37E-01	1	15	1196	21447	6.67%	5.58%	0.08%	0.07%	glycolysis to lactate	Hk3
			MP:0002088	abnormal embryonic growth/weight/body size	5.83E-01	6.43E-01	1	15	1216	21447	6.67%	5.67%	0.08%	0.07%	glycolysis to lactate	Hk2
			MP:0003633	abnormal nervous system physiology	5.88E-01	6.46E-01	2	15	2737	21447	13.33%	12.76%	0.07%	0.07%	glycolysis to lactate	Hk2, Slc16a3

			MP:0002132	abnormal respiratory system morphology	5.94E-01	6.51E-01	1	15	1250	21447	6.67%	5.83%	0.08%	0.07%	glycolysis to lactate	Ldha
			MP:0001672	abnormal embryo development	5.97E-01	6.53E-01	1	15	1259	21447	6.67%	5.87%	0.08%	0.07%	glycolysis to lactate	Hk2
			MP:0002752	abnormal somatic nervous system morphology	6.04E-01	6.60E-01	1	15	1285	21447	6.67%	5.99%	0.08%	0.07%	glycolysis to lactate	Slc16a3
			MP:0002063	abnormal learning/memory/conditioning	6.08E-01	6.63E-01	1	15	1299	21447	6.67%	6.06%	0.08%	0.07%	glycolysis to lactate	Slc16a3
			MP:0014114	abnormal cognition	6.12E-01	6.66E-01	1	15	1311	21447	6.67%	6.11%	0.08%	0.07%	glycolysis to lactate	Slc16a3
			MP:0013325	abnormal male reproductive gland morphology	6.21E-01	6.74E-01	1	15	1342	21447	6.67%	6.26%	0.07%	0.07%	glycolysis to lactate	Pfkm
			MP:0012321	abnormal total tissue mass	6.23E-01	6.75E-01	2	15	2909	21447	13.33%	13.56%	0.07%	0.07%	glycolysis to lactate	Gck, Hk2
			MP:0002081	perinatal lethality	6.32E-01	6.83E-01	1	15	1382	21447	6.67%	6.44%	0.07%	0.07%	glycolysis to lactate	Gck
			MP:0008250	abnormal myeloid leukocyte morphology	6.40E-01	6.90E-01	1	15	1411	21447	6.67%	6.58%	0.07%	0.07%	glycolysis to lactate	Ldha
			MP:0002106	abnormal muscle physiology	6.42E-01	6.91E-01	1	15	1420	21447	6.67%	6.62%	0.07%	0.07%	glycolysis to lactate	Pfkm
			MP:0004196	abnormal prenatal growth/weight/body size	6.46E-01	6.91E-01	1	15	1435	21447	6.67%	6.69%	0.07%	0.07%	glycolysis to lactate	Hk2
			MP:0001961	abnormal reflex	6.48E-01	6.91E-01	1	15	1441	21447	6.67%	6.72%	0.07%	0.07%	glycolysis to lactate	Ldhb
			MP:0003698	abnormal male reproductive system physiology	6.48E-01	6.91E-01	1	15	1443	21447	6.67%	6.73%	0.07%	0.07%	glycolysis to lactate	Ldhc
			MP:0005385	cardiovascular system phenotype	6.49E-01	6.91E-01	3	15	4565	21447	20.00%	21.29%	0.07%	0.07%	glycolysis to lactate	Gck, Ldha, Pfkm
			MP:0009205	abnormal internal male genitalia morphology	6.49E-01	6.91E-01	1	15	1445	21447	6.67%	6.74%	0.07%	0.07%	glycolysis to lactate	Pfkm
			MP:0003861	abnormal nervous system development	6.50E-01	6.91E-01	1	15	1450	21447	6.67%	6.76%	0.07%	0.07%	glycolysis to lactate	Hk2
			MP:0009198	abnormal male genitalia morphology	6.56E-01	6.94E-01	1	15	1471	21447	6.67%	6.86%	0.07%	0.07%	glycolysis to lactate	Pfkm
			MP:0001145	abnormal male reproductive system morphology	6.56E-01	6.94E-01	1	15	1471	21447	6.67%	6.86%	0.07%	0.07%	glycolysis to lactate	Pfkm
			MP:0002135	abnormal kidney morphology	6.76E-01	7.15E-01	1	15	1553	21447	6.67%	7.24%	0.06%	0.07%	glycolysis to lactate	Hk1
			MP:0000653	abnormal sex gland morphology	6.85E-01	7.23E-01	1	15	1590	21447	6.67%	7.41%	0.06%	0.07%	glycolysis to lactate	Pfkm
			MP:0002067	abnormal sensory capabilities/reflexes/nociception	7.03E-01	7.40E-01	1	15	1665	21447	6.67%	7.76%	0.06%	0.07%	glycolysis to lactate	Ldhb
			MP:0001614	abnormal blood vessel morphology	7.04E-01	7.40E-01	1	15	1672	21447	6.67%	7.80%	0.06%	0.07%	glycolysis to lactate	Pfkm
			MP:0002108	abnormal muscle morphology	7.06E-01	7.40E-01	1	15	1679	21447	6.67%	7.83%	0.06%	0.07%	glycolysis to lactate	Pfkm
			MP:0004924	abnormal behavior	7.08E-01	7.41E-01	4	15	6473	21447	26.67%	30.18%	0.06%	0.07%	glycolysis to lactate	Ldhb, Pfkm, Slc16a1, Slc16a3
			MP:0000218	increased leukocyte cell number	7.12E-01	7.44E-01	1	15	1708	21447	6.67%	7.96%	0.06%	0.07%	glycolysis to lactate	Ldha
			MP:0000516	abnormal renal/urinary system morphology	7.17E-01	7.47E-01	1	15	1730	21447	6.67%	8.07%	0.06%	0.07%	glycolysis to lactate	Hk1
			MP:0003492	abnormal involuntary movement	7.22E-01	7.51E-01	1	15	1754	21447	6.67%	8.18%	0.06%	0.07%	glycolysis to lactate	Ldhb
			MP:0002083	premature death	7.30E-01	7.58E-01	1	15	1793	21447	6.67%	8.36%	0.06%	0.07%	glycolysis to lactate	Pfkm
			MP:0000358	abnormal cell morphology	7.40E-01	7.66E-01	1	15	1840	21447	6.67%	8.58%	0.05%	0.07%	glycolysis to lactate	Gck
			MP:0005386	behavior/neurological phenotype	7.45E-01	7.70E-01	4	15	6756	21447	26.67%	31.50%	0.06%	0.07%	glycolysis to lactate	Ldhb, Pfkm, Slc16a1, Slc16a3
			MP:0005388	respiratory system phenotype	7.52E-01	7.76E-01	1	15	1902	21447	6.67%	8.87%	0.05%	0.07%	glycolysis to lactate	Ldha
			MP:0010678	abnormal skin adnexa morphology	7.53E-01	7.76E-01	1	15	1911	21447	6.67%	8.91%	0.05%	0.07%	glycolysis to lactate	Pfkl
			MP:0005381	digestive/alimentary phenotype	7.58E-01	7.79E-01	1	15	1936	21447	6.67%	9.03%	0.05%	0.07%	glycolysis to lactate	Slc16a1
			MP:0000313	abnormal cell death	7.77E-01	7.97E-01	1	15	2040	21447	6.67%	9.51%	0.05%	0.07%	glycolysis to lactate	Pfkm
			MP:0002882	abnormal neuron morphology	7.93E-01	8.11E-01	1	15	2134	21447	6.67%	9.95%	0.05%	0.07%	glycolysis to lactate	Slc16a3
			MP:0003632	abnormal nervous system morphology	7.96E-01	8.13E-01	2	15	3970	21447	13.33%	18.51%	0.05%	0.07%	glycolysis to lactate	Hk2, Slc16a3
			MP:0002152	abnormal brain morphology	8.24E-01	8.38E-01	1	15	2342	21447	6.67%	10.92%	0.04%	0.07%	glycolysis to lactate	Hk2
			MP:0005380	embryo phenotype	8.24E-01	8.38E-01	1	15	2343	21447	6.67%	10.92%	0.04%	0.07%	glycolysis to lactate	Hk2
			MP:0002160	abnormal reproductive system morphology	8.37E-01	8.50E-01	1	15	2441	21447	6.67%	11.38%	0.04%	0.07%	glycolysis to lactate	Pfkm
			MP:0000217	abnormal leukocyte cell number	8.43E-01	8.54E-01	1	15	2489	21447	6.67%	11.61%	0.04%	0.07%	glycolysis to lactate	Ldha
			MP:0001544	abnormal cardiovascular system physiology	8.45E-01	8.55E-01	1	15	2508	21447	6.67%	11.69%	0.04%	0.07%	glycolysis to lactate	Gck
			MP:0008246	abnormal leukocyte morphology	8.83E-01	8.91E-01	1	15	2855	21447	6.67%	13.31%	0.04%	0.07%	glycolysis to lactate	Ldha
			MP:0000716	abnormal immune system cell morphology	8.85E-01	8.92E-01	1	15	2882	21447	6.67%	13.44%	0.03%	0.07%	glycolysis to lactate	Ldha
			MP:0003631	nervous system phenotype	9.15E-01	9.20E-01	2	15	5280	21447	13.33%	24.62%	0.04%	0.07%	glycolysis to lactate	Hk2, Slc16a3
			MP:0001790	abnormal immune system physiology	9.41E-01	9.44E-01	1	15	3678	21447	6.67%	17.15%	0.03%	0.07%	glycolysis to lactate	Pklr
			MP:0002066	abnormal motor capabilities/coordination/movement	9.77E-01	9.79E-01	1	15	4764	21447	6.67%	22.21%	0.02%	0.07%	glycolysis to lactate	Ldhb

			MP:0000001	mammalian phenotype	1.00E+00	1.00E+00	15	15	21447	21447	100.00%	100.00%	0.07%	0.07%	glycolysis to lactate	Gck, Hk1, Hk2, Hk3, Hkdc1, Ldha, Ldhb, Ldhc, Pfkl, Pfkm, Pklr, Pkm, Slc16a1, Slc16a3, Slc16a8
