# Supplementary material for: Identification of Biochemical Pathways Responsible for Distinct Phenotypes Using Gene Ontology Causal Activity Models
Source: bioRxiv. 2023 May 25:2023.05.22.541760. Preprint. [Version 2] doi: 10.1101/2023.05.22.541760 (PMC10245817; doi:10.1101/2023.05.22.541760)
Supplement: Supplementary file 4 [file media-4.pdf]

Genes Used			TermID	Term	P	Q	k	n	K	N	k/n	K/N	k/K	n/N	Qset	Symbols
**glycolysis**	**pyruvate->acetylCoA**		MP:0013403	abnormal circulating lactate level	3.55E-06	2.00E-03	3	18	36	21319	16.67%	0.17%	8.33%	0.08%	Glycolysis to A-CoA	Mpc1, Mpc2, Pfkm
Gck (MGI:1270854)	*Vdac1* (MGI:106919)		MP:0030970	ketosis	7.50E-06	2.12E-03	3	18	46	21319	16.67%	0.22%	6.52%	0.08%	Glycolysis to A-CoA	Gck, Mpc1, Mpc2
Hk1 (MGI:96103)	*Mpc1* (MGI:1915240)		MP:0005291	abnormal glucose tolerance	5.97E-05	7.53E-03	6	18	882	21319	33.33%	4.14%	0.68%	0.08%	Glycolysis to A-CoA	Gck, Hkdc1, Mpc1, Mpc2, Pfkm, Vdac1
Hk2 (MGI:1315197)	*Mpc2* (MGI:1917706)		MP:0010770	preweaning lethality	6.60E-05	7.53E-03	13	18	5648	21319	72.22%	26.49%	0.23%	0.08%	Glycolysis to A-CoA	Dlat, Dld, Gck, Hk2, Hkdc1, Mpc2, Pdha1, Pdhb, Pdhx, Pfkl, Pfkm, Pkm, Vdac1
Hk3 (MGI:2670962)	*Dld* (MGI:107450)		MP:0002640	reticulocytosis	7.31E-05	7.53E-03	3	18	98	21319	16.67%	0.46%	3.06%	0.08%	Glycolysis to A-CoA	Hk1, Pfkm, Pklr
Hkdc1 (MGI:2384910)	*Dlat* (MGI:2385311)		MP:0005293	impaired glucose tolerance	7.99E-05	7.53E-03	5	18	561	21319	27.78%	2.63%	0.89%	0.08%	Glycolysis to A-CoA	Gck, Hkdc1, Mpc2, Pfkm, Vdac1
Pfkm (MGI:97548)	*Pdha1* (MGI:97532)		MP:0011913	abnormal reticulocyte cell number	1.09E-04	8.17E-03	3	18	112	21319	16.67%	0.53%	2.68%	0.08%	Glycolysis to A-CoA	Hk1, Pfkm, Pklr
Pfkp (MGI:1891833)	*Pdha1* (MGI:97532)		MP:0031085	increased erythrocyte osmotic fragility	1.27E-04	8.17E-03	2	18	20	21319	11.11%	0.09%	10.00%	0.08%	Glycolysis to A-CoA	Pfkm, Pklr
Pfkl (MGI:97547)	*Pdhb* (MGI:1915513)		MP:0002424	abnormal reticulocyte morphology	1.30E-04	8.17E-03	3	18	119	21319	16.67%	0.56%	2.52%	0.08%	Glycolysis to A-CoA	Hk1, Pfkm, Pklr
Pkm (MGI:97591)	*Pdhx* (MGI:1351627)		MP:0013405	increased circulating lactate level	1.84E-04	1.04E-02	2	18	24	21319	11.11%	0.11%	8.33%	0.08%	Glycolysis to A-CoA	Mpc1, Mpc2
Pklr (MGI:97604)			MP:0008762	embryonic lethality	2.56E-04	1.25E-02	8	18	2256	21319	44.44%	10.58%	0.35%	0.08%	Glycolysis to A-CoA	Dld, Gck, Hk2, Mpc2, Pdha1, Pdhb, Pkm, Vdac1
			MP:0010768	mortality/aging	2.65E-04	1.25E-02	14	18	7471	21319	77.78%	35.04%	0.19%	0.08%	Glycolysis to A-CoA	Dlat, Dld, Gck, Hk2, Hkdc1, Mpc2, Pdha1, Pdhb, Pdhx, Pfkl, Pfkm, Pklr, Pkm, Vdac1
	not annotated to a phenotype		MP:0003059	decreased insulin secretion	3.06E-04	1.33E-02	3	18	159	21319	16.67%	0.75%	1.89%	0.08%	Glycolysis to A-CoA	Gck, Mpc2, Pfkm
			MP:0003657	abnormal erythrocyte osmotic lysis	3.50E-04	1.41E-02	2	18	33	21319	11.11%	0.15%	6.06%	0.08%	Glycolysis to A-CoA	Pfkm, Pklr
			MP:0010769	abnormal survival	4.11E-04	1.55E-02	13	18	6649	21319	72.22%	31.19%	0.20%	0.08%	Glycolysis to A-CoA	Dlat, Dld, Gck, Hk2, Hkdc1, Mpc2, Pdha1, Pdhb, Pdhx, Pfkl, Pfkm, Pkm, Vdac1
			MP:0010163	hemolysis	4.65E-04	1.64E-02	2	18	38	21319	11.11%	0.18%	5.26%	0.08%	Glycolysis to A-CoA	Pfkm, Pklr
			MP:0005344	increased circulating bilirubin level	5.23E-04	1.69E-02	3	18	191	21319	16.67%	0.90%	1.57%	0.08%	Glycolysis to A-CoA	Hk1, Pfkm, Pklr
			MP:0001585	hemolytic anemia	5.68E-04	1.69E-02	2	18	42	21319	11.11%	0.20%	4.76%	0.08%	Glycolysis to A-CoA	Hk1, Pklr
			MP:0002575	increased circulating ketone body level	5.68E-04	1.69E-02	2	18	42	21319	11.11%	0.20%	4.76%	0.08%	Glycolysis to A-CoA	Mpc1, Mpc2
			MP:0001569	abnormal circulating bilirubin level	6.16E-04	1.74E-02	3	18	202	21319	16.67%	0.95%	1.49%	0.08%	Glycolysis to A-CoA	Hk1, Pfkm, Pklr
			MP:0003564	abnormal insulin secretion	7.99E-04	1.99E-02	3	18	221	21319	16.67%	1.04%	1.36%	0.08%	Glycolysis to A-CoA	Gck, Mpc2, Pfkm
			MP:0014195	abnormal endocrine pancreas secretion	8.31E-04	1.99E-02	3	18	224	21319	16.67%	1.05%	1.34%	0.08%	Glycolysis to A-CoA	Gck, Mpc2, Pfkm
			MP:0008931	abnormal paraventricular thalamic nucleus morphology	8.44E-04	1.99E-02	1	18	1	21319	5.56%	0.00%	100.00%	0.08%	Glycolysis to A-CoA	Pdha1
			MP:0000874	irregular external granule cell layer thickness	8.44E-04	1.99E-02	1	18	1	21319	5.56%	0.00%	100.00%	0.08%	Glycolysis to A-CoA	Hk2
			MP:0002694	abnormal pancreas secretion	9.31E-04	2.04E-02	3	18	233	21319	16.67%	1.09%	1.29%	0.08%	Glycolysis to A-CoA	Gck, Mpc2, Pfkm
			MP:0002078	abnormal glucose homeostasis	9.38E-04	2.04E-02	7	18	2056	21319	38.89%	9.64%	0.34%	0.08%	Glycolysis to A-CoA	Gck, Hkdc1, Mpc1, Mpc2, Pdha1, Pfkm, Vdac1
			MP:0003562	abnormal pancreatic beta cell physiology	1.18E-03	2.47E-02	3	18	253	21319	16.67%	1.19%	1.19%	0.08%	Glycolysis to A-CoA	Gck, Mpc2, Pfkm
			MP:0010147	abnormal endocrine pancreas physiology	1.24E-03	2.49E-02	3	18	257	21319	16.67%	1.21%	1.17%	0.08%	Glycolysis to A-CoA	Gck, Mpc2, Pfkm
			MP:0003457	abnormal circulating ketone body level	1.32E-03	2.56E-02	2	18	64	21319	11.11%	0.30%	3.12%	0.08%	Glycolysis to A-CoA	Mpc1, Mpc2
			MP:0003656	abnormal erythrocyte physiology	1.36E-03	2.56E-02	2	18	65	21319	11.11%	0.30%	3.08%	0.08%	Glycolysis to A-CoA	Pfkm, Pklr
			MP:0005266	abnormal metabolism	1.62E-03	2.62E-02	5	18	1079	21319	27.78%	5.06%	0.46%	0.08%	Glycolysis to A-CoA	Gck, Mpc1, Pfkl, Pfkm, Pklr
			MP:0011604	abnormal glucokinase activity	1.69E-03	2.62E-02	1	18	2	21319	5.56%	0.01%	50.00%	0.08%	Glycolysis to A-CoA	Gck
			MP:0020552	abnormal stria medullaris morphology	1.69E-03	2.62E-02	1	18	2	21319	5.56%	0.01%	50.00%	0.08%	Glycolysis to A-CoA	Pdha1
			MP:0020553	abnormal stria medullaris size	1.69E-03	2.62E-02	1	18	2	21319	5.56%	0.01%	50.00%	0.08%	Glycolysis to A-CoA	Pdha1
			MP:0011606	decreased glucokinase activity	1.69E-03	2.62E-02	1	18	2	21319	5.56%	0.01%	50.00%	0.08%	Glycolysis to A-CoA	Gck
			MP:0020554	decreased stria medullaris size	1.69E-03	2.62E-02	1	18	2	21319	5.56%	0.01%	50.00%	0.08%	Glycolysis to A-CoA	Pdha1
			MP:0001560	abnormal circulating insulin level	1.72E-03	2.62E-02	4	18	636	21319	22.22%	2.98%	0.63%	0.08%	Glycolysis to A-CoA	Gck, Mpc2, Pdha1, Pfkm
			MP:0002080	prenatal lethality	1.76E-03	2.62E-02	8	18	2995	21319	44.44%	14.05%	0.27%	0.08%	Glycolysis to A-CoA	Dld, Gck, Hk2, Mpc2, Pdha1, Pdhb, Pkm, Vdac1
			MP:0003984	embryonic growth retardation	2.28E-03	3.27E-02	4	18	687	21319	22.22%	3.22%	0.58%	0.08%	Glycolysis to A-CoA	Dld, Hk2, Pdha1, Pdhb
			MP:0002727	decreased circulating insulin level	2.31E-03	3.27E-02	3	18	320	21319	16.67%	1.50%	0.94%	0.08%	Glycolysis to A-CoA	Gck, Mpc2, Pfkm
			MP:0000208	decreased hematocrit	2.40E-03	3.30E-02	3	18	324	21319	16.67%	1.52%	0.93%	0.08%	Glycolysis to A-CoA	Hk1, Pfkm, Pklr
			MP:0005584	abnormal enzyme/coenzyme activity	2.70E-03	3.63E-02	3	18	338	21319	16.67%	1.59%	0.89%	0.08%	Glycolysis to A-CoA	Gck, Pfkl, Pklr
			MP:0004079	abnormal putamen morphology	3.37E-03	4.39E-02	1	18	4	21319	5.56%	0.02%	25.00%	0.08%	Glycolysis to A-CoA	Pdha1
			MP:0000187	abnormal triglyceride level	3.45E-03	4.39E-02	4	18	770	21319	22.22%	3.61%	0.52%	0.08%	Glycolysis to A-CoA	Gck, Hkdc1, Mpc1, Mpc2
			MP:0011100	preweaning lethality, complete penetrance	3.50E-03	4.39E-02	6	18	1889	21319	33.33%	8.86%	0.32%	0.08%	Glycolysis to A-CoA	Dlat, Hkdc1, Pdhb, Pdhx, Pfkl, Pkm
			MP:0010865	prenatal growth retardation	3.95E-03	4.86E-02	4	18	800	21319	22.22%	3.75%	0.50%	0.08%	Glycolysis to A-CoA	Dld, Hk2, Pdha1, Pdhb
			MP:0013663	increased myeloid cell number	4.13E-03	4.96E-02	3	18	393	21319	16.67%	1.84%	0.76%	0.08%	Glycolysis to A-CoA	Hk1, Pfkm, Pklr
			MP:0009976	abnormal cerebellar peduncle morphology	4.21E-03	4.96E-02	1	18	5	21319	5.56%	0.02%	20.00%	0.08%	Glycolysis to A-CoA	Pdha1
			MP:0002693	abnormal pancreas physiology	4.62E-03	5.32E-02	3	18	409	21319	16.67%	1.92%	0.73%	0.08%	Glycolysis to A-CoA	Gck, Mpc2, Pfkm
			MP:0002814	hyperchromasia	5.06E-03	5.71E-02	1	18	6	21319	5.56%	0.03%	16.67%	0.08%	Glycolysis to A-CoA	Hk1
			MP:0002596	abnormal hematocrit	5.66E-03	6.27E-02	3	18	440	21319	16.67%	2.06%	0.68%	0.08%	Glycolysis to A-CoA	Hk1, Pfkm, Pklr
			MP:0005563	abnormal hemoglobin content	5.84E-03	6.29E-02	3	18	445	21319	16.67%	2.09%	0.67%	0.08%	Glycolysis to A-CoA	Gck, Hk1, Pklr

			MP:0006353	increased glycosylated hemoglobin level	5.90E-03	6.29E-02	1	18	7	21319	5.56%	0.03%	14.29%	0.08%	Glycolysis to A-CoA	Gck
			MP:0002625	heart left ventricle hypertrophy	6.29E-03	6.58E-02	2	18	142	21319	11.11%	0.67%	1.41%	0.08%	Glycolysis to A-CoA	Pdha1, Pfkm
			MP:0005317	increased triglyceride level	6.48E-03	6.65E-02	3	18	462	21319	16.67%	2.17%	0.65%	0.08%	Glycolysis to A-CoA	Gck, Hkdc1, Mpc1
			MP:0013561	abnormal endocrine gland physiology	6.71E-03	6.77E-02	3	18	468	21319	16.67%	2.20%	0.64%	0.08%	Glycolysis to A-CoA	Gck, Mpc2, Pfkm
			MP:0006351	abnormal glycosylated hemoglobin level	7.57E-03	7.38E-02	1	18	9	21319	5.56%	0.04%	11.11%	0.08%	Glycolysis to A-CoA	Gck
			MP:0005237	abnormal olfactory tract morphology	7.57E-03	7.38E-02	1	18	9	21319	5.56%	0.04%	11.11%	0.08%	Glycolysis to A-CoA	Pdha1
			MP:0000245	abnormal erythropoiesis	8.11E-03	7.54E-02	2	18	162	21319	11.11%	0.76%	1.23%	0.08%	Glycolysis to A-CoA	Hk1, Pklr
			MP:0008515	thin retina outer nuclear layer	8.30E-03	7.54E-02	2	18	164	21319	11.11%	0.77%	1.22%	0.08%	Glycolysis to A-CoA	Hkdc1, Mpc1
			MP:0011110	preweaning lethality, incomplete penetrance	8.33E-03	7.54E-02	4	18	988	21319	22.22%	4.63%	0.40%	0.08%	Glycolysis to A-CoA	Gck, Pdhb, Pfkm, Vdac1
			MP:0011997	abnormal retina outer nuclear layer thickness	8.40E-03	7.54E-02	2	18	165	21319	11.11%	0.77%	1.21%	0.08%	Glycolysis to A-CoA	Hkdc1, Mpc1
			MP:0012736	abnormal polar body morphology	8.41E-03	7.54E-02	1	18	10	21319	5.56%	0.05%	10.00%	0.08%	Glycolysis to A-CoA	Pdha1
			MP:0010097	abnormal retina blood vessel morphology	9.20E-03	7.96E-02	2	18	173	21319	11.11%	0.81%	1.16%	0.08%	Glycolysis to A-CoA	Pdhb, Pdhx
			MP:0031318	enhanced skeletal muscle regeneration	9.25E-03	7.96E-02	1	18	11	21319	5.56%	0.05%	9.09%	0.08%	Glycolysis to A-CoA	Pfkm
			MP:0005437	abnormal glycogen level	9.30E-03	7.96E-02	2	18	174	21319	11.11%	0.82%	1.15%	0.08%	Glycolysis to A-CoA	Gck, Pfkm
			MP:0010034	abnormal erythrocyte clearance	1.01E-02	8.26E-02	1	18	12	21319	5.56%	0.06%	8.33%	0.08%	Glycolysis to A-CoA	Pklr
			MP:0013404	decreased circulating lactate level	1.01E-02	8.26E-02	1	18	12	21319	5.56%	0.06%	8.33%	0.08%	Glycolysis to A-CoA	Pfkm
			MP:0010035	increased erythrocyte clearance	1.01E-02	8.26E-02	1	18	12	21319	5.56%	0.06%	8.33%	0.08%	Glycolysis to A-CoA	Pklr
			MP:0020813	abnormal photoreceptor outer segment disc membrane morphology	1.09E-02	8.69E-02	1	18	13	21319	5.56%	0.06%	7.69%	0.08%	Glycolysis to A-CoA	Mpc1
			MP:0005639	hemosiderosis	1.09E-02	8.69E-02	1	18	13	21319	5.56%	0.06%	7.69%	0.08%	Glycolysis to A-CoA	Pklr
			MP:0005438	abnormal glycogen homeostasis	1.12E-02	8.81E-02	2	18	192	21319	11.11%	0.90%	1.04%	0.08%	Glycolysis to A-CoA	Gck, Pfkm
			MP:0008859	abnormal hair cycle catagen phase	1.26E-02	9.26E-02	1	18	15	21319	5.56%	0.07%	6.67%	0.08%	Glycolysis to A-CoA	Pfkl
			MP:0009761	abnormal meiotic spindle morphology	1.26E-02	9.26E-02	1	18	15	21319	5.56%	0.07%	6.67%	0.08%	Glycolysis to A-CoA	Pdha1
			MP:0013161	enhanced muscle regeneration	1.26E-02	9.26E-02	1	18	15	21319	5.56%	0.07%	6.67%	0.08%	Glycolysis to A-CoA	Pfkm
			MP:0010375	increased kidney iron level	1.26E-02	9.26E-02	1	18	15	21319	5.56%	0.07%	6.67%	0.08%	Glycolysis to A-CoA	Hk1
			MP:0011969	abnormal circulating triglyceride level	1.26E-02	9.26E-02	3	18	590	21319	16.67%	2.77%	0.51%	0.08%	Glycolysis to A-CoA	Gck, Mpc1, Mpc2
			MP:0009978	abnormal cerebellum white matter morphology	1.34E-02	9.60E-02	1	18	16	21319	5.56%	0.08%	6.25%	0.08%	Glycolysis to A-CoA	Pdha1
			MP:0008742	abnormal kidney iron level	1.34E-02	9.60E-02	1	18	16	21319	5.56%	0.08%	6.25%	0.08%	Glycolysis to A-CoA	Hk1
			MP:0001325	abnormal retina morphology	1.39E-02	9.80E-02	4	18	1146	21319	22.22%	5.38%	0.35%	0.08%	Glycolysis to A-CoA	Hkdc1, Mpc1, Pdhb, Pdhx
			MP:0009979	abnormal cerebellum deep nucleus morphology	1.43E-02	9.95E-02	1	18	17	21319	5.56%	0.08%	5.88%	0.08%	Glycolysis to A-CoA	Pdha1
			MP:0005418	abnormal circulating hormone level	1.48E-02	1.01E-01	4	18	1168	21319	22.22%	5.48%	0.34%	0.08%	Glycolysis to A-CoA	Gck, Mpc2, Pdha1, Pfkm
			MP:0010751	decreased susceptibility to parasitic infection induced morbidity/mortality	1.51E-02	1.01E-01	1	18	18	21319	5.56%	0.08%	5.56%	0.08%	Glycolysis to A-CoA	Pklr
			MP:0011819	increased pancreatic beta cell proliferation	1.51E-02	1.01E-01	1	18	18	21319	5.56%	0.08%	5.56%	0.08%	Glycolysis to A-CoA	Gck
			MP:0002864	abnormal ocular fundus morphology	1.51E-02	1.01E-01	4	18	1176	21319	22.22%	5.52%	0.34%	0.08%	Glycolysis to A-CoA	Hkdc1, Mpc1, Pdhb, Pdhx
			MP:0001770	abnormal iron level	1.57E-02	1.02E-01	2	18	229	21319	11.11%	1.07%	0.87%	0.08%	Glycolysis to A-CoA	Hk1, Pklr
			MP:0002088	abnormal embryonic growth/weight/body size	1.58E-02	1.02E-01	4	18	1190	21319	22.22%	5.58%	0.34%	0.08%	Glycolysis to A-CoA	Dld, Hk2, Pdha1, Pdhb
			MP:0005267	abnormal olfactory cortex morphology	1.59E-02	1.02E-01	1	18	19	21319	5.56%	0.09%	5.26%	0.08%	Glycolysis to A-CoA	Pdha1
			MP:0010579	increased heart left ventricle size	1.61E-02	1.02E-01	2	18	232	21319	11.11%	1.09%	0.86%	0.08%	Glycolysis to A-CoA	Pdha1, Pfkm
			MP:0005559	increased circulating glucose level	1.64E-02	1.02E-01	3	18	650	21319	16.67%	3.05%	0.46%	0.08%	Glycolysis to A-CoA	Gck, Mpc2, Pfkm
			MP:0005637	abnormal iron homeostasis	1.65E-02	1.02E-01	2	18	235	21319	11.11%	1.10%	0.85%	0.08%	Glycolysis to A-CoA	Hk1, Pklr
			MP:0009977	abnormal cerebellar granule cell migration	1.68E-02	1.03E-01	1	18	20	21319	5.56%	0.09%	5.00%	0.08%	Glycolysis to A-CoA	Hk2
			MP:0014171	increased fatty acid oxidation	1.76E-02	1.07E-01	1	18	21	21319	5.56%	0.10%	4.76%	0.08%	Glycolysis to A-CoA	Mpc1
			MP:0003948	abnormal gas homeostasis	1.82E-02	1.07E-01	3	18	676	21319	16.67%	3.17%	0.44%	0.08%	Glycolysis to A-CoA	Mpc1, Mpc2, Pfkm
			MP:0002590	increased mean corpuscular volume	1.84E-02	1.07E-01	2	18	249	21319	11.11%	1.17%	0.80%	0.08%	Glycolysis to A-CoA	Hk1, Pklr
			MP:0001672	abnormal embryo development	1.84E-02	1.07E-01	4	18	1246	21319	22.22%	5.84%	0.32%	0.08%	Glycolysis to A-CoA	Dld, Hk2, Pdha1, Pdhb
			MP:0000840	abnormal epithalamus morphology	1.84E-02	1.07E-01	1	18	22	21319	5.56%	0.10%	4.55%	0.08%	Glycolysis to A-CoA	Pdha1
			MP:0005547	abnormal Muller cell morphology	1.92E-02	1.10E-01	1	18	23	21319	5.56%	0.11%	4.35%	0.08%	Glycolysis to A-CoA	Mpc1
			MP:0008513	thin retina inner plexiform layer	1.92E-02	1.10E-01	1	18	23	21319	5.56%	0.11%	4.35%	0.08%	Glycolysis to A-CoA	Mpc1
			MP:0013293	embryonic lethality prior to tooth bud stage	2.06E-02	1.15E-01	4	18	1289	21319	22.22%	6.05%	0.31%	0.08%	Glycolysis to A-CoA	Dld, Hk2, Pdhb, Pkm
			MP:0004952	increased spleen weight	2.07E-02	1.15E-01	2	18	265	21319	11.11%	1.24%	0.75%	0.08%	Glycolysis to A-CoA	Hk1, Pfkm
			MP:0010401	increased skeletal muscle glycogen level	2.09E-02	1.15E-01	1	18	25	21319	5.56%	0.12%	4.00%	0.08%	Glycolysis to A-CoA	Pfkm

			MP:0009414	skeletal muscle fiber necrosis	2.09E-02	1.15E-01	1	18	25	21319	5.56%	0.12%	4.00%	0.08%	Glycolysis to A-CoA	Pfkm
			MP:0000188	abnormal circulating glucose level	2.11E-02	1.15E-01	4	18	1299	21319	22.22%	6.09%	0.31%	0.08%	Glycolysis to A-CoA	Gck, Mpc1, Mpc2, Pfkm
			MP:0003953	abnormal hormone level	2.14E-02	1.15E-01	4	18	1305	21319	22.22%	6.12%	0.31%	0.08%	Glycolysis to A-CoA	Gck, Mpc2, Pdha1, Pfkm
			MP:0003728	abnormal retina photoreceptor layer morphology	2.24E-02	1.18E-01	2	18	277	21319	11.11%	1.30%	0.72%	0.08%	Glycolysis to A-CoA	Hkdc1, Mpc1
			MP:0004102	abnormal dorsal striatum morphology	2.26E-02	1.18E-01	1	18	27	21319	5.56%	0.13%	3.70%	0.08%	Glycolysis to A-CoA	Pdha1
			MP:0003731	abnormal retina outer nuclear layer morphology	2.27E-02	1.18E-01	2	18	279	21319	11.11%	1.31%	0.72%	0.08%	Glycolysis to A-CoA	Hkdc1, Mpc1
			MP:0010578	abnormal heart left ventricle size	2.29E-02	1.18E-01	2	18	280	21319	11.11%	1.31%	0.71%	0.08%	Glycolysis to A-CoA	Pdha1, Pfkm
			MP:0006036	abnormal mitochondrial physiology	2.32E-02	1.18E-01	2	18	282	21319	11.11%	1.32%	0.71%	0.08%	Glycolysis to A-CoA	Mpc1, Mpc2
			MP:0002644	decreased circulating triglyceride level	2.34E-02	1.18E-01	2	18	283	21319	11.11%	1.33%	0.71%	0.08%	Glycolysis to A-CoA	Mpc1, Mpc2
			MP:0011108	embryonic lethality during organogenesis, incomplete penetrance	2.34E-02	1.18E-01	2	18	283	21319	11.11%	1.33%	0.71%	0.08%	Glycolysis to A-CoA	Gck, Vdac1
			MP:0014071	increased cardiac muscle glycogen level	2.42E-02	1.20E-01	1	18	29	21319	5.56%	0.14%	3.45%	0.08%	Glycolysis to A-CoA	Pfkm
			MP:0010087	increased circulating fructosamine level	2.42E-02	1.20E-01	1	18	29	21319	5.56%	0.14%	3.45%	0.08%	Glycolysis to A-CoA	Gck
			MP:0005195	abnormal posterior eye segment morphology	2.56E-02	1.26E-01	4	18	1378	21319	22.22%	6.46%	0.29%	0.08%	Glycolysis to A-CoA	Hkdc1, Mpc1, Pdhb, Pdhx
			MP:0003413	hair follicle degeneration	2.59E-02	1.26E-01	1	18	31	21319	5.56%	0.15%	3.23%	0.08%	Glycolysis to A-CoA	Pfkl
			MP:0009412	skeletal muscle fiber degeneration	2.67E-02	1.27E-01	1	18	32	21319	5.56%	0.15%	3.12%	0.08%	Glycolysis to A-CoA	Pfkm
			MP:0014070	abnormal cardiac muscle glycogen level	2.75E-02	1.27E-01	1	18	33	21319	5.56%	0.15%	3.03%	0.08%	Glycolysis to A-CoA	Pfkm
			MP:0011093	embryonic lethality at implantation, complete penetrance	2.75E-02	1.27E-01	1	18	33	21319	5.56%	0.15%	3.03%	0.08%	Glycolysis to A-CoA	Pkm
			MP:0011178	increased erythroblast number	2.75E-02	1.27E-01	1	18	33	21319	5.56%	0.15%	3.03%	0.08%	Glycolysis to A-CoA	Pklr
			MP:0011628	increased mitochondrial number	2.75E-02	1.27E-01	1	18	33	21319	5.56%	0.15%	3.03%	0.08%	Glycolysis to A-CoA	Gck
			MP:0009113	increased pancreatic beta cell mass	2.75E-02	1.27E-01	1	18	33	21319	5.56%	0.15%	3.03%	0.08%	Glycolysis to A-CoA	Gck
			MP:0004196	abnormal prenatal growth/weight/body size	2.77E-02	1.27E-01	4	18	1411	21319	22.22%	6.62%	0.28%	0.08%	Glycolysis to A-CoA	Dld, Hk2, Pdha1, Pdhb
			MP:0002874	decreased hemoglobin content	2.83E-02	1.29E-01	2	18	314	21319	11.11%	1.47%	0.64%	0.08%	Glycolysis to A-CoA	Hk1, Pklr
			MP:0002792	abnormal retina vasculature morphology	3.02E-02	1.36E-01	2	18	325	21319	11.11%	1.52%	0.62%	0.08%	Glycolysis to A-CoA	Pdhb, Pdhx
			MP:0002944	increased lactate dehydrogenase level	3.24E-02	1.44E-01	1	18	39	21319	5.56%	0.18%	2.56%	0.08%	Glycolysis to A-CoA	Pfkm
			MP:0005097	polychromatophilia	3.24E-02	1.44E-01	1	18	39	21319	5.56%	0.18%	2.56%	0.08%	Glycolysis to A-CoA	Hk1
			MP:0005039	hypoxia	3.33E-02	1.46E-01	1	18	40	21319	5.56%	0.19%	2.50%	0.08%	Glycolysis to A-CoA	Pfkm
			MP:0014069	abnormal skeletal muscle glycogen level	3.41E-02	1.46E-01	1	18	41	21319	5.56%	0.19%	2.44%	0.08%	Glycolysis to A-CoA	Pfkm
			MP:0008807	increased liver iron level	3.49E-02	1.46E-01	1	18	42	21319	5.56%	0.20%	2.38%	0.08%	Glycolysis to A-CoA	Hk1
			MP:0001759	increased urine glucose level	3.49E-02	1.46E-01	1	18	42	21319	5.56%	0.20%	2.38%	0.08%	Glycolysis to A-CoA	Gck
			MP:0003852	skeletal muscle necrosis	3.49E-02	1.46E-01	1	18	42	21319	5.56%	0.20%	2.38%	0.08%	Glycolysis to A-CoA	Pfkm
			MP:0002079	increased circulating insulin level	3.51E-02	1.46E-01	2	18	353	21319	11.11%	1.66%	0.57%	0.08%	Glycolysis to A-CoA	Gck, Pdha1
			MP:0031346	abnormal eye vasculature morphology	3.53E-02	1.46E-01	2	18	354	21319	11.11%	1.66%	0.56%	0.08%	Glycolysis to A-CoA	Pdhb, Pdhx
			MP:0011818	abnormal pancreatic beta cell proliferation	3.57E-02	1.46E-01	1	18	43	21319	5.56%	0.20%	2.33%	0.08%	Glycolysis to A-CoA	Gck
			MP:0031116	microvesicular hepatic steatosis	3.57E-02	1.46E-01	1	18	43	21319	5.56%	0.20%	2.33%	0.08%	Glycolysis to A-CoA	Gck
			MP:0008772	increased heart ventricle size	3.64E-02	1.46E-01	2	18	360	21319	11.11%	1.69%	0.56%	0.08%	Glycolysis to A-CoA	Pdha1, Pfkm
			MP:0013661	abnormal myeloid cell number	3.64E-02	1.46E-01	3	18	884	21319	16.67%	4.15%	0.34%	0.08%	Glycolysis to A-CoA	Hk1, Pfkm, Pklr
			MP:0001691	abnormal somite shape	3.65E-02	1.46E-01	1	18	44	21319	5.56%	0.21%	2.27%	0.08%	Glycolysis to A-CoA	Pdhb
			MP:0008527	embryonic lethality at implantation	3.65E-02	1.46E-01	1	18	44	21319	5.56%	0.21%	2.27%	0.08%	Glycolysis to A-CoA	Pkm
			MP:0001697	abnormal embryo size	3.66E-02	1.46E-01	3	18	886	21319	16.67%	4.16%	0.34%	0.08%	Glycolysis to A-CoA	Dld, Pdha1, Pdhb
			MP:0002722	abnormal immune system organ morphology	3.71E-02	1.46E-01	5	18	2292	21319	27.78%	10.75%	0.22%	0.08%	Glycolysis to A-CoA	Hk1, Hk3, Pdha2, Pfkm, Pklr
			MP:0011177	abnormal erythroblast number	3.73E-02	1.46E-01	1	18	45	21319	5.56%	0.21%	2.22%	0.08%	Glycolysis to A-CoA	Pklr
			MP:0003734	abnormal retina inner plexiform layer morphology	3.73E-02	1.46E-01	1	18	45	21319	5.56%	0.21%	2.22%	0.08%	Glycolysis to A-CoA	Mpc1
			MP:0009969	abnormal cerebral cortex pyramidal cell morphology	3.81E-02	1.49E-01	1	18	46	21319	5.56%	0.22%	2.17%	0.08%	Glycolysis to A-CoA	Pdha1
			MP:0002875	decreased erythrocyte cell number	3.86E-02	1.49E-01	2	18	372	21319	11.11%	1.74%	0.54%	0.08%	Glycolysis to A-CoA	Hk1, Pklr
			MP:0005318	decreased triglyceride level	3.86E-02	1.49E-01	2	18	372	21319	11.11%	1.74%	0.54%	0.08%	Glycolysis to A-CoA	Mpc1, Mpc2
			MP:0001758	abnormal urine glucose level	3.90E-02	1.49E-01	1	18	47	21319	5.56%	0.22%	2.13%	0.08%	Glycolysis to A-CoA	Gck
			MP:0006207	embryonic lethality during organogenesis	3.94E-02	1.49E-01	3	18	912	21319	16.67%	4.28%	0.33%	0.08%	Glycolysis to A-CoA	Gck, Mpc2, Vdac1
			MP:0000598	abnormal liver morphology	3.95E-02	1.49E-01	4	18	1576	21319	22.22%	7.39%	0.25%	0.08%	Glycolysis to A-CoA	Gck, Hk1, Hkdc1, Pfkm
			MP:0009415	skeletal muscle degeneration	4.06E-02	1.50E-01	1	18	49	21319	5.56%	0.23%	2.04%	0.08%	Glycolysis to A-CoA	Pfkm

			MP:0001588	abnormal hemoglobin	4.07E-02	1.50E-01	3	18	924	21319	16.67%	4.33%	0.32%	0.08%	Glycolysis to A-CoA	Gck, Hk1, Pklr
			MP:0004951	abnormal spleen weight	4.11E-02	1.50E-01	2	18	385	21319	11.11%	1.81%	0.52%	0.08%	Glycolysis to A-CoA	Hk1, Pfkm
			MP:0002447	abnormal erythrocyte morphology	4.12E-02	1.50E-01	4	18	1598	21319	22.22%	7.50%	0.25%	0.08%	Glycolysis to A-CoA	Gck, Hk1, Pfkm, Pklr
			MP:0002164	abnormal gland physiology	4.14E-02	1.50E-01	3	18	930	21319	16.67%	4.36%	0.32%	0.08%	Glycolysis to A-CoA	Gck, Mpc2, Pfkm
			MP:0013237	abnormal skeletal muscle regeneration	4.14E-02	1.50E-01	1	18	50	21319	5.56%	0.23%	2.00%	0.08%	Glycolysis to A-CoA	Pfkm
			MP:0001577	anemia	4.19E-02	1.51E-01	2	18	389	21319	11.11%	1.82%	0.51%	0.08%	Glycolysis to A-CoA	Hk1, Pklr
			MP:0002768	small adrenal glands	4.22E-02	1.51E-01	1	18	51	21319	5.56%	0.24%	1.96%	0.08%	Glycolysis to A-CoA	Hk3
			MP:0013707	abnormal hematopoietic precursor cell morphology	4.31E-02	1.53E-01	2	18	395	21319	11.11%	1.85%	0.51%	0.08%	Glycolysis to A-CoA	Pfkm, Pklr
			MP:0000691	enlarged spleen	4.37E-02	1.54E-01	3	18	950	21319	16.67%	4.46%	0.32%	0.08%	Glycolysis to A-CoA	Hk1, Pfkm, Pklr
			MP:0008738	abnormal liver iron level	4.38E-02	1.54E-01	1	18	53	21319	5.56%	0.25%	1.89%	0.08%	Glycolysis to A-CoA	Hk1
			MP:0011084	lethality at weaning, incomplete penetrance	4.46E-02	1.56E-01	1	18	54	21319	5.56%	0.25%	1.85%	0.08%	Glycolysis to A-CoA	Pfkm
			MP:0011176	abnormal erythroblast morphology	4.55E-02	1.57E-01	1	18	55	21319	5.56%	0.26%	1.82%	0.08%	Glycolysis to A-CoA	Pklr
			MP:0009172	small pancreatic islets	4.55E-02	1.57E-01	1	18	55	21319	5.56%	0.26%	1.82%	0.08%	Glycolysis to A-CoA	Gck
			MP:0008511	decreased retina inner nuclear layer thickness	4.63E-02	1.58E-01	1	18	56	21319	5.56%	0.26%	1.79%	0.08%	Glycolysis to A-CoA	Hkdc1
			MP:0000689	abnormal spleen morphology	4.66E-02	1.59E-01	4	18	1662	21319	22.22%	7.80%	0.24%	0.08%	Glycolysis to A-CoA	Hk1, Pdha2, Pfkm, Pklr
			MP:0013659	abnormal erythroid lineage cell morphology	4.72E-02	1.60E-01	4	18	1668	21319	22.22%	7.82%	0.24%	0.08%	Glycolysis to A-CoA	Gck, Hk1, Pfkm, Pklr
			MP:0000781	decreased corpus callosum size	4.79E-02	1.60E-01	1	18	58	21319	5.56%	0.27%	1.72%	0.08%	Glycolysis to A-CoA	Pdha1
			MP:0003135	increased erythroid progenitor cell number	4.79E-02	1.60E-01	1	18	58	21319	5.56%	0.27%	1.72%	0.08%	Glycolysis to A-CoA	Pklr
			MP:0009395	increased nucleated erythrocyte cell number	4.87E-02	1.62E-01	1	18	59	21319	5.56%	0.28%	1.69%	0.08%	Glycolysis to A-CoA	Pklr
			MP:0001625	cardiac hypertrophy	4.94E-02	1.63E-01	2	18	426	21319	11.11%	2.00%	0.47%	0.08%	Glycolysis to A-CoA	Pdha1, Pfkm
			MP:0011996	abnormal retina inner nuclear layer thickness	4.95E-02	1.63E-01	1	18	60	21319	5.56%	0.28%	1.67%	0.08%	Glycolysis to A-CoA	Hkdc1
			MP:0020573	abnormal corpus callosum size	5.11E-02	1.65E-01	1	18	62	21319	5.56%	0.29%	1.61%	0.08%	Glycolysis to A-CoA	Pdha1
			MP:0010953	abnormal fatty acid oxidation	5.11E-02	1.65E-01	1	18	62	21319	5.56%	0.29%	1.61%	0.08%	Glycolysis to A-CoA	Mpc1
			MP:0012397	abnormal nucleated erythrocyte cell number	5.11E-02	1.65E-01	1	18	62	21319	5.56%	0.29%	1.61%	0.08%	Glycolysis to A-CoA	Pklr
			MP:0003383	abnormal gluconeogenesis	5.19E-02	1.66E-01	1	18	63	21319	5.56%	0.30%	1.59%	0.08%	Glycolysis to A-CoA	Mpc1
			MP:0009114	decreased pancreatic beta cell mass	5.19E-02	1.66E-01	1	18	63	21319	5.56%	0.30%	1.59%	0.08%	Glycolysis to A-CoA	Gck
			MP:0011186	abnormal visceral endoderm morphology	5.27E-02	1.66E-01	1	18	64	21319	5.56%	0.30%	1.56%	0.08%	Glycolysis to A-CoA	Dld
			MP:0008518	retina outer nuclear layer degeneration	5.27E-02	1.66E-01	1	18	64	21319	5.56%	0.30%	1.56%	0.08%	Glycolysis to A-CoA	Hkdc1
			MP:0011091	prenatal lethality, complete penetrance	5.29E-02	1.66E-01	2	18	443	21319	11.11%	2.08%	0.45%	0.08%	Glycolysis to A-CoA	Pdha1, Pkm
			MP:0014068	abnormal muscle glycogen level	5.35E-02	1.66E-01	1	18	65	21319	5.56%	0.30%	1.54%	0.08%	Glycolysis to A-CoA	Pfkm
			MP:0003896	prolonged PR interval	5.35E-02	1.66E-01	1	18	65	21319	5.56%	0.30%	1.54%	0.08%	Glycolysis to A-CoA	Gck
			MP:0002138	abnormal hepatobiliary system morphology	5.39E-02	1.66E-01	4	18	1742	21319	22.22%	8.17%	0.23%	0.08%	Glycolysis to A-CoA	Gck, Hk1, Hkdc1, Pfkm
			MP:0002943	abnormal lactate dehydrogenase level	5.51E-02	1.68E-01	1	18	67	21319	5.56%	0.31%	1.49%	0.08%	Glycolysis to A-CoA	Pfkm
			MP:0000832	abnormal thalamus morphology	5.51E-02	1.68E-01	1	18	67	21319	5.56%	0.31%	1.49%	0.08%	Glycolysis to A-CoA	Pdha1
			MP:0001926	female infertility	5.55E-02	1.69E-01	2	18	455	21319	11.11%	2.13%	0.44%	0.08%	Glycolysis to A-CoA	Hk1, Pdha1
			MP:0001304	cataract	5.66E-02	1.71E-01	2	18	460	21319	11.11%	2.16%	0.43%	0.08%	Glycolysis to A-CoA	Hk3, Pdhx
			MP:0010866	abnormal prenatal body size	5.74E-02	1.72E-01	3	18	1061	21319	16.67%	4.98%	0.28%	0.08%	Glycolysis to A-CoA	Dld, Pdha1, Pdhb
			MP:0010951	abnormal lipid oxidation	5.75E-02	1.72E-01	1	18	70	21319	5.56%	0.33%	1.43%	0.08%	Glycolysis to A-CoA	Mpc1
			MP:0008547	abnormal neocortex morphology	5.83E-02	1.72E-01	1	18	71	21319	5.56%	0.33%	1.41%	0.08%	Glycolysis to A-CoA	Pdha1
			MP:0010398	decreased liver glycogen level	5.83E-02	1.72E-01	1	18	71	21319	5.56%	0.33%	1.41%	0.08%	Glycolysis to A-CoA	Gck
			MP:0008476	increased spleen red pulp amount	5.83E-02	1.72E-01	1	18	71	21319	5.56%	0.33%	1.41%	0.08%	Glycolysis to A-CoA	Hk1
			MP:0000750	abnormal muscle regeneration	5.91E-02	1.72E-01	1	18	72	21319	5.56%	0.34%	1.39%	0.08%	Glycolysis to A-CoA	Pfkm
			MP:0003058	increased insulin secretion	5.91E-02	1.72E-01	1	18	72	21319	5.56%	0.34%	1.39%	0.08%	Glycolysis to A-CoA	Gck
			MP:0000226	abnormal mean corpuscular volume	6.04E-02	1.75E-01	2	18	477	21319	11.11%	2.24%	0.42%	0.08%	Glycolysis to A-CoA	Hk1, Pklr
			MP:0002642	anisocytosis	6.15E-02	1.77E-01	1	18	75	21319	5.56%	0.35%	1.33%	0.08%	Glycolysis to A-CoA	Hk1
			MP:0002221	abnormal lymph organ size	6.30E-02	1.80E-01	4	18	1833	21319	22.22%	8.60%	0.22%	0.08%	Glycolysis to A-CoA	Hk1, Hk3, Pfkm, Pklr
			MP:0001547	abnormal lipid level	6.31E-02	1.80E-01	4	18	1834	21319	22.22%	8.60%	0.22%	0.08%	Glycolysis to A-CoA	Gck, Hkdc1, Mpc1, Mpc2
			MP:0008587	short photoreceptor outer segment	6.47E-02	1.82E-01	1	18	79	21319	5.56%	0.37%	1.27%	0.08%	Glycolysis to A-CoA	Mpc1
			MP:0012143	decreased a-wave amplitude	6.55E-02	1.82E-01	1	18	80	21319	5.56%	0.38%	1.25%	0.08%	Glycolysis to A-CoA	Hkdc1
			MP:0004809	increased hematopoietic stem cell number	6.55E-02	1.82E-01	1	18	80	21319	5.56%	0.38%	1.25%	0.08%	Glycolysis to A-CoA	Pfkm
			MP:0001586	abnormal erythrocyte cell number	6.58E-02	1.82E-01	2	18	501	21319	11.11%	2.35%	0.40%	0.08%	Glycolysis to A-CoA	Hk1, Pklr
			MP:0005026	decreased susceptibility to parasitic infection	6.63E-02	1.82E-01	1	18	81	21319	5.56%	0.38%	1.23%	0.08%	Glycolysis to A-CoA	Pklr
			MP:0012030	abnormal a-wave amplitude	6.70E-02	1.82E-01	1	18	82	21319	5.56%	0.38%	1.22%	0.08%	Glycolysis to A-CoA	Hkdc1

			MP:0000872	abnormal cerebellum external granule cell layer morphology	6.70E-02	1.82E-01	1	18	82	21319	5.56%	0.38%	1.22%	0.08%	Glycolysis to A-CoA	Hk2
			MP:0011635	abnormal mitochondrial crista morphology	6.70E-02	1.82E-01	1	18	82	21319	5.56%	0.38%	1.22%	0.08%	Glycolysis to A-CoA	Gck
			MP:0020811	abnormal photoreceptor outer segment size	6.70E-02	1.82E-01	1	18	82	21319	5.56%	0.38%	1.22%	0.08%	Glycolysis to A-CoA	Mpc1
			MP:0013292	embryonic lethality prior to organogenesis	6.73E-02	1.82E-01	3	18	1133	21319	16.67%	5.31%	0.26%	0.08%	Glycolysis to A-CoA	Dld, Hk2, Pkm
			MP:0012034	abnormal a-wave shape	6.78E-02	1.82E-01	1	18	83	21319	5.56%	0.39%	1.20%	0.08%	Glycolysis to A-CoA	Hkdc1
			MP:0005290	decreased oxygen consumption	6.78E-02	1.82E-01	1	18	83	21319	5.56%	0.39%	1.20%	0.08%	Glycolysis to A-CoA	Mpc2
			MP:0009850	embryonic lethality between implantation and placentation	6.81E-02	1.82E-01	2	18	511	21319	11.11%	2.40%	0.39%	0.08%	Glycolysis to A-CoA	Dld, Hk2
			MP:0003730	abnormal photoreceptor inner segment morphology	7.02E-02	1.86E-01	1	18	86	21319	5.56%	0.40%	1.16%	0.08%	Glycolysis to A-CoA	Mpc1
			MP:0002416	abnormal proerythroblast morphology	7.02E-02	1.86E-01	1	18	86	21319	5.56%	0.40%	1.16%	0.08%	Glycolysis to A-CoA	Pklr
			MP:0011634	abnormal mitochondrial inner membrane morphology	7.10E-02	1.87E-01	1	18	87	21319	5.56%	0.41%	1.15%	0.08%	Glycolysis to A-CoA	Gck
			MP:0000880	decreased Purkinje cell number	7.18E-02	1.89E-01	1	18	88	21319	5.56%	0.41%	1.14%	0.08%	Glycolysis to A-CoA	Pdha1
			MP:0006069	abnormal retina neuronal layer morphology	7.24E-02	1.89E-01	2	18	529	21319	11.11%	2.48%	0.38%	0.08%	Glycolysis to A-CoA	Hkdc1, Mpc1
			MP:0009112	abnormal pancreatic beta cell mass	7.26E-02	1.89E-01	1	18	89	21319	5.56%	0.42%	1.12%	0.08%	Glycolysis to A-CoA	Gck
			MP:0010519	atrioventricular block	7.34E-02	1.90E-01	1	18	90	21319	5.56%	0.42%	1.11%	0.08%	Glycolysis to A-CoA	Gck
			MP:0000889	abnormal cerebellar molecular layer	7.41E-02	1.90E-01	1	18	91	21319	5.56%	0.43%	1.10%	0.08%	Glycolysis to A-CoA	Pdha1
			MP:0005440	increased glycogen level	7.41E-02	1.90E-01	1	18	91	21319	5.56%	0.43%	1.10%	0.08%	Glycolysis to A-CoA	Pfkm
			MP:0000849	abnormal cerebellum morphology	7.45E-02	1.90E-01	2	18	538	21319	11.11%	2.52%	0.37%	0.08%	Glycolysis to A-CoA	Hk2, Pdha1
			MP:0000878	abnormal Purkinje cell number	7.49E-02	1.90E-01	1	18	92	21319	5.56%	0.43%	1.09%	0.08%	Glycolysis to A-CoA	Pdha1
			MP:0005439	decreased glycogen level	7.49E-02	1.90E-01	1	18	92	21319	5.56%	0.43%	1.09%	0.08%	Glycolysis to A-CoA	Gck
			MP:0013328	visceromegaly	7.55E-02	1.90E-01	3	18	1189	21319	16.67%	5.58%	0.25%	0.08%	Glycolysis to A-CoA	Hk1, Pfkm, Pklr
			MP:0000749	muscle degeneration	7.57E-02	1.90E-01	1	18	93	21319	5.56%	0.44%	1.08%	0.08%	Glycolysis to A-CoA	Pfkm
			MP:0005168	abnormal female meiosis	7.65E-02	1.91E-01	1	18	94	21319	5.56%	0.44%	1.06%	0.08%	Glycolysis to A-CoA	Pdha1
			MP:0002833	increased heart weight	7.67E-02	1.91E-01	2	18	547	21319	11.11%	2.57%	0.37%	0.08%	Glycolysis to A-CoA	Pdha1, Pfkm
			MP:0002118	abnormal lipid homeostasis	7.75E-02	1.92E-01	4	18	1965	21319	22.22%	9.22%	0.20%	0.08%	Glycolysis to A-CoA	Gck, Hkdc1, Mpc1, Mpc2
			MP:0011097	embryonic lethality between somite formation and embryo turning, complete penetrance	7.81E-02	1.92E-01	1	18	96	21319	5.56%	0.45%	1.04%	0.08%	Glycolysis to A-CoA	Hk2
			MP:0002092	abnormal eye morphology	7.83E-02	1.92E-01	5	18	2815	21319	27.78%	13.20%	0.18%	0.08%	Glycolysis to A-CoA	Hk3, Hkdc1, Mpc1, Pdhb, Pdhx
			MP:0005370	liver/biliary system phenotype	7.84E-02	1.92E-01	4	18	1973	21319	22.22%	9.25%	0.20%	0.08%	Glycolysis to A-CoA	Gck, Hk1, Hkdc1, Pfkm
			MP:0012144	decreased b-wave amplitude	7.88E-02	1.92E-01	1	18	97	21319	5.56%	0.45%	1.03%	0.08%	Glycolysis to A-CoA	Hkdc1
			MP:0003901	abnormal PR interval	7.96E-02	1.92E-01	1	18	98	21319	5.56%	0.46%	1.02%	0.08%	Glycolysis to A-CoA	Gck
			MP:0012106	impaired exercise endurance	8.04E-02	1.92E-01	1	18	99	21319	5.56%	0.46%	1.01%	0.08%	Glycolysis to A-CoA	Pfkm
			MP:0010392	prolonged QRS complex duration	8.04E-02	1.92E-01	1	18	99	21319	5.56%	0.46%	1.01%	0.08%	Glycolysis to A-CoA	Gck
			MP:0012031	abnormal b-wave amplitude	8.12E-02	1.92E-01	1	18	100	21319	5.56%	0.47%	1.00%	0.08%	Glycolysis to A-CoA	Hkdc1
			MP:0012035	abnormal b-wave shape	8.12E-02	1.92E-01	1	18	100	21319	5.56%	0.47%	1.00%	0.08%	Glycolysis to A-CoA	Hkdc1
			MP:0000427	abnormal hair cycle	8.12E-02	1.92E-01	1	18	100	21319	5.56%	0.47%	1.00%	0.08%	Glycolysis to A-CoA	Pfkl
			MP:0003921	abnormal heart left ventricle morphology	8.19E-02	1.92E-01	2	18	568	21319	11.11%	2.66%	0.35%	0.08%	Glycolysis to A-CoA	Pdha1, Pfkm
			MP:0010636	bundle branch block	8.20E-02	1.92E-01	1	18	101	21319	5.56%	0.47%	0.99%	0.08%	Glycolysis to A-CoA	Gck
			MP:0010560	intraventricular block	8.20E-02	1.92E-01	1	18	101	21319	5.56%	0.47%	0.99%	0.08%	Glycolysis to A-CoA	Gck
			MP:0010379	decreased respiratory quotient	8.27E-02	1.93E-01	1	18	102	21319	5.56%	0.48%	0.98%	0.08%	Glycolysis to A-CoA	Mpc1
			MP:0000847	abnormal metencephalon morphology	8.36E-02	1.94E-01	2	18	575	21319	11.11%	2.70%	0.35%	0.08%	Glycolysis to A-CoA	Hk2, Pdha1
			MP:0009786	decreased susceptibility to infection induced morbidity/mortality	8.58E-02	1.99E-01	1	18	106	21319	5.56%	0.50%	0.94%	0.08%	Glycolysis to A-CoA	Pklr
			MP:0008569	lethality at weaning	8.66E-02	2.00E-01	1	18	107	21319	5.56%	0.50%	0.93%	0.08%	Glycolysis to A-CoA	Pfkm
			MP:0009788	increased susceptibility to bacterial infection induced morbidity/mortality	8.74E-02	2.01E-01	1	18	108	21319	5.56%	0.51%	0.93%	0.08%	Glycolysis to A-CoA	Pklr
			MP:0004948	abnormal neuronal precursor proliferation	8.89E-02	2.02E-01	1	18	110	21319	5.56%	0.52%	0.91%	0.08%	Glycolysis to A-CoA	Hk2
			MP:0002899	fatigue	8.89E-02	2.02E-01	1	18	110	21319	5.56%	0.52%	0.91%	0.08%	Glycolysis to A-CoA	Pfkm
			MP:0010086	abnormal circulating fructosamine level	8.97E-02	2.02E-01	1	18	111	21319	5.56%	0.52%	0.90%	0.08%	Glycolysis to A-CoA	Gck
			MP:0012777	abnormal liver glycogen level	8.97E-02	2.02E-01	1	18	111	21319	5.56%	0.52%	0.90%	0.08%	Glycolysis to A-CoA	Gck
			MP:0001157	small seminal vesicle	8.97E-02	2.02E-01	1	18	111	21319	5.56%	0.52%	0.90%	0.08%	Glycolysis to A-CoA	Pfkm
			MP:0003727	abnormal retina layer morphology	9.10E-02	2.04E-01	2	18	604	21319	11.11%	2.83%	0.33%	0.08%	Glycolysis to A-CoA	Hkdc1, Mpc1
			MP:0013662	decreased myeloid cell number	9.33E-02	2.08E-01	2	18	613	21319	11.11%	2.88%	0.33%	0.08%	Glycolysis to A-CoA	Hk1, Pklr
			MP:0006206	embryonic lethality between somite formation and embryo turning	9.36E-02	2.08E-01	1	18	116	21319	5.56%	0.54%	0.86%	0.08%	Glycolysis to A-CoA	Hk2
			MP:0004077	abnormal striatum morphology	9.43E-02	2.09E-01	1	18	117	21319	5.56%	0.55%	0.85%	0.08%	Glycolysis to A-CoA	Pdha1

			MP:0009404	centrally nucleated skeletal muscle fibers	9.51E-02	2.10E-01	1	18	118	21319	5.56%	0.55%	0.85%	0.08%	Glycolysis to A-CoA	Pfkm
			MP:0011925	abnormal heart echocardiography feature	9.59E-02	2.11E-01	1	18	119	21319	5.56%	0.56%	0.84%	0.08%	Glycolysis to A-CoA	Gck
			MP:0001260	increased body weight	9.72E-02	2.12E-01	2	18	628	21319	11.11%	2.95%	0.32%	0.08%	Glycolysis to A-CoA	Gck, Pdha1
			MP:0012323	increased total tissue mass	9.72E-02	2.12E-01	2	18	628	21319	11.11%	2.95%	0.32%	0.08%	Glycolysis to A-CoA	Gck, Pdha1
			MP:0002224	abnormal spleen size	9.84E-02	2.14E-01	3	18	1331	21319	16.67%	6.24%	0.23%	0.08%	Glycolysis to A-CoA	Hk1, Pfkm, Pklr
			MP:0012029	abnormal electroretinogram waveform feature	9.89E-02	2.14E-01	1	18	123	21319	5.56%	0.58%	0.81%	0.08%	Glycolysis to A-CoA	Hkdc1
			MP:0006141	abnormal atrioventricular node conduction	1.00E-01	2.17E-01	1	18	125	21319	5.56%	0.59%	0.80%	0.08%	Glycolysis to A-CoA	Gck
			MP:0001148	enlarged testis	1.01E-01	2.17E-01	1	18	126	21319	5.56%	0.59%	0.79%	0.08%	Glycolysis to A-CoA	Pfkm
			MP:0001764	abnormal homeostasis	1.02E-01	2.17E-01	9	18	7032	21319	50.00%	32.98%	0.13%	0.08%	Glycolysis to A-CoA	Gck, Hk1, Hkdc1, Mpc1, Mpc2, Pdha1, Pfkm, Pklr, Vdac1
			MP:0009170	abnormal pancreatic islet size	1.02E-01	2.17E-01	1	18	127	21319	5.56%	0.60%	0.79%	0.08%	Glycolysis to A-CoA	Gck
			MP:0000841	abnormal hindbrain morphology	1.02E-01	2.17E-01	2	18	647	21319	11.11%	3.03%	0.31%	0.08%	Glycolysis to A-CoA	Hk2, Pdha1
			MP:0001698	decreased embryo size	1.02E-01	2.17E-01	2	18	648	21319	11.11%	3.04%	0.31%	0.08%	Glycolysis to A-CoA	Dld, Pdha1
			MP:0002332	abnormal exercise endurance	1.03E-01	2.17E-01	1	18	128	21319	5.56%	0.60%	0.78%	0.08%	Glycolysis to A-CoA	Pfkm
			MP:0005376	homeostasis/metabolism phenotype	1.06E-01	2.22E-01	10	18	8180	21319	55.56%	38.37%	0.12%	0.08%	Glycolysis to A-CoA	Gck, Hk1, Hkdc1, Mpc1, Mpc2, Pdha1, Pfkl, Pfkm, Pklr, Vdac1
			MP:0004022	abnormal cone electrophysiology	1.07E-01	2.23E-01	1	18	133	21319	5.56%	0.62%	0.75%	0.08%	Glycolysis to A-CoA	Mpc1
			MP:0000886	abnormal cerebellar granule layer morphology	1.09E-01	2.24E-01	1	18	136	21319	5.56%	0.64%	0.74%	0.08%	Glycolysis to A-CoA	Pdha1
			MP:0006009	abnormal neuronal migration	1.09E-01	2.24E-01	1	18	136	21319	5.56%	0.64%	0.74%	0.08%	Glycolysis to A-CoA	Hk2
			MP:0006410	abnormal common myeloid progenitor cell morphology	1.10E-01	2.24E-01	1	18	137	21319	5.56%	0.64%	0.73%	0.08%	Glycolysis to A-CoA	Pklr
			MP:0008801	abnormal erythroid progenitor cell morphology	1.10E-01	2.24E-01	1	18	137	21319	5.56%	0.64%	0.73%	0.08%	Glycolysis to A-CoA	Pklr
			MP:0003733	abnormal retina inner nuclear layer morphology	1.10E-01	2.24E-01	1	18	137	21319	5.56%	0.64%	0.73%	0.08%	Glycolysis to A-CoA	Hkdc1
			MP:0003956	abnormal body size	1.10E-01	2.24E-01	6	18	4057	21319	33.33%	19.03%	0.15%	0.08%	Glycolysis to A-CoA	Gck, Hk1, Hk2, Mpc1, Pdha1, Pfkm
			MP:0003898	abnormal QRS complex	1.10E-01	2.24E-01	1	18	138	21319	5.56%	0.65%	0.72%	0.08%	Glycolysis to A-CoA	Gck
			MP:0000189	hypoglycemia	1.10E-01	2.24E-01	1	18	138	21319	5.56%	0.65%	0.72%	0.08%	Glycolysis to A-CoA	Gck
			MP:0001264	increased body size	1.11E-01	2.24E-01	2	18	680	21319	11.11%	3.19%	0.29%	0.08%	Glycolysis to A-CoA	Gck, Pdha1
			MP:0001554	increased circulating free fatty acids level	1.11E-01	2.24E-01	1	18	139	21319	5.56%	0.65%	0.72%	0.08%	Glycolysis to A-CoA	Gck
			MP:0004564	enlarged myocardial fiber	1.12E-01	2.25E-01	1	18	140	21319	5.56%	0.66%	0.71%	0.08%	Glycolysis to A-CoA	Pdha1
			MP:0005391	vision/eye phenotype	1.13E-01	2.25E-01	5	18	3133	21319	27.78%	14.70%	0.16%	0.08%	Glycolysis to A-CoA	Hk3, Hkdc1, Mpc1, Pdhb, Pdhx
			MP:0004857	abnormal heart weight	1.13E-01	2.25E-01	2	18	686	21319	11.11%	3.22%	0.29%	0.08%	Glycolysis to A-CoA	Pdha1, Pfkm
			MP:0009355	increased liver triglyceride level	1.14E-01	2.27E-01	1	18	143	21319	5.56%	0.67%	0.70%	0.08%	Glycolysis to A-CoA	Hkdc1
			MP:0009967	abnormal neuron proliferation	1.16E-01	2.31E-01	1	18	146	21319	5.56%	0.68%	0.68%	0.08%	Glycolysis to A-CoA	Hk2
			MP:0001303	abnormal lens morphology	1.17E-01	2.31E-01	2	18	701	21319	11.11%	3.29%	0.29%	0.08%	Glycolysis to A-CoA	Hk3, Pdhx
			MP:0004021	abnormal rod electrophysiology	1.17E-01	2.31E-01	1	18	147	21319	5.56%	0.69%	0.68%	0.08%	Glycolysis to A-CoA	Mpc1
			MP:0005560	decreased circulating glucose level	1.19E-01	2.33E-01	2	18	710	21319	11.11%	3.33%	0.28%	0.08%	Glycolysis to A-CoA	Gck, Mpc1
			MP:0001125	abnormal oocyte morphology	1.19E-01	2.33E-01	1	18	150	21319	5.56%	0.70%	0.67%	0.08%	Glycolysis to A-CoA	Pdha1
			MP:0002175	decreased brain weight	1.20E-01	2.34E-01	1	18	151	21319	5.56%	0.71%	0.66%	0.08%	Glycolysis to A-CoA	Pdha1
			MP:0005253	abnormal eye physiology	1.21E-01	2.35E-01	2	18	717	21319	11.11%	3.36%	0.28%	0.08%	Glycolysis to A-CoA	Hkdc1, Mpc1
			MP:0000854	abnormal cerebellum development	1.22E-01	2.35E-01	1	18	153	21319	5.56%	0.72%	0.65%	0.08%	Glycolysis to A-CoA	Hk2
			MP:0002356	abnormal spleen red pulp morphology	1.22E-01	2.35E-01	1	18	153	21319	5.56%	0.72%	0.65%	0.08%	Glycolysis to A-CoA	Hk1
			MP:0004087	abnormal muscle fiber morphology	1.22E-01	2.35E-01	2	18	721	21319	11.11%	3.38%	0.28%	0.08%	Glycolysis to A-CoA	Pdha1, Pfkm
			MP:0005380	embryo phenotype	1.23E-01	2.36E-01	4	18	2312	21319	22.22%	10.84%	0.17%	0.08%	Glycolysis to A-CoA	Dld, Hk2, Pdha1, Pdhb
			MP:0000780	abnormal corpus callosum morphology	1.25E-01	2.39E-01	1	18	158	21319	5.56%	0.74%	0.63%	0.08%	Glycolysis to A-CoA	Pdha1
			MP:0004907	abnormal seminal vesicle size	1.26E-01	2.40E-01	1	18	159	21319	5.56%	0.75%	0.63%	0.08%	Glycolysis to A-CoA	Pfkm
			MP:0001289	persistence of hyaloid vascular system	1.27E-01	2.41E-01	1	18	160	21319	5.56%	0.75%	0.62%	0.08%	Glycolysis to A-CoA	Pdhx
			MP:0006007	abnormal basal ganglion morphology	1.28E-01	2.41E-01	1	18	162	21319	5.56%	0.76%	0.62%	0.08%	Glycolysis to A-CoA	Pdha1
			MP:0008219	abnormal dorsal telencephalic commissure morphology	1.28E-01	2.41E-01	1	18	162	21319	5.56%	0.76%	0.62%	0.08%	Glycolysis to A-CoA	Pdha1
			MP:0006361	abnormal female germ cell morphology	1.28E-01	2.41E-01	1	18	162	21319	5.56%	0.76%	0.62%	0.08%	Glycolysis to A-CoA	Pdha1
			MP:0010559	heart block	1.31E-01	2.46E-01	1	18	166	21319	5.56%	0.78%	0.60%	0.08%	Glycolysis to A-CoA	Gck
			MP:0000852	small cerebellum	1.32E-01	2.46E-01	1	18	167	21319	5.56%	0.78%	0.60%	0.08%	Glycolysis to A-CoA	Pdha1
			MP:0004808	abnormal hematopoietic stem cell morphology	1.34E-01	2.48E-01	1	18	169	21319	5.56%	0.79%	0.59%	0.08%	Glycolysis to A-CoA	Pfkm
			MP:0001326	retina degeneration	1.35E-01	2.49E-01	1	18	171	21319	5.56%	0.80%	0.58%	0.08%	Glycolysis to A-CoA	Mpc1
			MP:0003949	abnormal circulating lipid level	1.35E-01	2.49E-01	3	18	1532	21319	16.67%	7.19%	0.20%	0.08%	Glycolysis to A-CoA	Gck, Mpc1, Mpc2
			MP:0009642	abnormal blood homeostasis	1.36E-01	2.50E-01	7	18	5301	21319	38.89%	24.87%	0.13%	0.08%	Glycolysis to A-CoA	Gck, Hk1, Mpc1, Mpc2, Pdha1, Pfkm, Pklr
			MP:0004129	abnormal respiratory quotient	1.37E-01	2.52E-01	1	18	174	21319	5.56%	0.82%	0.57%	0.08%	Glycolysis to A-CoA	Mpc1

			MP:0001265	decreased body size	1.38E-01	2.53E-01	5	18	3339	21319	27.78%	15.66%	0.15%	0.08%	Glycolysis to A-CoA	Gck, Hk1, Hk2, Mpc1, Pfkm
			MP:0001454	abnormal cued conditioning behavior	1.39E-01	2.53E-01	1	18	176	21319	5.56%	0.83%	0.57%	0.08%	Glycolysis to A-CoA	Vdac1
			MP:0002884	abnormal pharyngeal arch morphology	1.39E-01	2.53E-01	1	18	177	21319	5.56%	0.83%	0.56%	0.08%	Glycolysis to A-CoA	Pdhb
			MP:0005451	abnormal body composition	1.40E-01	2.54E-01	5	18	3351	21319	27.78%	15.72%	0.15%	0.08%	Glycolysis to A-CoA	Gck, Hk2, Mpc1, Pdha1, Pfkm
			MP:0003729	abnormal photoreceptor outer segment morphology	1.41E-01	2.54E-01	1	18	179	21319	5.56%	0.84%	0.56%	0.08%	Glycolysis to A-CoA	Mpc1
			MP:0009944	abnormal olfactory lobe morphology	1.42E-01	2.56E-01	1	18	181	21319	5.56%	0.85%	0.55%	0.08%	Glycolysis to A-CoA	Pdha1
			MP:0020184	abnormal susceptibility to parasitic infection	1.43E-01	2.57E-01	1	18	182	21319	5.56%	0.85%	0.55%	0.08%	Glycolysis to A-CoA	Pklr
			MP:0002127	abnormal cardiovascular system morphology	1.45E-01	2.60E-01	5	18	3391	21319	27.78%	15.91%	0.15%	0.08%	Glycolysis to A-CoA	Gck, Pdha1, Pdhb, Pdhx, Pfkm
			MP:0000639	abnormal adrenal gland morphology	1.46E-01	2.60E-01	1	18	186	21319	5.56%	0.87%	0.54%	0.08%	Glycolysis to A-CoA	Hk3
			MP:0001473	reduced long term potentiation	1.47E-01	2.62E-01	1	18	188	21319	5.56%	0.88%	0.53%	0.08%	Glycolysis to A-CoA	Vdac1
			MP:0002199	abnormal brain commissure morphology	1.50E-01	2.65E-01	1	18	191	21319	5.56%	0.90%	0.52%	0.08%	Glycolysis to A-CoA	Pdha1
			MP:0011965	decreased total retina thickness	1.50E-01	2.65E-01	1	18	192	21319	5.56%	0.90%	0.52%	0.08%	Glycolysis to A-CoA	Pdhx
			MP:0010630	abnormal cardiac muscle tissue morphology	1.51E-01	2.65E-01	2	18	820	21319	11.11%	3.85%	0.24%	0.08%	Glycolysis to A-CoA	Pdha1, Pfkm
			MP:0005281	increased fatty acids level	1.51E-01	2.65E-01	1	18	193	21319	5.56%	0.91%	0.52%	0.08%	Glycolysis to A-CoA	Gck
			MP:0005217	abnormal pancreatic beta cell morphology	1.53E-01	2.65E-01	1	18	195	21319	5.56%	0.91%	0.51%	0.08%	Glycolysis to A-CoA	Gck
			MP:0001559	hyperglycemia	1.53E-01	2.65E-01	1	18	195	21319	5.56%	0.91%	0.51%	0.08%	Glycolysis to A-CoA	Gck
			MP:0009787	increased susceptibility to infection induced morbidity/mortality	1.53E-01	2.65E-01	1	18	195	21319	5.56%	0.91%	0.51%	0.08%	Glycolysis to A-CoA	Pklr
			MP:0013278	decreased fasting circulating glucose level	1.53E-01	2.66E-01	1	18	196	21319	5.56%	0.92%	0.51%	0.08%	Glycolysis to A-CoA	Gck
			MP:0031094	organomegaly	1.54E-01	2.66E-01	4	18	2512	21319	22.22%	11.78%	0.16%	0.08%	Glycolysis to A-CoA	Hk1, Pdha1, Pfkm, Pklr
			MP:0001931	abnormal oogenesis	1.58E-01	2.71E-01	1	18	203	21319	5.56%	0.95%	0.49%	0.08%	Glycolysis to A-CoA	Pdha1
			MP:0001614	abnormal blood vessel morphology	1.59E-01	2.71E-01	3	18	1650	21319	16.67%	7.74%	0.18%	0.08%	Glycolysis to A-CoA	Pdhb, Pdhx, Pfkm
			MP:0014134	abnormal embryo morphology	1.59E-01	2.71E-01	3	18	1651	21319	16.67%	7.74%	0.18%	0.08%	Glycolysis to A-CoA	Dld, Pdha1, Pdhb
			MP:0012775	abnormal brain weight	1.59E-01	2.71E-01	1	18	204	21319	5.56%	0.96%	0.49%	0.08%	Glycolysis to A-CoA	Pdha1
			MP:0001688	abnormal somite development	1.61E-01	2.74E-01	1	18	207	21319	5.56%	0.97%	0.48%	0.08%	Glycolysis to A-CoA	Pdhb
			MP:0011963	abnormal total retina thickness	1.62E-01	2.75E-01	1	18	208	21319	5.56%	0.98%	0.48%	0.08%	Glycolysis to A-CoA	Pdhx
			MP:0006108	abnormal hindbrain development	1.64E-01	2.77E-01	1	18	211	21319	5.56%	0.99%	0.47%	0.08%	Glycolysis to A-CoA	Hk2
			MP:0002060	abnormal skin morphology	1.66E-01	2.81E-01	3	18	1688	21319	16.67%	7.92%	0.18%	0.08%	Glycolysis to A-CoA	Hk1, Hk3, Pfkm
			MP:0005562	decreased mean corpuscular hemoglobin	1.70E-01	2.87E-01	1	18	220	21319	5.56%	1.03%	0.45%	0.08%	Glycolysis to A-CoA	Hk1
			MP:0000913	abnormal brain development	1.77E-01	2.97E-01	2	18	907	21319	11.11%	4.25%	0.22%	0.08%	Glycolysis to A-CoA	Hk2, Pdhb
			MP:0000778	abnormal nervous system tract morphology	1.77E-01	2.97E-01	1	18	230	21319	5.56%	1.08%	0.43%	0.08%	Glycolysis to A-CoA	Pdha1
			MP:0005288	abnormal oxygen consumption	1.79E-01	2.98E-01	1	18	232	21319	5.56%	1.09%	0.43%	0.08%	Glycolysis to A-CoA	Mpc2
			MP:0001700	abnormal embryo turning	1.81E-01	3.01E-01	1	18	235	21319	5.56%	1.10%	0.43%	0.08%	Glycolysis to A-CoA	Pdhb
			MP:0002891	increased insulin sensitivity	1.83E-01	3.03E-01	1	18	238	21319	5.56%	1.12%	0.42%	0.08%	Glycolysis to A-CoA	Mpc1
			MP:0005378	growth/size/body region phenotype	1.84E-01	3.04E-01	9	18	7894	21319	50.00%	37.03%	0.11%	0.08%	Glycolysis to A-CoA	Dld, Gck, Hk1, Hk2, Mpc1, Pdha1, Pdhb, Pfkm, Pklr
			MP:0002089	abnormal postnatal growth/weight/body size	1.85E-01	3.05E-01	6	18	4678	21319	33.33%	21.94%	0.13%	0.08%	Glycolysis to A-CoA	Gck, Hk1, Hk2, Mpc1, Pdha1, Pfkm
			MP:0003232	abnormal forebrain development	1.87E-01	3.07E-01	1	18	244	21319	5.56%	1.14%	0.41%	0.08%	Glycolysis to A-CoA	Pdhb
			MP:0012778	abnormal liver triglyceride level	1.87E-01	3.07E-01	1	18	244	21319	5.56%	1.14%	0.41%	0.08%	Glycolysis to A-CoA	Hkdc1
			MP:0003699	abnormal female reproductive system physiology	1.88E-01	3.08E-01	2	18	944	21319	11.11%	4.43%	0.21%	0.08%	Glycolysis to A-CoA	Hk1, Pdha1
			MP:0003717	pallor	1.89E-01	3.08E-01	1	18	247	21319	5.56%	1.16%	0.40%	0.08%	Glycolysis to A-CoA	Hk1
			MP:0008026	abnormal brain white matter morphology	1.90E-01	3.08E-01	1	18	248	21319	5.56%	1.16%	0.40%	0.08%	Glycolysis to A-CoA	Pdha1
			MP:0013279	increased fasting circulating glucose level	1.96E-01	3.16E-01	1	18	256	21319	5.56%	1.20%	0.39%	0.08%	Glycolysis to A-CoA	Gck
			MP:0005331	insulin resistance	1.96E-01	3.16E-01	1	18	256	21319	5.56%	1.20%	0.39%	0.08%	Glycolysis to A-CoA	Gck
			MP:0010067	increased red blood cell distribution width	1.97E-01	3.17E-01	1	18	258	21319	5.56%	1.21%	0.39%	0.08%	Glycolysis to A-CoA	Hk1
			MP:0001553	abnormal circulating free fatty acids level	1.99E-01	3.19E-01	1	18	261	21319	5.56%	1.22%	0.38%	0.08%	Glycolysis to A-CoA	Gck
			MP:0000180	abnormal circulating cholesterol level	1.99E-01	3.19E-01	2	18	979	21319	11.11%	4.59%	0.20%	0.08%	Glycolysis to A-CoA	Gck, Mpc1
			MP:0001004	abnormal retina photoreceptor morphology	2.03E-01	3.24E-01	1	18	267	21319	5.56%	1.25%	0.37%	0.08%	Glycolysis to A-CoA	Mpc1
			MP:0000192	abnormal mineral level	2.06E-01	3.26E-01	2	18	1001	21319	11.11%	4.70%	0.20%	0.08%	Glycolysis to A-CoA	Hk1, Pklr
			MP:0006035	abnormal mitochondrial morphology	2.07E-01	3.26E-01	1	18	273	21319	5.56%	1.28%	0.37%	0.08%	Glycolysis to A-CoA	Gck
			MP:0010680	abnormal skin adnexa physiology	2.07E-01	3.26E-01	1	18	273	21319	5.56%	1.28%	0.37%	0.08%	Glycolysis to A-CoA	Pfkl
			MP:0000377	abnormal hair follicle morphology	2.08E-01	3.26E-01	1	18	274	21319	5.56%	1.29%	0.36%	0.08%	Glycolysis to A-CoA	Pfkl
			MP:0002699	abnormal vitreous body morphology	2.08E-01	3.26E-01	1	18	274	21319	5.56%	1.29%	0.36%	0.08%	Glycolysis to A-CoA	Pdhx

			MP:0002059	abnormal seminal vesicle morphology	2.08E-01	3.26E-01	1	18	275	21319	5.56%	1.29%	0.36%	0.08%	Glycolysis to A-CoA	Pfkm
			MP:0009785	abnormal susceptibility to infection induced morbidity/mortality	2.08E-01	3.26E-01	1	18	275	21319	5.56%	1.29%	0.36%	0.08%	Glycolysis to A-CoA	Pklr
			MP:0005636	abnormal mineral homeostasis	2.09E-01	3.26E-01	2	18	1011	21319	11.11%	4.74%	0.20%	0.08%	Glycolysis to A-CoA	Hk1, Pklr
			MP:0000877	abnormal Purkinje cell morphology	2.11E-01	3.29E-01	1	18	279	21319	5.56%	1.31%	0.36%	0.08%	Glycolysis to A-CoA	Pdha1
			MP:0010358	abnormal free fatty acids level	2.13E-01	3.31E-01	1	18	282	21319	5.56%	1.32%	0.35%	0.08%	Glycolysis to A-CoA	Gck
			MP:0001488	increased startle reflex	2.14E-01	3.31E-01	1	18	283	21319	5.56%	1.33%	0.35%	0.08%	Glycolysis to A-CoA	Vdac1
			MP:0005551	abnormal eye electrophysiology	2.15E-01	3.32E-01	1	18	285	21319	5.56%	1.34%	0.35%	0.08%	Glycolysis to A-CoA	Mpc1
			MP:0002207	abnormal long term potentiation	2.18E-01	3.35E-01	1	18	289	21319	5.56%	1.36%	0.35%	0.08%	Glycolysis to A-CoA	Vdac1
			MP:0010066	abnormal red blood cell distribution width	2.19E-01	3.37E-01	1	18	291	21319	5.56%	1.36%	0.34%	0.08%	Glycolysis to A-CoA	Hk1
			MP:0003947	abnormal cholesterol level	2.21E-01	3.38E-01	2	18	1048	21319	11.11%	4.92%	0.19%	0.08%	Glycolysis to A-CoA	Gck, Mpc1
			MP:0012321	abnormal total tissue mass	2.22E-01	3.39E-01	4	18	2898	21319	22.22%	13.59%	0.14%	0.08%	Glycolysis to A-CoA	Gck, Hk2, Mpc1, Pdha1
			MP:0001574	abnormal oxygen level	2.24E-01	3.40E-01	1	18	298	21319	5.56%	1.40%	0.34%	0.08%	Glycolysis to A-CoA	Pfkm
			MP:0003084	abnormal skeletal muscle fiber morphology	2.24E-01	3.40E-01	1	18	298	21319	5.56%	1.40%	0.34%	0.08%	Glycolysis to A-CoA	Pfkm
			MP:0002628	hepatic steatosis	2.25E-01	3.41E-01	1	18	300	21319	5.56%	1.41%	0.33%	0.08%	Glycolysis to A-CoA	Gck
			MP:0012224	abnormal sterol level	2.27E-01	3.43E-01	2	18	1067	21319	11.11%	5.00%	0.19%	0.08%	Glycolysis to A-CoA	Gck, Mpc1
			MP:0000875	abnormal cerebellar Purkinje cell layer	2.29E-01	3.43E-01	1	18	305	21319	5.56%	1.43%	0.33%	0.08%	Glycolysis to A-CoA	Pdha1
			MP:0000316	cellular necrosis	2.29E-01	3.43E-01	1	18	305	21319	5.56%	1.43%	0.33%	0.08%	Glycolysis to A-CoA	Pfkm
			MP:0011096	embryonic lethality between implantation and somite formation, complete penetrance	2.30E-01	3.44E-01	1	18	307	21319	5.56%	1.44%	0.33%	0.08%	Glycolysis to A-CoA	Dld
			MP:0011181	increased hematopoietic cell number	2.32E-01	3.47E-01	3	18	1986	21319	16.67%	9.32%	0.15%	0.08%	Glycolysis to A-CoA	Hk1, Pfkm, Pklr
			MP:0003141	cardiac fibrosis	2.36E-01	3.50E-01	1	18	316	21319	5.56%	1.48%	0.32%	0.08%	Glycolysis to A-CoA	Gck
			MP:0002412	increased susceptibility to bacterial infection	2.36E-01	3.50E-01	1	18	316	21319	5.56%	1.48%	0.32%	0.08%	Glycolysis to A-CoA	Pklr
			MP:0005215	abnormal pancreatic islet morphology	2.36E-01	3.50E-01	1	18	317	21319	5.56%	1.49%	0.32%	0.08%	Glycolysis to A-CoA	Gck
			MP:0005278	abnormal cholesterol homeostasis	2.37E-01	3.50E-01	2	18	1098	21319	11.11%	5.15%	0.18%	0.08%	Glycolysis to A-CoA	Gck, Mpc1
			MP:0009165	abnormal endocrine pancreas morphology	2.37E-01	3.50E-01	1	18	318	21319	5.56%	1.49%	0.31%	0.08%	Glycolysis to A-CoA	Gck
			MP:0013658	abnormal myeloid cell morphology	2.38E-01	3.50E-01	4	18	2984	21319	22.22%	14.00%	0.13%	0.08%	Glycolysis to A-CoA	Gck, Hk1, Pfkm, Pklr
			MP:0001930	abnormal meiosis	2.38E-01	3.50E-01	1	18	320	21319	5.56%	1.50%	0.31%	0.08%	Glycolysis to A-CoA	Pdha1
			MP:0002409	decreased susceptibility to infection	2.40E-01	3.52E-01	1	18	323	21319	5.56%	1.52%	0.31%	0.08%	Glycolysis to A-CoA	Pklr
			MP:0003960	increased lean body mass	2.44E-01	3.54E-01	1	18	328	21319	5.56%	1.54%	0.30%	0.08%	Glycolysis to A-CoA	Pfkm
			MP:0000601	small liver	2.44E-01	3.54E-01	1	18	328	21319	5.56%	1.54%	0.30%	0.08%	Glycolysis to A-CoA	Pfkm
			MP:0000685	abnormal immune system morphology	2.44E-01	3.54E-01	5	18	4035	21319	27.78%	18.93%	0.12%	0.08%	Glycolysis to A-CoA	Hk1, Hk3, Pdha2, Pfkm, Pklr
			MP:0006205	embryonic lethality between implantation and somite formation	2.46E-01	3.56E-01	1	18	331	21319	5.56%	1.55%	0.30%	0.08%	Glycolysis to A-CoA	Dld
			MP:0005294	abnormal heart ventricle morphology	2.46E-01	3.56E-01	2	18	1129	21319	11.11%	5.30%	0.18%	0.08%	Glycolysis to A-CoA	Pdha1, Pfkm
			MP:0001552	increased circulating triglyceride level	2.48E-01	3.58E-01	1	18	335	21319	5.56%	1.57%	0.30%	0.08%	Glycolysis to A-CoA	Gck
			MP:0005193	abnormal anterior eye segment morphology	2.50E-01	3.59E-01	2	18	1140	21319	11.11%	5.35%	0.18%	0.08%	Glycolysis to A-CoA	Hk3, Pdhx
			MP:0000830	abnormal diencephalon morphology	2.50E-01	3.59E-01	1	18	338	21319	5.56%	1.59%	0.30%	0.08%	Glycolysis to A-CoA	Pdha1
			MP:0001730	embryonic growth arrest	2.51E-01	3.60E-01	1	18	340	21319	5.56%	1.59%	0.29%	0.08%	Glycolysis to A-CoA	Dld
			MP:0013330	abnormal male accessory sex gland morphology	2.53E-01	3.61E-01	1	18	343	21319	5.56%	1.61%	0.29%	0.08%	Glycolysis to A-CoA	Pfkm
			MP:0005280	abnormal fatty acids level	2.57E-01	3.66E-01	1	18	349	21319	5.56%	1.64%	0.29%	0.08%	Glycolysis to A-CoA	Gck
			MP:0002123	abnormal definitive hematopoiesis	2.60E-01	3.68E-01	2	18	1170	21319	11.11%	5.49%	0.17%	0.08%	Glycolysis to A-CoA	Hk1, Pklr
			MP:0001463	abnormal spatial learning	2.60E-01	3.68E-01	1	18	353	21319	5.56%	1.66%	0.28%	0.08%	Glycolysis to A-CoA	Vdac1
			MP:0001765	abnormal ion homeostasis	2.60E-01	3.68E-01	2	18	1173	21319	11.11%	5.50%	0.17%	0.08%	Glycolysis to A-CoA	Hk1, Pklr
			MP:0030572	abnormal pilosebaceous unit morphology	2.62E-01	3.68E-01	1	18	356	21319	5.56%	1.67%	0.28%	0.08%	Glycolysis to A-CoA	Pfkl
			MP:0001259	abnormal body weight	2.63E-01	3.68E-01	4	18	3112	21319	22.22%	14.60%	0.13%	0.08%	Glycolysis to A-CoA	Gck, Hk2, Mpc1, Pdha1
			MP:0010771	integument phenotype	2.63E-01	3.68E-01	4	18	3112	21319	22.22%	14.60%	0.13%	0.08%	Glycolysis to A-CoA	Hk1, Hk3, Pfkl, Pfkm
			MP:0005292	improved glucose tolerance	2.63E-01	3.68E-01	1	18	359	21319	5.56%	1.68%	0.28%	0.08%	Glycolysis to A-CoA	Mpc1
			MP:0011088	neonatal lethality, incomplete penetrance	2.63E-01	3.68E-01	1	18	359	21319	5.56%	1.68%	0.28%	0.08%	Glycolysis to A-CoA	Gck
			MP:0013657	abnormal blood cell morphology	2.64E-01	3.68E-01	4	18	3119	21319	22.22%	14.63%	0.13%	0.08%	Glycolysis to A-CoA	Gck, Hk1, Pfkm, Pklr
			MP:0013693	abnormal hemopoiesis	2.68E-01	3.72E-01	2	18	1197	21319	11.11%	5.61%	0.17%	0.08%	Glycolysis to A-CoA	Hk1, Pklr
			MP:0009956	abnormal cerebellar layer morphology	2.72E-01	3.76E-01	1	18	372	21319	5.56%	1.74%	0.27%	0.08%	Glycolysis to A-CoA	Pdha1
			MP:0000774	decreased brain size	2.72E-01	3.76E-01	1	18	373	21319	5.56%	1.75%	0.27%	0.08%	Glycolysis to A-CoA	Pdha1
			MP:0010508	abnormal heart electrocardiography waveform feature	2.75E-01	3.80E-01	1	18	378	21319	5.56%	1.77%	0.26%	0.08%	Glycolysis to A-CoA	Gck

			MP:0000278	abnormal myocardial fiber morphology	2.88E-01	3.96E-01	1	18	398	21319	5.56%	1.87%	0.25%	0.08%	Glycolysis to A-CoA	Pdha1
			MP:0003137	abnormal impulse conducting system conduction	2.96E-01	4.05E-01	1	18	411	21319	5.56%	1.93%	0.24%	0.08%	Glycolysis to A-CoA	Gck
			MP:0002063	abnormal learning/memory/conditioning	2.97E-01	4.05E-01	2	18	1286	21319	11.11%	6.03%	0.16%	0.08%	Glycolysis to A-CoA	Dlat, Vdac1
			MP:0020186	abnormal susceptibility to bacterial infection	2.97E-01	4.05E-01	1	18	413	21319	5.56%	1.94%	0.24%	0.08%	Glycolysis to A-CoA	Pklr
			MP:0000260	abnormal angiogenesis	3.00E-01	4.07E-01	1	18	418	21319	5.56%	1.96%	0.24%	0.08%	Glycolysis to A-CoA	Pfkm
			MP:0004097	abnormal cerebellar cortex morphology	3.00E-01	4.07E-01	1	18	418	21319	5.56%	1.96%	0.24%	0.08%	Glycolysis to A-CoA	Pdha1
			MP:0014114	abnormal cognition	3.00E-01	4.07E-01	2	18	1298	21319	11.11%	6.09%	0.15%	0.08%	Glycolysis to A-CoA	Dlat, Vdac1
			MP:0000274	enlarged heart	3.02E-01	4.09E-01	2	18	1304	21319	11.11%	6.12%	0.15%	0.08%	Glycolysis to A-CoA	Pdha1, Pfkm
			MP:0005179	decreased circulating cholesterol level	3.04E-01	4.10E-01	1	18	425	21319	5.56%	1.99%	0.24%	0.08%	Glycolysis to A-CoA	Mpc1
			MP:0011101	prenatal lethality, incomplete penetrance	3.05E-01	4.10E-01	1	18	426	21319	5.56%	2.00%	0.23%	0.08%	Glycolysis to A-CoA	Pdha1
			MP:0001695	abnormal gastrulation	3.07E-01	4.11E-01	1	18	429	21319	5.56%	2.01%	0.23%	0.08%	Glycolysis to A-CoA	Pdhb
			MP:0001417	decreased exploration in new environment	3.07E-01	4.11E-01	1	18	430	21319	5.56%	2.02%	0.23%	0.08%	Glycolysis to A-CoA	Dlat
			MP:0013560	abnormal endocrine gland morphology	3.08E-01	4.11E-01	2	18	1322	21319	11.11%	6.20%	0.15%	0.08%	Glycolysis to A-CoA	Gck, Hk3
			MP:0001924	infertility	3.08E-01	4.11E-01	2	18	1322	21319	11.11%	6.20%	0.15%	0.08%	Glycolysis to A-CoA	Hk1, Pdha1
			MP:0002152	abnormal brain morphology	3.12E-01	4.15E-01	3	18	2323	21319	16.67%	10.90%	0.13%	0.08%	Glycolysis to A-CoA	Hk2, Pdha1, Pdhb
			MP:0005384	cellular phenotype	3.14E-01	4.16E-01	6	18	5521	21319	33.33%	25.90%	0.11%	0.08%	Glycolysis to A-CoA	Gck, Hk2, Mpc1, Mpc2, Pdha1, Pfkm
			MP:0013277	abnormal fasting circulating glucose level	3.18E-01	4.20E-01	1	18	448	21319	5.56%	2.10%	0.22%	0.08%	Glycolysis to A-CoA	Gck
			MP:0002062	abnormal associative learning	3.19E-01	4.20E-01	1	18	450	21319	5.56%	2.11%	0.22%	0.08%	Glycolysis to A-CoA	Vdac1
			MP:0000783	abnormal forebrain morphology	3.21E-01	4.20E-01	2	18	1361	21319	11.11%	6.38%	0.15%	0.08%	Glycolysis to A-CoA	Pdha1, Pdhb
			MP:0005140	decreased cardiac muscle contractility	3.21E-01	4.20E-01	1	18	453	21319	5.56%	2.12%	0.22%	0.08%	Glycolysis to A-CoA	Pdha1
			MP:0003983	decreased cholesterol level	3.22E-01	4.20E-01	1	18	455	21319	5.56%	2.13%	0.22%	0.08%	Glycolysis to A-CoA	Mpc1
			MP:0012225	decreased sterol level	3.22E-01	4.20E-01	1	18	455	21319	5.56%	2.13%	0.22%	0.08%	Glycolysis to A-CoA	Mpc1
			MP:0002429	abnormal blood cell morphology/development	3.22E-01	4.20E-01	2	18	1366	21319	11.11%	6.41%	0.15%	0.08%	Glycolysis to A-CoA	Hk1, Pklr
			MP:0005385	cardiovascular system phenotype	3.23E-01	4.20E-01	5	18	4481	21319	27.78%	21.02%	0.11%	0.08%	Glycolysis to A-CoA	Gck, Pdha1, Pdhb, Pdhx, Pfkm
			MP:0000788	abnormal cerebral cortex morphology	3.24E-01	4.20E-01	1	18	458	21319	5.56%	2.15%	0.22%	0.08%	Glycolysis to A-CoA	Pdha1
			MP:0005319	abnormal enzyme/coenzyme level	3.26E-01	4.23E-01	2	18	1379	21319	11.11%	6.47%	0.15%	0.08%	Glycolysis to A-CoA	Gck, Pfkm
			MP:0001589	abnormal mean corpuscular hemoglobin	3.29E-01	4.26E-01	1	18	468	21319	5.56%	2.20%	0.21%	0.08%	Glycolysis to A-CoA	Hk1
			MP:0001262	decreased body weight	3.32E-01	4.28E-01	3	18	2407	21319	16.67%	11.29%	0.12%	0.08%	Glycolysis to A-CoA	Gck, Hk2, Mpc1
			MP:0012322	decreased total tissue mass	3.33E-01	4.28E-01	3	18	2408	21319	16.67%	11.30%	0.12%	0.08%	Glycolysis to A-CoA	Gck, Hk2, Mpc1
			MP:0000771	abnormal brain size	3.33E-01	4.28E-01	1	18	474	21319	5.56%	2.22%	0.21%	0.08%	Glycolysis to A-CoA	Pdha1
			MP:0003935	abnormal craniofacial development	3.34E-01	4.28E-01	1	18	476	21319	5.56%	2.23%	0.21%	0.08%	Glycolysis to A-CoA	Pdhb
			MP:0001286	abnormal eye development	3.35E-01	4.29E-01	1	18	478	21319	5.56%	2.24%	0.21%	0.08%	Glycolysis to A-CoA	Pdhx
			MP:0002106	abnormal muscle physiology	3.37E-01	4.29E-01	2	18	1412	21319	11.11%	6.62%	0.14%	0.08%	Glycolysis to A-CoA	Pdha1, Pfkm
			MP:0001944	abnormal pancreas morphology	3.37E-01	4.29E-01	1	18	481	21319	5.56%	2.26%	0.21%	0.08%	Glycolysis to A-CoA	Gck
			MP:0010025	decreased total body fat amount	3.39E-01	4.30E-01	1	18	484	21319	5.56%	2.27%	0.21%	0.08%	Glycolysis to A-CoA	Pfkm
			MP:0000702	enlarged lymph nodes	3.40E-01	4.30E-01	1	18	486	21319	5.56%	2.28%	0.21%	0.08%	Glycolysis to A-CoA	Hk3
			MP:0011092	embryonic lethality, complete penetrance	3.41E-01	4.31E-01	1	18	488	21319	5.56%	2.29%	0.20%	0.08%	Glycolysis to A-CoA	Pdha1
			MP:0002968	increased circulating alkaline phosphatase level	3.42E-01	4.31E-01	1	18	489	21319	5.56%	2.29%	0.20%	0.08%	Glycolysis to A-CoA	Gck
			MP:0003861	abnormal nervous system development	3.46E-01	4.35E-01	2	18	1441	21319	11.11%	6.76%	0.14%	0.08%	Glycolysis to A-CoA	Hk2, Pdhb
			MP:0001258	decreased body length	3.48E-01	4.35E-01	1	18	500	21319	5.56%	2.35%	0.20%	0.08%	Glycolysis to A-CoA	Pfkm
			MP:0002169	no abnormal phenotype detected	3.48E-01	4.35E-01	3	18	2471	21319	16.67%	11.59%	0.12%	0.08%	Glycolysis to A-CoA	Hk2, Pklr, Pkm
			MP:0000952	abnormal CNS glial cell morphology	3.48E-01	4.35E-01	1	18	501	21319	5.56%	2.35%	0.20%	0.08%	Glycolysis to A-CoA	Mpc1
			MP:0002873	normal phenotype	3.49E-01	4.35E-01	3	18	2474	21319	16.67%	11.60%	0.12%	0.08%	Glycolysis to A-CoA	Hk2, Pklr, Pkm
			MP:0005379	endocrine/exocrine gland phenotype	3.63E-01	4.52E-01	4	18	3607	21319	22.22%	16.92%	0.11%	0.08%	Glycolysis to A-CoA	Gck, Hk3, Mpc2, Pfkm
			MP:0002084	abnormal developmental patterning	3.69E-01	4.58E-01	1	18	539	21319	5.56%	2.53%	0.19%	0.08%	Glycolysis to A-CoA	Pdhb
			MP:0008948	decreased neuron number	3.69E-01	4.58E-01	1	18	539	21319	5.56%	2.53%	0.19%	0.08%	Glycolysis to A-CoA	Pdha1
			MP:0000738	impaired muscle contractility	3.71E-01	4.58E-01	1	18	541	21319	5.56%	2.54%	0.18%	0.08%	Glycolysis to A-CoA	Pdha1
			MP:0000266	abnormal heart morphology	3.74E-01	4.61E-01	3	18	2578	21319	16.67%	12.09%	0.12%	0.08%	Glycolysis to A-CoA	Gck, Pdha1, Pfkm
			MP:0000351	increased cell proliferation	3.76E-01	4.62E-01	1	18	552	21319	5.56%	2.59%	0.18%	0.08%	Glycolysis to A-CoA	Gck
			MP:0002972	abnormal cardiac muscle contractility	3.77E-01	4.62E-01	1	18	553	21319	5.56%	2.59%	0.18%	0.08%	Glycolysis to A-CoA	Pdha1
			MP:0000701	abnormal lymph node size	3.77E-01	4.62E-01	1	18	553	21319	5.56%	2.59%	0.18%	0.08%	Glycolysis to A-CoA	Hk3

			MP:0013189	abnormal exploration in a new environment	3.79E-01	4.63E-01	1	18	556	21319	5.56%	2.61%	0.18%	0.08%	Glycolysis to A-CoA	Dlat
			MP:0003091	abnormal cell migration	3.83E-01	4.67E-01	1	18	564	21319	5.56%	2.65%	0.18%	0.08%	Glycolysis to A-CoA	Hk2
			MP:0005621	abnormal cell physiology	3.87E-01	4.71E-01	5	18	4830	21319	27.78%	22.66%	0.10%	0.08%	Glycolysis to A-CoA	Gck, Hk2, Mpc1, Mpc2, Pfkm
			MP:0000350	abnormal cell proliferation	3.88E-01	4.71E-01	2	18	1574	21319	11.11%	7.38%	0.13%	0.08%	Glycolysis to A-CoA	Gck, Hk2
			MP:0000259	abnormal vascular development	3.88E-01	4.71E-01	1	18	574	21319	5.56%	2.69%	0.17%	0.08%	Glycolysis to A-CoA	Pfkm
			MP:0001256	abnormal body length	3.91E-01	4.73E-01	1	18	579	21319	5.56%	2.72%	0.17%	0.08%	Glycolysis to A-CoA	Pfkm
			MP:0002406	increased susceptibility to infection	3.94E-01	4.75E-01	1	18	584	21319	5.56%	2.74%	0.17%	0.08%	Glycolysis to A-CoA	Pklr
			MP:0011085	postnatal lethality, complete penetrance	3.95E-01	4.75E-01	1	18	586	21319	5.56%	2.75%	0.17%	0.08%	Glycolysis to A-CoA	Gck
			MP:0009643	abnormal urine homeostasis	3.96E-01	4.76E-01	1	18	589	21319	5.56%	2.76%	0.17%	0.08%	Glycolysis to A-CoA	Gck
			MP:0005406	abnormal heart size	3.97E-01	4.76E-01	2	18	1603	21319	11.11%	7.52%	0.12%	0.08%	Glycolysis to A-CoA	Pdha1, Pfkm
			MP:0001486	abnormal startle reflex	3.97E-01	4.76E-01	1	18	591	21319	5.56%	2.77%	0.17%	0.08%	Glycolysis to A-CoA	Vdac1
			MP:0002151	abnormal neural tube morphology	4.02E-01	4.80E-01	1	18	600	21319	5.56%	2.81%	0.17%	0.08%	Glycolysis to A-CoA	Pdhb
			MP:0000202	abnormal circulating alkaline phosphatase level	4.03E-01	4.81E-01	1	18	603	21319	5.56%	2.83%	0.17%	0.08%	Glycolysis to A-CoA	Gck
			MP:0000965	abnormal sensory neuron morphology	4.06E-01	4.83E-01	1	18	608	21319	5.56%	2.85%	0.16%	0.08%	Glycolysis to A-CoA	Mpc1
			MP:0001413	abnormal response to new environment	4.10E-01	4.86E-01	1	18	615	21319	5.56%	2.88%	0.16%	0.08%	Glycolysis to A-CoA	Dlat
			MP:0002108	abnormal muscle morphology	4.17E-01	4.93E-01	2	18	1669	21319	11.11%	7.83%	0.12%	0.08%	Glycolysis to A-CoA	Pdha1, Pfkm
			MP:0003634	abnormal glial cell morphology	4.17E-01	4.93E-01	1	18	630	21319	5.56%	2.96%	0.16%	0.08%	Glycolysis to A-CoA	Mpc1
			MP:0002396	abnormal hematopoietic system morphology/development	4.23E-01	4.99E-01	5	18	5018	21319	27.78%	23.54%	0.10%	0.08%	Glycolysis to A-CoA	Gck, Hk1, Pdha2, Pfkm, Pklr
			MP:0009931	abnormal skin appearance	4.25E-01	5.00E-01	1	18	645	21319	5.56%	3.03%	0.16%	0.08%	Glycolysis to A-CoA	Hk1
			MP:0008946	abnormal neuron number	4.29E-01	5.03E-01	1	18	653	21319	5.56%	3.06%	0.15%	0.08%	Glycolysis to A-CoA	Pdha1
			MP:0000759	abnormal skeletal muscle morphology	4.29E-01	5.03E-01	1	18	653	21319	5.56%	3.06%	0.15%	0.08%	Glycolysis to A-CoA	Pfkm
			MP:0003632	abnormal nervous system morphology	4.34E-01	5.07E-01	4	18	3947	21319	22.22%	18.51%	0.10%	0.08%	Glycolysis to A-CoA	Hk2, Mpc1, Pdha1, Pdhb
			MP:0003959	abnormal lean body mass	4.34E-01	5.07E-01	1	18	664	21319	5.56%	3.11%	0.15%	0.08%	Glycolysis to A-CoA	Pfkm
			MP:0005329	abnormal myocardium layer morphology	4.40E-01	5.13E-01	1	18	676	21319	5.56%	3.17%	0.15%	0.08%	Glycolysis to A-CoA	Pdha1
			MP:0004848	abnormal liver size	4.44E-01	5.16E-01	1	18	684	21319	5.56%	3.21%	0.15%	0.08%	Glycolysis to A-CoA	Pfkm
			MP:0010545	abnormal heart layer morphology	4.45E-01	5.16E-01	1	18	686	21319	5.56%	3.22%	0.15%	0.08%	Glycolysis to A-CoA	Pdha1
			MP:0002339	abnormal lymph node morphology	4.48E-01	5.18E-01	1	18	691	21319	5.56%	3.24%	0.14%	0.08%	Glycolysis to A-CoA	Hk3
			MP:0005620	abnormal muscle contractility	4.49E-01	5.19E-01	1	18	695	21319	5.56%	3.26%	0.14%	0.08%	Glycolysis to A-CoA	Pdha1
			MP:0002083	premature death	4.53E-01	5.23E-01	2	18	1789	21319	11.11%	8.39%	0.11%	0.08%	Glycolysis to A-CoA	Pdha1, Pfkm
			MP:0003631	nervous system phenotype	4.65E-01	5.34E-01	5	18	5244	21319	27.78%	24.60%	0.10%	0.08%	Glycolysis to A-CoA	Hk2, Mpc1, Pdha1, Pdhb, Vdac1
			MP:0011098	embryonic lethality during organogenesis, complete penetrance	4.66E-01	5.34E-01	1	18	730	21319	5.56%	3.42%	0.14%	0.08%	Glycolysis to A-CoA	Mpc2
			MP:0000358	abnormal cell morphology	4.66E-01	5.34E-01	2	18	1834	21319	11.11%	8.60%	0.11%	0.08%	Glycolysis to A-CoA	Gck, Pdha1
			MP:0003107	abnormal response to novelty	4.66E-01	5.34E-01	1	18	731	21319	5.56%	3.43%	0.14%	0.08%	Glycolysis to A-CoA	Dlat
			MP:0011180	abnormal hematopoietic cell number	4.76E-01	5.44E-01	3	18	3006	21319	16.67%	14.10%	0.10%	0.08%	Glycolysis to A-CoA	Hk1, Pfkm, Pklr
			MP:0004811	abnormal neuron physiology	4.78E-01	5.44E-01	1	18	755	21319	5.56%	3.54%	0.13%	0.08%	Glycolysis to A-CoA	Hk2
			MP:0002398	abnormal bone marrow cell morphology/development	4.86E-01	5.53E-01	1	18	774	21319	5.56%	3.63%	0.13%	0.08%	Glycolysis to A-CoA	Pklr
			MP:0013656	abnormal hematopoietic cell morphology	4.93E-01	5.60E-01	4	18	4236	21319	22.22%	19.87%	0.09%	0.08%	Glycolysis to A-CoA	Gck, Hk1, Pfkm, Pklr
			MP:0001657	abnormal induced morbidity/mortality	4.95E-01	5.61E-01	1	18	794	21319	5.56%	3.72%	0.13%	0.08%	Glycolysis to A-CoA	Pklr
			MP:0012382	abnormal blood cell physiology	4.98E-01	5.63E-01	2	18	1945	21319	11.11%	9.12%	0.10%	0.08%	Glycolysis to A-CoA	Pfkm, Pklr
			MP:0001793	abnormal susceptibility to infection	5.01E-01	5.65E-01	1	18	807	21319	5.56%	3.79%	0.12%	0.08%	Glycolysis to A-CoA	Pklr
			MP:0011182	decreased hematopoietic cell number	5.10E-01	5.73E-01	2	18	1985	21319	11.11%	9.31%	0.10%	0.08%	Glycolysis to A-CoA	Hk1, Pklr
			MP:0008540	abnormal cerebral hemisphere morphology	5.10E-01	5.73E-01	1	18	829	21319	5.56%	3.89%	0.12%	0.08%	Glycolysis to A-CoA	Pdha1
			MP:0005025	abnormal response to infection	5.19E-01	5.82E-01	1	18	849	21319	5.56%	3.98%	0.12%	0.08%	Glycolysis to A-CoA	Pklr
			MP:0002925	abnormal cardiovascular development	5.24E-01	5.84E-01	1	18	861	21319	5.56%	4.04%	0.12%	0.08%	Glycolysis to A-CoA	Pfkm
			MP:0020000	abnormal response to antigen	5.24E-01	5.84E-01	1	18	861	21319	5.56%	4.04%	0.12%	0.08%	Glycolysis to A-CoA	Pklr
			MP:0000959	abnormal somatic sensory system morphology	5.24E-01	5.84E-01	1	18	862	21319	5.56%	4.04%	0.12%	0.08%	Glycolysis to A-CoA	Mpc1
			MP:0002161	abnormal fertility/fecundity	5.33E-01	5.92E-01	2	18	2068	21319	11.11%	9.70%	0.10%	0.08%	Glycolysis to A-CoA	Hk1, Pdha1
			MP:0001545	abnormal hematopoietic system physiology	5.36E-01	5.94E-01	2	18	2081	21319	11.11%	9.76%	0.10%	0.08%	Glycolysis to A-CoA	Pfkm, Pklr
			MP:0002163	abnormal gland morphology	5.36E-01	5.94E-01	3	18	3269	21319	16.67%	15.33%	0.09%	0.08%	Glycolysis to A-CoA	Gck, Hk3, Pfkm
			MP:0005397	hematopoietic system phenotype	5.40E-01	5.98E-01	5	18	5651	21319	27.78%	26.51%	0.09%	0.08%	Glycolysis to A-CoA	Gck, Hk1, Pdha2, Pfkm, Pklr
			MP:0004849	abnormal testis size	5.43E-01	5.99E-01	1	18	906	21319	5.56%	4.25%	0.11%	0.08%	Glycolysis to A-CoA	Pfkm

			MP:0005502	abnormal renal/urinary system physiology	5.47E-01	6.02E-01	1	18	917	21319	5.56%	4.30%	0.11%	0.08%	Glycolysis to A-CoA	Gck
			MP:0002882	abnormal neuron morphology	5.49E-01	6.04E-01	2	18	2129	21319	11.11%	9.99%	0.09%	0.08%	Glycolysis to A-CoA	Mpc1, Pdha1
			MP:0020846	abnormal cell motility	5.51E-01	6.05E-01	1	18	928	21319	5.56%	4.35%	0.11%	0.08%	Glycolysis to A-CoA	Hk2
			MP:0005367	renal/urinary system phenotype	5.57E-01	6.10E-01	2	18	2160	21319	11.11%	10.13%	0.09%	0.08%	Glycolysis to A-CoA	Gck, Hk1
			MP:0002058	neonatal lethality	5.62E-01	6.14E-01	1	18	955	21319	5.56%	4.48%	0.10%	0.08%	Glycolysis to A-CoA	Gck
			MP:0002208	abnormal germ cell morphology	5.90E-01	6.43E-01	1	18	1029	21319	5.56%	4.83%	0.10%	0.08%	Glycolysis to A-CoA	Pdha1
			MP:0002206	abnormal CNS synaptic transmission	5.97E-01	6.49E-01	1	18	1048	21319	5.56%	4.92%	0.10%	0.08%	Glycolysis to A-CoA	Vdac1
			MP:0001732	postnatal growth retardation	6.03E-01	6.53E-01	1	18	1067	21319	5.56%	5.00%	0.09%	0.08%	Glycolysis to A-CoA	Gck
			MP:0001919	abnormal reproductive system physiology	6.04E-01	6.53E-01	2	18	2342	21319	11.11%	10.99%	0.09%	0.08%	Glycolysis to A-CoA	Hk1, Pdha1
			MP:0005369	muscle phenotype	6.04E-01	6.53E-01	2	18	2343	21319	11.11%	10.99%	0.09%	0.08%	Glycolysis to A-CoA	Pdha1, Pfkm
			MP:0001146	abnormal testis morphology	6.06E-01	6.55E-01	1	18	1075	21319	5.56%	5.04%	0.09%	0.08%	Glycolysis to A-CoA	Pfkm
			MP:0000787	abnormal telencephalon morphology	6.08E-01	6.56E-01	1	18	1081	21319	5.56%	5.07%	0.09%	0.08%	Glycolysis to A-CoA	Pdha1
			MP:0002160	abnormal reproductive system morphology	6.13E-01	6.60E-01	2	18	2381	21319	11.11%	11.17%	0.08%	0.08%	Glycolysis to A-CoA	Pdha1, Pfkm
			MP:0005387	immune system phenotype	6.15E-01	6.61E-01	5	18	6076	21319	27.78%	28.50%	0.08%	0.08%	Glycolysis to A-CoA	Hk1, Hk3, Pdha2, Pfkm, Pklr
			MP:0013558	abnormal exocrine gland morphology	6.18E-01	6.62E-01	1	18	1109	21319	5.56%	5.20%	0.09%	0.08%	Glycolysis to A-CoA	Pfkm
			MP:0005452	abnormal adipose tissue amount	6.24E-01	6.68E-01	1	18	1127	21319	5.56%	5.29%	0.09%	0.08%	Glycolysis to A-CoA	Pfkm
			MP:0002085	abnormal embryonic tissue morphology	6.26E-01	6.68E-01	1	18	1132	21319	5.56%	5.31%	0.09%	0.08%	Glycolysis to A-CoA	Pdhb
			MP:0001731	abnormal postnatal growth	6.29E-01	6.71E-01	1	18	1143	21319	5.56%	5.36%	0.09%	0.08%	Glycolysis to A-CoA	Gck
			MP:0003635	abnormal synaptic transmission	6.33E-01	6.73E-01	1	18	1153	21319	5.56%	5.41%	0.09%	0.08%	Glycolysis to A-CoA	Vdac1
			MP:0021009	abnormal synaptic physiology	6.36E-01	6.74E-01	1	18	1162	21319	5.56%	5.45%	0.09%	0.08%	Glycolysis to A-CoA	Vdac1
			MP:0001544	abnormal cardiovascular system physiology	6.36E-01	6.74E-01	2	18	2477	21319	11.11%	11.62%	0.08%	0.08%	Glycolysis to A-CoA	Gck, Pdha1
			MP:0001929	abnormal gametogenesis	6.52E-01	6.90E-01	1	18	1213	21319	5.56%	5.69%	0.08%	0.08%	Glycolysis to A-CoA	Pdha1
			MP:0005389	reproductive system phenotype	6.55E-01	6.92E-01	3	18	3846	21319	16.67%	18.04%	0.08%	0.08%	Glycolysis to A-CoA	Hk1, Pdha1, Pfkm
			MP:0001570	abnormal circulating enzyme level	6.71E-01	7.07E-01	1	18	1278	21319	5.56%	5.99%	0.08%	0.08%	Glycolysis to A-CoA	Gck
			MP:0002752	abnormal somatic nervous system morphology	6.72E-01	7.07E-01	1	18	1280	21319	5.56%	6.00%	0.08%	0.08%	Glycolysis to A-CoA	Mpc1
			MP:0013325	abnormal male reproductive gland morphology	6.76E-01	7.10E-01	1	18	1293	21319	5.56%	6.07%	0.08%	0.08%	Glycolysis to A-CoA	Pfkm
			MP:0003633	abnormal nervous system physiology	6.88E-01	7.21E-01	2	18	2716	21319	11.11%	12.74%	0.07%	0.08%	Glycolysis to A-CoA	Hk2, Vdac1
			MP:0002081	perinatal lethality	7.00E-01	7.32E-01	1	18	1379	21319	5.56%	6.47%	0.07%	0.08%	Glycolysis to A-CoA	Gck
			MP:0002082	postnatal lethality	7.03E-01	7.34E-01	1	18	1391	21319	5.56%	6.52%	0.07%	0.08%	Glycolysis to A-CoA	Gck
			MP:0009205	abnormal internal male genitalia morphology	7.04E-01	7.34E-01	1	18	1394	21319	5.56%	6.54%	0.07%	0.08%	Glycolysis to A-CoA	Pfkm
			MP:0009198	abnormal male genitalia morphology	7.11E-01	7.38E-01	1	18	1421	21319	5.56%	6.67%	0.07%	0.08%	Glycolysis to A-CoA	Pfkm
			MP:0001145	abnormal male reproductive system morphology	7.11E-01	7.38E-01	1	18	1421	21319	5.56%	6.67%	0.07%	0.08%	Glycolysis to A-CoA	Pfkm
			MP:0001961	abnormal reflex	7.12E-01	7.38E-01	1	18	1425	21319	5.56%	6.68%	0.07%	0.08%	Glycolysis to A-CoA	Vdac1
			MP:0002135	abnormal kidney morphology	7.34E-01	7.60E-01	1	18	1513	21319	5.56%	7.10%	0.07%	0.08%	Glycolysis to A-CoA	Hk1
			MP:0000653	abnormal sex gland morphology	7.41E-01	7.65E-01	1	18	1539	21319	5.56%	7.22%	0.06%	0.08%	Glycolysis to A-CoA	Pfkm
			MP:0002572	abnormal emotion/affect behavior	7.43E-01	7.66E-01	1	18	1550	21319	5.56%	7.27%	0.06%	0.08%	Glycolysis to A-CoA	Dlat
			MP:0000003	abnormal adipose tissue morphology	7.48E-01	7.69E-01	1	18	1569	21319	5.56%	7.36%	0.06%	0.08%	Glycolysis to A-CoA	Pfkm
			MP:0005375	adipose tissue phenotype	7.59E-01	7.80E-01	1	18	1620	21319	5.56%	7.60%	0.06%	0.08%	Glycolysis to A-CoA	Pfkm
			MP:0002067	abnormal sensory capabilities/reflexes/nociception	7.66E-01	7.85E-01	1	18	1651	21319	5.56%	7.74%	0.06%	0.08%	Glycolysis to A-CoA	Vdac1
			MP:0000516	abnormal renal/urinary system morphology	7.73E-01	7.91E-01	1	18	1686	21319	5.56%	7.91%	0.06%	0.08%	Glycolysis to A-CoA	Hk1
			MP:0003492	abnormal involuntary movement	7.83E-01	8.00E-01	1	18	1735	21319	5.56%	8.14%	0.06%	0.08%	Glycolysis to A-CoA	Vdac1
			MP:0000428	abnormal craniofacial morphology	7.88E-01	8.04E-01	1	18	1761	21319	5.56%	8.26%	0.06%	0.08%	Glycolysis to A-CoA	Pdhb
			MP:0005382	craniofacial phenotype	7.93E-01	8.07E-01	1	18	1786	21319	5.56%	8.38%	0.06%	0.08%	Glycolysis to A-CoA	Pdhb
			MP:0010678	abnormal skin adnexa morphology	8.14E-01	8.27E-01	1	18	1901	21319	5.56%	8.92%	0.05%	0.08%	Glycolysis to A-CoA	Pfkl
			MP:0005416	abnormal circulating protein level	8.22E-01	8.34E-01	1	18	1951	21319	5.56%	9.15%	0.05%	0.08%	Glycolysis to A-CoA	Gck
			MP:0000313	abnormal cell death	8.36E-01	8.47E-01	1	18	2037	21319	5.56%	9.55%	0.05%	0.08%	Glycolysis to A-CoA	Pfkm
			MP:0008469	abnormal protein level	8.51E-01	8.60E-01	1	18	2140	21319	5.56%	10.04%	0.05%	0.08%	Glycolysis to A-CoA	Gck
			MP:0005386	behavior/neurological phenotype	8.68E-01	8.76E-01	4	18	6728	21319	22.22%	31.56%	0.06%	0.08%	Glycolysis to A-CoA	Dlat, Mpc1, Pfkm, Vdac1
			MP:0010832	lethality during fetal growth through weaning	8.97E-01	9.04E-01	1	18	2533	21319	5.56%	11.88%	0.04%	0.08%	Glycolysis to A-CoA	Gck
			MP:0004924	abnormal behavior	9.43E-01	9.48E-01	3	18	6446	21319	16.67%	30.24%	0.05%	0.08%	Glycolysis to A-CoA	Dlat, Pfkm, Vdac1
			MP:0001790	abnormal immune system physiology	9.67E-01	9.70E-01	1	18	3675	21319	5.56%	17.24%	0.03%	0.08%	Glycolysis to A-CoA	Pklr
			MP:0002066	abnormal motor capabilities/coordination/movement	9.89E-01	9.91E-01	1	18	4741	21319	5.56%	22.24%	0.02%	0.08%	Glycolysis to A-CoA	Vdac1

			MP:0000001	mammalian phenotype	1.00E+00	1.00E+00	18	18	21319	21319	100.00%	100.00%	0.08%	0.08%	Glycolysis to A-CoA	Dlat, Dld, Gck, Hk1, Hk2, Hk3, Hkdc1, Mpc1, Mpc2, Pdha1, Pdha2, Pdhb, Pdhx, Pfkl, Pfkm, Pklr, Pkm, Vdac1
