# Supplementary material for: Identification of Biochemical Pathways Responsible for Distinct Phenotypes Using Gene Ontology Causal Activity Models
Source: bioRxiv. 2023 May 25:2023.05.22.541760. Preprint. [Version 2] doi: 10.1101/2023.05.22.541760 (PMC10245817; doi:10.1101/2023.05.22.541760)
Supplement: Supplementary file 5 [file media-5.pdf]

Number of phenotypes per gene	hypoglycemia (MP:0000189)	increased liver triglyceride level (MP: 0009355)	decreased circulating triglyceride level (MP: 0002644)	increased liver glycogen level (MP: 0010400)	increased kidney glycogen level (MP: 0031003)	decreased granulocyte number (MP: 0000334)	abnormal midbrain development (MP:0003864)	abnormal gluconeogenesis (MP: 0003383)	increased circulating triglyceride level (MP: 0001552)	hepatic steatosis (MP: 0002628)
6	Cebpa	Cebpa				Cebpa		Cebpa	Cebpa	Cebpa
5		Bmal1	Bmal1			Bmal1		Bmal1	Bmal1	
4	Cebpb		Cebpb					Cebpb	Cebpb	
4		Pparg	Pparg						Pparg	Pparg
3		Ncoa3	Ncoa3	Ncoa3						
3	Foxa2	Foxa2							Foxa2	
3			Foxo1					Foxo1	Foxo1	
3	Klf15							Klf15	Klf15	
3		Nr1h4							Nr1h4	Nr1h4
3	Ppara								Ppara	Ppara
3								Clock	Clock	Clock
2		Ar							Ar	
2		Egr1					Egr1			
2	Foxa1		Foxa1							
2			Mlxipl	Mlxipl						
2			Hnf4a							Hnf4a
2		Mecp2							Mecp2	
2	Nfe2l2									Nfe2l2
2		Nr1h3								Nr1h3
2			Pitx3							Pitx3
2		Prdm16								Prdm16
2		Prox1								Prox1
2						Stat5b			Stat5b	
2						Trp53				Trp53
2						Zeb1			Zeb1	
2			Bcl6					Bcl6		
2									Stat3	Stat3
1			Aebp1							
1	Arx									
1						Bcl6b				
1						Bhlhe41				
1						Cebpe				
1						E2f4				
1			Elk4							
1							En1			
1						Etv6				
1						Fli1				
1						Gata1				
1						Gata3				
1							Gbx2			
1						Gfi1				
1						Gfi1b				
1							Grhl2			
1			Grhl3							
1						Gsx1				
1						Ikzf1				
1							Junb			
1	Men1									
1						Mitf				
1						Myb				
1						Mybl2				
1			Neurog3							
1			Nkx2-3							
1			Nr2c2							
1		Nr3c1								
1							Nr4a2			
1							Nr6a1			
1							Otx2			
1							Pax3			
1			Pax7							
1	Phox2a									
1						Plscr1				
1	Ptf1a									
1							Rest			
1						Spi1				
1			Srebf1							

1			Srebf2							
1						Stat5a				
1	Tbx19									
1	Tcf7l2									
1	Twist2									
1			Xbp1							
1	Zbtb20									
1						Zfp595				
1			Zfp704							
1								Foxo6		
1								Atf4		
1								Hif1a		
1									Plagl2	
1									Fosl1	
1									Pax5	
1									Nr2f2	
1									Ppard	
1									Hnf1b	
1										Ebf1
1										Arnt2
1										Atf6
1										Pou3f2
1										Nfe2l1
1										Hnf1a
1										Myc
1										Ahr
1										Ddit3
